# Twinned-magnesium metal negative electrodes enable reversible magnesium electrodeposition

**DOI:** 10.1038/s41467-026-77543-8

**Published:** 2026-09-04

**Authors:** Guodong Zou, Jingli Ren, Jinming Wang, Yong Sun, Weihao Yang, Tingting Niu, Jinyu Li, Carlos Fernandez, Jean-Jacques Gaumet, Qiuming Peng

**Affiliations:** 1 State Key Laboratory of Metastable Materials Science and Technology, Yanshan University, Qinhuangdao, China; 2 School of Pharmacy and Life Sciences, Robert Gordon University, Aberdeen, UK; 3 Laboratoire de Chimie et Physique, Approche Multi-échelles des Milieux Complexes, Institute Jean Barriol, Université de Lorraine, Metz, France

**Keywords:** Batteries, Batteries, Batteries

## Abstract

The favourable thermodynamic properties of magnesium make it a promising negative electrode for rechargeable Mg-metal batteries. However, parasitic side reactions and uncontrolled deposition at the unstable electrode/electrolyte interface severely hinder reversible electrodeposition. Here we demonstrate that a high density of twin defects via cyclic-multidirectional deformation markedly increases the diffusion rate and simultaneously suppresses interfacial reactions. Comprehensive morphological characterisation combined with theoretical calculations reveals that the twinned-magnesium negative electrode exhibits significantly enhanced kinetics, leading to a reduced dimensionless electrochemical Damköhler number and indicating that magnesium deposition becomes less diffusion-limited. Furthermore, reorganisation energy analysis based on Marcus–Hush–Chidsey kinetics, in conjunction with the battery overpotential, indicates that the twin structure effectively mitigates parasitic reactions between the negative electrode and the electrolyte, thereby significantly reducing the passivation effect and enabling long-term reversible magnesium electrodeposition. Consequently, the designed twinned-magnesium electrodes in an asymmetric coin cell achieve a Coulombic efficiency of 99.53% over 2000 cycles at 30 mA cm^−2^ with a capacity of 2 mAh cm^−2^, competitive with reported magnesium metal negative electrodes. This simple strategy has been successfully extended to the zinc metal system as a proof of concept, demonstrating the universal potential of twin engineering for achieving reversible metal electrodeposition.

## Abstract (web summary)

Twin boundaries in magnesium can enable fast charging. Here, the authors engineer these crystal defects to guide metal growth, enabling stable cycling at 30 mA cm^-2^. This design allows for rapid plating and stripping, paving the way for high-power batteries that charge in minutes.

## Introduction

Rechargeable magnesium (Mg)-metal batteries are promising candidates for “beyond lithium-ion” technologies owing to their use of metallic Mg negative electrodes^1,2^. Mg metal has several advantages, including a high theoretical capacity (3,883 mAh mL^−1^ and 2,205 mAh g^−1^), a relatively low redox potential (2.37 V vs. standard hydrogen electrode), natural abundance in the Earth’s crust (seventh most abundant element), and an intrinsic resistance to dendrite formation^3,4^. Nevertheless, the long-standing perception of dendrite-free Mg has been invalidated by recent results confirming dendritic growth during plating and nonuniform stripping in halide-based electrolytes^5^. Additionally, the interfacial reaction between Mg metal and halide electrolytes often produces poor ionic conducting surface layers, which severely limit its reversibility and hinder the practical utilisation of active Mg negative electrode^6^.

To overcome these challenges, it is crucial to suppress parasitic reactions and regulate Mg deposition, both of which are effective for extending battery lifespan. Currently, two primary anode-side strategies have been widely explored to ameliorate these issues. One common approach involves constructing artificial interfacial layers—such as graphite-based waterproof coatings—to prevent direct contact between Mg and trace water impurities. This approach effectively suppresses surface passivation, enhances ion transport kinetics, and consequently mitigates dendrite formation^7–9^. However, the high charge density and strong solvation of Mg^2+^ ions, combined with the dynamic nature of electrolytes (e.g., component consumption and concentration fluctuations), render these artificial layers prone to short-term effectiveness but long-term failure^10–12^. The other universal strategy involves developing three-dimensional Mg negative electrode frameworks, which facilitate planar Mg growth, improve charge transfer and reduce battery impedance because of their large surface area^13^. Nevertheless, these frameworks also limit ion diffusion and tend to induce top-surface deposition during high-capacity cycling, resulting in inefficient space utilisation and limited durability^14^. Hence, an ideal negative electrode structure should simultaneously facilitate rapid Mg^2+^ transport, suppress interfacial passivation, and enable uniform dense planar Mg deposition, increasing low interfacial resistance and long-term operational stability. More recently, the intrinsic manipulation of metals has been reported to be an effective strategy for tailoring the physicochemical properties of metal electrodes^15,16^. Several methods, such as introducing alloying elements or compounds and adjusting exposed facets, can markedly affect metal deposition morphology^17–19^. Specifically, metal compounds exhibit strong ionic affinity, which improves ion transport and enables favourable diffusion dynamics, resulting in more uniform and dendrite-free deposition^20^. Unfortunately, alloy phases often have poor stability because of element segregation or interfacial reactions^21^. On the other hand, although low-index surfaces such as Mg(0002) are theoretically advantageous^19,22^, their preparation requires strict control of growth parameters—including temperature, concentration, reaction time, and additive content—because even slight variations cause deviations in orientation. Therefore, developing a suitable Mg-based negative electrode remains a substantial technical challenge.

Twin defects, which act as mirror planes that divide the lattice into symmetrical domains, are regarded as highly stable because their interfacial energy is lower than that of grain boundaries^23^. Unlike other lithium or sodium metals, a hexagonal close-packed Mg metal has predominantly twinning deformation behaviour at low temperatures because of its limited independent slip systems and low twinning resistance^24,25^. In general, deformation twins are produced under sufficient stress in deformable materials without the need for heat treatment, which is a convenient process for preparing twins in Mg negative electrodes. Notably, the density of deformation twins increases with increasing deformation and cycling strain, especially for cycle-multiple-directional deformation^26^. Moreover, a high density of twins not only reduces diffusion barriers and improves ion transport but also increases corrosion resistance^27–29^. Thus, twin engineering might be a promising method for the development of high-performance Mg negative electrodes.

In this work, we systematically investigate the relationship between surface microstructure and electrochemical performance, highlighting how the classical twinning structure, induced by cyclic-multiple-directional deformation, improves Mg transport kinetics and enables uniform deposition. Simultaneously, the intrinsic corrosion resistance and stability of the twinned structures were exploited to construct the Mg negative electrode–electrolyte interface, thereby minimising side reactions. Comprehensive electrochemical analyses demonstrate that the twinned structure has a strong Mg affinity, reducing both the nucleation and diffusion barriers and facilitating favourable nucleation–growth. The effect of twinning during subsequent deposition is further supported by the Damköhler number. Furthermore, a comparison of the reconstruction energy, determined via Marcus–Hush–Chidsey kinetics, in conjunction with the battery overpotential, indicates that the twinned structure effectively suppresses interfacial passivation, even under high current density conditions. As a result, half-cells incorporating twinned Mg negative electrodes achieved a Coulombic efficiency of 99.53% after 2,000 cycles at 30 mA cm^−2^/2 mAh cm^−2^ and stable Mg plating/stripping for 800 hours at 10 mA cm^−2^/5 mAh cm^−2^. With respect to the full-cell configuration with a Mo_6_S_8_ positive electrode, 84.7% of the capacity was retained after 10,000 cycles. Furthermore, the successful extension of this twin-engineering strategy to Zn metal serves as a proof-of-concept validation, underscoring its potential generality for designing stable metallic negative electrodes.

## Results

### Characteristics of twinned-Mg negative electrodes

**Fig. 1:**
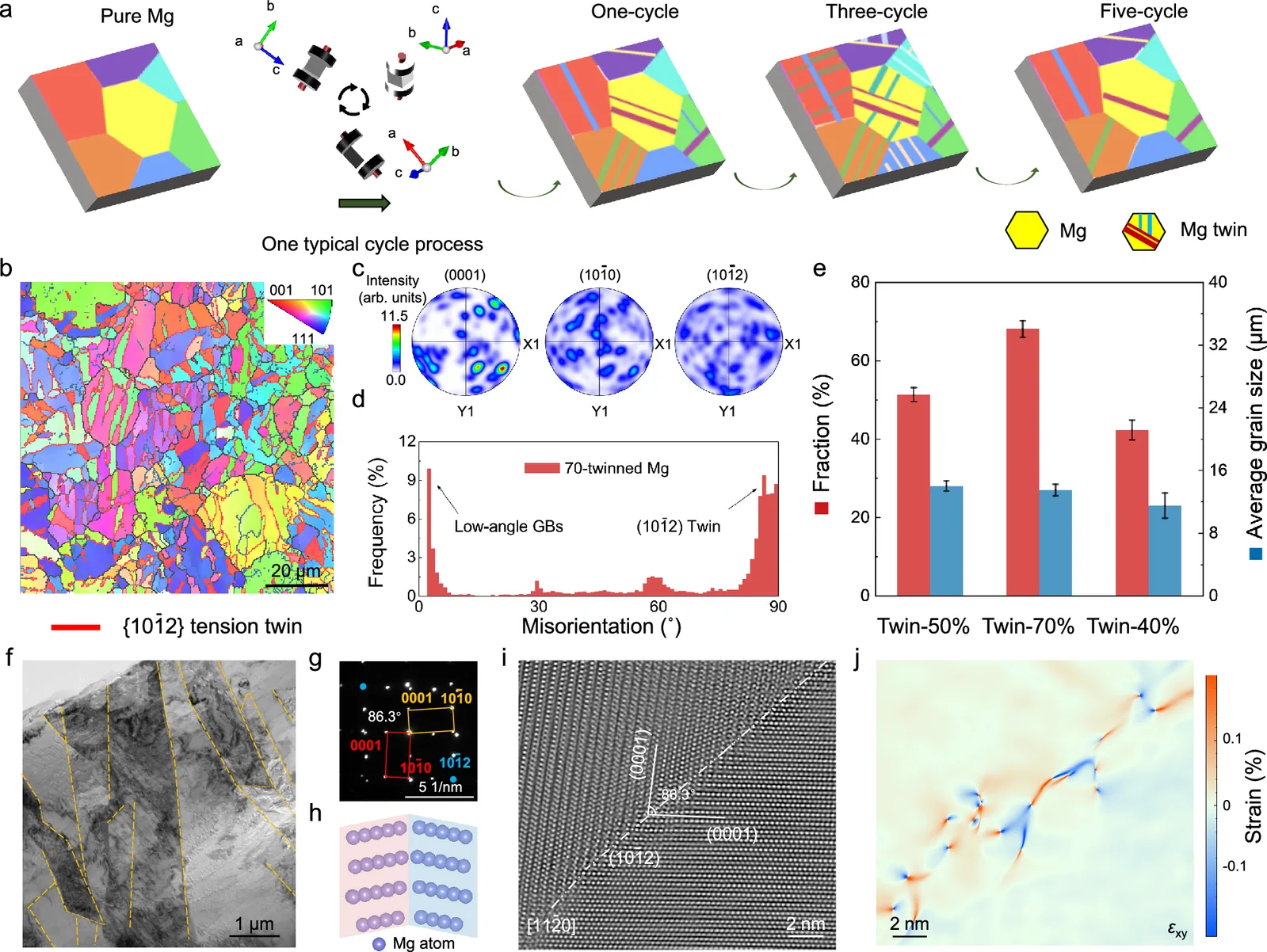
Structural characteristics of twinned Mg. **a** Schematic illustration of the preparation process of twinned-Mg anodes. **b** Orientation maps of 70-twinned Mg. **c** (0001), (10$\bar{1}$0) and (10$\bar{1}$2) pole figures of the 70-twinned Mg. **d** Statistical analyses of the misorientation shown in Fig. 1b. **e** Fraction of twins and average grain size of different negative electrodes. (Sample size (*n*) = 3 for fraction of twins; data are presented as mean values ± standard deviation (SD); Sample size (*n*) = 20 for average grain size; data are presented as mean values ± SD). **f** TEM image of the 70-twinned Mg. The yellow dashed line represents the twin boundary. **g** Corresponding SAED pattern of Fig. 1f. The red and yellow boxes represent the diffraction of the matrix and the twin, respectively, with mirror symmetry across the (10$\bar{1}$2) plane. **h** Crystal structure of the twinned Mg. **i** Atomic resolution HAADF image of the (10$\bar{1}$2) twins taken along the [11$\bar{2}$0] axis. The white dashed line represents the twin boundary. **j** Geometric phase analysis for HRTEM of twinned Mg. Source data for **d** and **e**are provided as a Source Data file.

A cyclic, multi-directional deformation route was employed to fabricate Mg metal negative electrodes enriched with twin structures (Fig. 1a). Specifically, on an electronic universal testing machine, pure Mg ingot samples were subjected to one, three, or five successive compressions along three principal axes. Owing to the intrinsic crystallography of hexagonal close-packed Mg, the geometric resistance to twinning shear is substantially greater than that to dislocation slip, indicating that twinning is the dominant deformation mechanism^24^. To further examine the twinned microstructure, multiscale characterisation was conducted. As evidenced by electron backscatter diffraction (EBSD) inverse pole figure (IPF), the undeformed Mg lacked twinning defects (Supplementary Fig. S1a, Fig. S2a). However, after a single-cycle compression, local stress concentrations reached the critical threshold, activating the (10$\bar{1}$2) twin system (Supplementary Fig. S1b, S2b). Moreover, (10$\bar{1}$2) twins were observed in nearly all the grains after three cycles of compression (Fig. 1b–d, Supplementary Fig. S3 and Supplementary Note 1). However, after five cycles of compression, obvious detwinning occurred, because slip replaced twinning as the dominant mechanism beyond the critical deformation limit (Supplementary Fig. S1c, S2c). Subsequently, according to quantitative EBSD analysis (Fig. 1e), twin boundaries account for approximately 70% of the total interface length (involving grain boundaries and twin boundaries) after three cycles of compression (defined as 70-twinned Mg), with the misorientation angles concentrated at ~86.3°. In contrast, the fractions of (10$\bar{1}$2) twin boundaries after one or five cycles of compression were only 50% and 40%, respectively (defined as 50-twinned Mg and 40-twinned Mg, respectively). Note that the average grain size remained nearly unchanged before and after deformation (Fig. 1e), suggesting that compression primarily affected the twin fraction rather than the grain size. Consequently, the 70-twinned Mg sample was selected for subsequent electrochemical tests to evaluate the effect of the twin microstructure on performance.

In detail, as shown in transmission electron microscopy (TEM) images, several deformation twins that occurred during the deformation process were confirmed in the 70-twinned Mg sample (Fig. 1f). Selected area electron diffraction (SAED) patterns from the same region exhibit typical mirror symmetry, with a deviation in orientation of 86.3° between the matrix and the twin domains, suggesting the presence of (10$\bar{1}$2) twins (Fig. 1g)^30^. This structural model of Mg twins is presented in Fig. 1h. High-resolution micrographs further revealed a well-defined interface between the Mg matrix and the twin boundary (Fig. 1i). The lattice fringes of Mg revealed a distinct mirror-image orientation relationship along the (10$\bar{1}$2) plane under the [11$\bar{2}$0] zone axis. Moreover, geometric phase analysis (GPA) images indicated that twins experience obvious strain fields and host abundant high-surface-energy atoms in 70-twinned Mg, indicating that twin boundaries may serve as electrochemically active sites (Fig. 1j)^31^.

### Structural evolution of Mg deposition

**Fig. 2:**
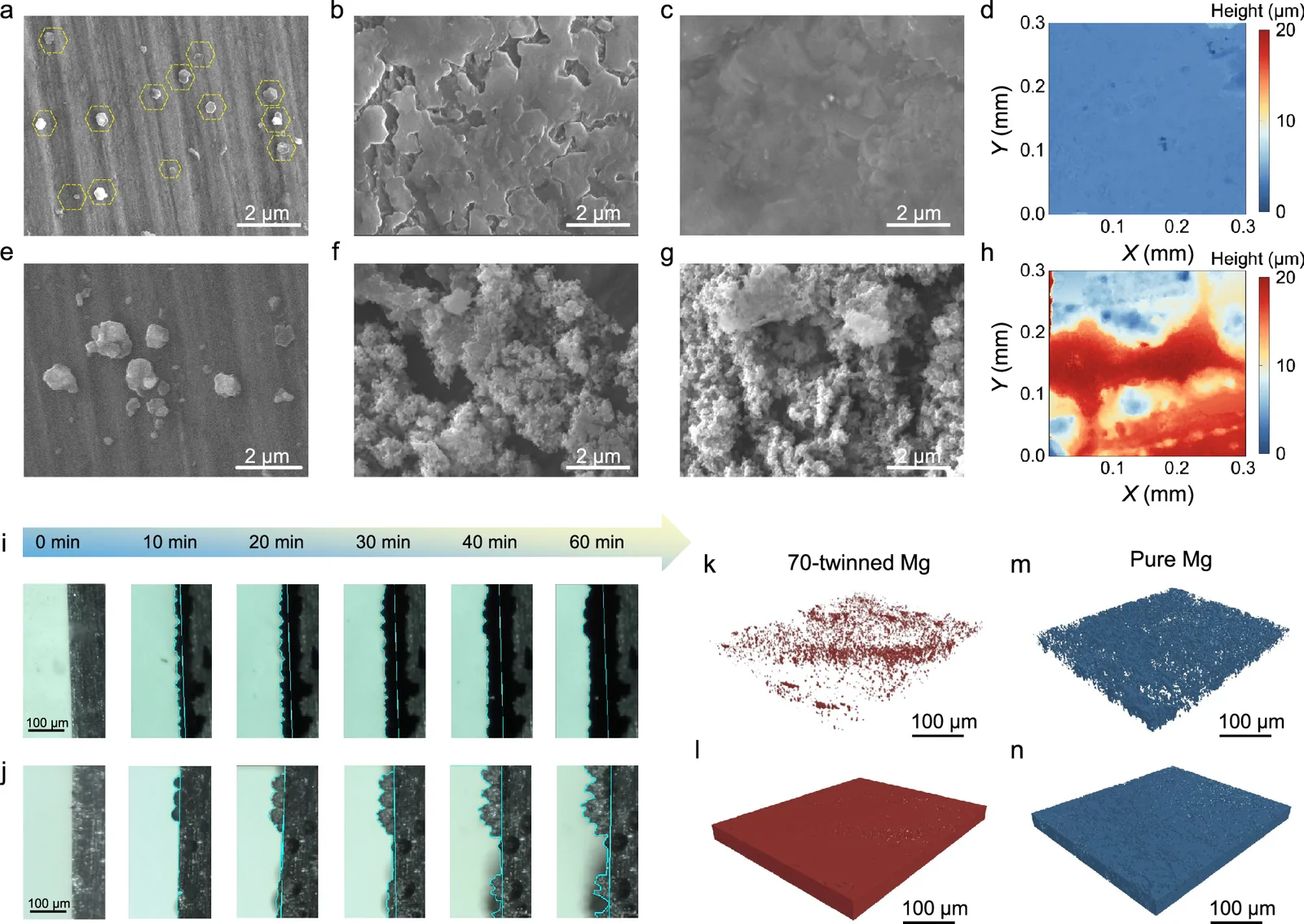
Mg nucleation and growth on different substrates. Uniform Mg deposition on a 70-twinned Mg negative electrode at a current density of 10 mA cm^−2^, with deposition densities of 0.1 mAh cm^‒2^ (**a**), 2 mAh cm^‒2^ (**b**), 10 mAh cm^‒2^ (**c**). **d** LCSM imaging of deposited morphology on the 70-twinned Mg (10 mA cm^‒2^, 5 mAh cm^‒2^). Non-uniform Mg deposition on pure Mg negative electrode at 0.1 mAh cm^‒2^ (**e**), 2 mAh cm^‒2^ (**f**), 10 mAh cm^‒2^ (**g**). **h** CLSM imaging of deposited morphology on the pure Mg surface (10 mA cm^‒2^, 5 mAh cm^‒2^). In situ optical images of Mg plating on the 70-twinned Mg (**i**) and the pure Mg (**j**) in separator-free symmetric cells at 10 mA cm^‒2^, recorded every 10 min. 3D reconstructed morphologies obtained by CT, showing porosity and crack distribution of the 70-twinned Mg electrodes (**k**, **l**) and the pure Mg electrodes (**m**, **n**) after deposition (10 mA cm^‒2^, 10 mAh cm^‒2^). All the tests were measured at 25 °C.

Mg deposition proceeds through two successive stages—initially nucleation followed by sustained growth—which are controlled by the substrate in accordance with electrode kinetics^32^. On twinned Mg negative electrodes, a well-ordered hexagonal morphology is observed at a low deposition capacity of 0.1 mAh cm^−2^. As deposition continues, the surface remains dendrite-free, and the hexagonal domains expand laterally (Fig. 2a–c and Supplementary Fig. S4). In contrast, pure Mg negative electrodes reveal markedly different evolution: even at 0.1 mAh cm^‒2^, nucleation occurs sparsely and in a highly disordered manner, resulting in an uneven surface and slow nucleation dynamics (Fig. 2e). When the deposition further increases to 10 mAh cm^‒2^, the layer becomes increasingly irregular and poorly adhered to the substrate (Fig. 2f-g and Supplementary Fig. S4). These behaviours are consistent across an extensive range of current densities (Supplementary Fig. S5). Additionally, the 70-twinned Mg surfaces exhibit compact, uniform deposits with thicknesses similar to the theoretical predictions, with measured deposit thicknesses of 6.09, 14.63, and 27.07 μm at 2, 5, and 10 mAh cm^‒2^, respectively (Supplementary Figs. S6, 7 and Supplementary Note 2). In contrast, the pure Mg electrodes experience nonuniform growth and develop uneven protrusions, which reduce the packing density and increase the surface energy. The measured deposit thicknesses are far greater than the calculated values (Supplementary Fig. S7). Notably, in the other three chloride-free electrolyte systems, the 70-twinned Mg electrode consistently exhibits markedly more uniform deposition than the pure Mg electrode (Supplementary Fig. S8). The resulting surface morphology is smoother and denser, showing trends highly consistent with those observed in the APC electrolyte. Therefore, the enhanced Mg deposition behaviour primarily originates from the intrinsic regulatory effect of the twin structure within the Mg electrode, rather than from the specific chemical characteristics of the APC electrolyte.

Furthermore, surface roughness measurements of Mg deposits at 5 mAh cm^‒2^, obtained with a laser confocal scanning microscope (LCSM), further confirm these trends (Fig. 2d, h). The 70-twinned Mg electrode shows smooth and homogeneous deposits, whereas the pure Mg electrode develops pits and protrusions up to 20 μm in depth. These striking differences are attributable to variations in Mg adsorption and surface diffusion kinetics, which are discussed in subsequent sections. Direct observation with in situ optical microscopy under 10 mA cm^‒2^ provides further insight (Fig. 2i, j). Specifically, the pure Mg electrode rapidly formed random protrusions that evolved into irregular bumps after 60 min. Conversely, the 70-twinned Mg electrode, with its exposed (0002) facets and nanosheet array architecture, facilitated the growth of a compact, uniformly distributed nucleation layer (Supplementary Fig. S9)^19^. This stable morphology throughout plating highlights the role of substrate optimisation and surface structuring in guiding Mg nucleation and deposition behaviour.

To further elucidate the three-dimensional structural characteristics of plated Mg without damaging its internal microstructure during cell disassembly, X-ray computed tomography (CT) was employed to analyse the electrode after deposition with an areal capacity of 10 mAh cm^‒2^. CT imaging relies on the differential attenuation of X-ray intensity through materials of various densities, enabling the spatial distribution of pores and bulk Mg to be distinguished on the basis of luminance contrast^33^. Reconstructed 3D images of the pore distribution are shown in Fig. 2k–n, revealing that the 70-twinned Mg has the lowest porosity of ~0.78%, whereas the Mg electrode has the highest porosity, approaching 17.9%. These results reveal that the twinned Mg structure effectively suppresses pore formation during Mg plating, reducing the electrode surface area exposed to the electrolyte and mitigating undesirable side reactions.

### Long-term reversible Mg plating/stripping performance

**Fig. 3:**
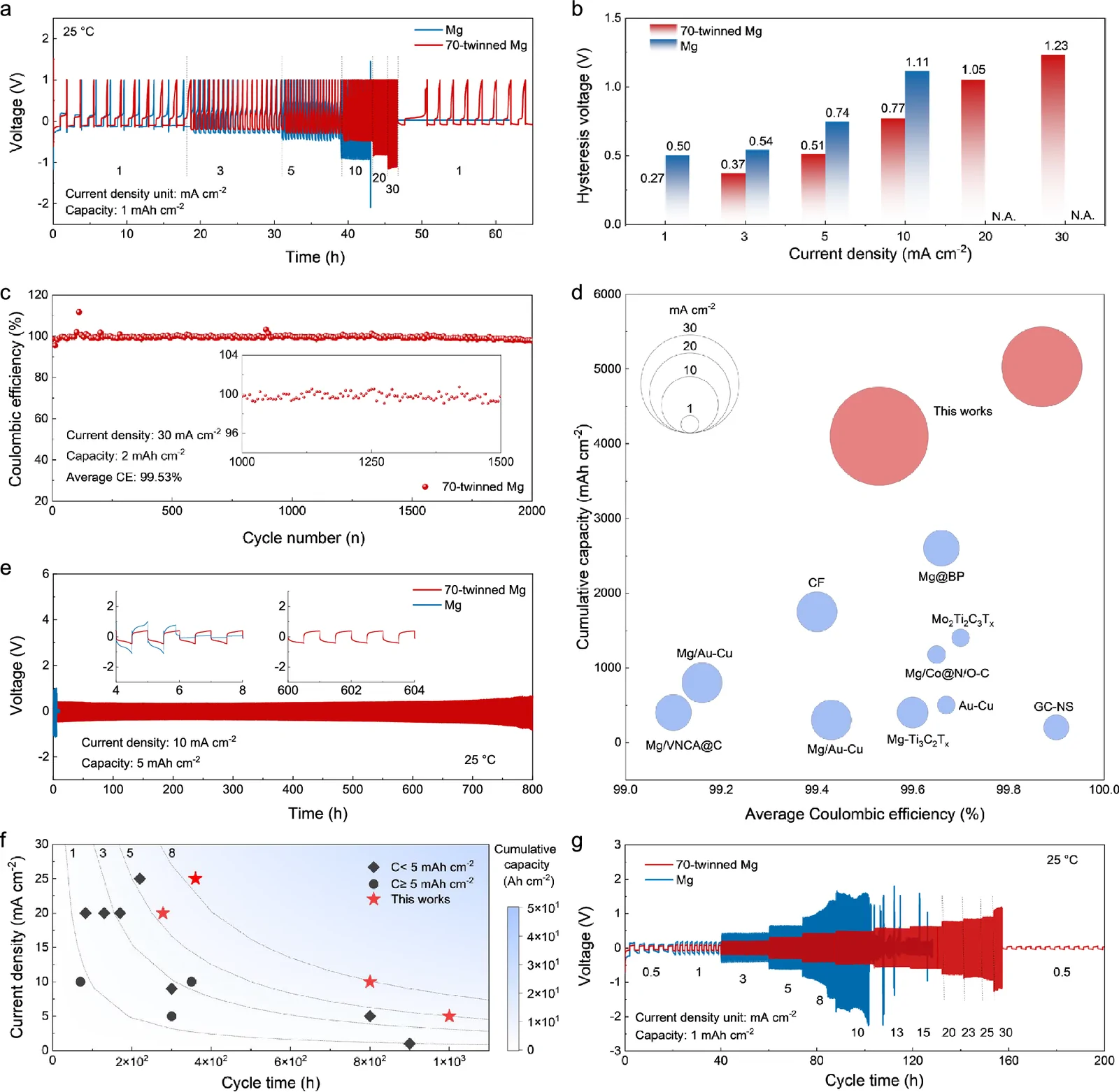
Asymmetric and symmetric cell performances. **a** Rate performance of different cells with the capacity of 1 mAh cm^‒2^. **b** Hysteresis voltage corresponding to various current densities for the rate performance of different cells. **c** The CE values of different cells at 30 mA cm^−2^ and 2 mAh cm^−2^. **d** A comparison of CE performance in recent demonstrations with our work. The source of the literature data shown in this figure can be found in Supplementary Information, Table S1. **e** Cyclic stability of symmetric cells at 10 mA cm^‒2^/5 mA h cm^‒2^. **f** Symmetric cells performance comparison between 70-twinned Mg cells and recently reported Mg cells using different strategies. The source of the literature data shown in this figure can be found in Supplementary Information, Table S2. **g** Rate performance of the pure Mg and 70-twinned Mg symmetric cells at different current densities from 0.5 to 30 mA cm^‒2^ with the same capacity of 1 mAh cm^‒2^. All the tests were measured at 25 °C. Source data are provided as a Source Data file.

To evaluate the effect of the morphology of the twinned Mg deposit on the electrochemical performance, we galvanostatically measured the coulombic efficiency (CE) of Mg plating/stripping in asymmetric cells on the basis of a Cu substrate with an areal capacity of 1 mAh cm^‒2^. The 70-twinned Mg||Cu half-cell exhibited stable Mg plating/stripping even at a current density of 30 mA cm^‒2^, demonstrating the ability of the Mg-twinned structure to stabilise Mg electrodeposition across an extensive current range (Fig. 3a). In turn, The Mg||Cu half-cell short circuits at 20 mA cm^‒2^ and shows higher overpotentials, indicating the inhomogeneous nature of Mg plating/stripping (Fig. 3b)^34^. Notably, even when the areal capacity of the 70-twinned Mg||Cu half-cell is increased to 2 mAh cm^‒2^, the battery maintains stable Mg plating/stripping for more than 2000 cycles, with an average CE of 99.53% at 30 mA cm^‒2^, and for more than 2500 cycles, with an average CE of 99.87% at 20 mA cm^‒2^ (Fig. 3c and Supplementary Fig. S10). These results indicate that the 70-twinned Mg electrode is competitive with reported Mg negative electrodes (Fig. 3d and Supplementary Table S1), highlighting the potential of twinned structures for high-power Mg batteries.

To further determine the effect of the twin structure on the electrochemical performance, symmetric coin cells were evaluated. With increasing twin fractions, the Mg plating/stripping lifespan significantly increased, indicating that a higher density of twins effectively stabilised Mg deposition (Fig. 3e and Supplementary Fig. S11). The 70-twinned Mg||70-twinned Mg symmetric cell achieved the longest stability, sustaining 800 hours of operation at 10 mA cm^‒2^ and 5 mAh cm^‒2^, outperforming the 50-twinned Mg, 40-twinned Mg, and pure Mg cells. This increased stability is attributable to the homogeneous plating/stripping morphology preserved by the 70-twinned Mg electrodes, which mitigates uneven growth and interfacial degradation (Supplementary Fig. S12). In addition, the 70-twinned Mg electrode still retains a dense distribution of twinned structure after 50 cycles at a current density of 10 mA cm^−2^ and a capacity of 5 mAh cm^‒2^ (Supplementary Fig. S13). More importantly, even under more stringent conditions, the 70-twinned Mg cells maintained long-term stable Mg plating/stripping, competitive with reported Mg metal electrodes (Fig. 3f, Supplementary Fig. S14 and Supplementary Table S2). Attractively, the 70-twinned Mg||70-twinned Mg symmetric cell had favourable rate capability under increasing current densities up to 30 mA cm^‒2^ (Fig. 3g), whereas the Mg | |Mg cell showed severe voltage fluctuations and large overpotentials. Moreover, the satisfactory rate performance of the 70-twinned Mg||70-twinned Mg symmetric cells can also be maintained in the other three chlorine-free electrolytes (Supplementary Fig. S15).

### Nucleation and growth mechanism of Mg

**Fig. 4:**
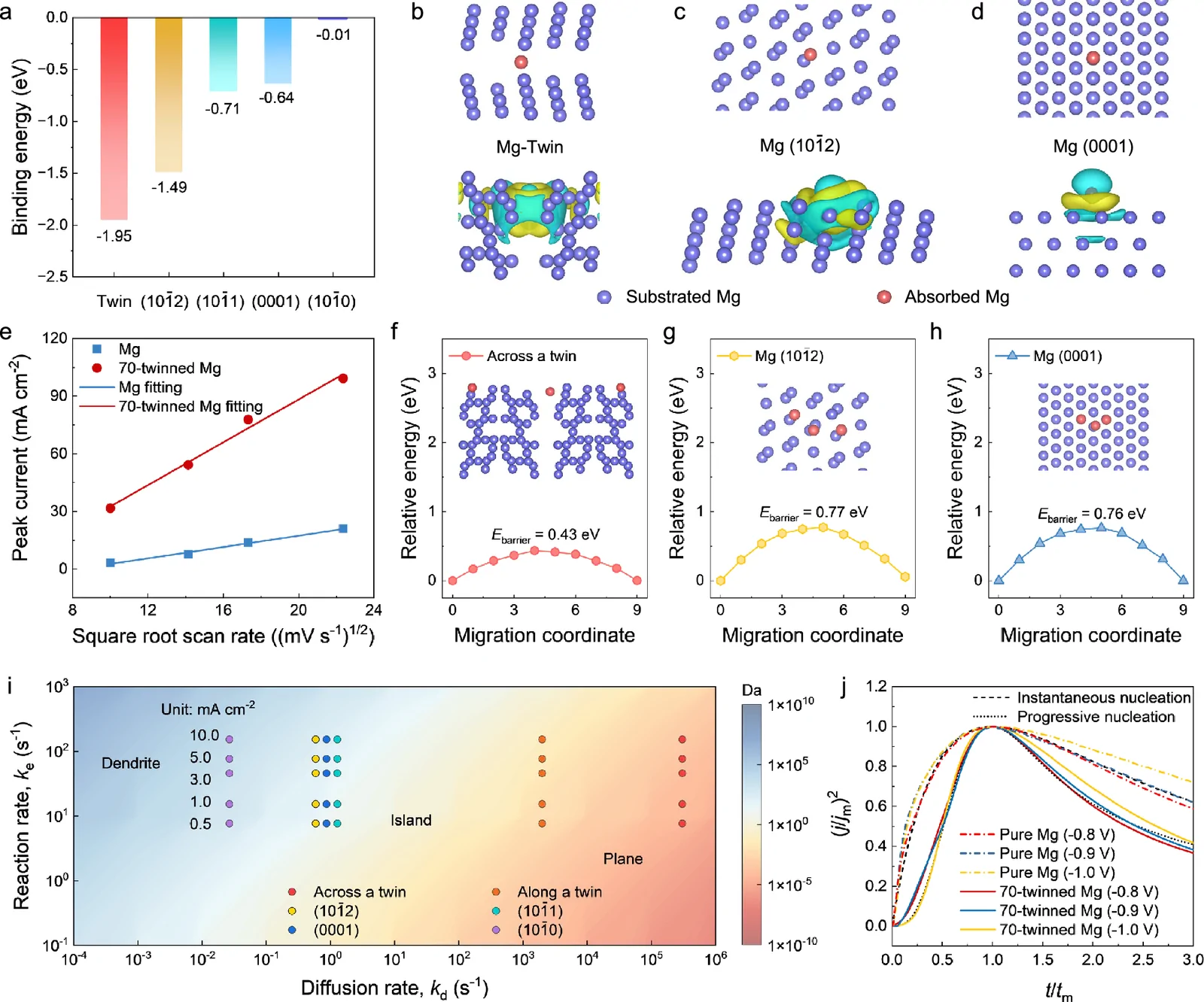
Magnesiumphilic mechanism. **a** Binding energies of Mg adatoms on Mg-Twin structure, Mg(10$\bar{1}$2), Mg(10$\bar{1}$1), Mg(0001), and Mg(10$\bar{1}$0). Geometry-optimized atomic configuration and differential charge density of Mg adsorbed Mg-Twin structure surface (**b**), Mg(10$\bar{1}$2) (**c**), and Mg(0001) (**d**). **e** The dependence of the peak current on the square root of the scan rate. Energy profiles for the diffusion process of Mg on Mg twin structure (**f**), Mg(10$\bar{1}$2) (**g**), and Mg(0001) (**h**). Insets: schematic representations of the corresponding diffusion pathways for twinned Mg structure, Mg(10$\bar{1}$2), and Mg(0001). **i** Deposition morphology phase map as a function of Damköhler number. **j** Plots of *t*/*t*_m_ and (*j*/*j*_m_)^2^ in comparison with theoretical 3D models for a Mg cell using Mg and 70-twinned Mg electrodes. Source data for (**a**) and (**e**–**j**) are provided as a Source Data file.

The transformation of Mg ions into metallic Mg is essentially controlled by nucleation and crystal growth, which determines the morphology of Mg deposits and strongly affects the resulting electrochemical performance^35^. To elucidate the role of electrode composition in this process, we evaluated the interaction energies and diffusion barriers of Mg on several representative low-index planes—Mg(10$\bar{1}$2), Mg(10$\bar{1}$1), Mg(0001), Mg(10$\bar{1}$0), and the Mg twin surface—for both the pure Mg electrode and the 70-twinned Mg electrode. Notably, Mg atoms preferentially adsorb at twin sites rather than on other crystal planes because of their stronger binding energy (E_twin_ = –1.95 eV) (Fig. 4a). Furthermore, obvious charge transfer occurs from Mg to the twin structure (Fig. 4b–d and Supplementary Fig. S16), as indicated by delocalised electrons distributed throughout the conjugation structure^36,37^. In contrast, electrons remain localised at the adsorption sites on the defect-free Mg surface, decreasing the interfacial coupling. To examine the effect of the interfacial layer on Mg transport, linear sweep voltammetry (LSV) was performed at scan rates of 100–500 mV s^‒1^ (Supplementary Fig. S17). The diffusion behaviour can theoretically be described by the Randles–Ševčík equation^38^:

1 $${J}_{p}=\left(2.65\times {10}^{5}\right){{{{\rm{n}}}}}^{\frac{1}{2}}{{SC}}_{{Mg}}^{*}{D}_{{Mg}}^{*}{{{{\rm{\nu }}}}}^{\frac{1}{2}}$$

where *J*_p_ represents the peak current, n denotes the number of electrons, S indicates the electrode area, *D*_Mg_ represents the Mg-ion diffusion coefficient, *C*_Mg_ denotes the Mg-ion concentration, and *ν* indicates the scan rate. As expressed in Eq. (1), *D*_Mg_ is directly proportional to the slope of the *J*_p_/*ν*^1/2^ plot. By analysing the variation in *J*_p_ at scan rates of 100–500 mV s⁻¹, the corresponding slopes were obtained (Fig. 4e). The results reveal that the slope of the 70-twinned Mg electrode is greater than that of the pure Mg electrode, indicating a markedly increased Mg-ion diffusion rate in the 70-twinned Mg electrode^39^. In addition, the calculated diffusion barriers reveal that the energy requirement for Mg migration near the twin boundary is significantly reduced (Fig. 4f–h and Supplementary Fig. S18). Specifically, the diffusion barriers for Mg migration across and along the twin boundary are 0.43 eV and 0.56 eV, respectively, which are substantially lower than those calculated for bulk Mg, namely, 0.77 eV for Mg (10$\bar{1}$2), 0.76 eV for Mg (0001), 0.75 eV for Mg (10$\bar{1}$1), and 0.85 eV for Mg (10$\bar{1}$0). These results indicate that the twin boundary acts as an energetically favourable diffusion pathway. These kinetic advantages directly affect deposition behaviour, because the morphology of Mg deposits is determined by the competition between charge-transfer reactions and surface diffusion^40,41^. When Mg transport is limited and the reaction rate is relatively high, dendritic growth becomes prevalent. Conversely, when surface diffusion proceeds more rapidly than the electrochemical reaction does, planar deposition occurs. This balance can be quantitatively assessed on the basis of the nondimensional electrochemical Damköhler number (*D*_a_)^5,42^, which is defined as the ratio of the electrochemical reaction rate (*k*_e_) to the surface diffusion rate (*k*_d_) (Supplementary Note 3). For several low-index crystal planes in pure Mg, the *D*_a_ value is significantly greater than 1, indicating that electrochemical reactions dominate surface diffusion, favouring dendrite formation (Fig. 4i and Supplementary Table S3). Notably, we further obtain four Mg single-crystal surfaces with well-defined orientations and correlate the observed Mg deposition behaviours on these surfaces with the corresponding *D*a number results. This one-to-one comparison more clearly validates the reliability of the *D*a analysis in describing deposition kinetic differences among different crystallographic planes (Supplementary Figs. S19 and S20 and Supplementary Note 4). In contrast, the presence of twinned structures in twinned Mg substantially alleviates diffusion limitations, resulting *D*a values much smaller than 1 and thereby promoting uniform, planar Mg deposition (Fig. 4i and Supplementary Note 4)^5,43^.

The electrocrystallisation of new phases is strongly controlled by overpotential and can be directly tracked through current transients^20^. Hence, chronoamperometry (CA) was employed to investigate the early-stage nucleation kinetics of Mg deposition (Supplementary Figs. S21–23). The results indicate that all measured transients reveal a distinct peak current (*j*_m_) that is characteristic of a 3D nucleation process^44^. To better assess the nucleation mechanism, the CA transients obtained at closely spaced overpotentials (−0.8, −0.9, and −1.0 V vs. Mg/Mg^2+^) were normalized with respect to the peak current density (*j*ₘ) and the corresponding peak time (*t*ₘ), and subsequently compared with the theoretical predictions of the Scharifker–Hills (S–H) model (Supplementary Note 5)^45^. Notably, prior to mechanistic interpretation, the applicability of the S–H model was explicitly verified by evaluating the diagnostic parameter (*j*_max_)^2^*t_max_ across the different overpotentials^46^. For both pure Mg and 70-twinned Mg electrodes, (*j*_max_)^2^*t_max_ remains within a narrow and consistent range, confirming that the experimental conditions satisfy the diffusion-controlled nucleation assumption inherent to the S–H model (Supplementary Table S4)^47–49^. Subsequently, according to this model, nucleation can proceed in two modes: instantaneous (3DI), in which all nuclei form at the onset, or progressive (3DP), in which nuclei gradually form. Based on the normalized transients, Mg deposition on pure Mg is predominantly described by the 3DI nucleation mode (Fig. 4j). It should be emphasized, however, that the S–H model is specifically intended to describe early-stage nucleation kinetics and does not directly capture long-term growth processes or predict the final deposition morphology. Nevertheless, early-stage nucleation characteristics can be indirectly linked to subsequent growth behaviour^20,50,51^. In particular, the 3DI mode observed on pure Mg electrodes implies rapid and highly localized nucleation at a limited number of active sites, which can result in accelerated local ion consumption and rapid depletion of available nucleation sites^52^. COMSOL simulations (Supplementary Fig. S24) further demonstrate that, with such 3DI-like nucleation characteristics, these initial inhomogeneities are prone to being magnified during subsequent growth, thereby increasing morphological instability^50^. In contrast, the normalized transients of the 70-twinned Mg electrode tend to exhibit the 3DP nucleation mode (Fig. 4j). This behaviour originates from the internal twin structures, whose strong magnesiophilicity supplies a large number of nucleation sites that can be progressively activated^20^. The resulting uniformly distributed nuclei effectively regulate subsequent Mg growth, leading to a more homogeneous deposition process, in good agreement with the experimentally observed smoother and denser Mg layers (Fig. 2a-c).

### Interface passivation suppression mechanism

**Fig. 5:**
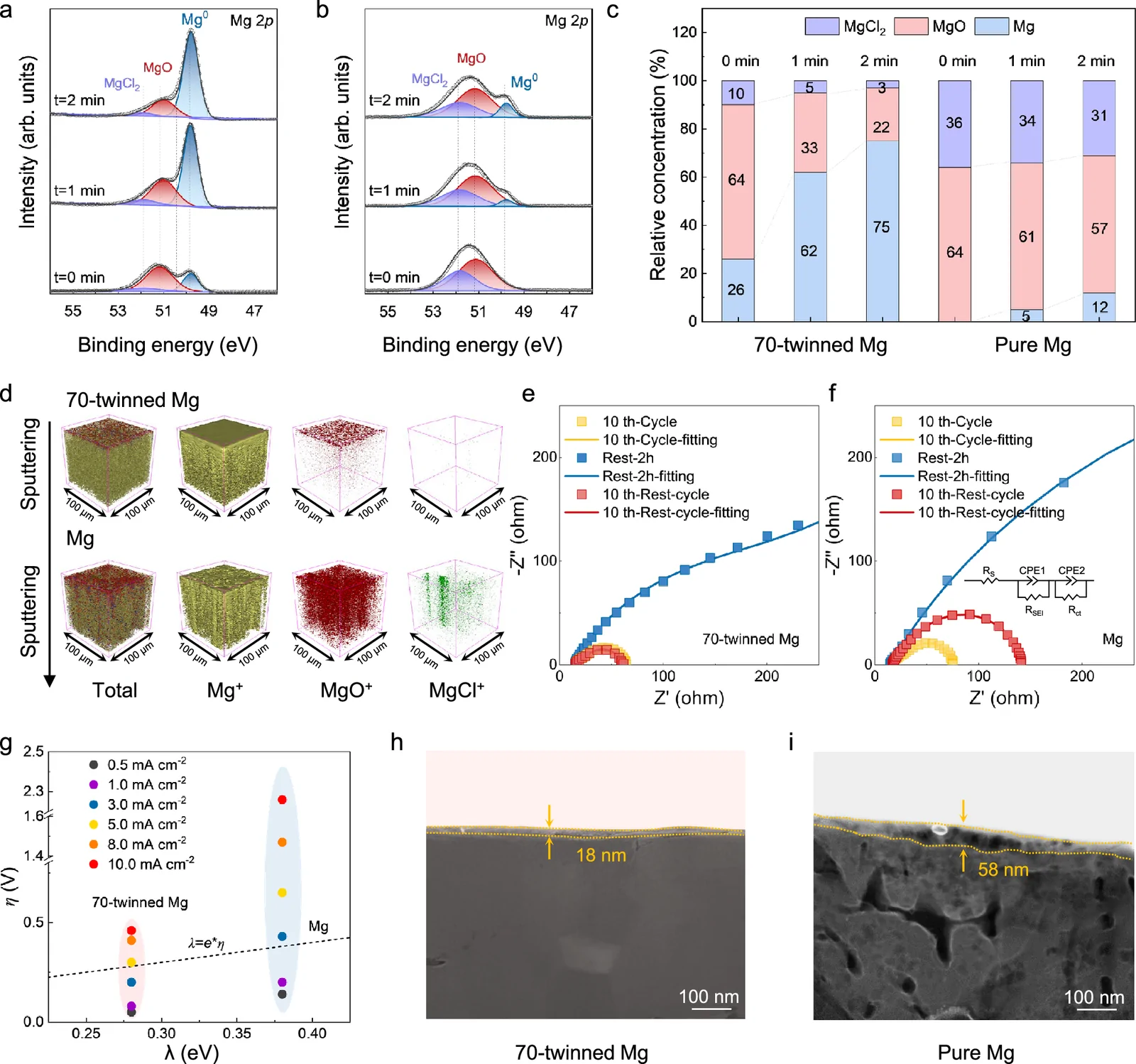
Interface analysis and characterisation. High-resolution XPS spectra of Mg 2*p* of 70-twinned Mg (**a**) and pristine Mg (**b**) electrodes after Mg plating/stripping (10 cycles at 10 mA cm^‒2^). The black dot plot represents the original data, the black line represents the total fitted data, and the purple, red, and blue areas represent MgCl_2_, MgO, and Mg^0^, respectively. **c** The relative concentrations of decomposition species measured on the surface after Mg plating/stripping at sputtering depths of 0, 1, and 2 min. **d** 3D rendering for the Mg^+^, MgO^+^, and MgCl^+^ secondary ions and the overlay in the ToF-SIMS tested for the interfaces formed on the Mg and 70-twinned Mg electrodes. The EIS spectra of a 70-twinned Mg||70-twinned Mg (**e**) and a Mg ||Mg cell (**f**) measured after Mg plating/stripping (10 cycles), resting for 2 hoursand re-plating/stripping period, under conditions of 10 mA cm⁻^2^ and 1 mAh cm⁻^2^. The inset corresponds to the fitted circuit. **g** The correlation between overpotential (*η*) and reorganization energy (*λ*) for different reaction modes of the Mg negative electrodes. The dashed line represents when the *η* for electron transfer is equal to *λ*. TEM images of the interface and deposited Mg after Mg plating/stripping for the 70-twinned Mg (**h**) and pure Mg (**i**) electrodes. Source data for **a**–**c** and **e**–**g** are provided as a Source Data file.

Owing to its relatively high activity, Mg metal readily generates interfacial compounds such as MgO, which has poor electrical conductivity and thus hinders charge transfer^53^. To elucidate the stabilising effect of twinned-Mg, cycled Mg metal batteries were characterised by X-ray photoelectron spectroscopy (XPS). As illustrated in Fig. 5a–c and Supplementary Fig. S25, the interfacial composition differs substantially between the pure Mg electrode and the twinned Mg electrode. For the pure Mg electrode, the Mg 2*p* and Cl 2*p* spectra reveal a relatively high abundance of MgO and MgCl_2_, with minimal variation in sputtering depth. This finding indicates continuous electrolyte decomposition, which not only fails to protect the electrode surface but also increases structural degradation of the deposited Mg. In contrast, the interface on the 70-twinned Mg electrode contains considerably lower amounts of MgO and MgCl_2_, with their concentrations gradually decreasing with depth. Notably, the passivation products on the surface of the pure Mg electrode increased significantly after 50 cycles compared to after 10 cycles (Supplementary Fig. S26). However, for the 70-twinned Mg negative electrode, the interfacial chemical composition and the relative proportions of surface species remain essentially unchanged after 10, 50, and 100 cycles (Supplementary Fig. S27). These findings suggest the formation of a thin, self-limiting interphase that equilibrates with the electrolyte and freshly deposited Mg. The limited accumulation of decomposition products reduces passivation, facilitates more complete Mg stripping, suppresses localised deposition, and results in a compact crystalline morphology after Mg plating/stripping^54^. Consequently, the 70-twinned Mg electrode enables more reversible and stable Mg deposition behaviour. To confirm the reduction of electrolyte-derived interphases on 70-twinned Mg electrodes, depth-profiling time-of-flight secondary ion mass spectrometry (TOF-SIMS) was conducted. For the 70-twinned Mg symmetric cells (Fig. 5d), MgO⁺ species were detected only at the surface of deposits with an areal capacity of 5 mAh cm^‒2^ at a current density of 10 mA cm^‒2^ but were absent in the bulk. The MgCl⁺ species exhibited a similar behaviour but with a lower intensity. These observations, in agreement with the results of the XPS analysis, indicate a significant reduction in interfacial products during electroplating.

To further confirm that the twinned structure effectively suppresses interfacial passivation, long-term in situ electrochemical impedance spectroscopy (EIS) measurements were performed. Specifically, both cells exhibited an increase in interfacial resistance during the remainder of the period, suggesting the formation of a transient interphase on the freshly deposited Mg surface^55^. Notably, upon reapplication of the electrodeposition/stripping current, the 70-twinned Mg electrode reverted to its initial state, indicating reversible interfacial behaviour (Fig. 5e and Supplementary Table S5), and maintained a stable interfacial impedance even after 100 cycles (Supplementary Fig. S28). These observations indicate that 70-twinned Mg electrodes sustain dynamic interfacial chemistry with rapid charge-transfer kinetics during Mg plating/stripping. More importantly, the interphase formed during the static equilibration of 70-twinned Mg does not hinder the ordered deposition of a close-packed hexagonal layered structure. However, the impedance of pure Mg symmetric cells remained significantly higher than the initial value after Mg plating/stripping, revealing sluggish charge-transfer processes during deposition and stripping (Fig. 5f and Supplementary Table S5), which further deteriorated after 100 cycles (Supplementary Fig. S28). Concurrently, widespread bulk interphase formation obstructs ion transport because of the sluggish migration of Mg^2+^ through the interphase in pure Mg electrodes, resulting in highly uneven morphologies^55^. Additionally, at the theoretical mechanism level, DFT calculations are used to demonstrate the crucial roles of electrode potential and twin structure in interfacial evolution^56,57^. Considering that tetrahydrofuran (THF) serves as the solvent in the APC electrolyte and [MgCl·5THF]^+^ species act as its active components, we selected [MgCl·5THF]⁺ and THF as two representative models for our calculations. Based on these models, we systematically compared the reaction thermodynamics on pure Mg and twinned Mg under different electrode potentials (Supplementary Figs. S29–S32). At the same electrode potential, the Gibbs free energy for the reduction of [MgCl·5THF]^+^ and THF on twinned Mg is significantly higher than that on pure Mg (ΔG_twinned-Mg_ > ΔG_pure-Mg_), indicating that the twin structure intrinsically increases the energy barrier for the formation of interfacial passivation products (MgO and MgCl_2_). In addition, for the same negative electrode material, increasing the electrode potential markedly increases the Gibbs free energy of the reduction reaction, thereby suppressing interfacial side reactions. Notably, this “potential-interfacial reaction synergistic suppression effect” on twinned Mg provides clear thermodynamic evidence that the twin structure is conducive to interfacial passivation suppression.

In addition, to explain the favourable charge-transfer behaviour of the 70-twinned Mg electrode compared with that of the pure Mg electrode, the electron transfer from the electrode to Mg^2+^ ions was investigated. In electrochemical reactions occurring at the electrode, the overpotential (η) corresponds to the shift in the Fermi level of the electrode required for each electron transfer, whereas the reorganisation energy (λ) represents the energetic cost of rearranging the solvation shell to accept the electron^58,59^. The λ values were extracted from Tafel plots of the Mg negative electrode by fitting with Marcus–Hush–Chidsey kinetics (Supplementary Fig. S33). The fitting equation is expressed as follows:

2 $$i=A{\int }_{\!\!\!\!-\infty }^{+\infty }exp \left\{-\frac{{\left(x-\lambda \pm \widetilde{\eta }\right)}^{2}}{4\lambda }\right\}\frac{{dx}}{1+exp \left\{x\right\}}$$

where A represents the preexponential factor, $\widetilde{\eta}$ represents the dimensionless overpotential, and i denotes the current density. The fitted *λ* values were subsequently converted to unscaled reorganisation energies (eV). Notably, the fitted *λ* is correlated with *η* in two distinct regions (Fig. 5g). With respect to the 70-twinned Mg, *η* only slightly differs from the theoretical *λ* for Mg²⁺ redox, indicating that the main reaction is Mg deposition/stripping with only minor parasitic processes^60^. However, the *η* for the pure Mg electrode differs considerably from the theoretical *λ*, indicating that electrolyte decomposition dominates. This persistent decomposition damages the electrode interface, resulting in sluggish charge-transfer kinetics and thicker interphases^59,61^. In addition, transmission electron microscopy (TEM) directly confirms these findings: the interfacial layer on the 70-twinned Mg electrode is thinner (~18 nm) and significantly denser (Fig. 5h), whereas the interphase layer on the pure Mg electrode reaches a thickness of ~58 nm and presents porous and uneven features (Fig. 5i).

### Full-cell performance

**Fig. 6:**
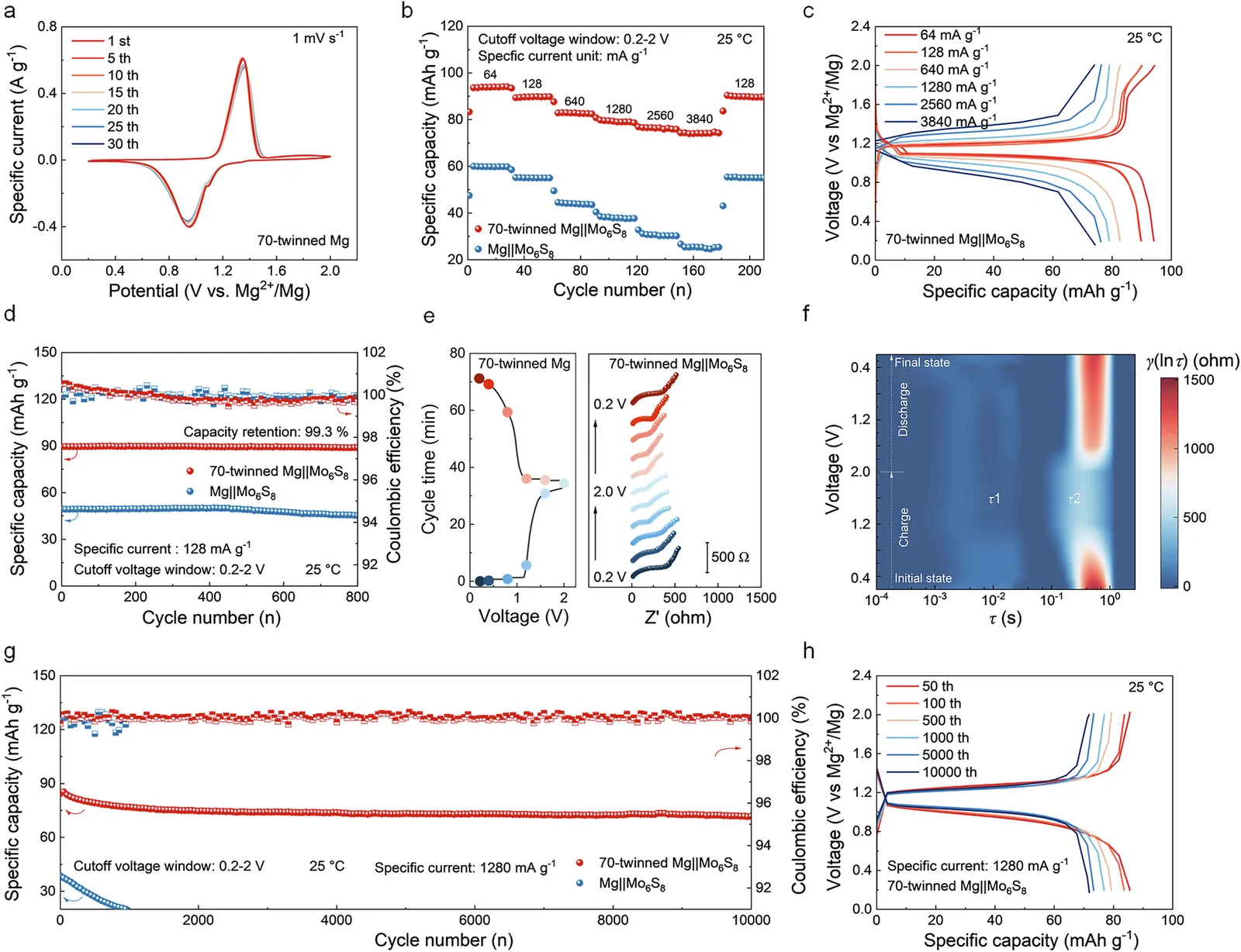
Electrochemical performance of full cells. **a** CV curve of 70-twinned Mg||Mo_6_S_8_ full cells at different cycles. **b** Rate performance of the Mg||Mo_6_S_8_ and 70-twinned Mg||Mo_6_S_8_ full cells at increasing currents from 64 to 3840 mA g^‒1^ (1 C = 128 mA g^‒1^). **c** Charge-discharge curves corresponding to the rate performance of 70-twinned Mg||Mo_6_S_8_ full cells. **d** Cycling stability of the Mg||Mo_6_S_8_ and 70-twinned Mg||Mo_6_S_8_ cells at 128 mA g^‒1^. **e** In situ EIS results of the 70-twinned Mg ||Mo_6_S_8_. **f** Corresponding contour plots of the calculated DRT results from 70-twinned Mg||Mo_6_S_8_. **g** Cycling stability of the Mg||Mo_6_S_8_ and 70-twinned Mg||Mo_6_S_8_ cells at 1280 mA g^‒1^. **h** Charge-discharge curves corresponding to the performance of 70-twinned Mg||Mo_6_S_8_ full cells at 1280 mA g^‒1^. All the tests were measured at 25 °C. Source data are provided as a Source Data file.

To evaluate the practical applicability of the twinned Mg electrode, Mg full cells were assembled on the basis of Mo_6_S_8_ as the positive electrode. The cyclic voltammetry (CV) curve of Mo_6_S_8_ (Fig. 6a) exhibited two distinct reduction peaks at 0.95 and 1.09 V and a corresponding oxidation peak at 1.35 V, indicating the highly reversible redox behaviour of Mo_6_S_8_ during cycling. The overlapping voltage profiles confirmed the reversibility of the Mo_6_S_8_ positive electrode during cycling. Owing to this reversibility, the 70-twinned Mg||Mo_6_S_8_ full cells had reversible capacities of 94, 90, 82, 79, 76, and 74 mAh g^‒1^ at current rates of 64, 128, 640, 1280, 2560, and 3840 C mA g^‒1^ (1 C = 128 mA g^‒1^), respectively (Fig. 6b, c). When the current rate was reduced to 128 mA g^‒1^, the capacity rapidly increased to 90 mAh g^‒1^, indicating a satisfactory rate ability. In contrast, conventional Mg||Mo_6_S_8_ full cells had markedly lower capacity retention at high current densities (Supplementary Fig. S34a), which can be attributed to sluggish Mg^2^⁺ diffusion and the formation of an inhomogeneous interface film caused by uneven Mg deposition^62^. This difference highlights the critical role of the twin-structured Mg negative electrode in facilitating uniform ion transport and stable interfacial behaviour.

Furthermore, long-term cycling tests revealed that the 70-twinned Mg||Mo_6_S_8_ full cells had a capacity of 88.9 mAh g^‒1^ at 128 mA g^‒1^ after 800 cycles, corresponding to a high retention of 99.3% (Fig. 6d and Supplementary Fig. S34b), whereas the Mg||Mo_6_S_8_ cells experienced a significant decrease in capacity and electrochemical performance (Supplementary Fig. S34c). To further investigate the interfacial evolution of different full cells, in situ EIS was conducted to obtain Nyquist plots at various charge–discharge states (Fig. 6e and Supplementary Fig. S35). Compared with Mg||Mo_6_S_8_, the 70-twinned Mg||Mo_6_S_8_ cells exhibited a lower overall impedance during cycling, indicating improved electrochemical kinetics. To analyze the kinetic processes, distribution of relaxation times (DRT) analysis was carried out via DRT tools^63,64^. Specifically, two characteristic relaxation times were identified: τ_1_ (<0.01 s), which is associated with ion transport across the interface, and τ_2_ (0.01–1 s), which is related to charge-transfer reactions. In Mg||Mo_6_S_8_ cells, both τ_1_ and τ_2_ had large peak intensities during cycling (Supplementary Fig. S36), suggesting dynamic changes and increasing barriers for ion and electron transport^65^. In contrast, the 70-twinned Mg||Mo_6_S_8_ cells exhibited significantly weaker and more stable τ_1_ and τ_2_ peaks (Fig. 6f and Supplementary Note 6), revealing decreased interfacial resistance and steady charge-transfer behaviour.

In particular, practical full cells are often required to operate under high current rates, which significantly hinders long-term stability. To examine this aspect, we evaluated the cycling performance of the 70-twinned Mg||Mo_6_S_8_ full cells at 5 C and 10 C (Fig. 6g, h and Supplementary Fig. S37). Notably, the cells maintained 74.3 mAh g^‒1^ after 1500 cycles at 5 C and 71.8 mAh g^‒1^ after 10,000 cycles at 10 C. These capacities not only surpass those of conventional Mg||Mo_6_S_8_ full cells but also outperform comparable full-cell systems (Supplementary Fig. S38 and Table S6). This desirable stability under extreme cycling conditions reveals the improved rate capability of the 70-twinned Mg||Mo_6_S_8_ configuration.

## Discussion

To verify the universality of the twinning-defect-induced highly reversible electrodeposition strategy, we further extended this concept from Mg to Zn metal systems, building upon the successful application of twinned-Mg in significantly enhancing the electrochemical performance of Mg metal batteries. The primary purpose of the Zn study is to serve as a proof-of-concept, aiming to examine whether core strategies derived from the Mg system can be transferred to other metal systems. The choice of Zn as a proof-of-concept system carries important crystallographic and electrochemical implications. From the perspective of crystal defect theory, Zn also adopts an HCP structure and can readily form twin defects during deformation progress. Its typical (10$\bar{1}$2) twins exhibit coherent atomic arrangements and low interfacial energies similar to those found in Mg systems^66^. From the perspective of materials science and electrochemistry, Zn metal batteries have high theoretical capacity (820 mAh g^‒1^), low reduction potential (‒0.76 V vs. SHE), environmental friendliness, and abundant natural reserves^17^. Moreover, they are also plagued by uncontrollable non-uniform growth. Based on these attributes, we also successfully fabricated twinned-Zn electrodes via cyclic multiple-directional deformation processes (Supplementary Fig. S39 and S40). Electrochemical testing revealed that the symmetric cells assembled with the twinned-Zn electrodes achieved stable Zn plating/stripping under high current densities without any observable Zn protrusion formation (Supplementary Fig. S41 and S42), while also exhibiting reduced accumulation of by-products (Supplementary Fig. S43). These results demonstrate that the twin structures play a continuous regulatory and stabilising role during dynamic metal deposition, consistent with the trends observed in the Mg system, thereby providing experimental support for the generality of the proposed strategy. A more in-depth mechanistic elucidation of Zn deposition behaviour will be the focus of future studies.

In summary, we present a distinct deformation-twinning strategy for regulating the interfacial stability and deposition behaviour of Mg negative electrodes. Multiscale characterisation confirms the formation of abundant (10$\bar{1}$2) twins within the fabricated Mg metal. Detailed morphological analyses further reveal that these twins facilitate uniform and compact deposition in halogen-based electrolytes, effectively suppressing uneven growth. Moreover, complementary theoretical calculations reveal that the magnesiumphilic twin structure increases the diffusion rate, resulting in Mg deposition that becomes progressively less diffusion-limited and gradually transitions toward a regime in which reaction kinetics play a more prominent role. In parallel, the reduced reorganisation energy and low overpotential minimise the interfacial impedance and nearly eliminate the passivation layer. These attributes enable highly stable and reversible electrodeposition of twinned Mg negative electrodes. While this work focuses on an in-depth mechanistic investigation of the Mg system, the successful preliminary extension of the strategy to Zn metal provides an important proof-of-concept for its general applicability and opens a promising pathway toward the development of dendrite-free metal batteries.

## Methods

Figure 1a and Supplementary Fig. 21 were created using PowerPoint (PPT, 2021). The experimental data were extracted and processed using Origin 2022 software.

### Materials and electrode preparation

Pure Mg samples were prepared by homogenising ingots (Xinding Metal Materials (Taizhou) Co., Ltd., China; Purity: 99.9 wt.%) at 450 °C (heating rate: 10 °C/min) for 24 hours, followed by water quenching to ambient temperature and subsequent extrusion at 360 °C with an extrusion ratio of 36 and an extrusion speed of 1 mm s^‒1^. Twinned-Mg samples were then obtained by subjecting the pure Mg samples (15 × 15 × 15 mm^3^) to repeated compression cycles along the three principal axes for one, three, and five cycles. Specifically, compression tests were carried out on a Z100HT electronic universal testing machine (Zeck Co. Ltd, Germany) at a strain rate of 10^‒3 ^s^‒1^. A cyclic mode alternating between low strain (3%) and high strain (6.5%) was employed, which enabled the formation of finer twin lamellae. For preparing twinned-Zn sample, the same procedure was adopted but with homogenisation at 320 °C and extrusion at 250 °C, and the repeated compression was applied for three cycles. The compressed samples were subsequently cut into the required dimensions (Diamerter 10 mm, Thickness 0.7 mm) and polished (3000# sandpaper) for further characterisation and electrochemical testing.

Chevrel-phase Mo_6_S_8_ was synthesized via a high-temperature solid-state reaction route. Firstly, MoS_2_, Cu and Mo powders (All raw materials were obtained from Aladdin Co., Ltd.; Purity ≥ 99 %) with a molar ratio of 2:1:1 were ball-milled at 480 rpm for 6 hours under Ar protection. The mixture was transferred to a tube furnace, heated from 25 °C to 1000 °C at a heating rate of 5 °C/min in 5% H_2_/Ar atmosphere, and held at this temperature for 12 hours to obtain the bulk Cu_2_Mo_6_S_8_ precursor. Subsequently, the milled precursor powder was immersed in 6 mol/L HCl solution with continuous stirring for 24 hours. After the reaction, the suspension was repeatedly rinsed with deionized water and centrifuged for purification. Finally, the treated sample was dried in a vacuum oven at 60 °C for 12 hours to acquire the target Mo_6_S_8_ powder.

### Microstructural characterisation

XRD patterns were obtained from polished sample surfaces via a Rigaku D/MAX/2500/HB diffractometer with Cu Kα radiation at 40 kV and 200 mA. Data were collected over a 2θ range of 20–80° with a step size of 0.02° and a scan rate of 4° min^‒1^. The crystal orientation was determined via EBSD. The samples were prepared via mechanical grinding with up to 5000-grit SiC paper, after which they were polished with a water-free diamond suspension (1 μm particle size) and electrochemically polished in an AC2 electrolyte (The electrolyte consists of a mixture of 800 ml ethanol, 100 ml propanol, 18.5 ml distilled water, 10 g hydroxyquinoline, 75 g citric acid, 41.5 g sodium thiocyanate, and 15 ml perchloric acid; all reagents were obtained from Aladdin Co. Ltd., purity ≥ 99%) at 20 V for 60 s at ‒30 °C. EBSD was conducted on a JEOL JSM-7800 microscope equipped with an HKL-EBSD system, and the data were analysed with Channel 5 software. TEM and HRTEM analyses were performed on a JEM-F200 microscope operating at 200 kV. Thin foils were prepared via mechanical polishing from 500 μm to 20–30 μm, punched into 3 mm discs, and further thinned by ion-beam milling on a Gatan 691 system. Surface topography was investigated via field-emission SEM. SEM imaging was conducted at 5 kV with an 8 mm working distance and 5 s acquisition time, whereas elemental mapping was performed at 20 kV with a 2 min acquisition. The morphology of the electrode before and after deposition was observed via confocal laser scanning microscopy. Notably, cycled electrodes were disassembled using a coin cell disassembler (MSK-110D, Shenzhen Kejing Star Technology Company) to prevent bending of the electrode. In addition, the cell for in situ optical microscopy was fabricated using a mould supplied by Tianjin Goshi Ruilian Photoelectric Technology Co., Ltd. Mg was employed for both the working and counter electrodes, with the APC electrolyte (0.4 M PhMgCl in tetrahydrofuran (THF) with 0.25 M AlCl_3_; Suzhou DoDoChem Co., Ltd., China) serving as the medium. An electrochemical workstation provided the power source and applied a current density of 10 mA cm^‒2^. CT scanning technology was employed to assess the compactness of the sedimentary layer. Specifically, an Xradia 515 Versa 3D X-ray microscope was used to perform high-resolution, high-contrast scanning imaging of localised sample regions at a voxel resolution of 0.3 µm. Dragonfly software was used to divide and extract internal pores within the sedimentary layer, calculate porosity, and ultimately achieve three-dimensional structural reconstruction of both pores and sediments. Data analysis was conducted on a volume of 300 × 250 × 20 µm. XPS was conducted on a Thermo Scientific Escalab 250Xi instrument equipped with an Al Kα source. The spectra were processed using Avantage software, in which all data were calibrated with respect to the signal of adventitious carbon at 284.8 eV. The samples were prepared in a glovebox and immediately transferred to an argon-sealed container. Time-of-flight secondary ion mass spectrometry was performed with a Bi_3_⁺⁺ primary ion beam at 30 kV over a 100×100 μm^2^ area, with sputtering achieved with a 2 kV Ar⁺ beam at a rate of 0.11 nm s^‒1^. Additionally, during the electrode testing process, the samples were transported using an Ar gas-filled transfer box to ensure they remained uncontaminated and stable during the transfer.

### Cell assembly and electrochemical measurements

All CR2032-type coin cells with 316 stainless steel spring (Diameter 15.4 mm × Thickness 1.1 mm, MTI) were assembled in an argon-filled glovebox, with oxygen and H_2_O concentrations below 0.1 ppm. Moreover, metal electrodes were prepared immediately before assembling the battery. In addition, all electrochemical cycling tests were conducted in a constant temperature box at 25 °C. The conductive additives included Super P carbon black (Super P, purity: 99%) and a PVDF binder (PVDF, purity: 99%, Mw = 1000000) (Shenzhen Kejing Star Technology Company). Copper foil (purity 99.8%, 12 μm) and nickel foil (purity 99%, 12 μm) served as current collectors (Shenzhen Kejing Star Technology Company). Electrochemical measurements were performed on a multichannel cell tester (CT2001A, LAND). Symmetric Mg||Mg cells were fabricated on the basis of Mg electrodes as both the positive electrode and negative electrode, separated by a GF/D separator with 47 mm diameter, 670 μm thickness, and 2.7 μm pore size (GFD Wattman, UK) soaked in APC electrolyte, to evaluate the deposition and stripping behaviour. Additionally, symmetric Zn||Zn cells were constructed using Zn electrodes and a 2 M ZnSO_4_ aqueous solution as the electrolyte (ZnSO_4_ purity 99.7%, Aladdin). With respect to the Cu||Mg cells, Cu foil was used as the positive electrode, and Mg was used as the negative electrode, with a cut-off voltage of 1.0 V, to examine the Mg plating/stripping stability. Tafel plots, LSV, CV, EIS, and CA were constructed on a Biologic VMP3 electrochemical workstation. Notably, all CA experiments discussed in this paper were conducted as symmetric cell tests under a strictly defined three-electrode configuration, using Swagelak-type cells. In addition, the working electrode was either pure Mg foil or twinned-Mg foil, while both the counter and quasi-reference electrodes were pure Mg foils; Unless otherwise specified, all electrodes were polished with 3000# sandpaper and cut into 10 mm diameter discs. A glass fibre separator was used, and 200 μL of APC electrolyte was added to ensure sufficient wetting. This configuration ensures an unambiguous definition of the working-electrode potential and eliminates polarization contributions from the counter electrode, thereby satisfying the fundamental requirements for kinetic analysis based on nucleation models. For the full-cell tests, Mo_6_S_8_ was employed as the positive electrode material. The Mo_6_S_8_ slurry was prepared by mixing Mo_6_S_8_ powder, Super P, and a PVDF binder at a weight ratio of 8:1:1, followed by coating on nickel foil. The electrodes were dried under vacuum at 60 °C overnight. The loading of the active materials was 1.5 mg cm^‒2^. Galvanostatic cycling was conducted between 0.2 and 2.0 V at various current densities. In situ EIS was performed on an electrochemical workstation over a frequency range of 100 kHz to 1 Hz. The EIS test was set with 6 points per decade of frequency with an amplitude of 10 mV, using a potentiostatic signal. Prior to the EIS measurements, the sample was held at a quasi-steady-state potential for 5 min to ensure stability.

### DFT calculations

Spin-polarized density functional theory (DFT) calculations were performed using the Vienna Ab initio Simulation Package (VASP) with the projector augmented wave (PAW) approach^67^. A plane-wave cutoff energy of 400 eV was adopted. Electron–ion interactions were treated with PAW pseudopotentials, while exchange–correlation effects were described by the Perdew–Burke–Ernzerhof (PBE) functional within the generalized gradient approximation (GGA)^68^. Long-range van der Waals interactions were considered using the DFT-D3(BJ) dispersion correction. Surface reaction mechanisms were modelled by constructing slab geometries with a vacuum layer of 15 Å to suppress spurious periodic interactions. The Brillouin zone was sampled with a Monkhorst–Pack k-point grid at a spacing of 0.04 Å^‒1^. Electronic self-consistent field iterations converged to 10^‒5 ^eV, and atomic relaxations were continued until the residual forces were below 0.03 eV Å^‒1^. Transition states were located via the climbing-image nudged elastic band (CI-NEB) method^69^. The Gibbs free energy (G) for each species at temperature T was subsequently obtained by incorporating the vibrational contributions into the electronic energy: G = accurate total electronic energy+zero-point energy-T*vibrational entropy. For the surface reaction where tetrahydrofuran (THF) decomposes on the Mg (pure Mg/twinnedMg) substrate (Mg_sub_) to form MgO species and C_4_H_8_, the reaction free energy ΔG was calculated using the formula: ΔG = G[(MgO /Mgsub)*]−G(C_4_H_8_O)* −G(Mgsub) + G(C_4_H_8_)+2eφ. Here, G[(MgOads/Mgsub)*] is the free energy of the Mg substrate with MgO species, G(C_4_H_8_O)* is the free energy of the adsorbed tetrahydrofuran reactant, G(Mgsub) is the free energy of the clean substrate, G(C_4_H_8_) is the free energy of the butene product, e is the elementary charge, and φ the electrode potential. For the surface reaction where [MgCl·5THF]^+^ decomposes on the Mg (pure Mg/twinnedMg) substrate (Mgsub) to form MgCl species and other species. The reaction free energy ΔG was calculated using the formula: ΔG = G[(MgCl_2_/Mgsub)*]+10 G(C_4_H_8_O) + G[(Mg/Mgsub)*]-2G([MgCl·5THF]^+^)-G(Mgsub) + 2eφ. Here, G[(MgCl_2_/Mgsub)*] is the free energy of the Mg substrate with MgCl_2_ species, and G[(Mg/Mgsub)*] is the free energy of the Mg substrate with Mg species.

### Finite element simulations

Finite-element modelling was performed using COMSOL Multiphysics 6.1 to elucidate the dynamic behaviour of Mg electrodeposition. Within the tertiary current distribution framework, ionic transport in the electrolyte—encompassing both diffusion and migration—was governed by the Nernst–Planck formalism:

3 $${J}_{{{{{\rm{M}}}}g}^{2+}}=-{D}_{{{{{\rm{M}}}}g}^{2+}}({\nabla }_{{c}_{0}}+\frac{{zF}{c}_{0}}{{RT}}\nabla \varphi )$$

where *J*_Mg_^2+^ denotes the Mg^2+^ flux, *D*_Mg_^2+^ is the diffusion coefficient, *c*_0_ is the Mg^2+^ concentration, *z* is the transferred electron numbers, *F* is the Faraday’s constant, *R* is the gas constant, *T* is the absolute temperature and *φ* corresponds the electrolyte potential.

The dependence of local current density on overpotential and Mg^2+^ concentration was described using Butler-Volmer kinetics:

4 $$i={i}_{0}\left[exp \left(\frac{{\alpha }_{a}F\eta }{{RT}}\right)-\frac{{c}_{{{{{\rm{M}}}}g}^{2+}}}{{c}_{0}}exp \left(-\frac{{\alpha }_{c}F\eta }{{RT}}\right)\right]$$

where *i*_0_ is the exchange current density, *α* is the charge transfer coefficient, *η* is the overpotential and *c*_Mg_^2+^ represents the interfacial Mg^2+^ concentration.

## Supplementary information

Supplementary Information

Transparent Peer Review file

## Source data

Source Data

## Data Availability

The datasets generated during and/or analyzed during the current study are available from the corresponding author on reasonable request. The DFT data are available at https://doi.org/10.24435/materialscloud:vz-av. Source data are provided with this paper.
