# Author Correction: Twinned-magnesium metal negative electrodes enable reversible magnesium electrodeposition

**DOI:** 10.1038/s41467-026-78751-y

**Published:** 2026-10-19

**Authors:** Guodong Zou, Jingli Ren, Jinming Wang, Yong Sun, Weihao Yang, Tingting Niu, Jinyu Li, Carlos Fernandez, Jean-Jacques Gaumet, Qiuming Peng

**Affiliations:** 1 State Key Laboratory of Metastable Materials Science and Technology, Yanshan University, Qinhuangdao, China; 2 School of Pharmacy and Life Sciences, Robert Gordon University, Aberdeen, UK; 3 Laboratoire de Chimie et Physique, Approche Multi-échelles des Milieux Complexes, Institute Jean Barriol, Université de Lorraine, Metz, France

**Keywords:** Batteries, Batteries, Batteries

Correction to: *Nature Communications* https://doi.org/10.1038/s41467-026-77543-8, published online 4 September 2026

In the version of this article initially published, there were typographical errors, where in the Fig. 2d legend, “LCSM” originally appeared as “CLSM” and in the Fig. 5b caption, “MgCl_2_, MgO, and Mg^0^” originally appeared as “MgCl_2_, MgO, and MgO.” The text is amended in the HTML and PDF versions of the article.

