# Supplementary material for: Twinned-magnesium metal negative electrodes enable reversible magnesium electrodeposition
Source: Nat Commun. 2026 Sep 4;17:9599. doi: 10.1038/s41467-026-77543-8 (PMC13554073; doi:10.1038/s41467-026-77543-8)
Supplement: Supplementary file 1 [file 41467_2026_77543_MOESM1_ESM.pdf]

Supporting Information

Twinned-magnesium metal negative electrodes enable reversible magnesium electrodeposition

Guodong Zou[1], Jingli Ren[1], Jinming Wang[1,*], Yong Sun[1], Weihao Yang[1], Tingting Niu[1], Jinyu Li[1], Carlos Fernandez[2], Jean-Jacques Gaumet[3], Qiuming Peng[1,*]

[1]*State Key Laboratory of Metastable Materials Science and Technology, Yanshan University, Qinhuangdao, 066004, P.R. China*

[2]*School of Pharmacy and Life Sciences, Robert Gordon University, Aberdeen, AB107GJ, United Kingdom*

[3]*Laboratoire de Chimie et Physique, Approche Multi-échelles des Milieux Complexes, Institute Jean Barriol, Université de Lorraine, Metz 57070, France*

Supporting Figures 1-43

Supporting Note 1-6

Supporting Tables 1-6

Corresponding Authors: wjm@ysu.edu.cn; pengqiuming@ysu.edu.cn

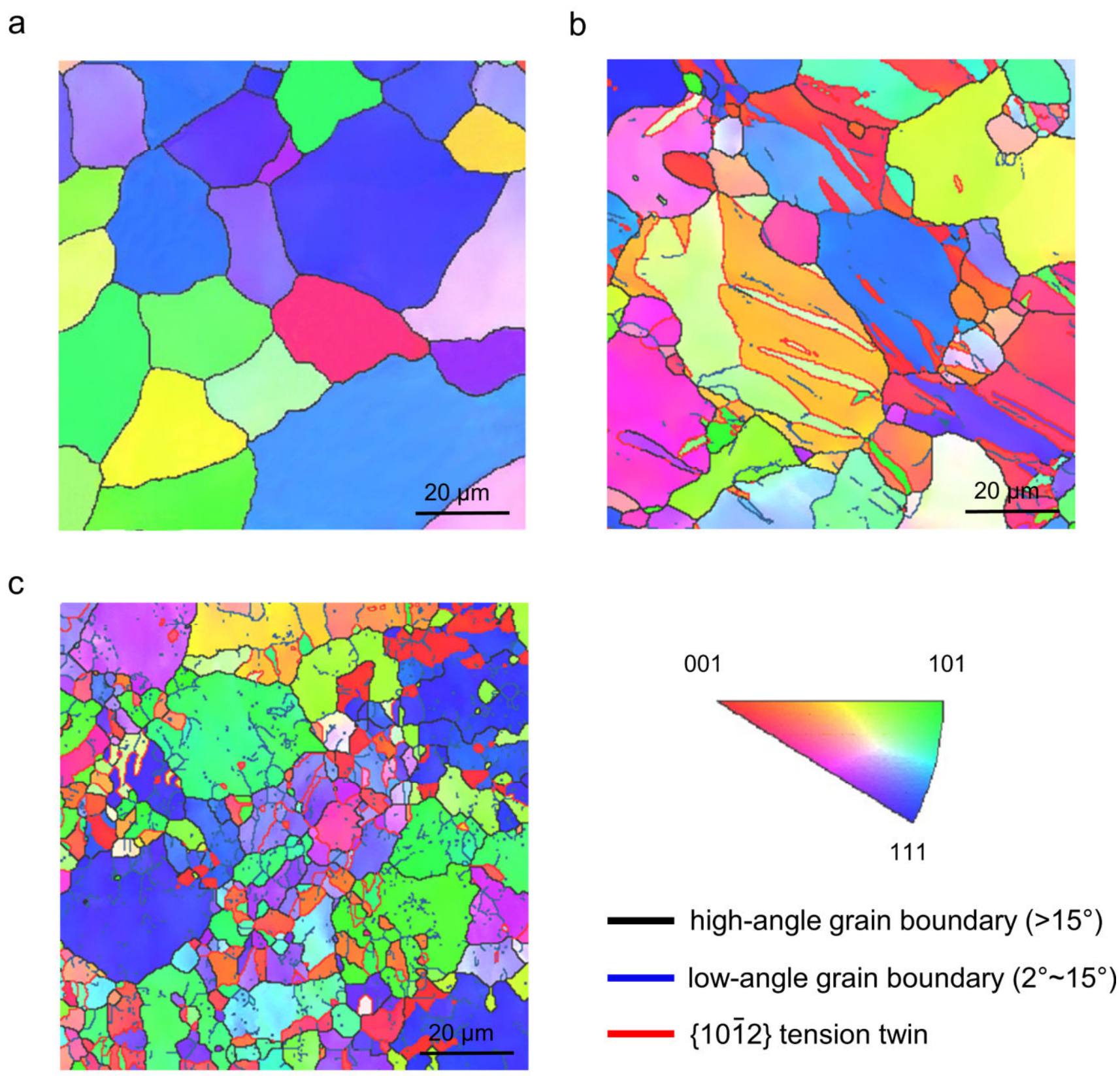

Supplementary Fig. 1. a-c Orientation maps of pure Mg (a), 50-twinned Mg (b), and 40-twinned Mg (c).

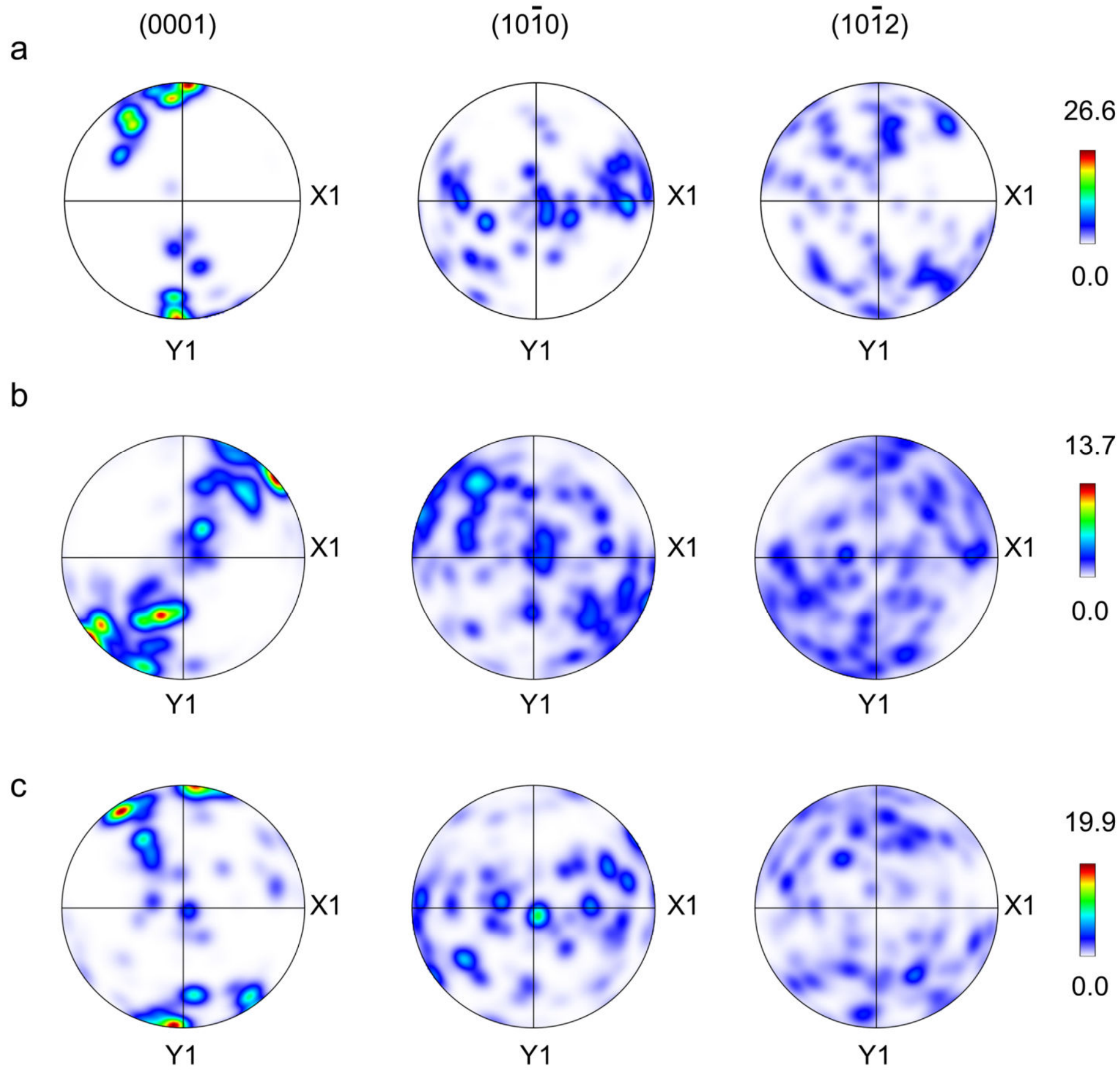

Supplementary Fig. 2. a-c (0001) (10$\bar{1}$0) and (10$\bar{1}$2) pole figures of the pure Mg (a), 50-twinned Mg (b),and 40-twinned Mg (c).

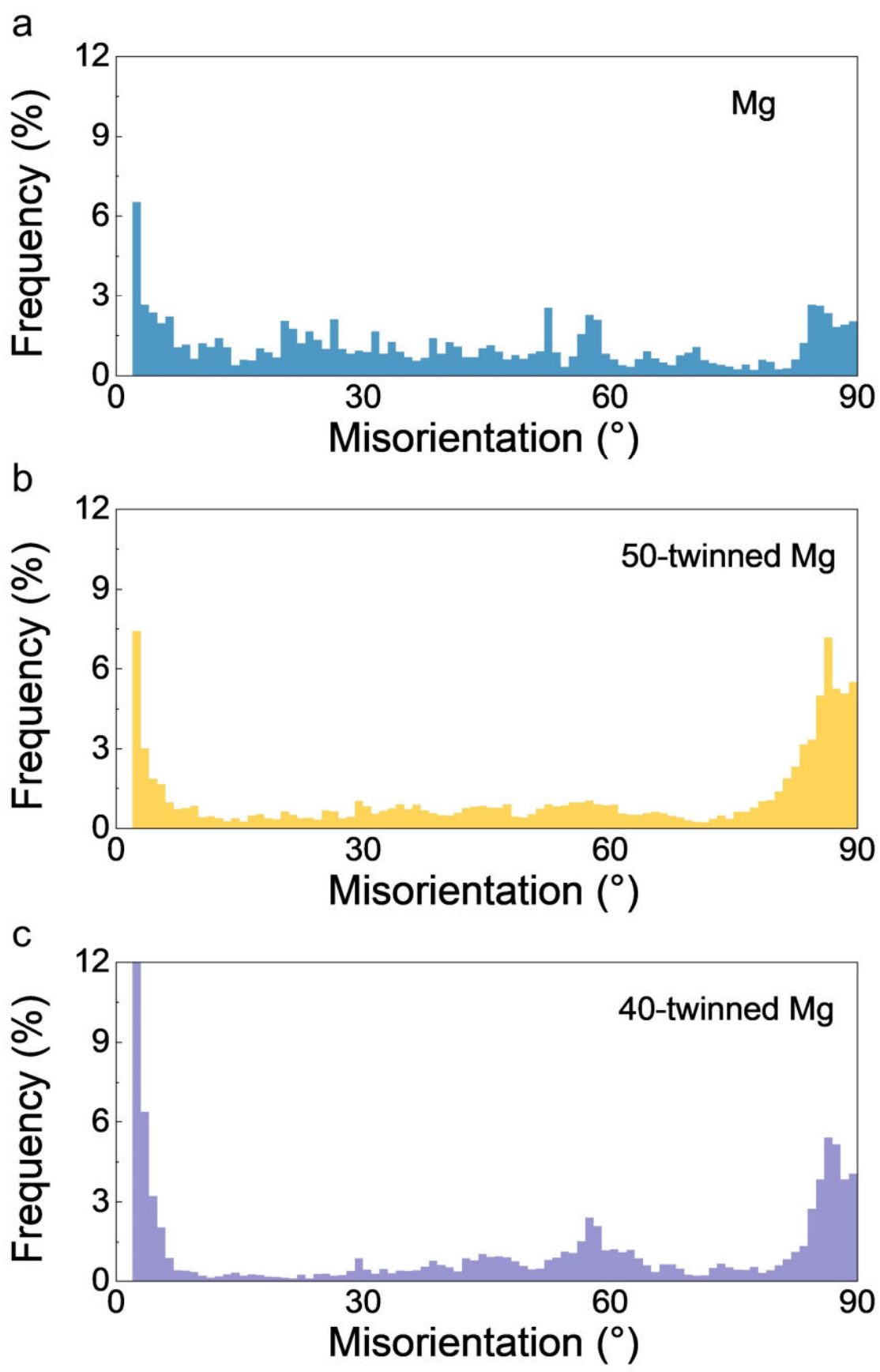

Supplementary Fig. 3. a-c Statistical analyses of the misorientation on the Mg (a), 50-twinned Mg (b),and 40-twinned Mg (c). Source data are provided as a Source Data file.

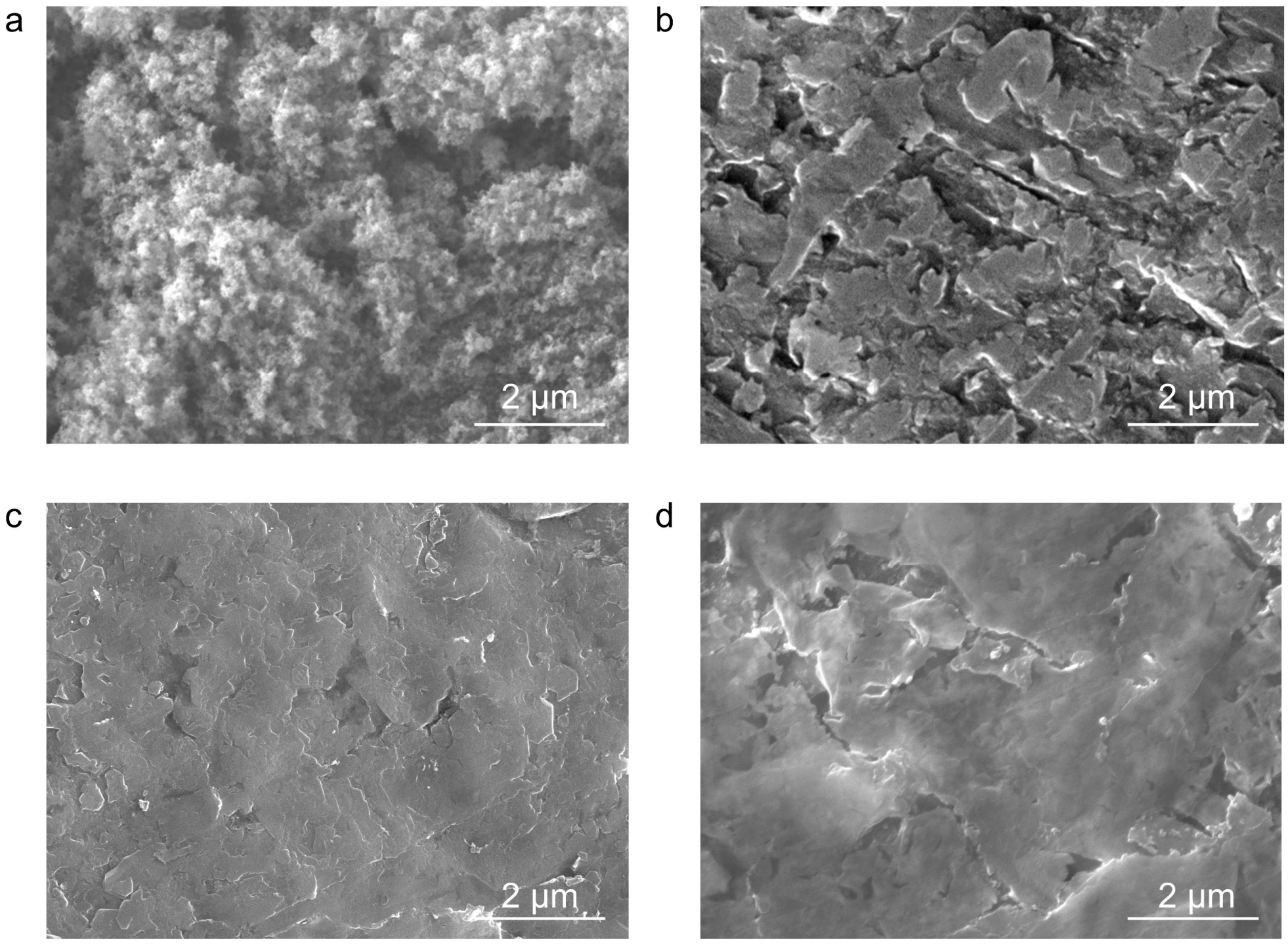

Supplementary Fig. 4. a-d Mg deposition morphology on Mg (a), 50-twinned Mg (b), 70-twinned Mg (c) and 40-twinned Mg (d) surfaces under 10 mA cm^{-2}, with 5 mAh cm^{-2}. All the tests were measured at 25 °C.

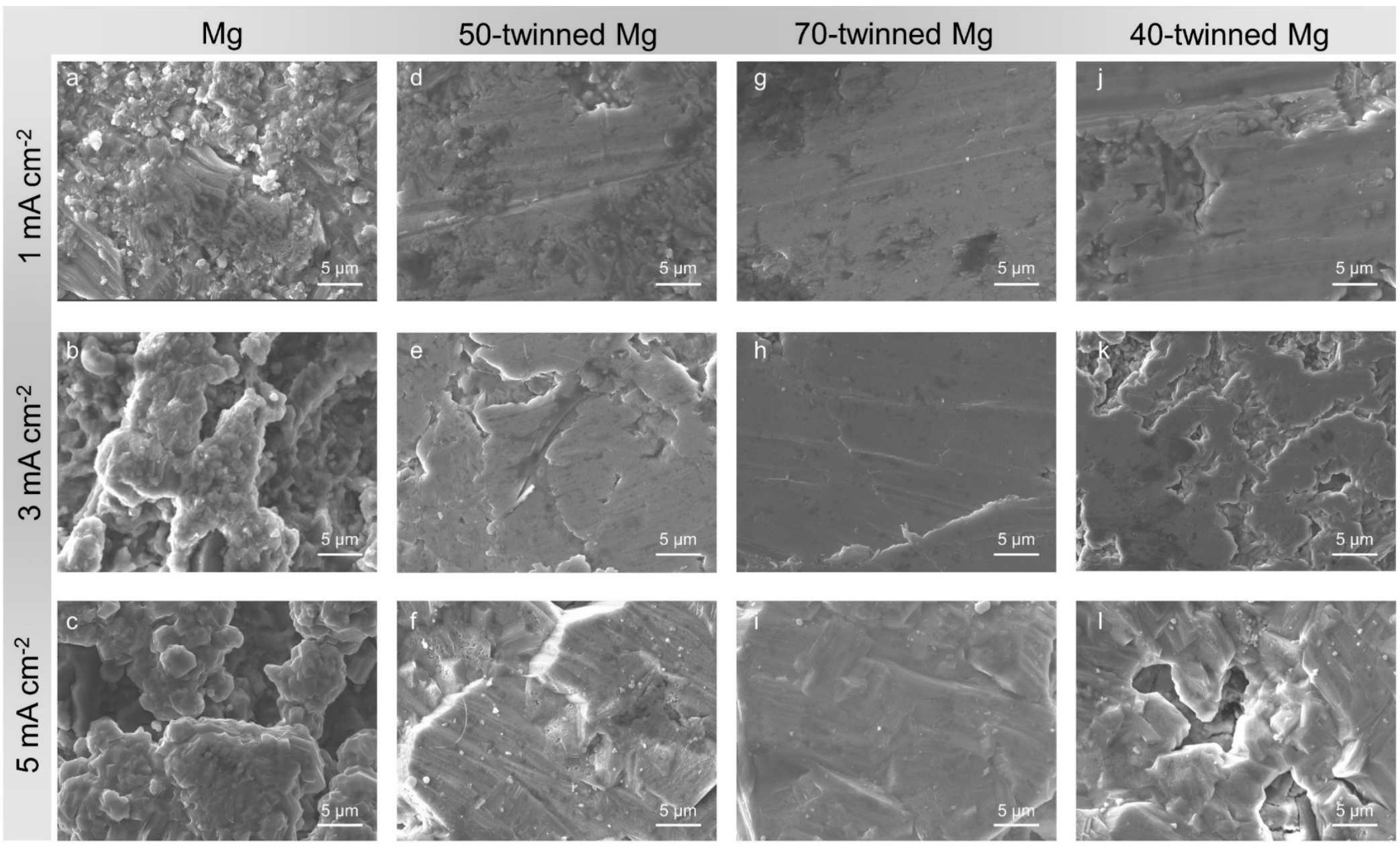

Supplementary Fig. 5. a-c Mg deposited on bare Mg at 1 mA cm^{-2} (a), 3 mA cm^{-2} (b), 5 mA cm^{-2} (c). **d-f** Mg deposited on 50-twinned Mg at 1 mA cm^{-2} (d), 3 mA cm^{-2} (e), 5 mA cm^{-2} (f). **g-i** Mg deposited on 70-twinned Mg at 1 mA cm^{-2} (g), 3 mA cm^{-2} (h), 5 mA cm^{-2} (i). **j-l** Mg deposited on 40-twinned Mg at 1 mA cm^{-2} (j), 3 mA cm^{-2} (k), 5 mA cm^{-2} (l). The deposition capacities are 1 mAh cm^{-2}. All the tests were measured at 25 °C.

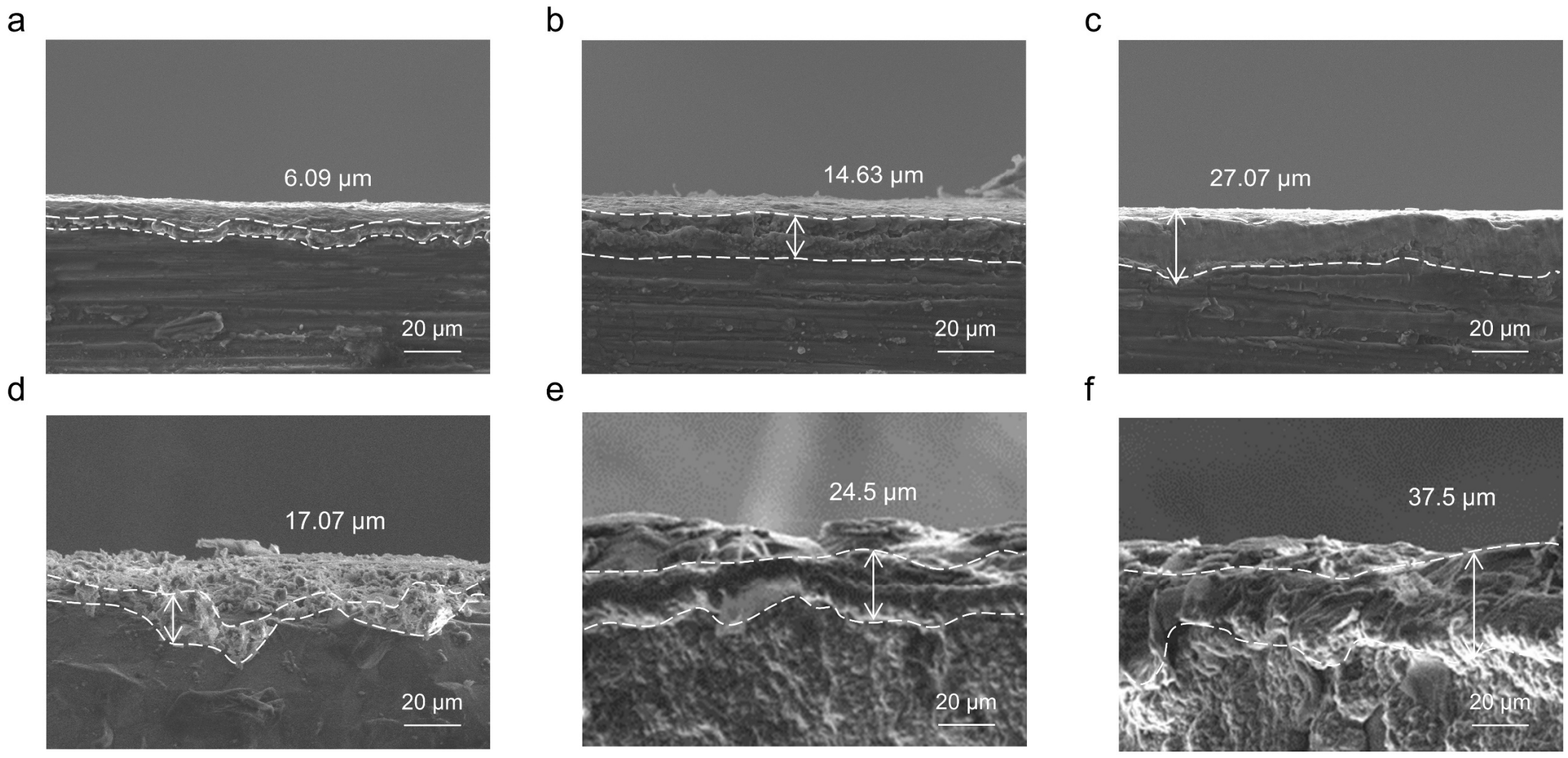

Supplementary Fig. 6. a-c Cross-section SEM images of Mg deposited on 70-twinned Mg under 10 mA cm^{-2}, with 2 mAh cm^{-2} (a), 5 mAh cm^{-2} (b), and 10 mAh cm^{-2} (c). **d-f** Cross-section SEM images of Mg deposited on pure Mg under 10 mA cm^{-2}, with 2 mAh cm^{-2} (d), 5 mAh cm^{-2} (e), and 10 mAh cm^{-2} (f). All the tests were measured at 25 °C.

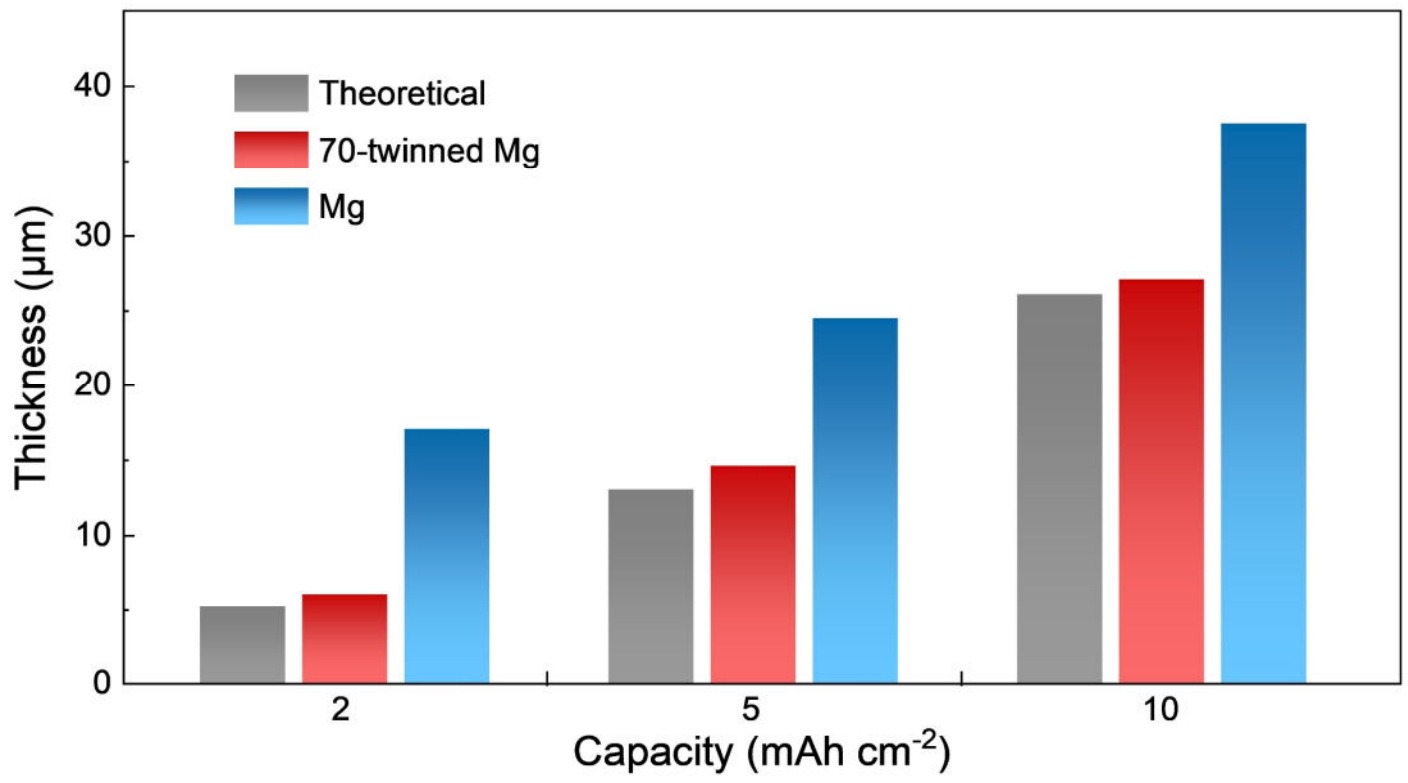

Supplementary Fig. 7. Summarized average thickness data of Mg deposited on Mg and 70-twinned Mg under 2 mAh cm^{-2}, 5 mAh cm^{-2},and 10 mAh cm^{-2}. The current density is 10 mA cm^{-2}. Source data are provided as a Source Data file.

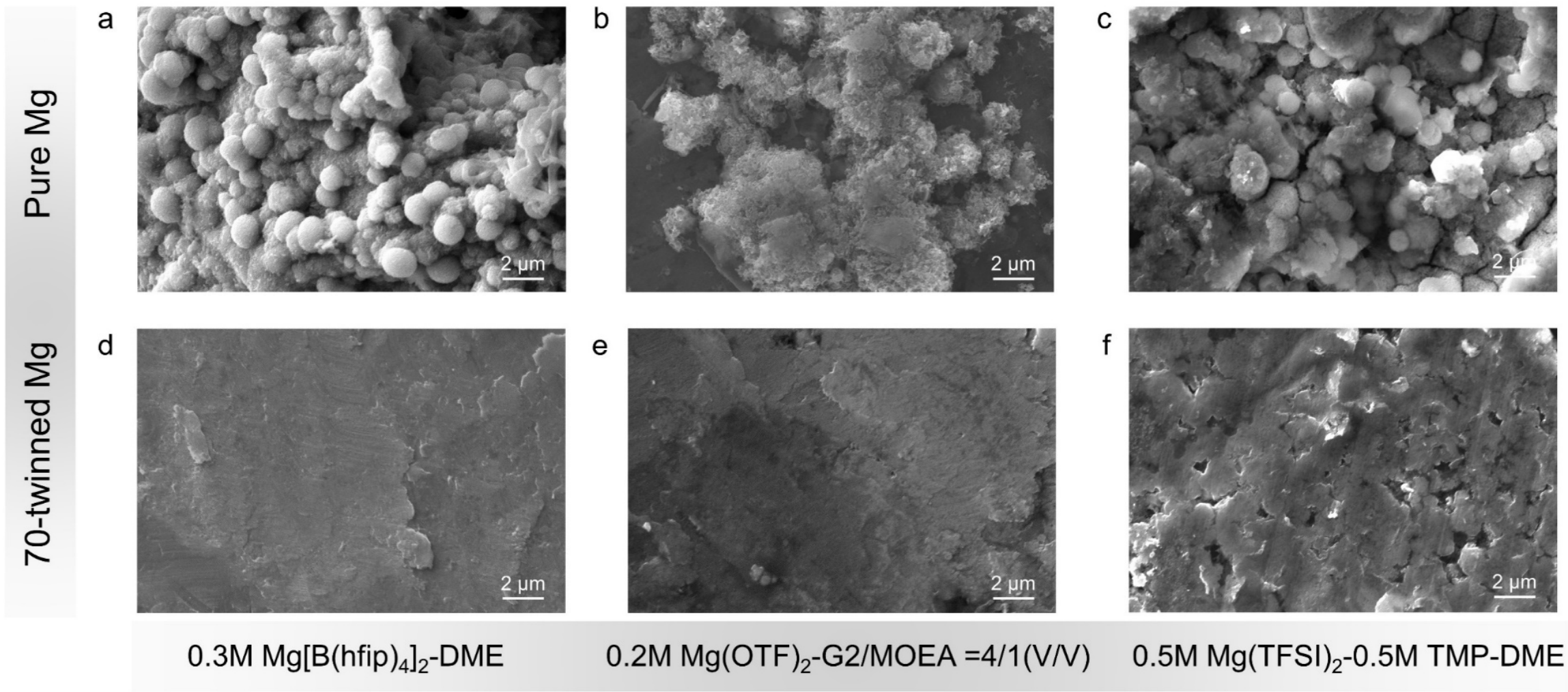

Supplementary Fig. 8. a-f Mg deposition morphology on pure Mg (a-c) and 70-twinned Mg (d-f) surfaces under 10 mA cm^{-2}, with 5 mAh cm^{-2} in different electrolyte. All the tests were measured at 25 °C. ($Mg[B(hfip)_4]_2$: Magnesium tetrakis(hexafluoroisopropyloxy)borate; DME: 1-2 dimethoxyethane; $Mg(OTF)_2$: Magnesium trifluoromethanesulphonate; G2: Diglyme; MOEA: 2-Methoxyethylamine; $Mg(TFSI)_2$: Magnesium bis(trifluoromethylsulfonyl)imide; TMP: Trimethyl phosphate)

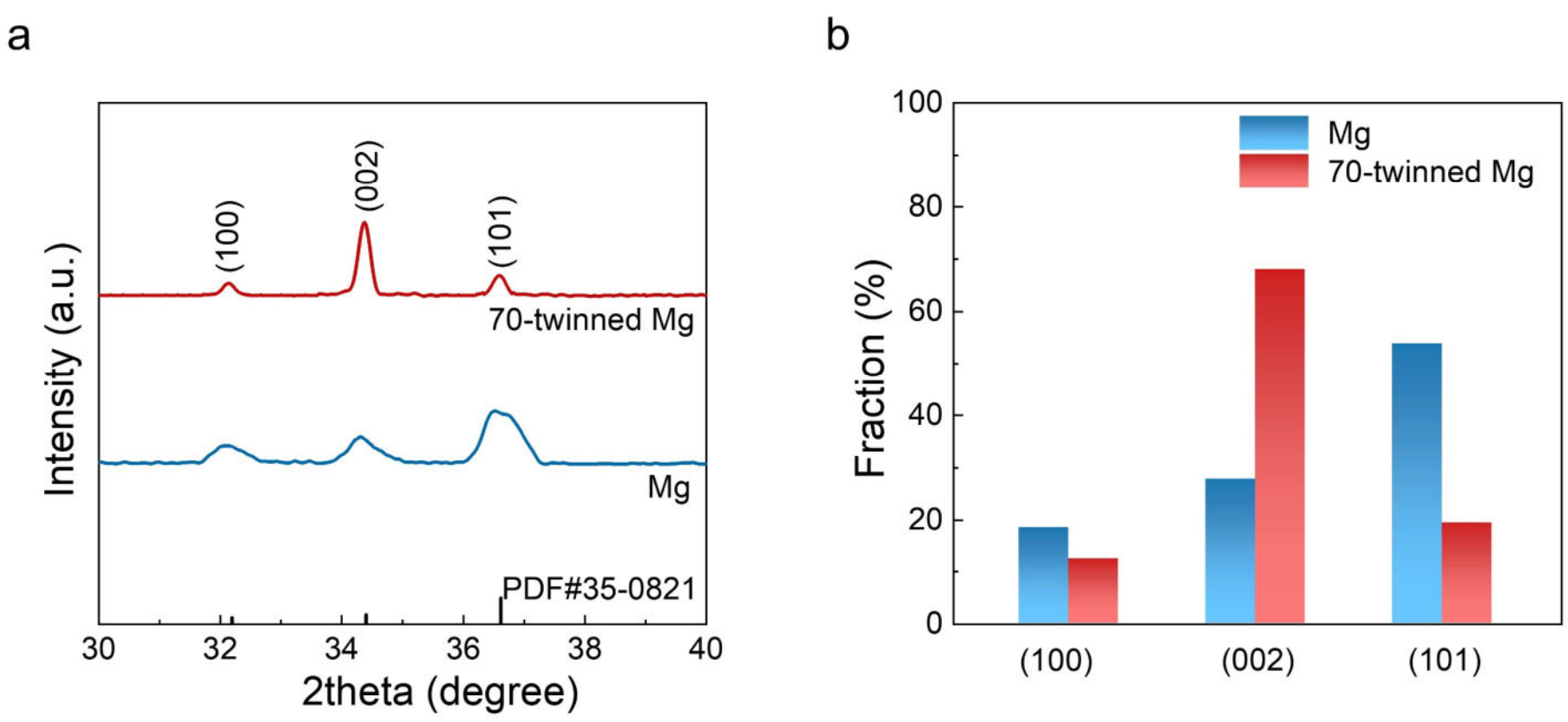

Supplementary Fig. 9. a XRD patterns of Mg and 70-twinned Mg electrodes after plating. **b** The corresponding deposited crystal plane fraction. Source data are provided as a Source Data file.

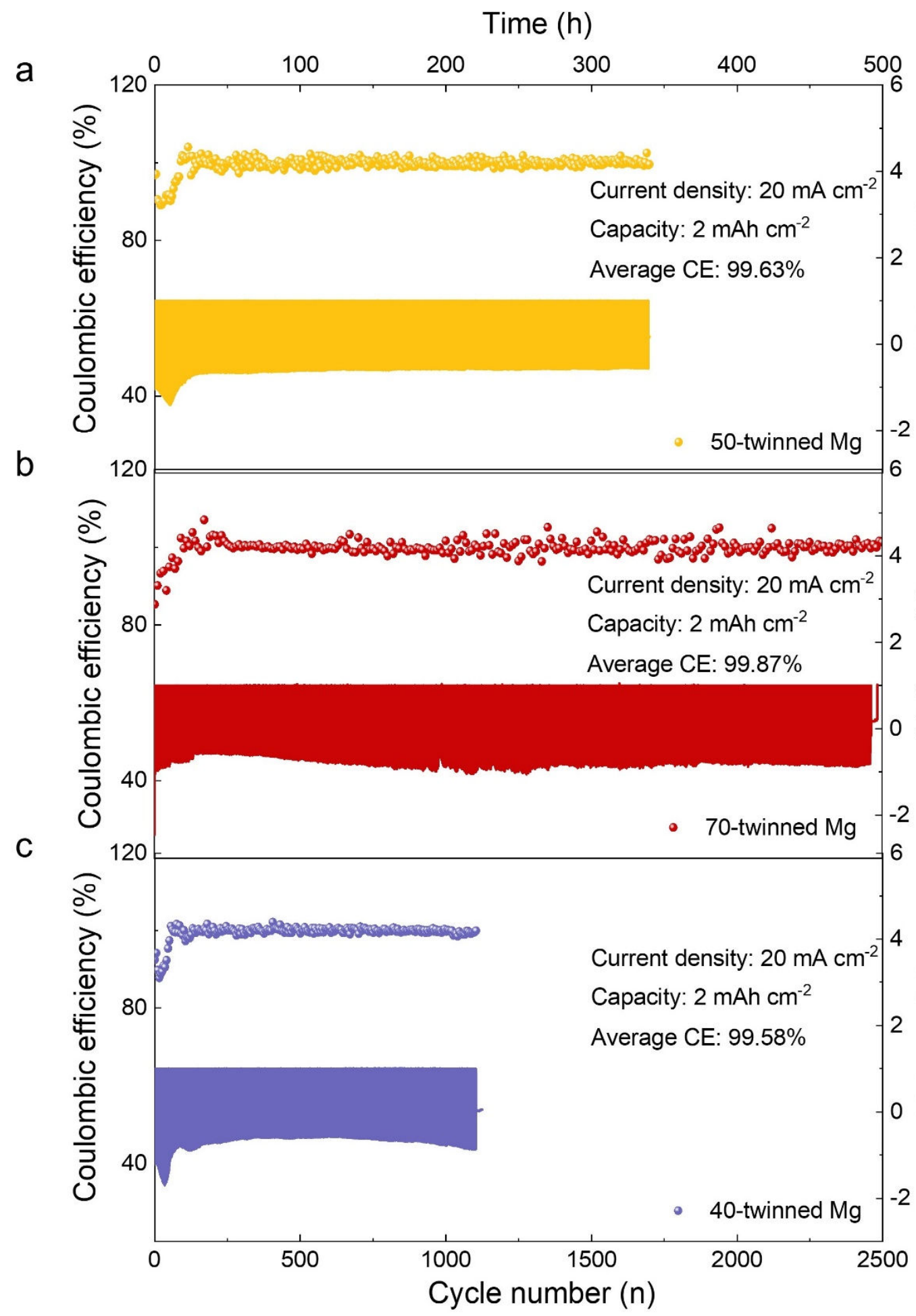

Supplementary Fig. 10. a-c Mg plating/stripping performances in 50-twinned Mg||Cu (a) 70-twinned Mg ||Cu (b) and 40-twinned Mg||Cu (c) cells at 20 mA cm^{-2}/2 mAh cm^{-2}. All the tests were measured at 25 °C. Source data are provided as a Source Data file.

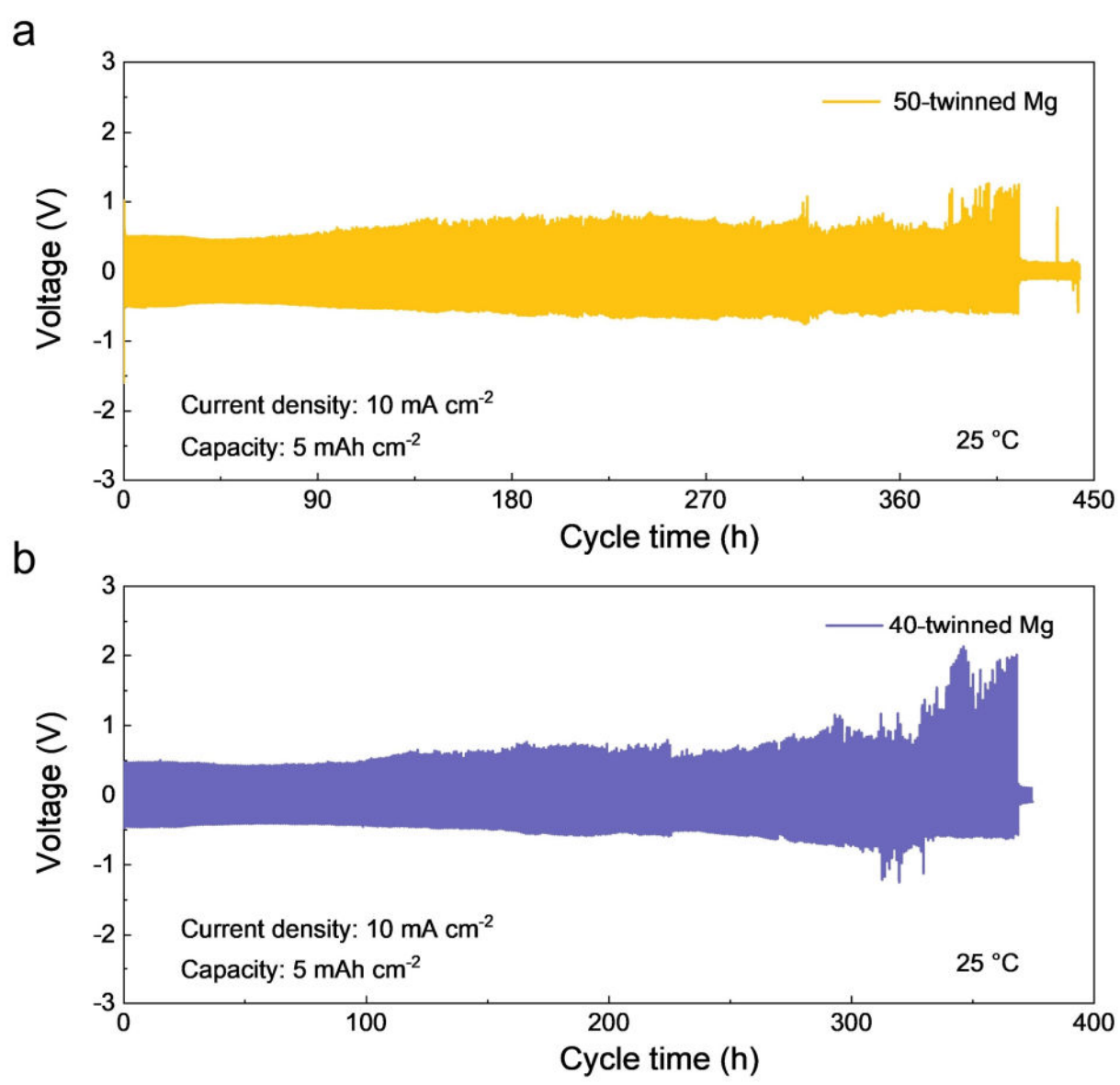

Supplementary Fig. 11. a, b Cyclic stability of the 50-twinned Mg||50-twinned Mg (a) and 40-twinned Mg||40-twinned Mg (b) symmetric cells at 10 mA cm^{-2}/5 mAh cm^{-2}. All the tests were measured at 25 °C. Source data are provided as a Source Data file.

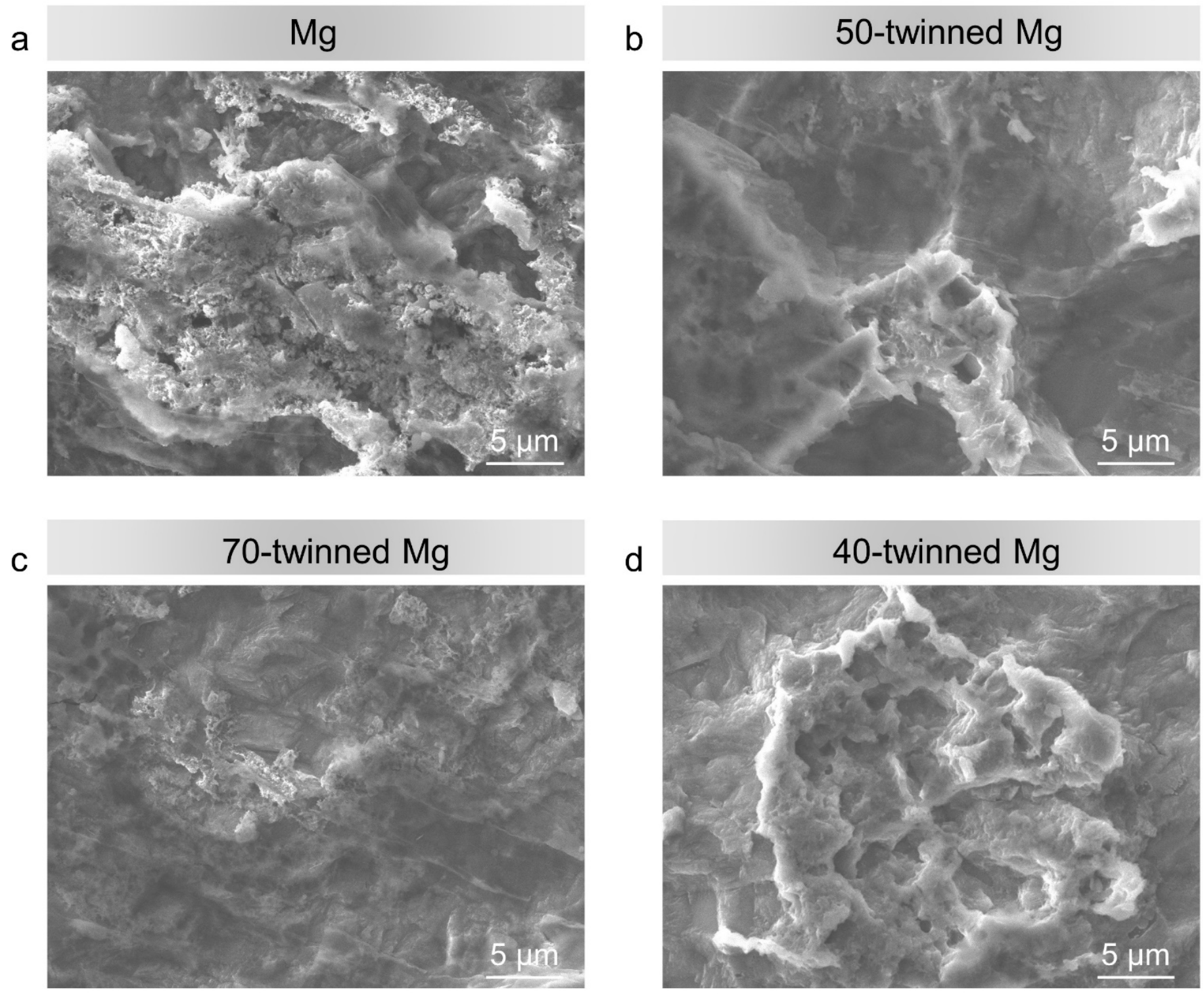

Supplementary Fig. 12. a-d SEM images of Mg (a), 50-twinned Mg (b), 70-twinned Mg (c), and 40-twinned Mg (d) electrodes after Mg plating/stripping at a current density of 10 mA cm^{-2} and a capacity of 5 mAh cm^{-2} for 10 cycles. All the tests were measured at 25 °C.

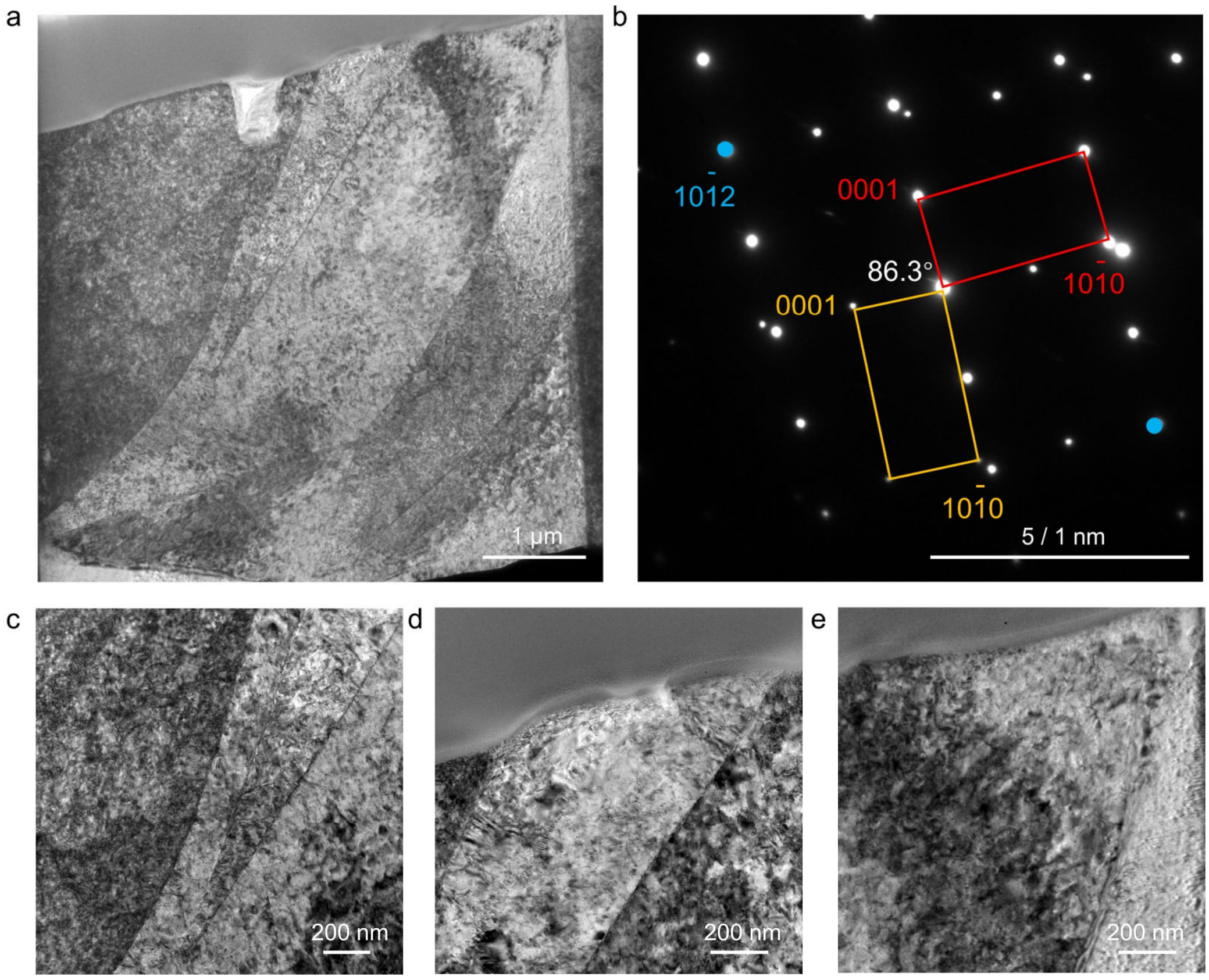

Supplementary Fig. 13. a-e TEM image (a) and corresponding SAED (b) of 70-twinned Mg after Mg plating/stripping (50 cycles, 10 mA cm^{-2}, 5 mAh cm^{-2}), as well as magnified detail images (c-e).

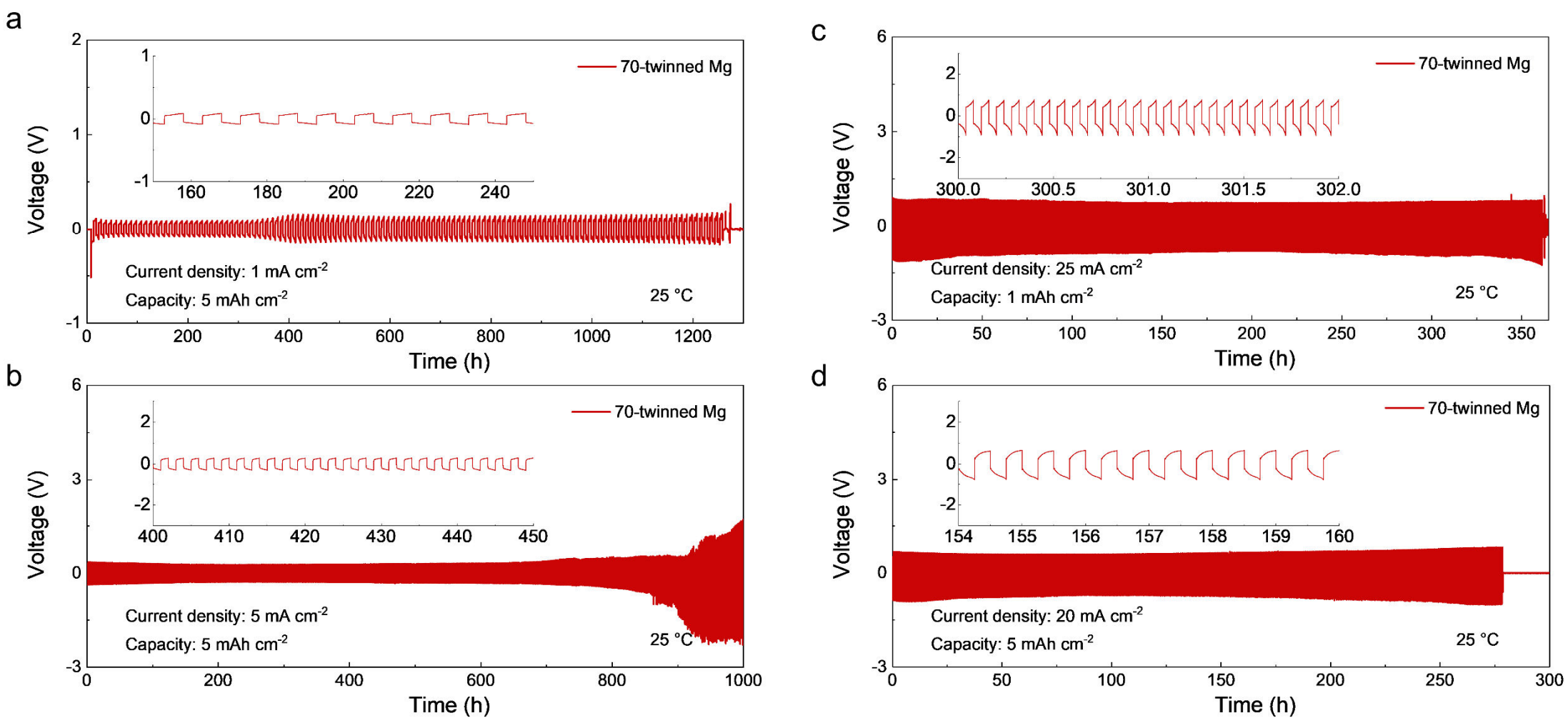

Supplementary Fig. 14. a-d Cyclic stability of different symmetric cells at 1 mA cm^{-2}/5 mAh cm^{-2} (a), 5 mA cm^{-2}/5 mAh cm^{-2}, (b) 25 mA cm^{-2}/1 mAh cm^{-2} (c), 20 mA cm^{-2}/5 mAh cm^{-2} (d). All the tests were measured at 25 °C. Source data are provided as a Source Data file.

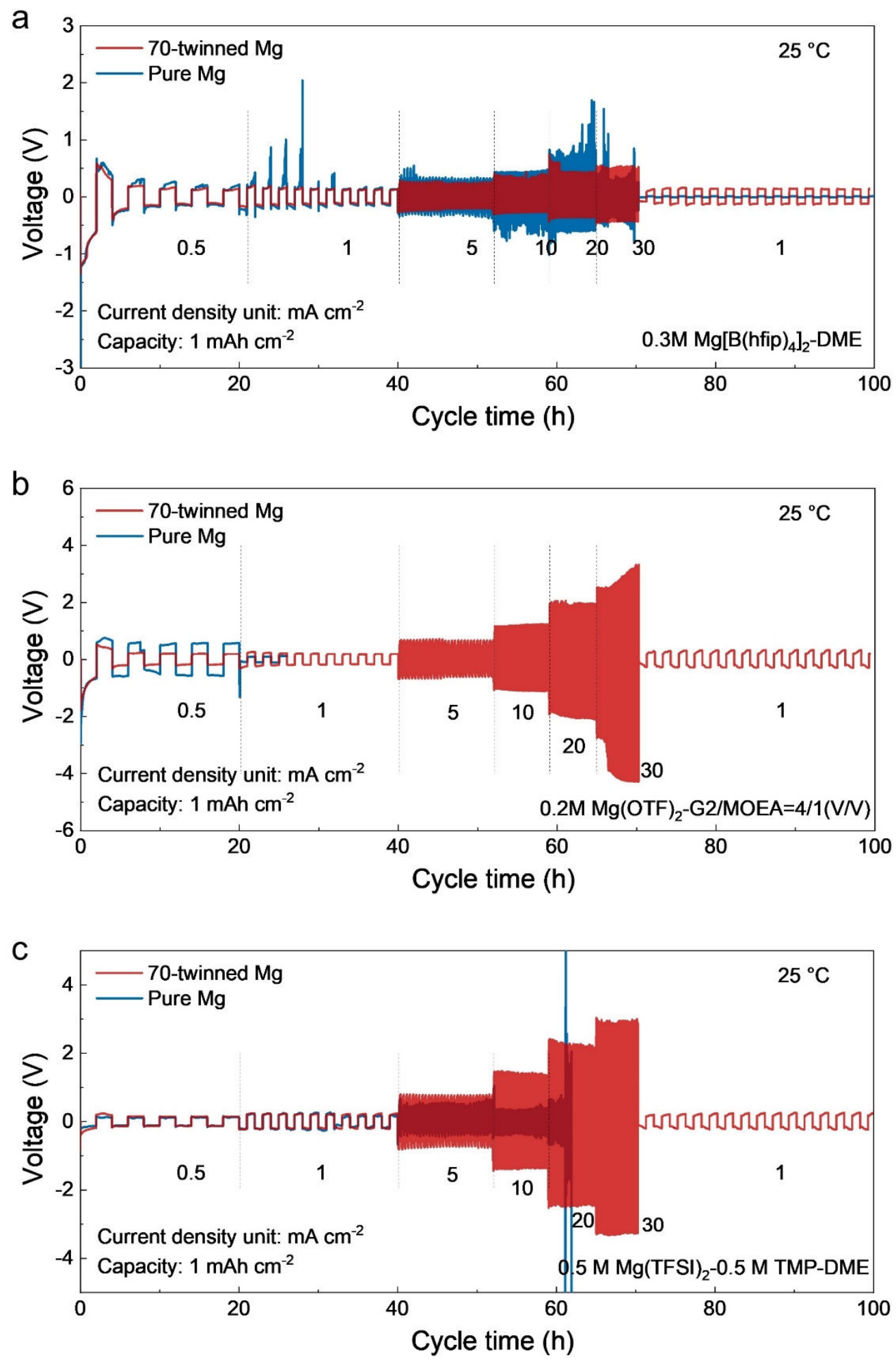

Supplementary Fig. 15. a-c Rate performance of the pure Mg and 70-twinned Mg symmetric cells in different electrolyte at various current densities with the same capacity of 1 mAh cm^{-2}. All the tests were measured at 25 °C. Source data are provided as a Source Data file.

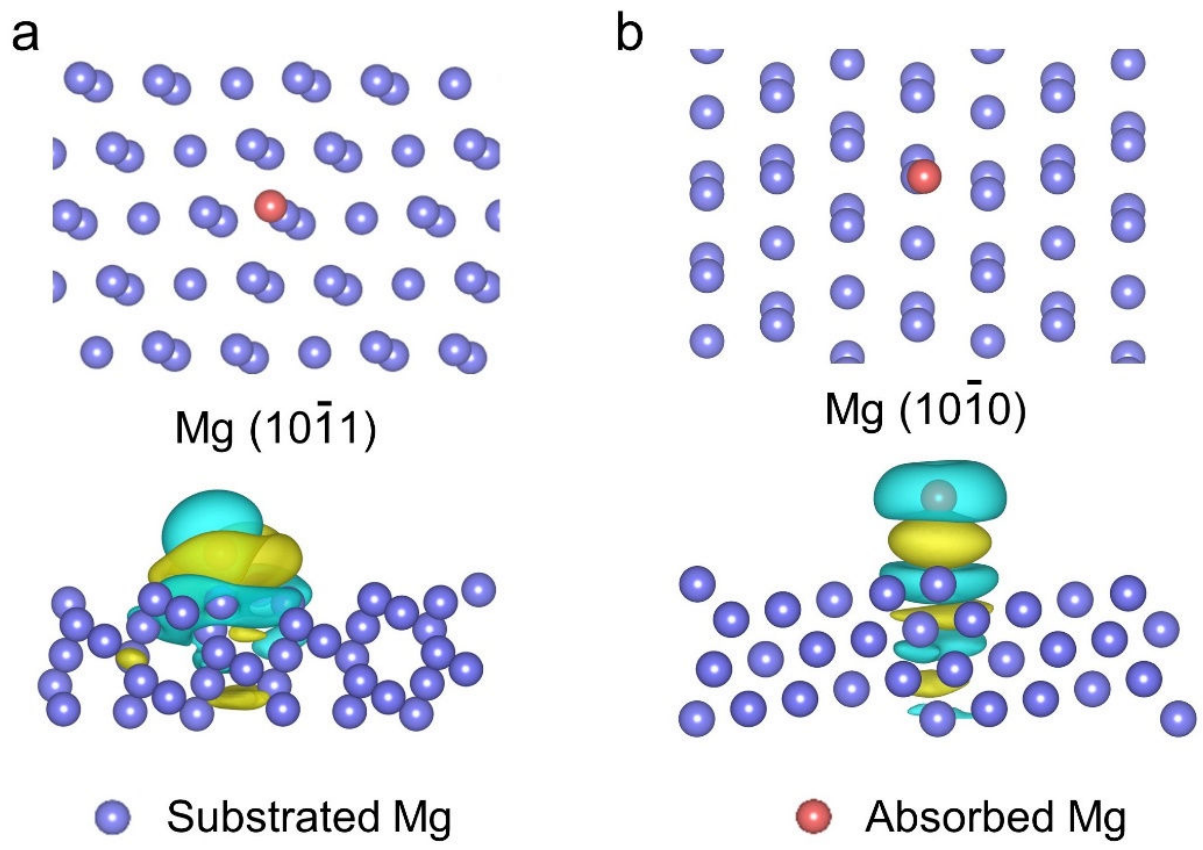

Supplementary Fig. 16. a, b Geometry-optimized atomic configuration and differential charge density of Mg adsorbed Mg(10$\bar{1}$1) (a), and Mg(10$\bar{1}$0) (b).

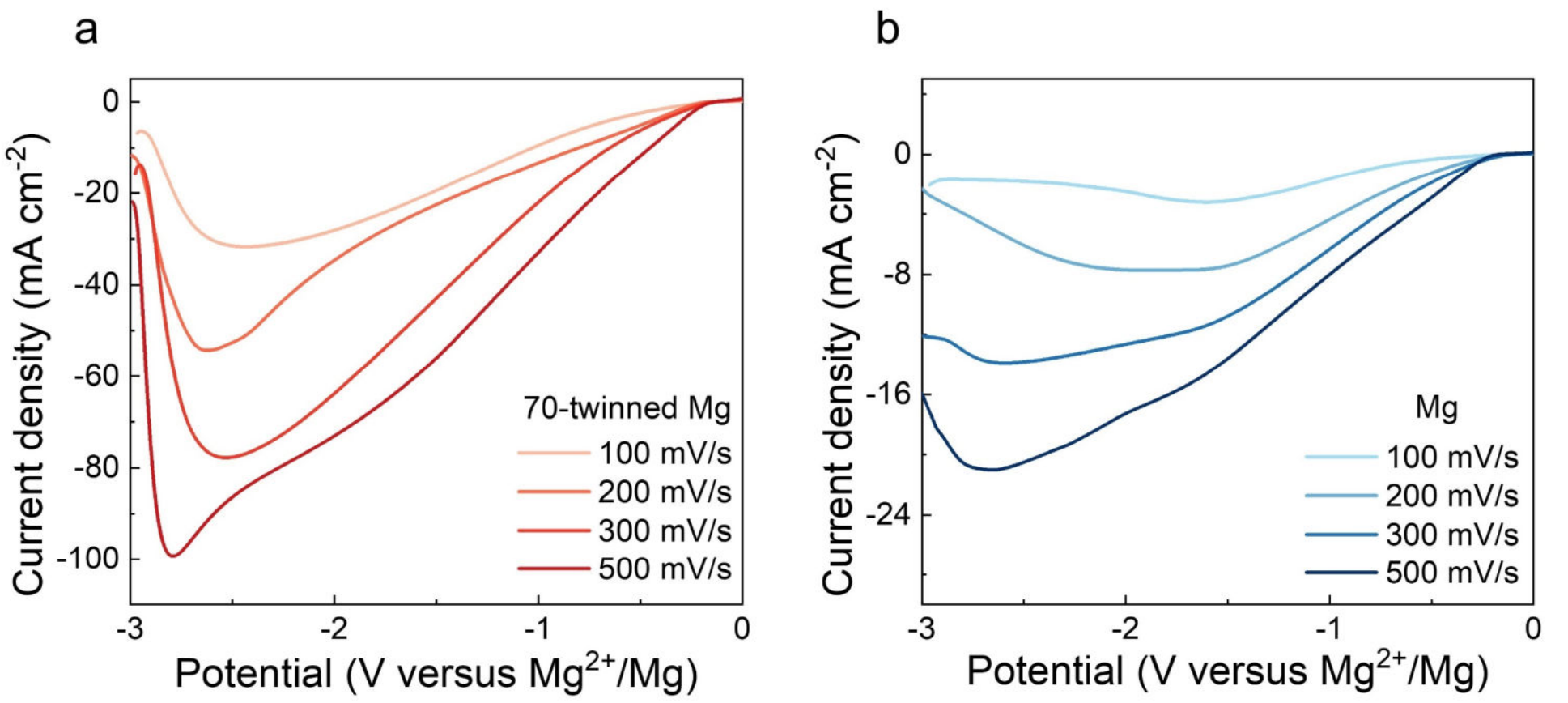

Supplementary Fig. 17. a, b LSV profiles of Mg electrodepositionon the 70-twinned Mg (a) or Mg (b) with a fast scan rate from 100 to 500 mV s^{-1}. All the tests were measured at 25 °C. Source data are provided as a Source Data file.

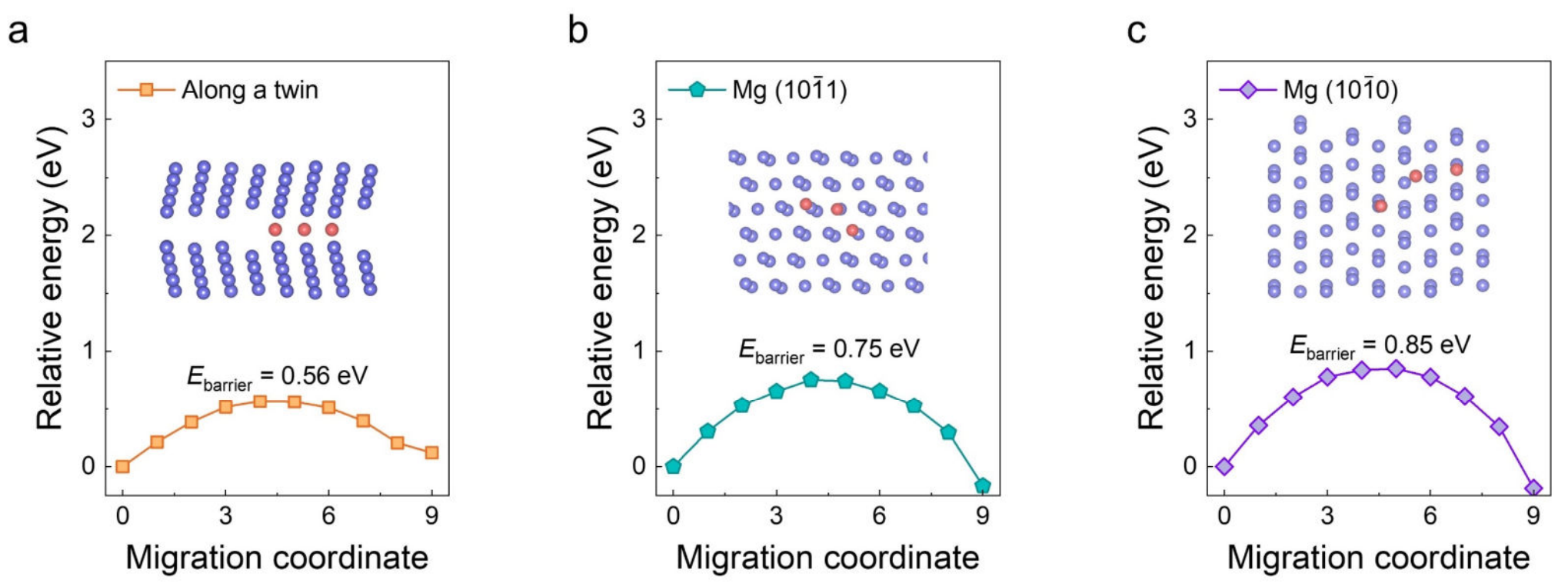

Supplementary Fig. 18. a-c Energy profiles for the diffusion process of Mg on Mg Twin structure (Along a twin) (a), Mg(10$\bar{1}$1) (b), and Mg(10$\bar{1}$0) (c). Insets: schematic representations of the corresponding diffusion pathways for Mg twin structure, Mg(10$\bar{1}$1), and Mg(10$\bar{1}$0). Source data are provided as a Source Data file.

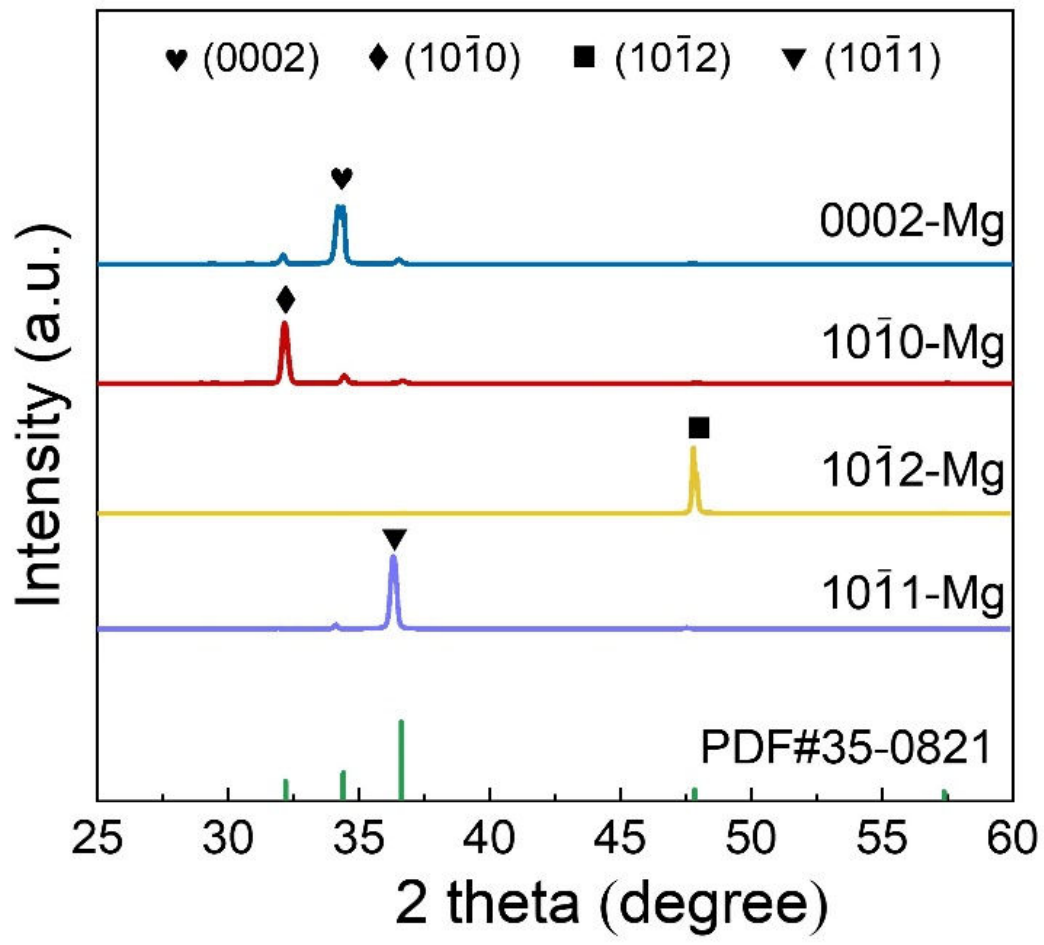

Supplementary Fig. 19. XRD patterns of four Mg single crystals: (0002)-Mg, (10$\bar{1}$1)-Mg, (10$\bar{1}$2)-Mg, and (10$\bar{1}$0)-Mg. Source data are provided as a Source Data file.

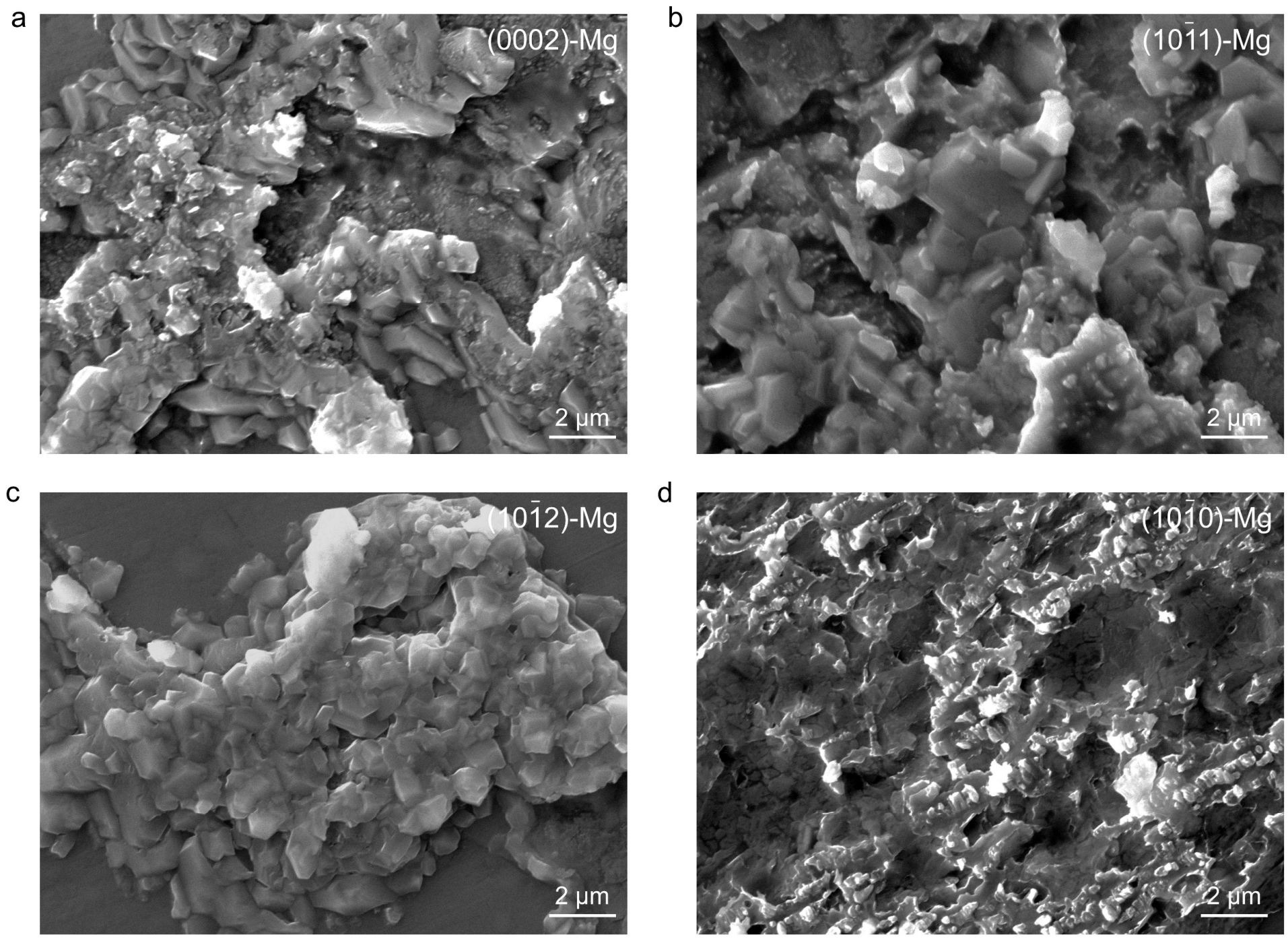

Supplementary Fig. 20. a-d SEM morphology of magnesium deposited on (0002)-Mg, (10$\bar{1}$1)-Mg, (10$\bar{1}$2)-Mg, and (10$\bar{1}$0)-Mg surfaces at a current density of 10 mA cm^{-2} and a capacity of 2 mAh cm^{-2}. All the tests were measured at 25 °C.

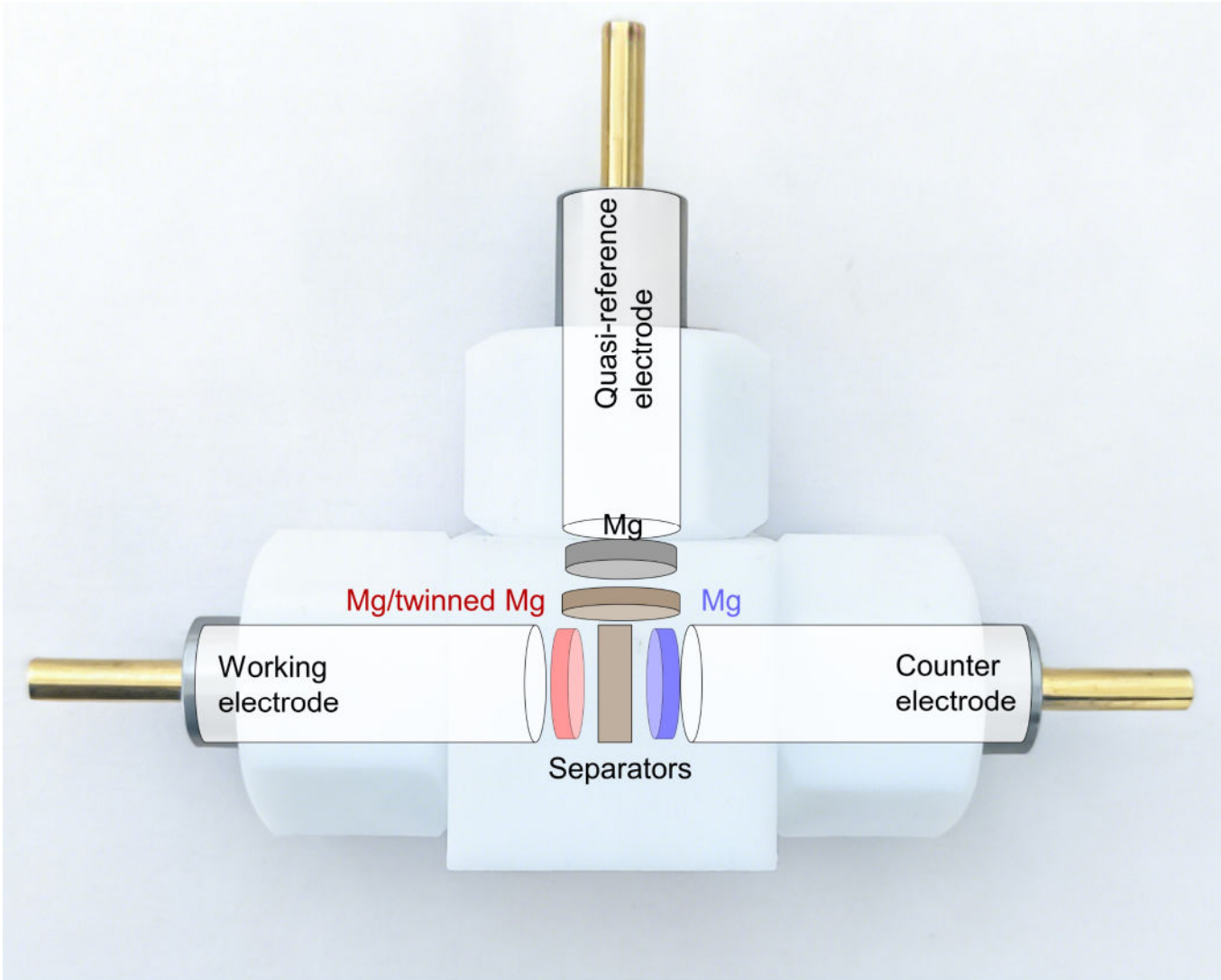

Supplementary Fig. 21. Schematic diagram of the Swagelok-type cell.

a

b

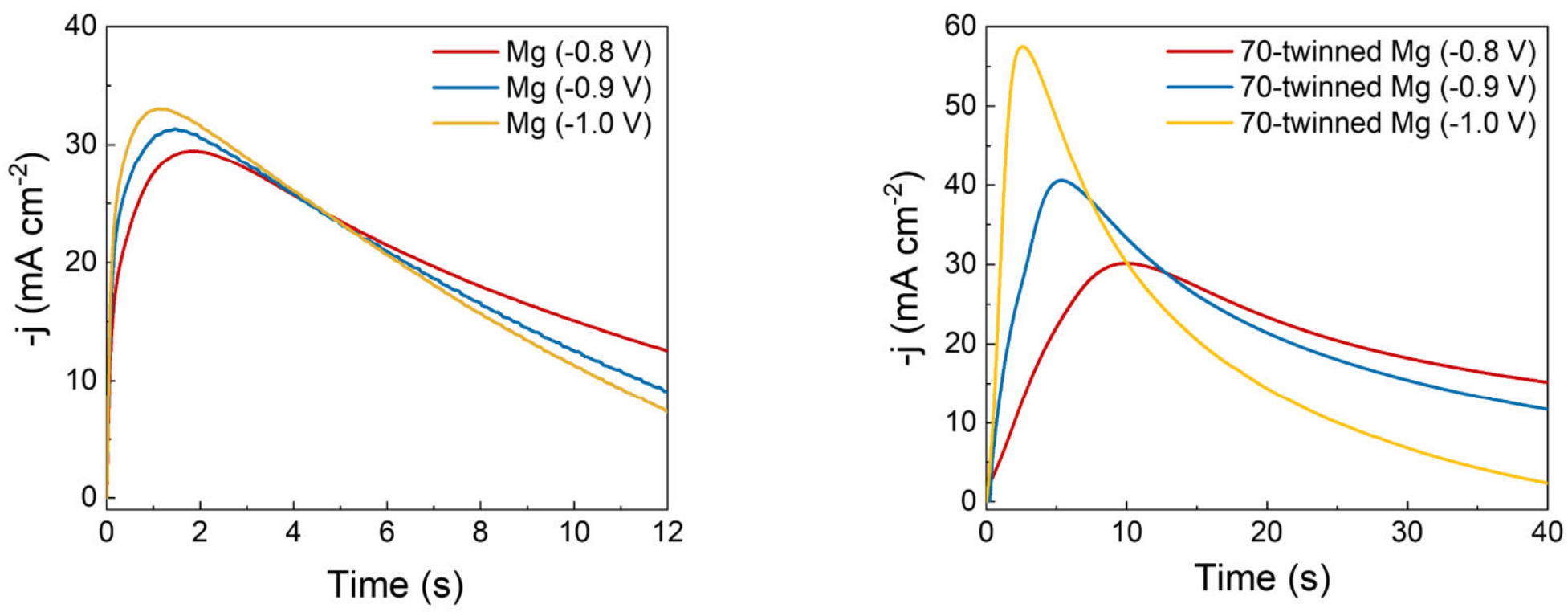

Supplementary Fig. 22. a, b Current-time transients obtained at -0.8 V, -0.9 V, -1.0 V potentials of pure Mg (a) and 70-twinned Mg (b). All the tests were measured at 25 °C. Source data are provided as a Source Data file.

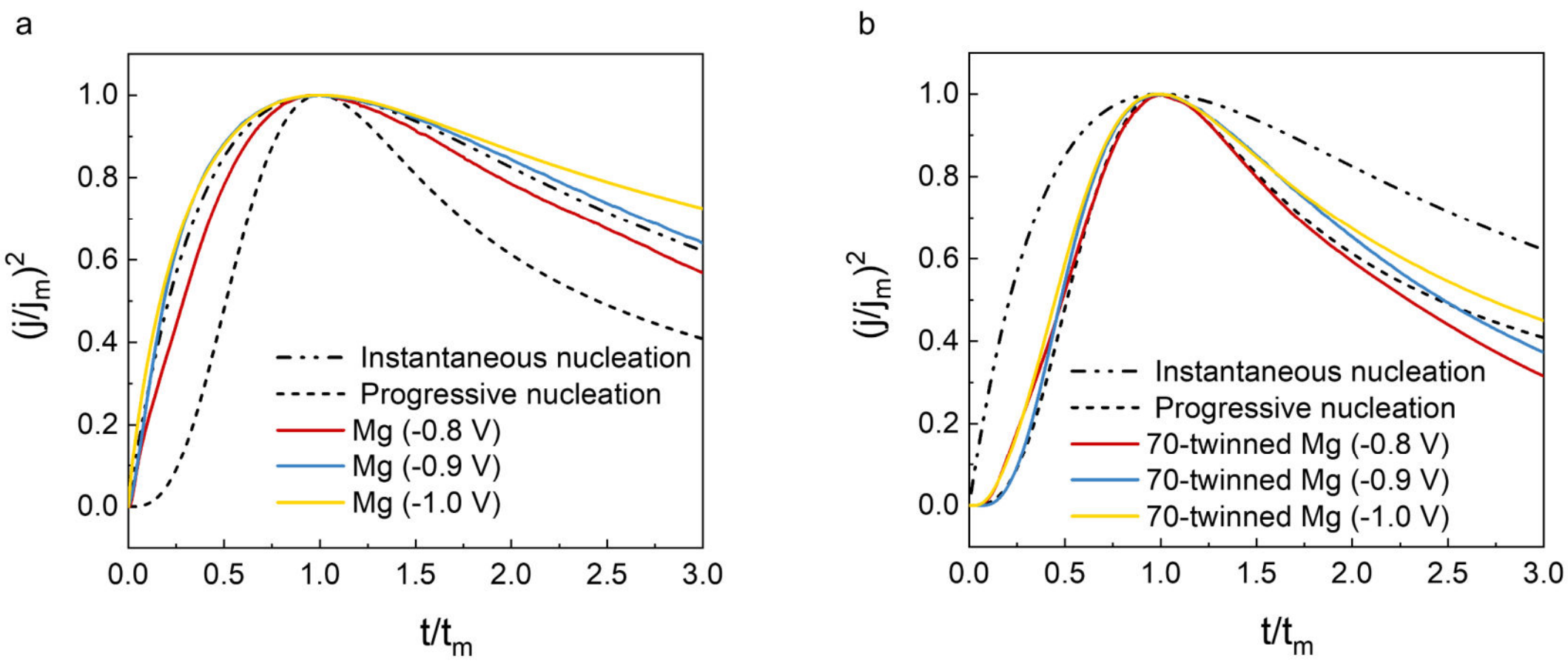

Supplementary Fig. 23. a, b Plots of t/tm and $(j/j_{\mathrm{m}})^2$ in comparison with theoretical 3D models for a Mg cell using Mg (a) and 70-twinned Mg electrodes (b) with 400# roughness. A single dash and a double dash represent theoretical 3D models of progressive nucleation and instantaneous nucleation, respectively. Source data are provided as a Source Data file.

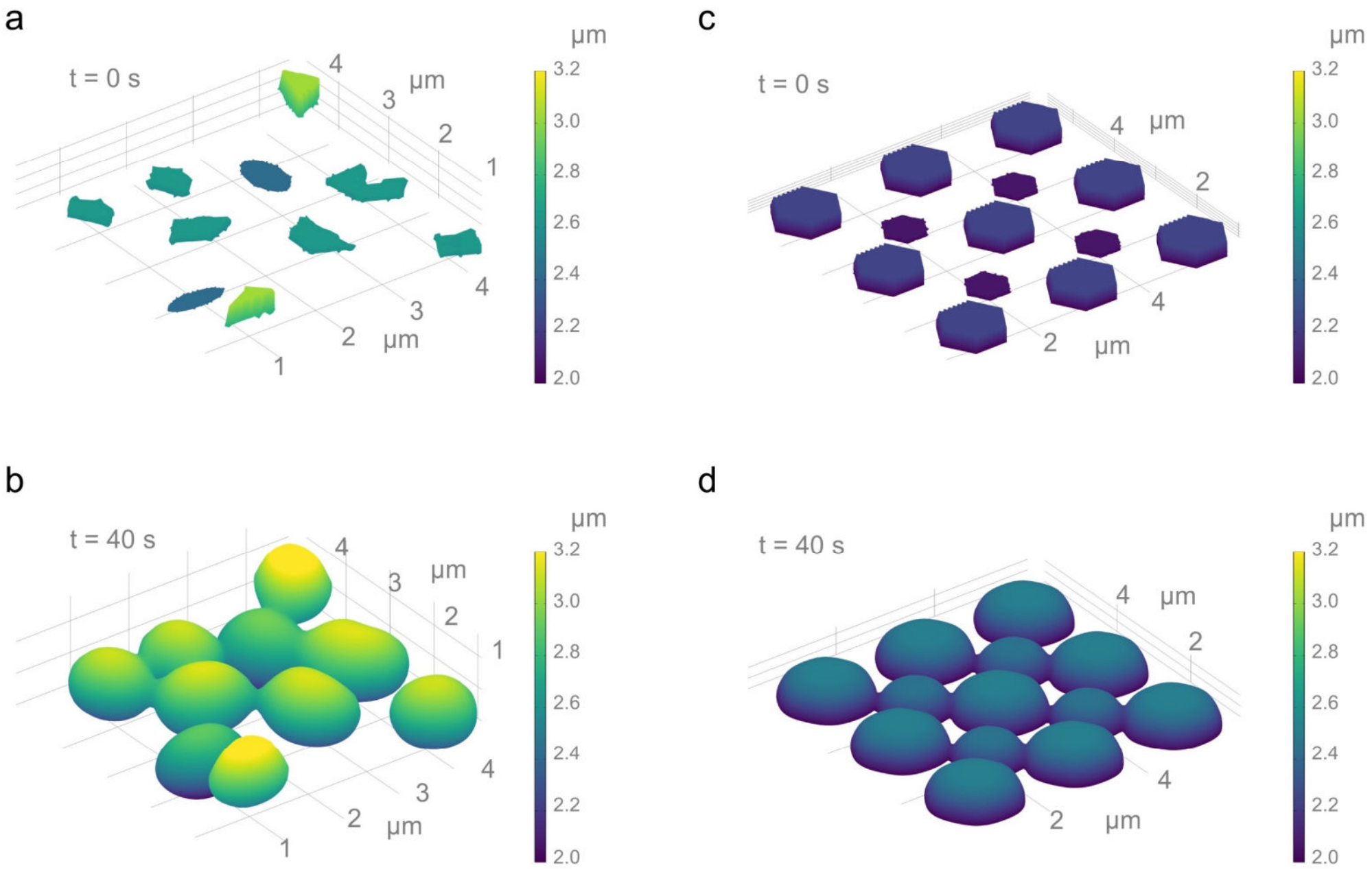

Supplementary Fig. 24. a, b Simulated morphology at 0 s (a) and 40 s (b) under the condition of non-uniform nucleation distribution (representing pure Mg). **c, d** Simulated morphology at 0 s (c) and 40 s (d) under the condition of uniform nucleation distribution (representing twinned Mg).

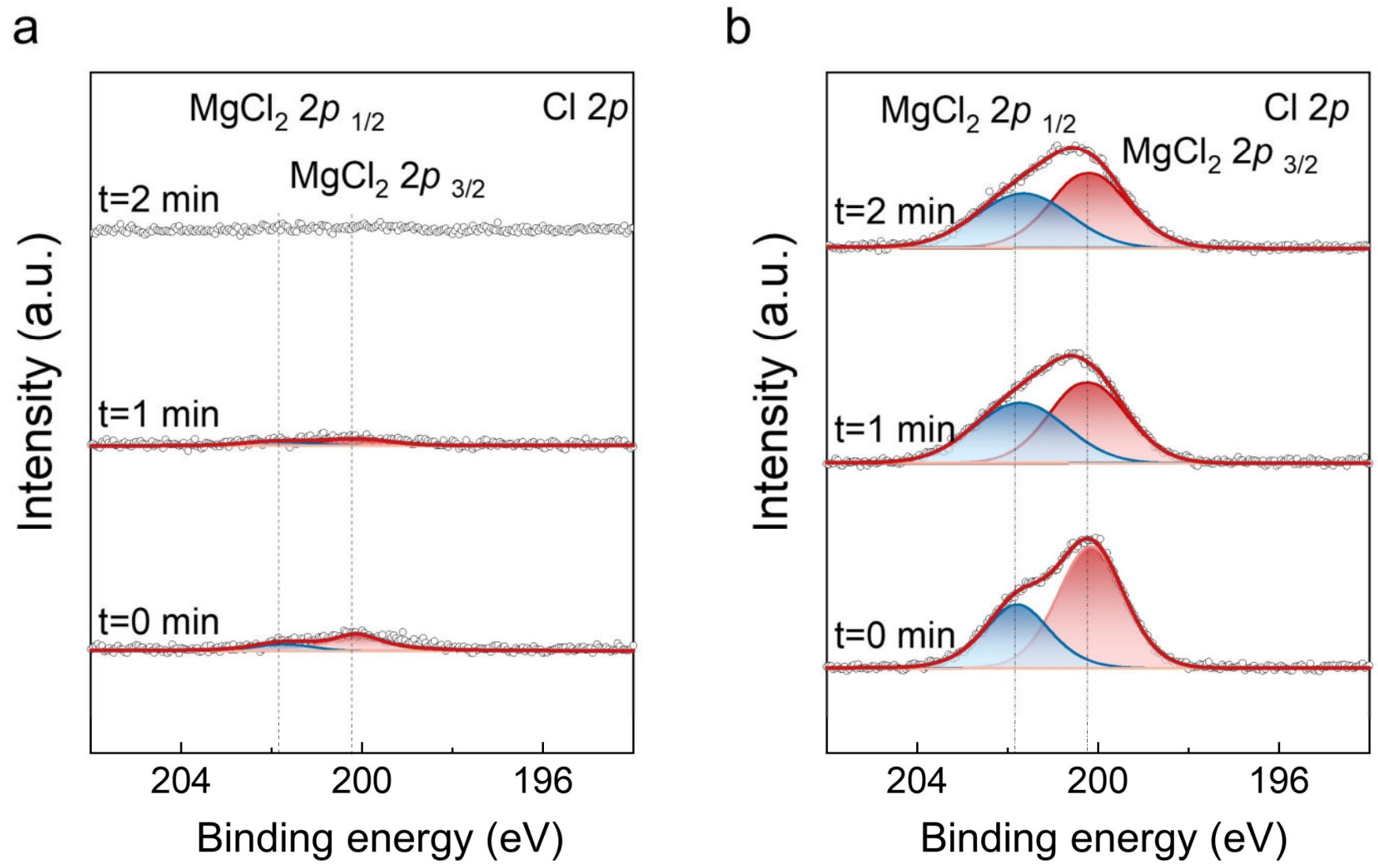

Supplementary Fig. 25. a, b High-resolution XPS spectra of Cl 2*p* of 70-twinned Mg (a) and pristine Mg (b) electrodes after Mg plating/stripping (10 cycles at 10 mA cm^{-2}). The black dot plot represents the original data, the black line represents the total fitted data, and the red, and blue areas represent $MgCl_2$ $2p_{1/2}$ and $MgCl_2$ $2p_{3/2}$, respectively. Source data are provided as a Source Data file. Source data are provided as a Source Data file.

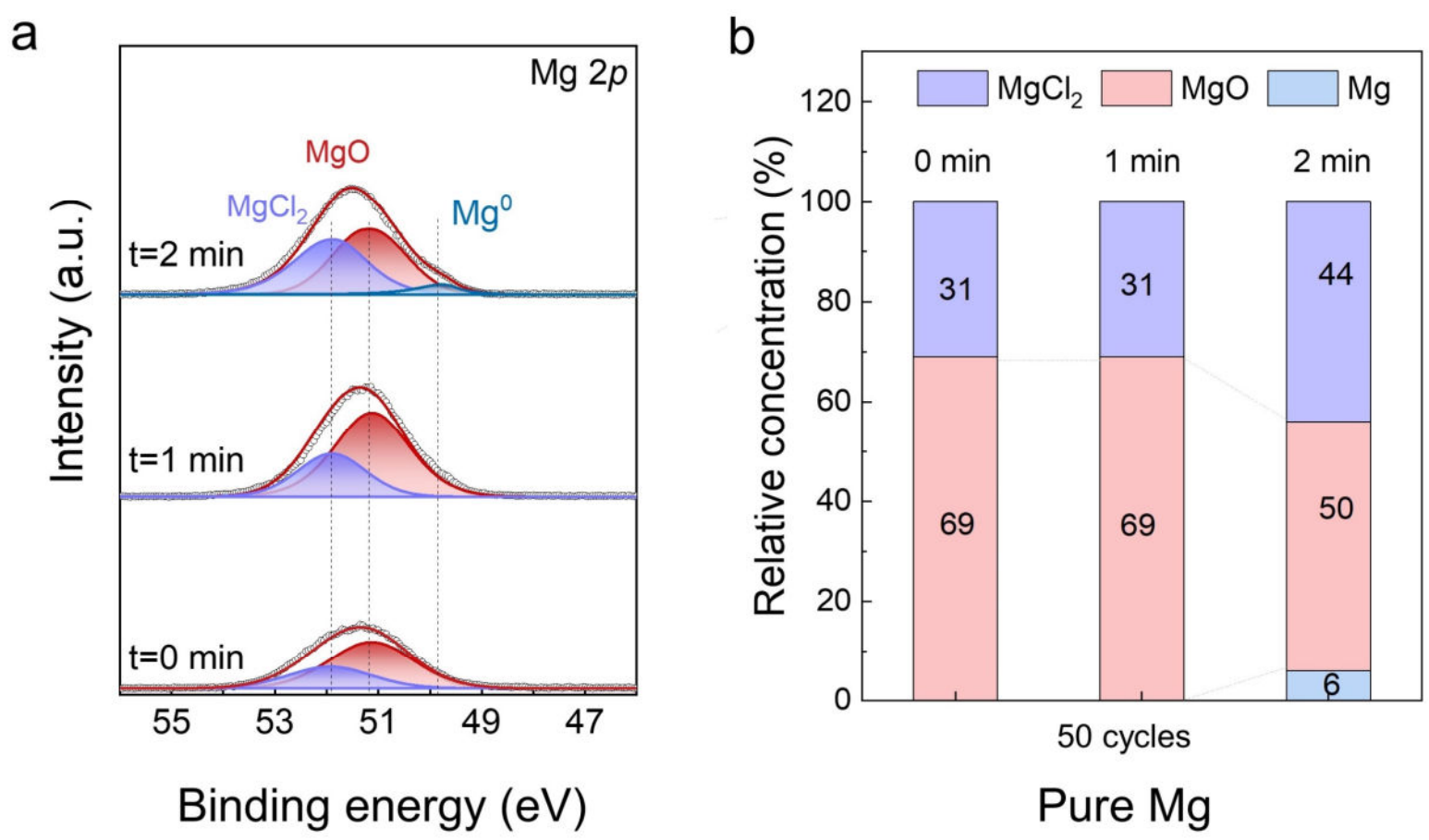

Supplementary Fig. 26. a High-resolution XPS spectra of Mg 2*p* of pure Mg electrodes after Mg plating/stripping (50 cycles) at 10 mA cm^{-2}, 5 mAh cm^{-2}. The black dot plot represents the original data, the black line represents the total fitted data, and the purple, red, and blue areas represent $MgCl_2$, MgO, and MgO, respectively. **b** The relative concentrations of decomposition species measured on the surface after Mg plating/stripping (50 cycles) at sputtering depths of 0, 1 and 2 min. Source data are provided as a Source Data file.

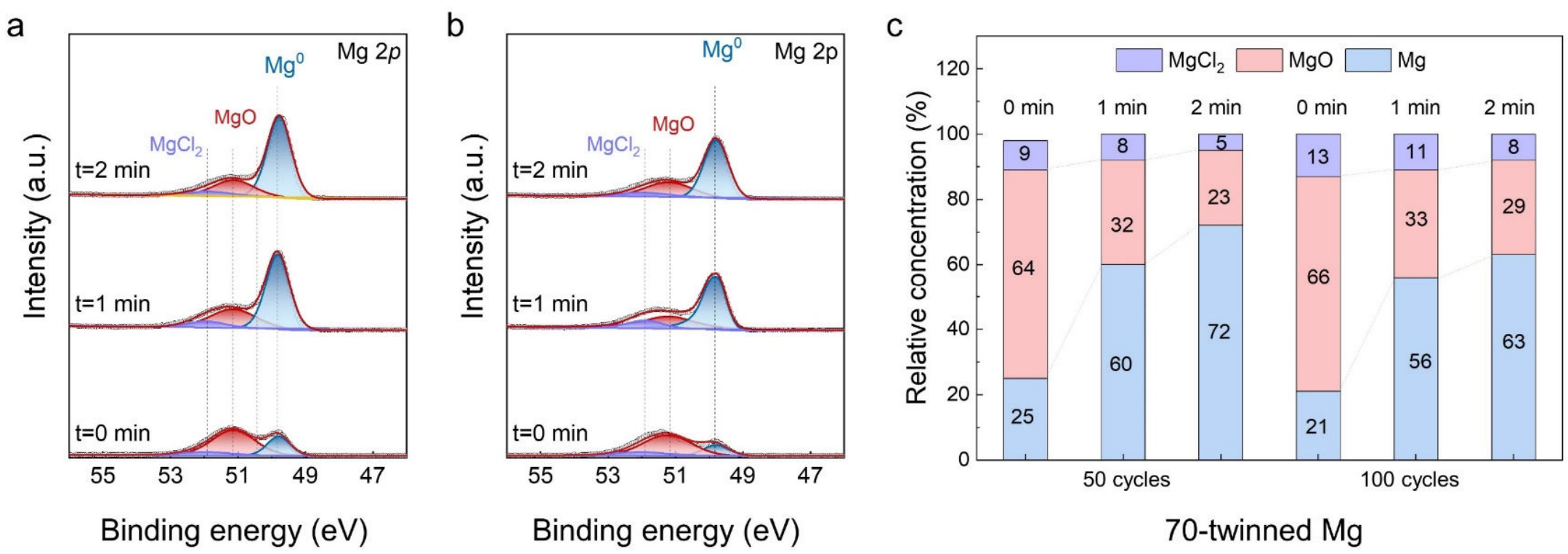

Supplementary Fig. 27. a, b High-resolution XPS spectra of Mg 2*p* of 70-twinned Mg electrodes after Mg plating/stripping (50 cycles) (a) and Mg plating/stripping (100 cycles) (b) at 10 mA cm^{-2}, 5 mAh cm^{-2}. The black dot plot represents the original data, the black line represents the total fitted data, and the purple, red, and blue areas represent $MgCl_2$, MgO, and MgO, respectively. **c** The relative concentrations of decomposition species measured on the surface after Mg plating/stripping (50 cycles) and Mg plating/stripping (100 cycles) at sputtering depths of 0, 1 and 2 min. Source data are provided as a Source Data file.

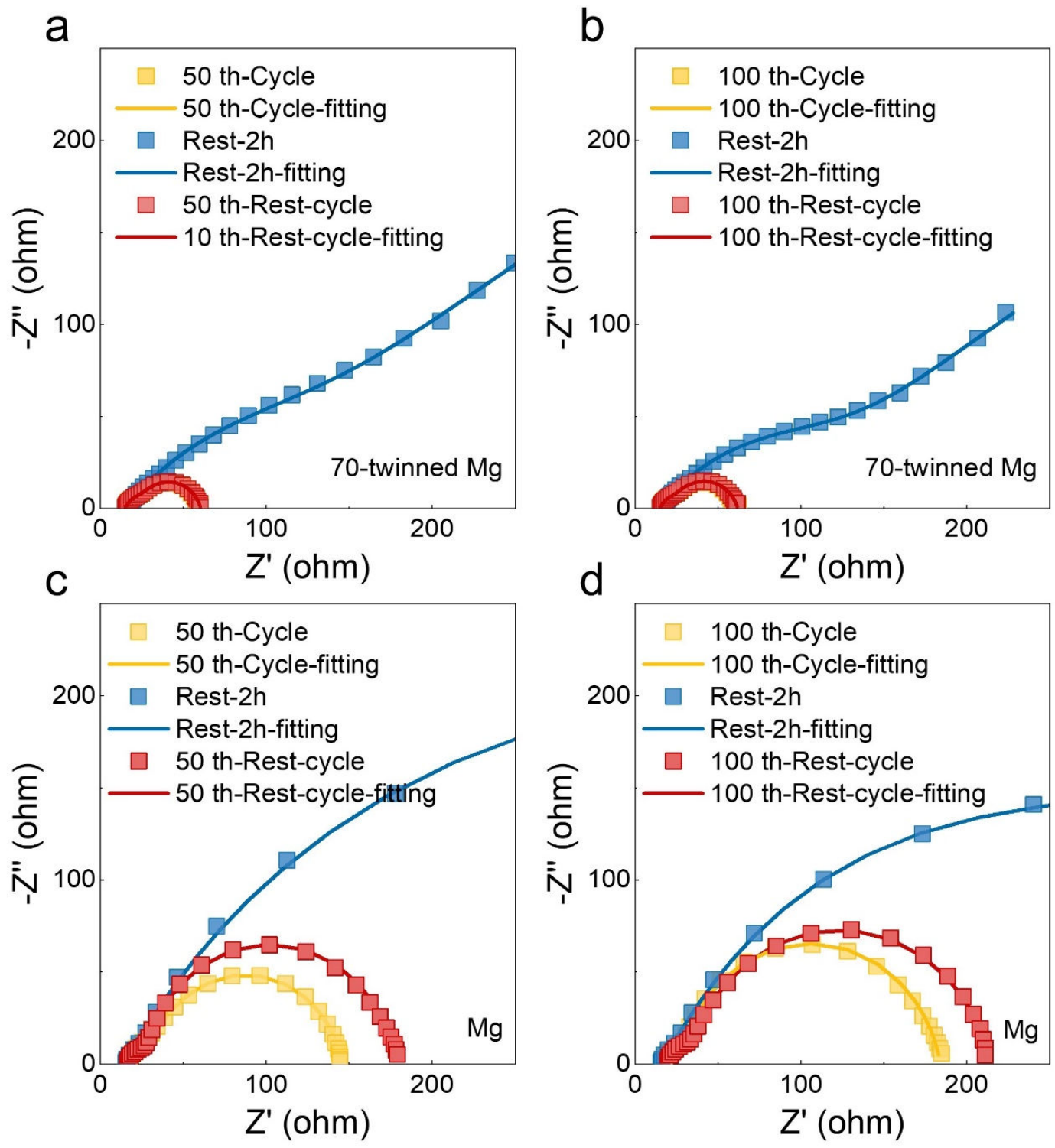

Supplementary Fig. 28. a-d The in situ EIS spectra of a 70-twinned Mg||70-twinned Mg (a, b) and a Mg||Mg cell (c, d) cell measured after Mg plating/stripping (50 cycles) (a, c) and Mg plating/stripping (100 cycles) (b, d), resting for 2 h, and re- plating/stripping period, under conditions of 10 mA cm^{-2} and 1 mAh cm^{-2}. All the tests were measured at 25 °C. Source data are provided as a Source Data file.

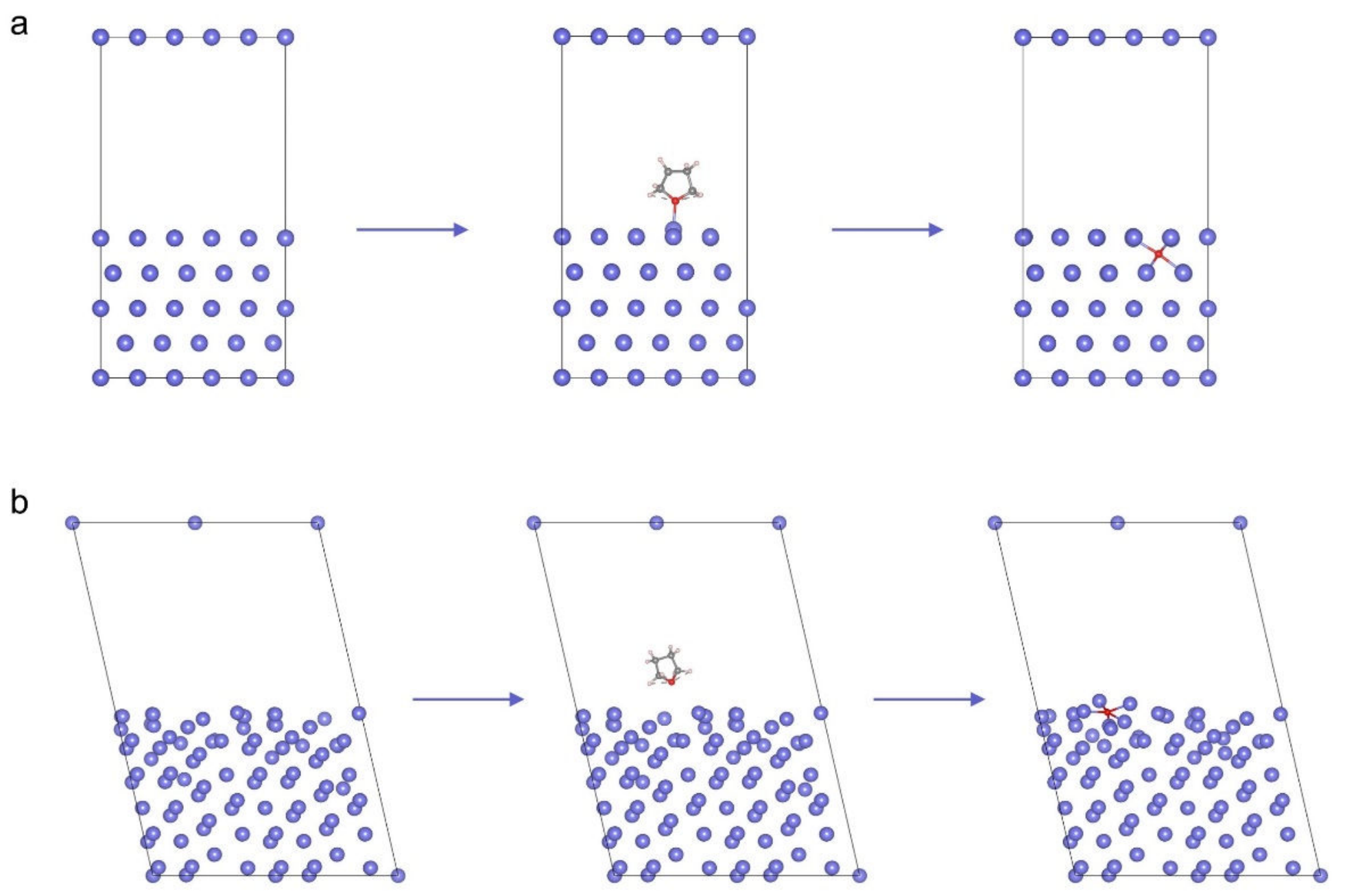

Supplementary Fig. 29. a, b Configurational changes before and after adsorption of THF molecules on pure Mg (a) and 70-twinned Mg (b) surfaces. (red, oxygen; grey, carbon; white, hydrogen; and purple, Mg).

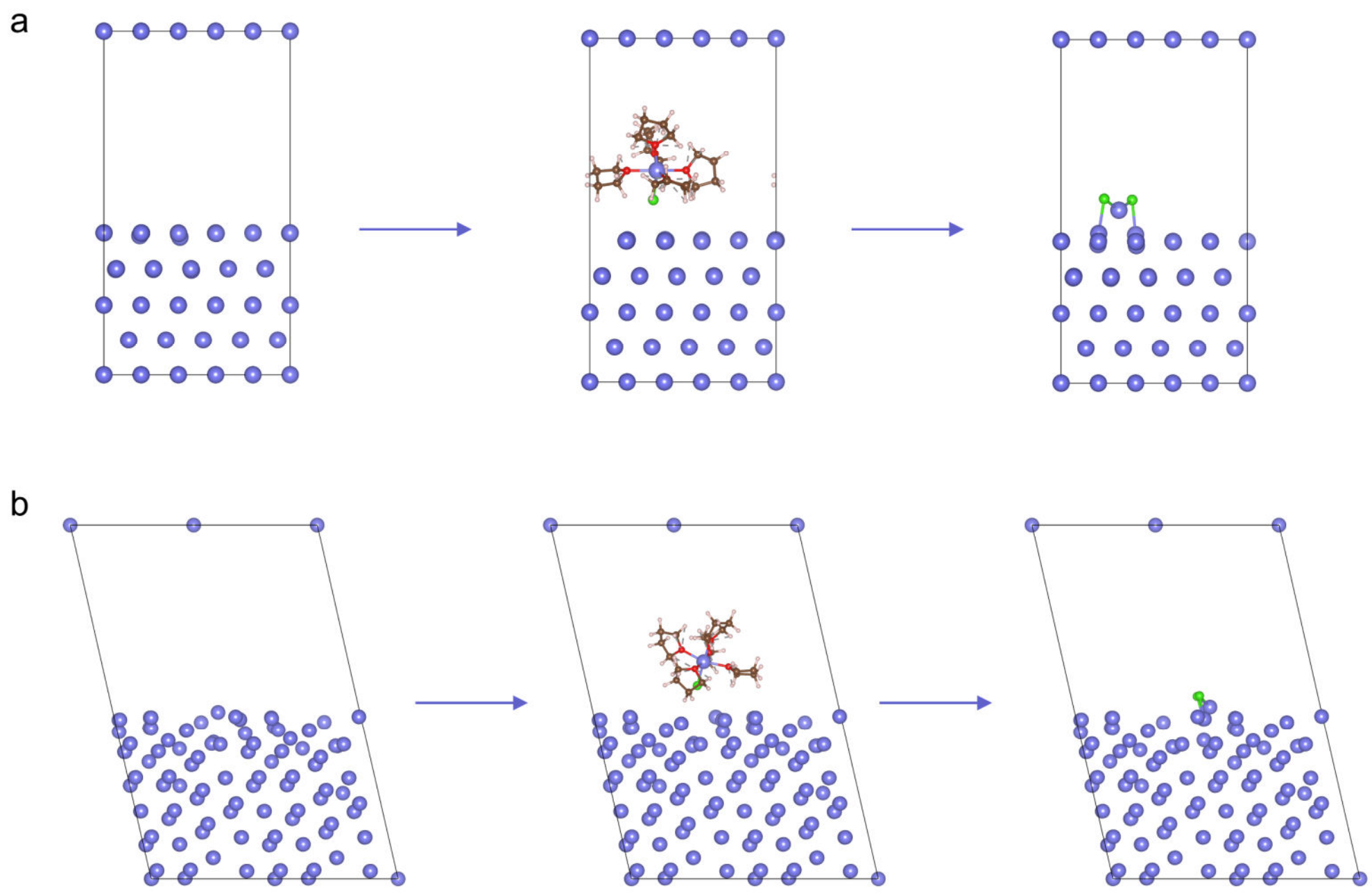

Supplementary Fig. 30. a, b Configurational changes before and after adsorption of $[MgCl{\cdot}5THF]^{+}$ on pure Mg (a) and 70-twinned Mg (b) surfaces. (red, oxygen; green, chlorine; grey, carbon; white, hydrogen; and purple, Mg).

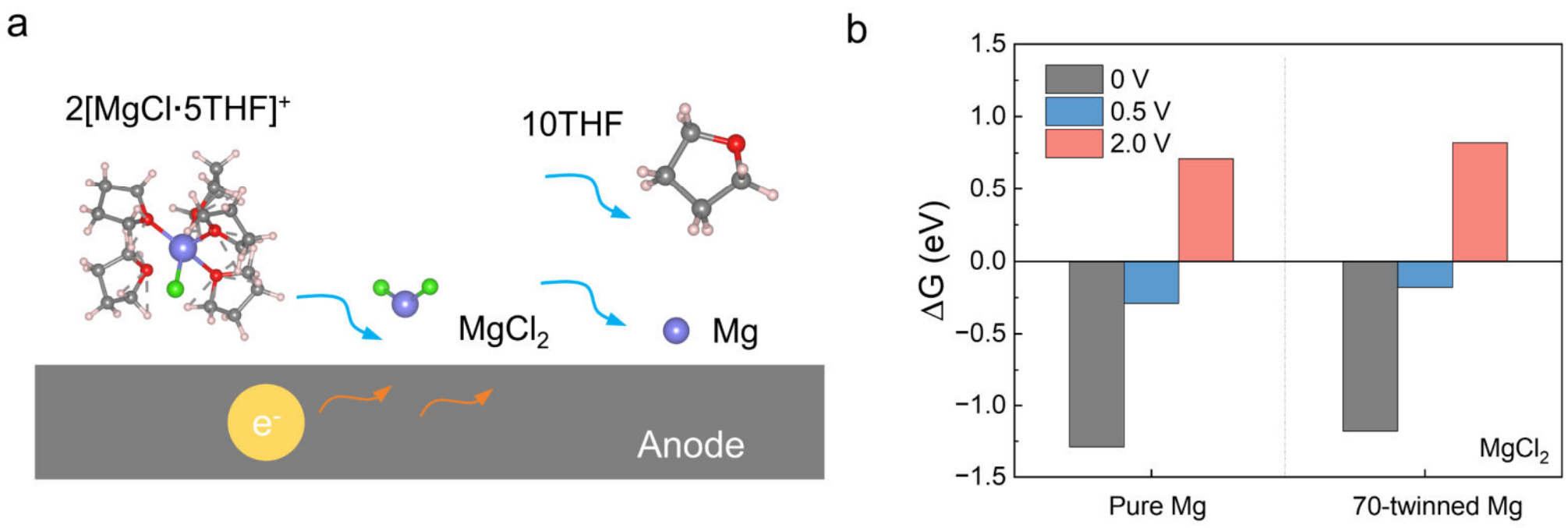

Supplementary Fig. 31. Theoretical calculation of the Gibbs free energy change of the reaction forming THF, adsorbed Mg or $MgCl_2$ on different negative electrodes. **a** Scheme of the calculation model (red, oxygen; green, chlorine; grey, carbon; white, hydrogen; and purple, Mg). **b** The free energy calculation results. (The reason for choosing potentials of 0.5 V and 2.0 V is that the overpotential of a 70-twinned Mg symmetric cell at 10 mA cm^{-2} is 0.43V (~0.5 V); while the overpotential of a pure Mg symmetric cell at 10 mA cm^{-2} is 2.26V (~2.0 V)). Source data for **(b)** are provided as a Source Data file.

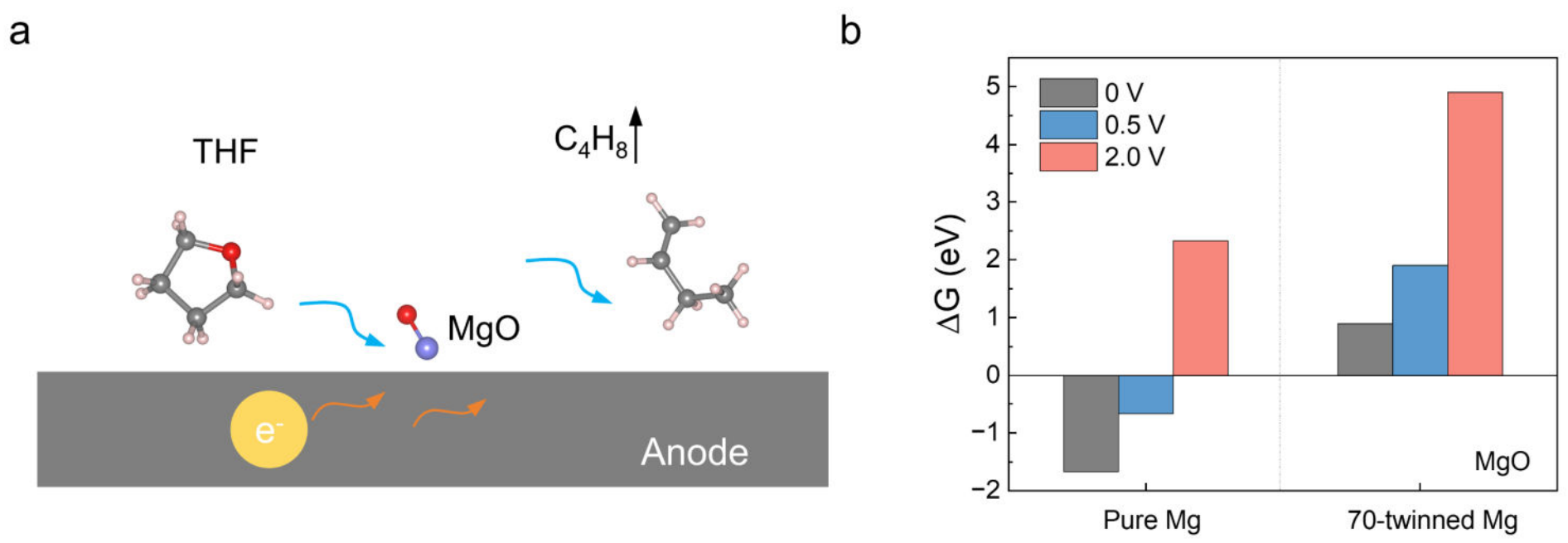

Supplementary Fig. 32. Theoretical calculation of the Gibbs free energy change of the reaction forming C_4H_8 or MgO on different negative electrodes. a) Scheme of the calculation model (red, oxygen; grey, carbon; white, hydrogen; and purple, Mg); b) the free energy calculation results. (The reason for choosing potentials of 0.5 V and 2.0 V is that the overpotential of a 70-twinned Mg symmetric cell at 10 mA cm^{-2} is 0.43V (~0.5 V); while the overpotential of a pure Mg symmetric cell at 10 mA cm^{-2} is 2.26V (~2.0 V)). Source data for **(b)** are provided as a Source Data file.

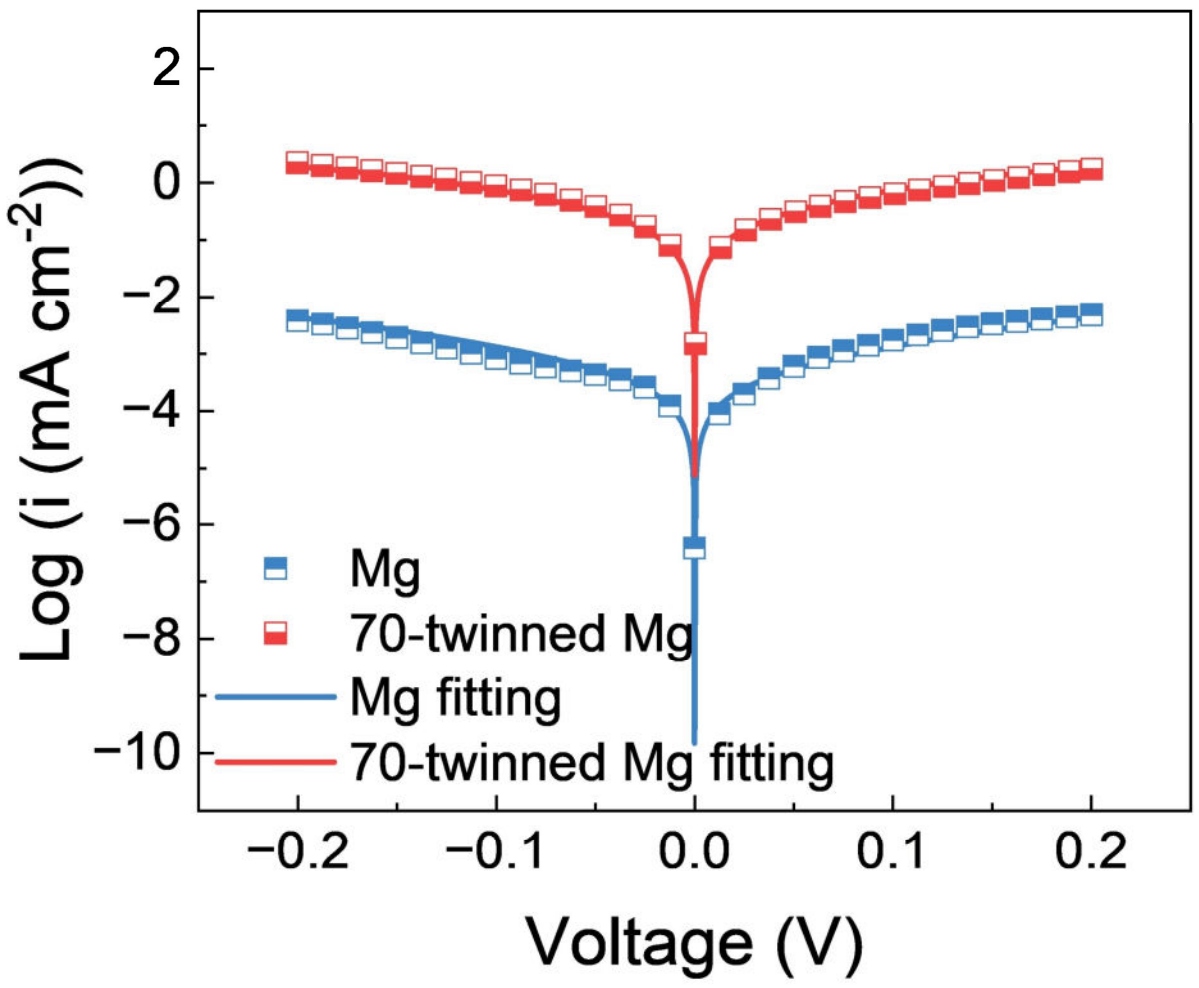

Supplementary Fig. 33. The Tafel plots for Mg plating/stripping in Mg and 70-twinned Mg electrodes and fitting with Marcus-Hush-Chidsey kinetics. The scan rate is 10 mV/s. All the tests were measured at 25 °C. Source data are provided as a Source Data file.

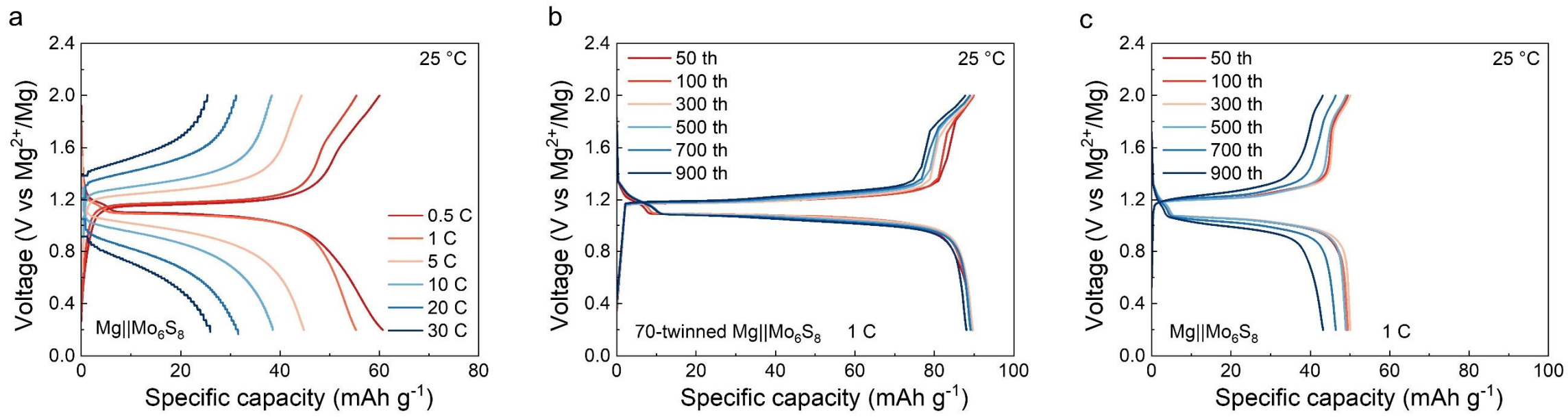

Supplementary Fig. 34. **a** Charge-discharge curves corresponding to the rate performance of Mg||Mo_6S_8 full cells. **b, c** Charge-discharge curves corresponding to the performance of 70-twinned Mg||Mo_6S_8 (b) and Mg||Mo_6S_8 (c) full cells at 128 mA g^{-1}. All the tests were measured at 25 °C. Source data are provided as a Source Data file.

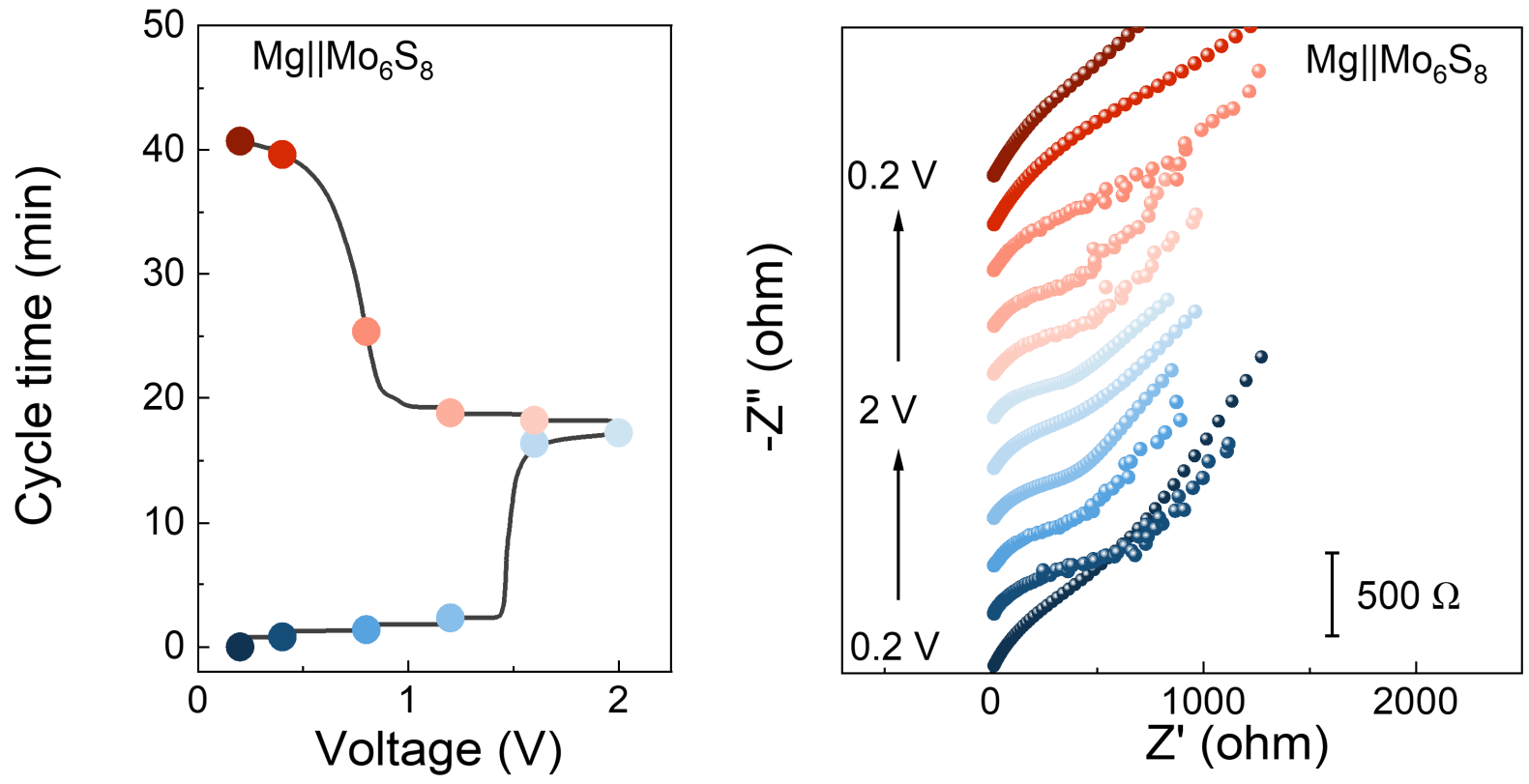

Supplementary Fig. 35. In situ EIS results of the Mg||Mo_6S_8. All the tests were measured at 25 °C. Source data are provided as a Source Data file.

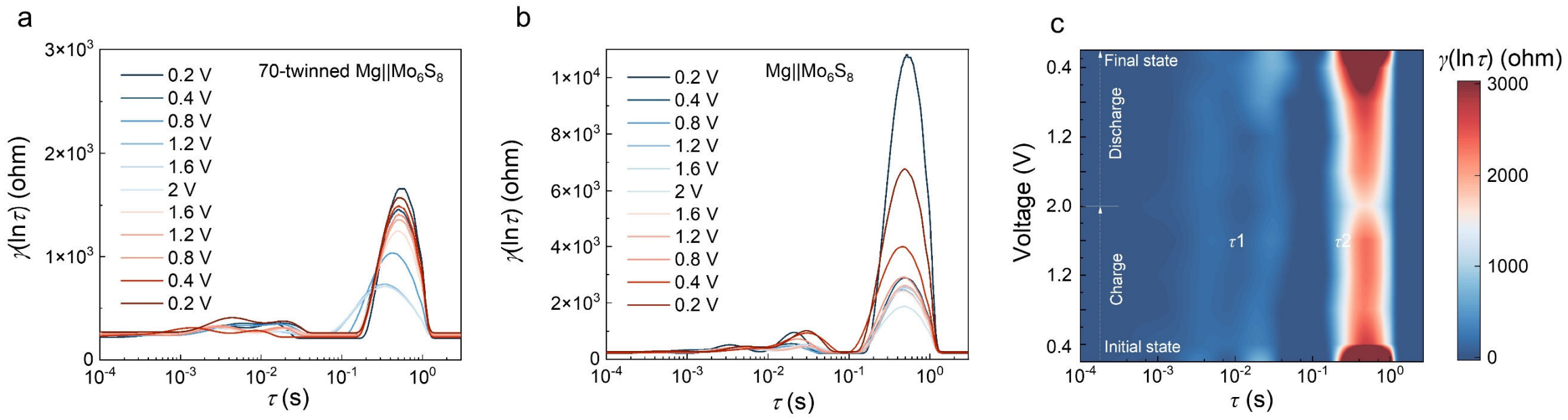

Supplementary Fig. 36. a, b Distribution of Relaxation Times (DRT) calculated from the in situ EIS for the discharge and charge process of (a) 70-twinned Mg||Mo_6S_8 and (b) Mg||Mo_6S_8. **c** Corresponding contour plots of the calculated DRT results from Mg||Mo_6S_8. Source data are provided as a Source Data file.

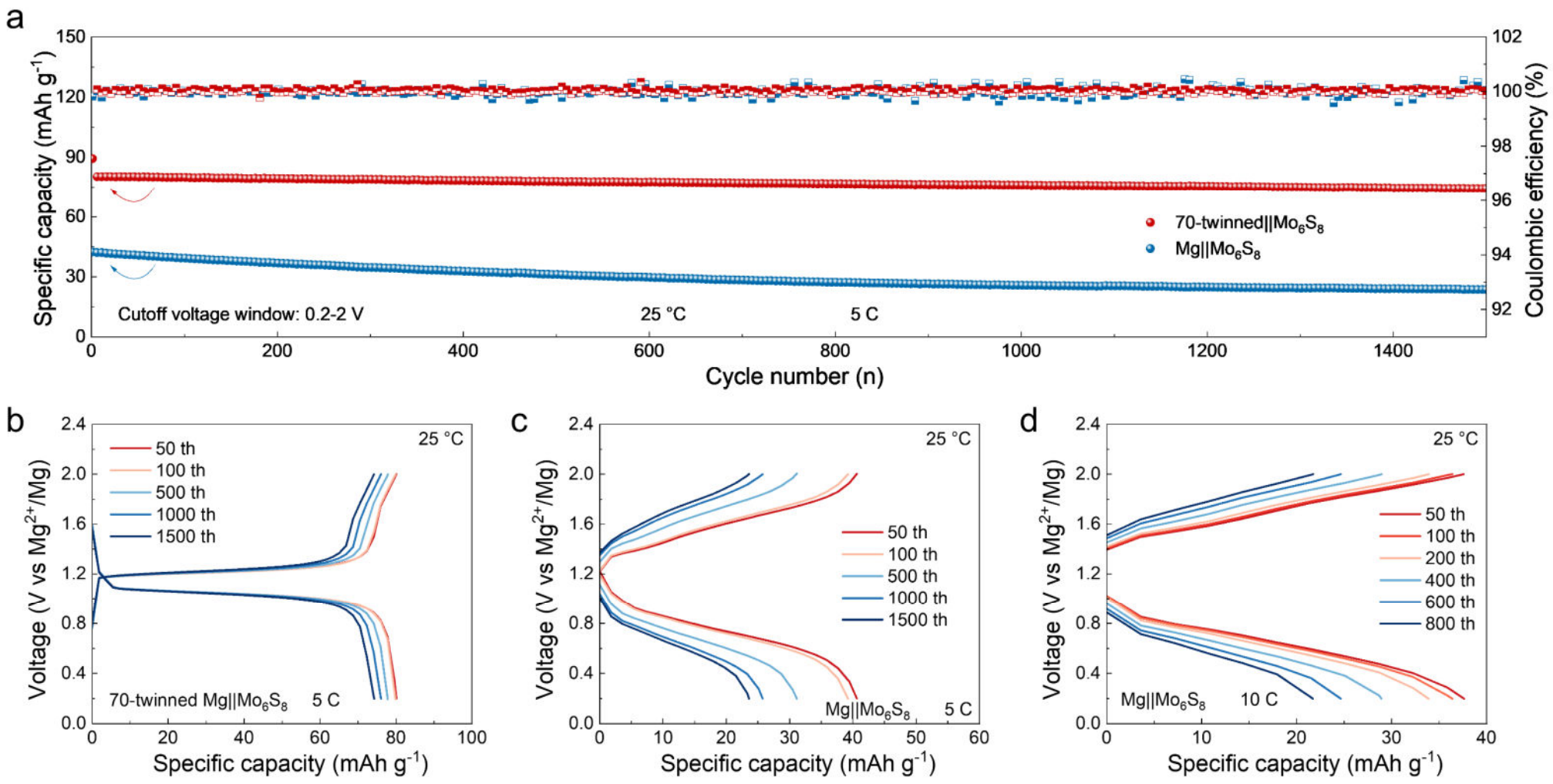

Supplementary Fig. 37. a Cycling stability of the Mg||Mo_6S_8 and 70-twinned Mg||Mo_6S_8 cells at 5 C. **b, c** Charge-discharge curves corresponding to the performance of 70-twinned Mg||Mo_6S_8 and Mg||Mo_6S_8 full cells at 5 C. **d** Charge-discharge curves corresponding to the performance of Mg||Mo_6S_8 full cells at 10 C. All the tests were measured at 25 °C. Source data are provided as a Source Data file.

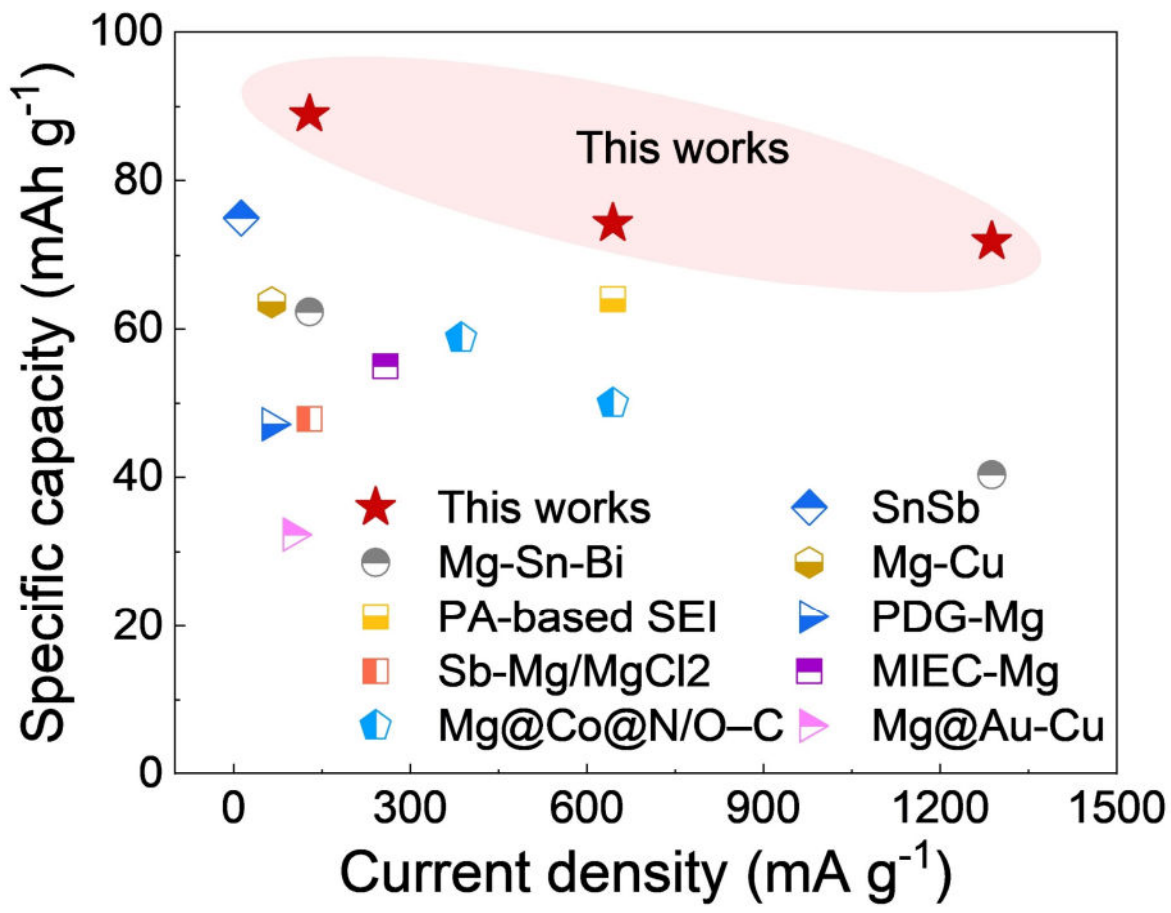

Supplementary Fig. 38. Comparison of specific capacity between the 70-twinned Mg||Mo_6S_8 cell and previously reported Mo_6S_8-based full cells at different current densities. The source of the literature data shown in this figure can be found in Supplementary Information, Table S6. Source data are provided as a Source Data file.

a b

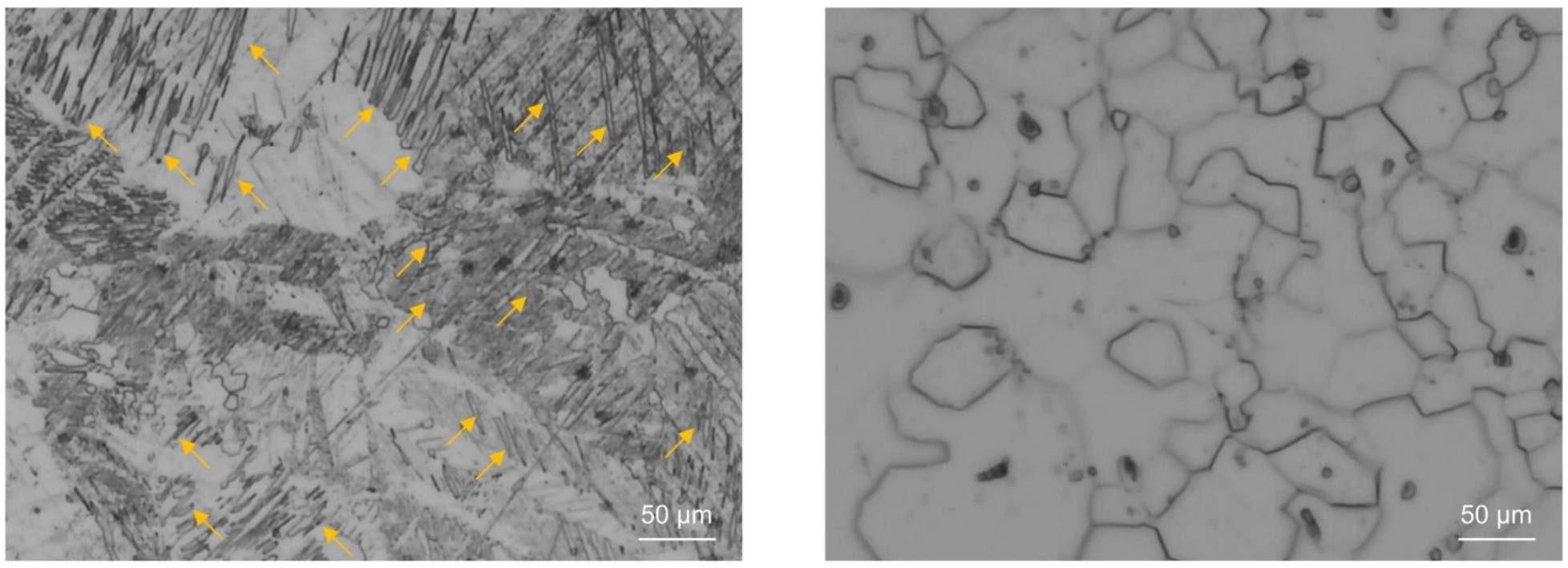

Supplementary Fig. 39. The microstructure of the twinned Zn (a) and pure Zn (b) electrodes. The yellow arrows point to twin regions within the grains.

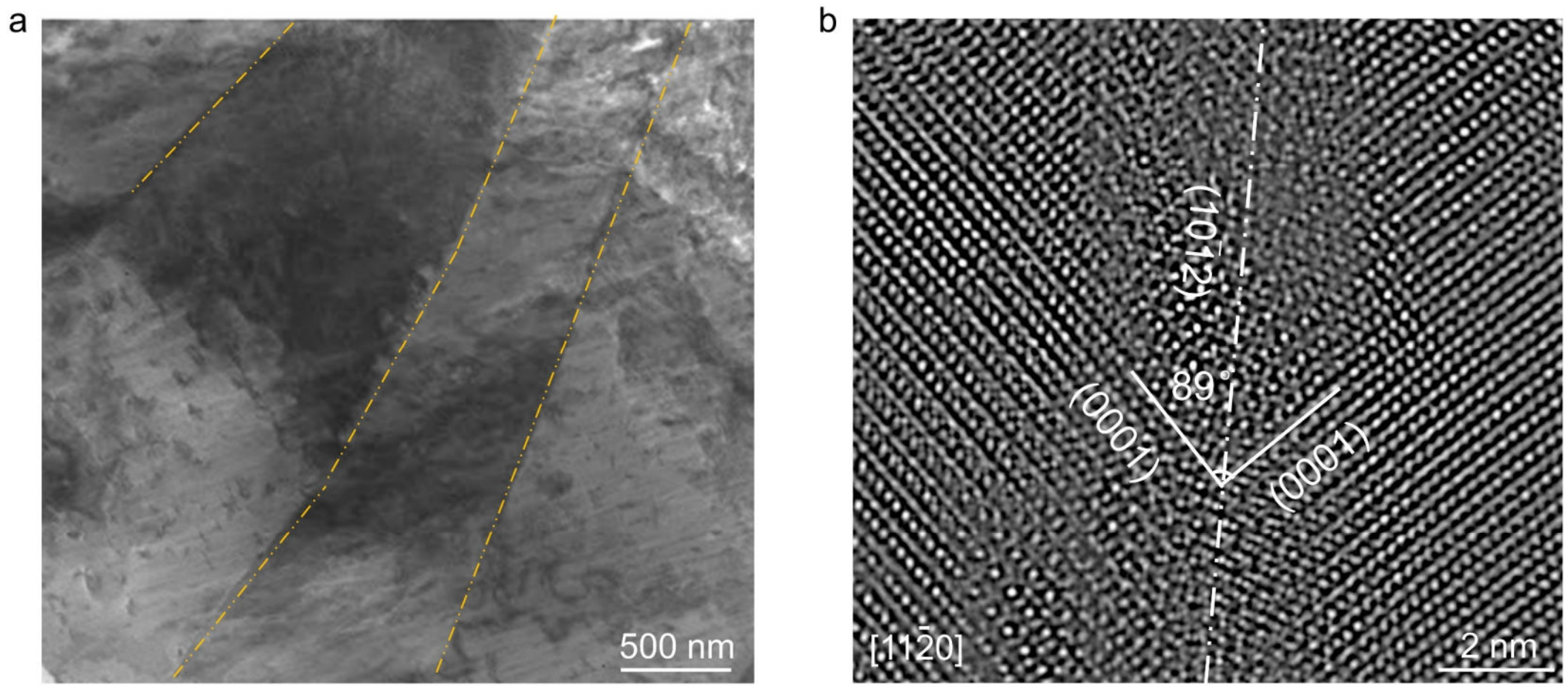

Supplementary Fig. 40. a TEM image of the twinned Zn. **b** Atomic resolution HAADF image of the (10$\bar{1}$2) twins taken along the [11$\bar{2}$0] axis.

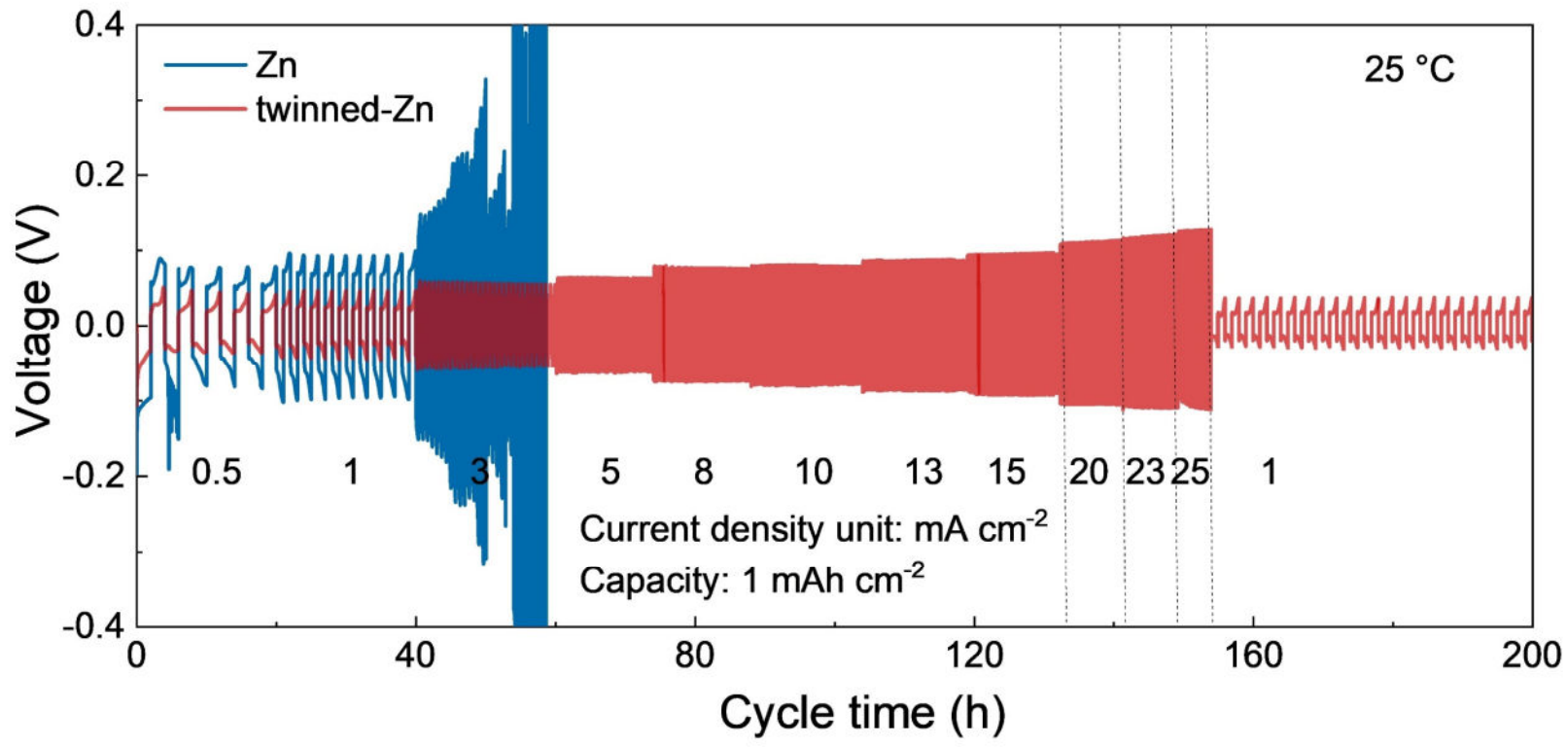

Supplementary Fig. 41. Rate performance of the pure Zn and twinned Zn symmetric cells at different current densities from 0.5 to 25 mA cm^{-2} with the same capacity of 1 mAh cm^{-2}. All the tests were measured at 25 °C. Source data are provided as a Source Data file.

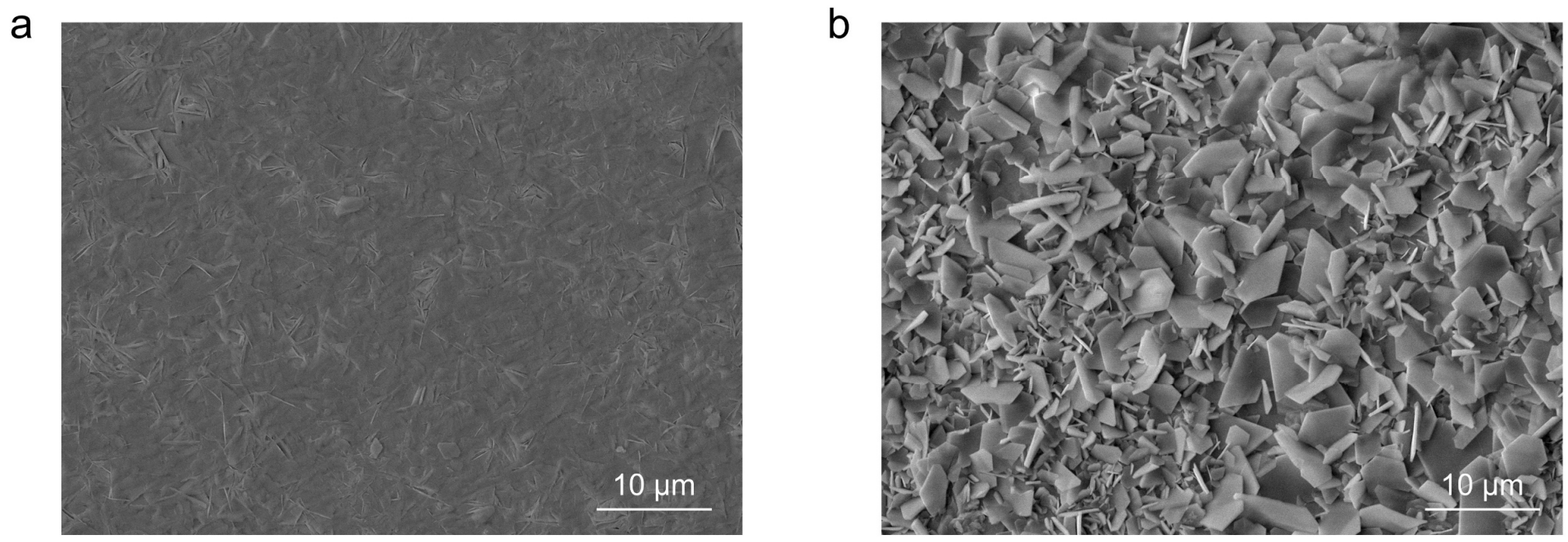

Supplementary Fig. 42. SEM images of twinned Zn (a) and pure Zn (b) electrodes after Zn plating/stripping under variable current density conditions. All the tests were measured at 25 °C.

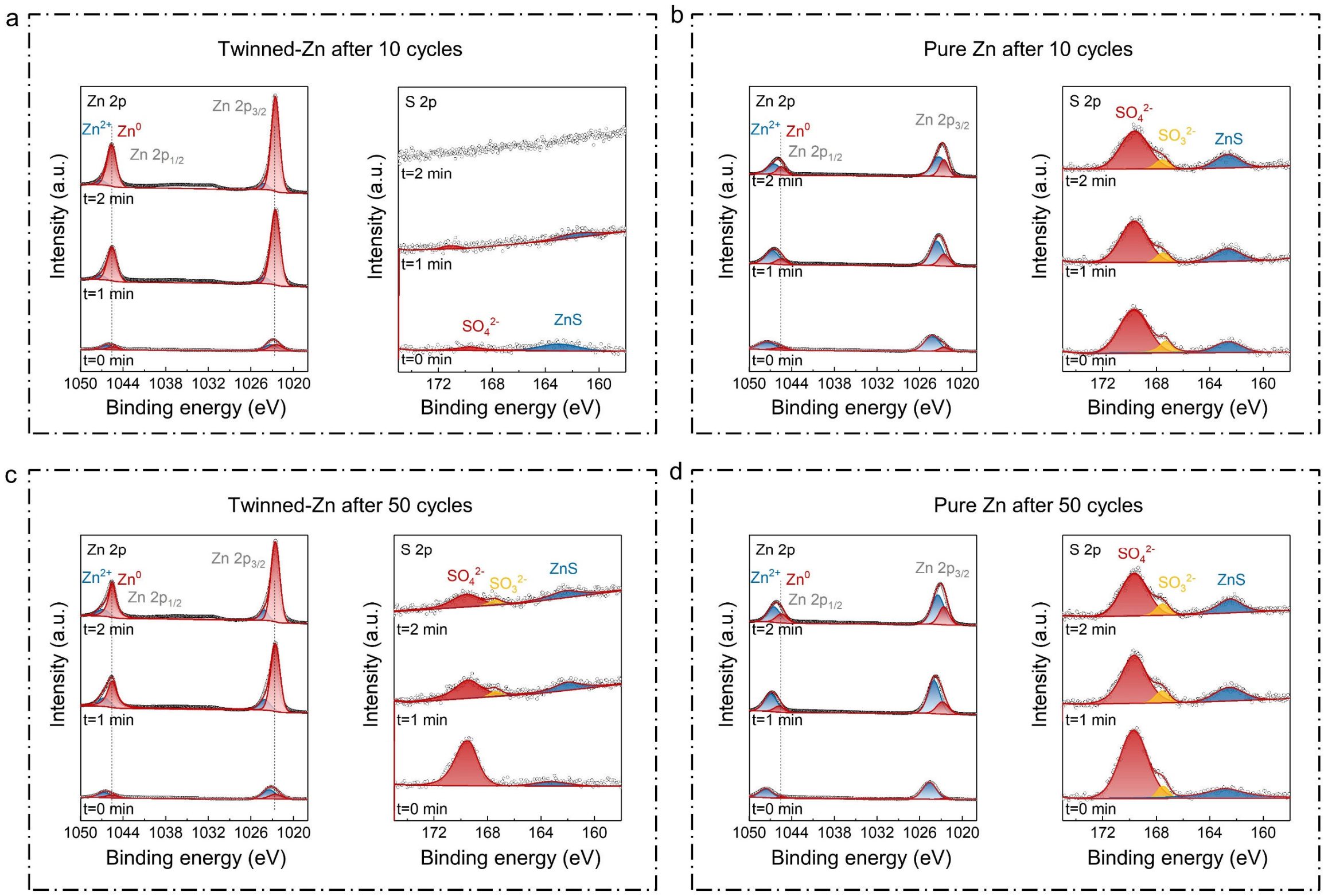

Supplementary Fig.43. a, b High-resolution XPS spectra of Zn 2*p* and S 2*p* of twinned Zn (a) and pure Zn (b) after Zn plating/stripping (10 cycles). c, d High-resolution XPS spectra of Zn 2*p* and S 2*p* of twinned Zn (c) and pure Zn (d) after Zn plating/stripping (50 cycles). Source data are provided as a Source Data file.

Supplementary Note 1

Mg has a hexagonal close-packed crystal structure, among which $(10\bar{1}2)$ twinning serves as one of its crucial deformation modes with a limited silp system at low temperatures[1]. During twinning deformation, the crystal structures on either side of the twin plane undergo mirror-symmetric shear, which adheres to specific crystallographic relationships[2]. With respect to $(10\bar{1}2)$ twinning, a rotation of approximately 86.3° about the $[11\bar{2}0]$ axis achieves orientation matching between the matrix and the twin. This derivation can be performed with orientation relationship formulas, as presented below.

$$\theta = \cos^{-1}\left(\frac{c^2 - 3a^2}{c^2 + 3a^2}\right) \quad (1)$$

High-resolution TEM observations indicate that the matrix orientation is (0001) under the $[11\bar{2}0]$ zone axis. After deformation, the c-axis tilts, resulting in a specific angle between the basal plane and the twin plane. The misorientation angle can be determined by calculating the transformation of Miller indices between the two grains.

Substituting a *c/a* ratio of 1.623 into the equation, the theoretical angle can be obtained. The obvious peak near 86.3° in the corresponding chart strongly agrees with the theoretical calculation, confirming that a significant proportion of the grain boundaries in the sample are $(10\bar{1}2)$ twin boundaries. This type of twin readily forms during the plastic deformation of Mg alloys and represents an essential mechanism for adjusting crystal orientation to accommodate strain.

Supplementary Note 2

To better illustrate the evolution of porosity with increasing deposition capacity, the thickness of Mg layers at different capacities is summarized in Supplementary Fig. S6. The results show that, irrespective of the applied capacity, Mg deposited on 70-twinned Mg consistently exhibits a much higher density (i.e., smaller thickness), which agrees with the CT observations and confirms that deposition on 70-twinned Mg is more uniform and dense.

In Supplementary Fig. 7, the black bars indicate the thickness of a fully dense Mg deposit. For reference, the thickness of Mg corresponding to a deposited capacity of 1 mAh cm^{-2} is approximately 2.6 μm, calculated using the following equation[3]:

$$Thickness = \frac{CM}{nF} * \frac{1}{\rho} \tag{2}$$

where C is the areal capacity (mAh cm^{-2}), M the molar mass (g mol^{-1}), n the number of electrons transferred, F the Faraday constant (C mol^{-1}), and ρ the density (g cm^{-3}). Thus, for a deposition capacity of 1 mAh cm^{-2}, the calculated thickness is about 2.6 μm.

Supplementary Note 3

The deposition morphology on a substrate is determined by the relative magnitudes of the electrochemical reaction rate (k_e) and the surface diffusion rate (k_d)[4, 5].

$$D_a = \frac{k_e}{k_d} \tag{3}$$

For Mg deposition on various substrates, these parameters are related to the experimental current density and the self-diffusion coefficient of Mg. The electrochemical reaction rate, k_e, can be derived from the experimental current density, J, as follows:

$$k_e = \frac{Ja^2}{F} N_A \tag{4}$$

Where a is the size of one lattice cell, F is Faraday's constant, and N_A is Avogadro's constant. The values of these parameters are listed in Supplementary Table 3**.** This paper introduces three primary current densities: 0.5, 1, 3, 5, and 10 mA cm^{-2}, respectively. Correspondingly, the k_e values are 7.80, 15.60, 46.81, 78.02 and 156.03 s^{-1}, respectively.

The surface diffusion rate, k_d, is calculated using Equation S4 and is dependent on the activation energy barrier for self-diffusion, E_{diff}, temperature,T, and jump frequency for Mg diffusion, v. The literature reports average values of jump frequency, which range between 10^{12}-10^{13} s^{-1}. Therefore, a value of $v = 5\times10^{12}$ s^{-1} has been utilized where k_B represents the Boltzmann constant.

$$k_d = v\, exp\left(\frac{-E_{diff}}{k_B T}\right) \tag{5}$$

The self-diffusion energy barrier, E_{diff}, determines the magnitude of the diffusion rate and therefore must be accurately measured to capture the underlying dynamics of the Mg system. In this study, density functional theory (DFT) calculations were employed to evaluate the diffusion barriers of Mg on different surfaces. The calculated barriers for diffusion traverse the twin, across the twin, Mg($10\bar{1}2$), Mg(0001), Mg($10\bar{1}1$), and Mg($10\bar{1}0$) surfaces are 0.43, 0.56,

0.77, 0.76, 0.75, and 0.85 eV, respectively. These values correspond to diffusion rates of 2.98 $\times 10^5$, 1.96 $\times 10^3$, 1.26 $\times 10^0$, 0.85 $\times 10^0$, 0.58 $\times 10^0$, and 2.62 $\times 10^{-2}$ s^{-1}, respectively.

Supplementary Note 4

Mg single crystals were grown using a Bridgman-type crystal growth furnace, followed by crystallographic orientation cutting to obtain four well-defined Mg single-crystal surfaces: (0002)-Mg, $(10\bar{1}1)$-Mg, $(10\bar{1}2)$-Mg, and $(10\bar{1}0)$-Mg. The experimental parameters are as follows: To prepare Mg single crystals with a diameter of 12 mm and a height of 30 mm, a dual-temperature zone heating method was used to establish a stable temperature gradient. The high-temperature zone was set at 780˚C, and the low-temperature zone was set at 600˚C, with the temperature gradient precisely controlled within 1˚C /cm. The crucible descent rate was adjusted to 10 mm/h, and Ar gas was used as a protective atmosphere throughout the experiment to prevent oxidation contamination of the Mg single crystals during preparation. These surfaces correspond exactly to the crystal planes used in our theoretical calculations of adsorption energy, surface diffusion barriers, and associated Da numbers.

We then systematically examined the Mg deposition morphologies on these four crystallographic surfaces under electrochemical conditions at a current density of 10 mA cm^{-2} and a capacity of 2 mAh cm^{-2}. The experimental results show that (0002)-Mg, $(10\bar{1}1)$-Mg, and $(10\bar{1}2)$-Mg surfaces predominantly exhibit island-type deposition morphologies, while the $(10\bar{1}0)$-Mg surface displays even more pronounced deposition inhomogeneity, accompanied by localized uneven features. Importantly, these observed morphologies are fully consistent with the Da-based theoretical calculations, where higher Da values (reactions dominate) favor localized nucleation and island growth, whereas lower Da values (diffusion predominate) correspond to more uniform deposition tendencies. This one-to-one correspondence directly validates the effectiveness of the Damköhler analysis, which links surface diffusion kinetics and reaction kinetics to deposition morphology under actual electrochemical conditions.

For the 70-twinned Mg electrode, the structure is more complex due to its heterogeneous surface nature. EBSD analysis confirmed the presence of multiple crystal orientations within

the matrix, along with approximately 70% of the internal interfaces are $(10\bar{1}2)$ typed tension twin. Notably, deposition on pristine single-crystal surfaces alone leads to island-like morphologies rather than planar growth, indicating that the observed uniform deposition on 70-twinned Mg cannot be explained solely by basal-plane exposure. Therefore, in our analysis, we adopt a justified approximation by identifying twin boundaries as the dominant structural feature governing deposition behavior. These twin boundaries reduces Mg diffusion barriers, as demonstrated by DFT, effectively accelerating surface Mg transport and lowering the Da number to values far below 1. As a result, the presence of a high density of twin boundaries shifts the effective deposition regime from diffusion-limited statement toward a state in which diffusion constraints are substantially alleviated, enabling more homogeneous nucleation and planar growth. In this sense, the Da number serves as the link between diffusion kinetics at the atomic level and microstructural deposition patterns, meaning that the deposition behavior observed at the electrode can be explained by calculating diffusion rates and reaction rates.

Supplementary Note 5

At the initial stage of metal deposition ($t < t_m$), a large number of active nucleation sites are rapidly consumed as metal ions preferentially deposit onto energetically favorable locations on the substrate surface. This rapid nucleation process leads to a sharp increase in localized ion concentration gradients. As deposition proceeds into the later stage ($t > t_m$), the diffusion zones surrounding adjacent nuclei begin to overlap, thereby reducing the effective diffusion pathways available for ion transport. Consequently, the supply of metal ions to each growing nucleus becomes limited, leading to a decrease in the overall deposition current. This transition reflects the dynamic evolution of interfacial processes during electrochemical deposition[6].

The relationship between the normalized current density $(j/j_m)^2$ and the normalized nucleation time (t/t_m) can be used to distinguish two nucleation modes: instantaneous and progressive.

$$3DI: \left(\frac{j}{j_m}\right)^2 = 1.9542\left(\frac{t}{t_m}\right)^{-1}\left\{1 - exp\left[-1.2564\left(\frac{t}{t_m}\right)\right]\right\}^2 \quad (6)$$

$$3DP: \left(\frac{j}{j_m}\right)^2 = 1.2254\left(\frac{t}{t_m}\right)^{-1}\left\{1 - exp\left[-2.3367\left(\frac{t}{t_m}\right)^2\right]\right\}^2 \quad (7)$$

Equation S6 corresponds to instantaneous nucleation, while Equation S7 represents progressive nucleation. In the case of instantaneous nucleation, nuclei form immediately, resulting in a rapid increase in their number followed by a subsequent decline. By contrast, progressive nucleation is characterised by a gradual increase in the number of nuclei, which continues to rise slowly until saturation is reached.

Notably, S–H model was derived based on idealised assumptions. However, in the Mg-on-Mg deposition systems, these assumptions are not not strictly fulfilled because the substrate inherently has non-idealized surface morphology. Therefore, it may cause a certain deviation between the experimental transient and the ideal S-H curve. To address this concern, we compared the Mg electrode with a rougher surface treatment (400# polished) and the 3000#

polished Mg electrode mentioned in the main text, and performed identical CA measurements. The results show that increased surface roughness indeed leads to a more pronounced deviation from the ideal S–H theoretical curves. However, this deviation does not alter the qualitative identification of nucleation modes: the pure Mg electrode consistently exhibits behaviour characteristic of three-dimensional instantaneous nucleation (3DI), whereas the twinned Mg electrode follows a three-dimensional progressive nucleation (3DP) trend. This indicates that while surface roughness affects the degree of agreement with the ideal model, it does not invalidate the comparative analysis of nucleation mechanisms in our study.

Supplementary Note 6

The Distribution of Relaxation Times (DRT) method has been widely employed for the accurate deconvolution and quantification of individual resistance contributions in electrochemical systems, thereby effectively avoiding artificial errors that often arise from equivalent circuit fitting. In essence, DRT is a mathematical transformation technique that converts impedance spectra from the frequency domain, represented by Nyquist plots, into the time domain to yield a DRT profile according to the following equation[7, 8].

$$Z_{DRT} = R_{\infty} + \int_{-\infty}^{\infty} \frac{\gamma(ln\tau)}{1 + i2\pi} dln\tau \quad (8)$$

In which, each electrochemical process with a characteristic relaxation time appears as a distinct local maximum in the continuous distribution function. The magnitude of the impedance associated with a specific process can be quantitatively evaluated from the integrated area under its corresponding peak, enabling a clear separation and identification of processes such as charge-transfer resistance or interfacial film resistance[9, 10].

Supplementary Table 1. A comparison of CE performance in recent demonstrations with our work.

	Electrolyte	Current density ($mA\ cm^{-2}$)	Areal capacity per cycle ($mAh\ cm^{-2}$)	Cycle number (n)	Cumulative capacity ($mAh\ cm^{-2}$)	Average CE value (%)	Ref.
Mg@BP	All phenyl complex (APC)	4	4	650	2600	99.66	11
Mg//Au-Cu	0.3 M $Mg(OTf)_2$+0.2 M $MgCl_2$ in DME	1	1	500	500	99.67	12
Mg//Au-Cu	0.3 M $Mg(OTf)_2$+0.2 M $MgCl_2$ in DME	5	2.5	120	300	99.43	12
Mg//Au-Cu	0.3 M $Mg(OTf)_2$+0.2 M $MgCl_2$ in DME	5	3.75	213	798.75	99.16	12
$Mo_2Ti_2C_3$	0.3 M $Mg(OTf)_2$+0.2 M $MgCl_2$ in DME	1	1	1400	1400	99.7	13
Mg||$Ti_3C_2T_x$	0.3 M $Mg(OTf)_2$+0.2 M $MgCl_2$ in DME	3	1	400	400	99.6	14
CF	All phenyl complex (APC)	5	5	350	1750	99.4	15
GC-NS	All phenyl complex (APC)	2	0.2	1000	200	99.9	16
Mg//Co@N/O-C	All phenyl complex (APC)	1	1	1175	1175	99.65	17
Mg//VNCA@C	All phenyl complex (APC)	4	4	100	400	99.1	18
This work	All phenyl complex (APC)	20	2	2515	5030	99.87	-
This work	All phenyl complex (APC)	30	2	2050	4100	99.53	-

Supplementary Table 2. Symmetric cells performance comparison between our works and recently reported Mg cells using different strategies.

	Electrolyte	Current density (mA cm^{-2})	Areal capacity per cycle (mAh cm^{-2})	Cycle time (h)	Cumulative capacity (Ah cm^{-2})	Ref.
PDG-Mg	0.3 M $Mg(OTf)_2$+0.2 M $MgCl_2$ in DME	1	1	900	0.9	[19]
(002)-Mg	All phenyl complex (APC)	5	5	300	1.5	[20]
PA-Al@Mg	All phenyl complex (APC)	9	1	300	2.7	[21]
PA-Al@Mg		5	1	800	4	[21]
Ge-based layer	0.5 M $Mg(TFSI)_2$/DME	10	5	350	3.5	[22]
Mg//VNCA@C	All phenyl complex (APC)	10	10	70	0.7	[18]
Mg	MLCC-1T1B	20	3	170	3.4	[23]
Mg	0.2 M $Mg(OTf)_2$ in DME + 5.4 vol% CP	20	3	130	2.6	[24]
Mg		25	1	220	5.5	[24]
Mg	$Mg(CB_{11}H_{12})_2$/DME-TG	20	3	83	1.66	[25]
**This work**	**All phenyl complex (APC)**	**5**	**5**	**1000**	**5**	**-**
**This work**		**10**	**5**	**800**	**8**	**-**
**This work**		**20**	**5**	**278**	**5.56**	**-**
**This work**		**25**	**1**	**360**	**9**	**-**

Supplementary Table 3. Model parameters.

Parameters		Values	Units
a	Lattice cell dimension	0.5	nm
J	Average current density	0.5,1,3,5,10	mA cm^{-2}
F	Faraday's constant	96487	C/mol
N_A	Avogadro's constant	6.022×10^{23}	L/mol
T	Operating temperature	300	K
v	Vibration frequency	5×10^{12}	s^{-1}
k_B	Boltzmann's constant	8.617×10^{-5}	eV K^{-1}

Supplementary Table 4. Summary of t_{max}*$(j_{max})^2$ values as requirement for the applicability of the S-H Method.

	70-twinned Mg			Pure Mg		
Potential (*vs.*Mg/Mg^{2+})	-0.8 V	-0.9 V	-1.0 V	-0.8 V	-0.9 V	-1.0 V
j_m ($mA*cm^{-2}$)	-30.13	-40.56	-57.49	-29.48	-31.37	-33.05
t_m (s)	10.01	5.33	2.56	1.81	1.47	1.10
$t_m*j_m^2$	$9.09*10^3$	$8.77*10^3$	$8.46*10^3$	$1.57*10^3$	$1.45*10^3$	$1.20*10^3$

Supplementary Table 5. Fitting parameters of EIS curves of different state samples in terms of fitting circuits. The values in parentheses are numerical error values.

		10th-Cycle	Rest-2h	10th-Rest-cycle
70 twinned Mg	R_S (Ω)	14.88$_{(4.3)}$	14.47$_{(2.75)}$	14.83$_{(3.29)}$
	R_{SEI} (Ω)	9.58$_{(1.88)}$	49.65$_{(5.28)}$	8.7$_{(2.27)}$
	R_{ct} (Ω)	39.75$_{(3.51)}$	171.4$_{(1.56)}$	37.42$_{(2.11)}$
Mg	R_S (Ω)	16.64$_{(0.34)}$	14.19$_{(0.59)}$	16.49$_{(0.4)}$
	R_{SEI} (Ω)	14.89$_{(2.15)}$	110.6$_{(2.64)}$	17.95$_{(2.08)}$
	R_{ct} (Ω)	43.92$_{(5.55)}$	754.1$_{(3.61)}$	107.3$_{(4.02)}$
		50th-cycle	Rest-2h	50th-Rest-cycle
70 twinned Mg	R_S (Ω)	14.8$_{(2.76)}$	16.59$_{(1.69)}$	14.99$_{(1.73)}$
	R_{SEI} (Ω)	8.33$_{(1.49)}$	25.88$_{(1.93)}$	8.3$_{(0.63)}$
	R_{ct} (Ω)	37$_{(0.36)}$	157.9$_{(5.12)}$	37.17$_{(0.45)}$
Mg	R_S (Ω)	16.74$_{(2.58)}$	15.22$_{(2.03)}$	16.75$_{(1.38)}$
	R_{SEI} (Ω)	17.12$_{(3.91)}$	103.9$_{(9.77)}$	19.53$_{(2.56)}$
	R_{ct} (Ω)	112.1$_{(2.17)}$	519.9$_{(13.9)}$	141.8$_{(2.45)}$
		100th-cycle	Rest-2h	100th-Rest-cycle
70 twinned Mg	R_S (Ω)	14.96$_{(1.56)}$	16.55$_{(2.03)}$	15.11$_{(3.17)}$
	R_{SEI} (Ω)	8.27$_{(2.03)}$	14.85$_{(1.55)}$	8.56$_{(1.43)}$
	R_{ct} (Ω)	37.66$_{(1.11)}$	144$_{(3.19)}$	37.63$_{(2.96)}$
Mg	R_S (Ω)	16.89$_{(1.22)}$	15.55$_{(1.67)}$	18.69$_{(0.32)}$
	R_{SEI} (Ω)	19.1$_{(2.29)}$	74.62$_{(2.06)}$	21.5$_{(3.95)}$
	R_{ct} (Ω)	145.4$_{(0.56)}$	430.7$_{(5.91)}$	163.7$_{(0.41)}$

Supplementary Table 6. Summary of Mo_6S_8-based full cells in recent research studies.

Full cell	Electrolyte	Mo_6S_8 mass loading (mg cm^{-2})	Voltage window (V)	Current density (mA g^{-1})	Specific capacity (mAh g^{-1})	Ref.
PA-based artificial SEI layers‖Mo_6S_8	All phenyl complex (APC)	1-2	0.1-1.7	644	64	26
SnSb‖Mo_6S_8	All phenyl complex (APC)	1	0.4-1.5	12.88	75	27
Mg@Au-Cu‖Mo_6S_8	0.3 M $Mg(OTf)_2$+0.2 M $MgCl_2$ in DME	1.91	0.2-2	100	32.3	12
Mg@VNCA@C‖Mo_6S_8	All phenyl complex (APC)	-	-	128.8	55.4	18
Mg-Cu‖Mo_6S_8	0.8 M $Mg(TFSI)_2$-2$MgCl_2$-DME	5	0.4-2.3	64.4	63.6	28
Sb-Mg/$MgCl_2$‖Mo_6S_8	0.3 M $Mg(OTf)_2$+0.2 M $MgCl_2$ in DME	1.55	0.2-2	128.8	47.9	29
MIEC-Mg‖Mo_6S_8	(0.25 M $Mg(TFSI)_2$/DME-0.5 M $MgCl_2$	1	0.2-1.6	257.6	55	30
Mg-Sn-Bi@Mg‖Mo_6S_8	All phenyl complex (APC)	-	0.2-1.95	128.8	62.3	31
Mg-Sn-Bi@Mg‖Mo_6S_8				1288	40.3	31
PDG-Mg‖Mo_6S_8	0.3 M $Mg(OTf)_2$+0.2 M $MgCl_2$ in DME	1.42	0.2-2.0	64.4	47.2	19
Mg@Co@N/O-C‖Mo_6S_8	All phenyl complex (APC)	1.13	0.4-2.0	386.4	58.8	17
				644	50	17
**This work**	**All phenyl complex (APC)**	**1.5**	**0.2-2**	**128.8**	**88.93**	-
				**644**	**74.28**	-
				**1288**	**71.84**	-

References

1. Su, H., et al. Atomic-scale insights into grain boundary-mediated plasticity mechanisms in a magnesium alloy subjected to cyclic deformation. *Acta Mater.* **277**, 120210 (2024).
2. Meng, L., Yang, P., Xie, Q.-G. & Mao, W. Analyses on compression twins in magnesium. *Mater. Trans.* **49**, 710-714 (2008).
3. Zou, G., et al. A nanotwinned-alloy strategy enables fast sodium deposition dynamics. *Nat. Commun.* **16**, 1795 (2025).
4. Hao, F., Verma, A. & Mukherjee, P. P. Mesoscale complexations in lithium electrodeposition. *ACS Appl. Mater. Inter.* **10**, 26320-26327 (2018).
5. Davidson, R., et al. Formation of magnesium dendrites during electrodeposition. *ACS Energy Lett.* **4**, 375-376 (2018).
6. Scharifker, B. & Hills, G. Theoretical and experimental studies of multiple nucleation. *Electrochim. Acta* **28**, 879-889 (1983).
7. Steinhauer, M., Risse, S., Wagner, N. & Friedrich, K. A. Investigation of the solid electrolyte interphase formation at graphite anodes in lithium-ion batteries with electrochemical impedance spectroscopy. *Electrochim. Acta* **228**, 652-658 (2017).
8. Single, F., Horstmann, B. & Latz, A. Theory of impedance spectroscopy for lithium batteries. *J. Phy. Chem. C* **123**, 27327-27343 (2019).
9. Yang Lu, et al. The carrier transition from Li atoms to Li vacancies in solid-state lithium alloy anodes. *Sci. Adv.* **7**, eabi5520 (2021).
10. Xu, L., et al. Revisiting the electrochemical impedance spectroscopy of porous electrodes in Li‐ion batteries by employing reference electrode. *Angew. Chem. Int. Ed.* **63**, e202406054 (2024).
11. Zhao, Q., et al. High-capacity, fast-charging and long-life magnesium/black phosphorous composite negative electrode for non-aqueous magnesium battery. *Nat. Commun.* **15**, 8680 (2024).
12. Li, Y., et al. High utilization of composite magnesium metal anodes enabled by a magnesiophilic coating. *Nano Lett.* **22**, 6808-6815 (2022).
13. Li, Y., et al. Double-transition-metal MXene films promoting deeply rechargeable magnesium metal batteries. *Small Methods* **7**, 2201598 (2023).
14. Li, Y., et al. MXene-based anode-free magnesium metal battery. *Adv. Funct. Mater.* **33**, 2303067 (2023).
15. Hao, Y., et al. An ultra-thin composite magnesium anode with controlled (002) facet

orientation by heterogeneous epitaxial electrodeposition for highly stable lean-magnesium metal batteries. *Acta Mater.* **293**, 121107 (2025).

16. Lim, H.-D., et al. Magnesiophilic graphitic carbon nanosubstrate for highly efficient and fast-rechargeable Mg metal batteries. *ACS Appl. Mater. Interfaces* **11**, 38754-38761 (2019).
17. Yang, J., et al. Ion recognition enables fast Mg–Cl bond dissociation kinetics and better Mg plating/stripping reversibility. *J. Magnes. Alloy* **12**, 5205-5215 (2024).
18. Song, Z., et al. Uniform magnesium electrodeposition via synergistic coupling of current homogenization, geometric confinement, and chemisorption effect. *Adv. Mater.* **33**, 2100224 (2021).
19. Li, Y., et al. Toward waterproof magnesium metal anodes by uncovering water-induced passivation and drawing water-tolerant interphases. *Nat. Commun.* **15**, 9364 (2024).
20. Bi, J., et al. Enhancing reversibility and stability of Mg metal anodes: high-exposure (002) facets and nanosheet arrays for superior Mg plating/stripping. *Angew. Chem. Int. Ed.* **63**, e202407770 (2024).
21. Wen, T., et al. Large-scale integration of the ion-reinforced phytic acid layer stabilizing magnesium metal anode. *ACS Nano* **18**, 11740-11752 (2024).
22. Zhang, J., et al. Rechargeable Mg metal batteries enabled by a protection layer formed in vivo. *Energy Storage Mater.* **26**, 408-413 (2020).
23. Xiao, J., et al. Cosolvent‐assisted formation of charged ion‐solvent clusters and solid electrolyte interphase for high‐performance magnesium metal batteries. *Adv. Energy Mater.* **12**, 2202602 (2022).
24. Yang, G., et al. Realizing horizontal magnesium platelet deposition and suppressed surface passivation for high-performance magnesium metal batteries. *Energy Environ. Sci.* **17**, 1141-1152 (2024).
25. Dong, H., et al. High-power Mg batteries enabled by heterogeneous enolization redox chemistry and weakly coordinating electrolytes. *Nat. Energy* **5**, 1043-1050 (2020).
26. Wen, T., et al. Rational design of artificial interphase buffer layer with 3D porous channel for uniform deposition in magnesium metal anodes. *Energy Storage Mater.* **55**, 816-825 (2023).
27. Cheng, Y., et al. Interface promoted reversible Mg insertion in nanostructured tin–antimony alloys. *Adv. Mater.* **27**, 6598-6605 (2015).
28. Sun, Y., et al. Non-nucleophilic electrolyte with non-fluorinated hybrid solvents for

long-life magnesium metal batteries. *Energy Environ. Sci.* **16**, 265-274 (2023).

29. Li, Y., et al. Grain-boundary-rich triphasic artificial hybrid interphase toward practical magnesium metal anodes. *Adv. Funct. Mater.* **33**, 2210639 (2022).

30. Wang, A., et al. Gradient perovskite ionic–electronic heterointerphases for deep cycling Mg metal anodes. *Adv. Funct. Mater.* **32**, (2022).

31. Chai, X., et al. Ternary Mg alloy-based artificial interphase enables high-performance rechargeable magnesium batteries. *Energy Storage Mater.* **70**, (2024).
