# Supplementary material for: Twinned-magnesium metal negative electrodes enable reversible magnesium electrodeposition
Source: Nat Commun. 2026 Sep 4;17:9599. doi: 10.1038/s41467-026-77543-8 (PMC13554073; doi:10.1038/s41467-026-77543-8)
Supplement: Supplementary file 2 [file 41467_2026_77543_MOESM2_ESM.pdf]

nature portfolio

# Peer Review File

## Twinned-magnesium metal negative electrodes enable reversible magnesium electrodeposition

Corresponding Author: Professor Qiuming Peng

Parts of this Peer Review File have been redacted as indicated to remove third-party material.

**This file contains all reviewer reports in order by version, followed by all author rebuttals in order by version.**

Version 0:

Reviewer comments:

Reviewer #1

(Remarks to the Author)
The article presents a novel strategy for fabricating magnesium metal anodes with a high density of twining through cyclic multidirectional deformation. The resulting twinned-Mg anode significantly enhances ion diffusion kinetics while effectively suppressing undesirable interfacial reactions. The electrochemical performance is exceptional, as demonstrated by an asymmetric coin cell employing the twinned-Mg anode in APC electrolyte, which achieves a Coulombic efficiency of 99.53% for over 2000 cycles at a high current density of 30 mA $cm^{-2}$ and a capacity of 2 mAh $cm^{-2}$. To the best of our knowledge, this represents the highest reported Coulombic efficiency for a Mg metal anode to date. Furthermore, the authors provide a thorough microstructural analysis, establishing a clear relationship between the twin-boundary defects and the improved electrochemical deposition behavior. The insights offered by this work present a significant advancement toward the realization of high-performance magnesium metal batteries. However, to meet the high standards of the journal, I suggest minor revisions to address the points detailed below prior to publication.

1. Twins are also frequently observed in the research of magnesium and its magnesium alloys as structural materials. They are an important means of strengthening and toughening magnesium and its alloys. At room temperature or lower temperatures, once twins form in magnesium alloys, their stability is relatively poor during subsequent deformation or service, and detwinning is prone to occur. So, how stable are magnesium twins during the electrochemical cycling process in your study?
2. Authors prepared Twinned magnesium anodes of different contents by cyclic-multiple-directional deformation route. How do the contents of different twins affect the electrochemical performance of Mg anodes?
3. How are the proportions of the three samples (Tiwn-40%, Tiwn-50% and 70 Tiwn-50%) determined? Please provide a detailed explanation. Additionally, are the twin crystal types of the three samples the same?
4. Please provide the fitting circuit diagrams for Figures 5e and 5f and carry out the fitting.
5. TOF-SIMS results show that no $MgO^+$/$MgCl^+$ is detected in the bulk phase, but it is still detected on the surface. Does this mean that the surface passivation layer will continue to grow and affect performance under longer cycle periods or higher capacities?
6. For the convenience of readers, please mark the size of the sample test area in the diagram in Top-SIMS in Fig 5d.
7. The authors conducted TEM to demonstrate that the 70-twined Mg sample had a thinner SEI after battery cycling. However, the image in Fig. 5h is too blurry. Please replace it.
8. In Fig. 6c, when comparing the charge and discharge curves of the full cells of the 70-twined Mg sample at different rates, voltage fluctuations occur above 1.6V in the charging curves at 0.5C and 1C rates. Why?
9. In the article, APC is used as the electrolyte. For the convenience of readers, please use the full name for the first time and specify what the specific components of APC are?

Reviewer #2

(Remarks to the Author)
This manuscript reports the use of deformation-induced twinning in magnesium metal to enhance deposition reversibility in APC-based electrolytes. The authors demonstrate improved cycling performance and a detailed structural analysis of the twinned Mg. While the topic is timely and the authors present substantial experimental data, I find that the current manuscript lacks the conceptual depth, mechanistic clarity, and generality required for publication in Nature Communications. The main

claims particularly the nearly bare-metal interface and the general applicability of the twin-engineering concept are not fully supported by the provided evidence.
Several major concerns limit the scientific impact and rigor of the study:
- The central argument that twin boundaries suppress interphase formation and promote a nearly bare-metal interface is insufficiently supported. Most interfacial analyses (XPS, ToF-SIMS) are ex situ and taken after only 10 cycles, which does not convincingly capture interphase evolution.
- The entire study is performed exclusively in APC ($AlCl_3$/PhMgCl), an electrolyte known to inherently form Mg–Cl complex layers and promote relatively smooth deposition. It is therefore unclear whether the observed improvements originate from the twin structure or from APC's intrinsic chemistry.
- Although the authors show reduced diffusion barriers along twin boundaries, the manuscript does not establish how this translates to suppressed MgO/$MgCl_2$ formation or reduced electrolyte decomposition. The mechanistic connection between crystallographic defects and chemical passivation remains speculative.
- Although DFT calculations illustrate reduced diffusion barriers, the correlation between theoretical predictions and observed morphology requires deeper analysis. For example, the manuscript does not clarify how the Damköhler number analysis corresponds to experimental nucleation behavior under realistic conditions.

Reviewer #3

(Remarks to the Author)
The submitted paper draft reports investigations about Twinned-magnesium metal anodes which show highly reversible electrodeposition, with, on the one hand, impressive performance, an interesting procedure for the anodes preparation and impressive claims overall. On the other hand, there are some fundamental flaws in the application of electrochemical models and methodologies which make the supporting investigations invalid, thus compromising the whole argument.

For this reason, this draft cannot be published in Nature Communications. Given the lack of scientifically solid and sound foundations, major revisions would not be enough to address its shortcomings.

Specifically:
- Electrochemical experiments are constantly ran in a 2-electrode setup; considering the very sensitive subject of multivalent battery chemistries, in which a clear definition of potential values is fundamental, that´s already a non-negligible flaw in the foundations of this study;
- One fundamental flaw is the misapplication and reporting of the Scharifker-Hills model on nucleation and growth. Once more, and more importantly here, a 3-electrode setup is of paramount importance for the clear definition of the potential values, and that is missing. The model in its application involves the sequential repetitions of step-wise chronoamperometric measurements within small intervals to check whether the model is applicable, through the calculation of a diagnostic parameter, which is the product (jmax)2*tmax which in principle should remain if not constant, in a very close range of values. this observation alone makes the application of the model in this draft invalid and conceptually wrong. The draft does not unambiguously states if a symmetric cell or an asymmetric cell is used. Only one potential is reported and more importantly the curve fittings, which don´t overlap completely with the theoretical curves (3DP and 3DI) in the most important region of the plot, i.e. before tmax, are just assigned to a general "mixed 3DP + 3DI" mechanism which, has a limited to none reference in literature; the statement anyway provides no reference or further explanation;
- The statement that 3DI nucleation enables "uncontrolled dendritic growth" has no literature reference and besides that, it is a bold and baseless statement since the model itself does not describe the later stages of growth, but only earlier initial times, which may or may have not any correlation with the final bulk deposits;
- Unfortunately this fundamentally wrong approach to the S-H model has been applied to another paper with similar structure (Damköhler number, S-H model, twinning strategy), published earlier this year in Nature Communications from almost all the same authors. Doi: https://doi.org/10.1038/s41467-025-56957-w

Reviewer #4

(Remarks to the Author)
This manuscript reports a deformation-induced twin-engineering strategy for magnesium metal anodes that enables highly reversible Mg electrodeposition under high current density and areal capacity. By introducing a high density of coherent $(10\bar{1}2)$ twins, the authors convincingly demonstrate enhanced Mg surface diffusion, suppressed parasitic interfacial reactions, and excellent long-term cycling stability. The work is original and mechanistically well grounded, relying on intrinsic crystallographic defect engineering rather than coatings, alloying, or electrolyte modification. The link between twin boundaries, reduced diffusion barriers, favorable Damköhler numbers, and uniform planar deposition is well supported by a combination of DFT calculations, electrochemical analysis, and multiscale structural characterization.
The experimental evidence is particularly strong, with complementary use of EBSD, TEM, in situ optical microscopy, and three-dimensional X-ray CT providing a coherent and convincing picture of deposition morphology and interfacial stability. The electrochemical performance, including high Coulombic efficiency over thousands of cycles at high current density and areal capacity, clearly exceeds the current state of the art for Mg metal anodes, and the full-cell validation further supports the practical relevance of the approach. Overall, the manuscript is well-suited for publication in Nature Communications.

The manuscript would be further strengthened by addressing the following minor points, which mainly concern clarification of interpretation and scope rather than additional experimental work:
1. The manuscript states that the twin structure shifts Mg deposition from diffusion-controlled to reaction-controlled. While the Damköhler analysis supports a relative shift, diffusion remains important in Mg electrodeposition. The wording could be softened to indicate that deposition becomes less diffusion-limited rather than strictly reaction-controlled.
2. Although long-term electrochemical stability is demonstrated, the structural stability of twin boundaries during extended cycling is not explicitly discussed. A brief comment on whether the twin density is expected to persist during cycling, or post-cycling structural evidence if available, would strengthen the conclusions.
3. All electrochemical data are obtained in APC-type halide electrolytes. A brief discussion on the expected applicability and possible limitations of the twin-engineering strategy in other Mg electrolyte systems would enhance its generality.
4. The extension of the strategy to Zn metal supports the generality of the concept, but the mechanistic analysis is less detailed than for Mg. It would be appropriate to explicitly frame the Zn results as a proof-of-concept validation rather than a fully equivalent mechanistic study.

Version 1:

Reviewer comments:

Reviewer #1

(Remarks to the Author)
The author have well addressed my comments. Therefore, I recommend the publication of this work in Nature Communications.

Reviewer #2

(Remarks to the Author)
I appreciate that the authors have made a substantial effort to address the comments. In particular, the revised manuscript now includes additional experimental characterization (e.g., XPS after extended cycling and in situ EIS monitoring) and theoretical calculations intended to support the proposed mechanism. These additions improve the manuscript and partially address the concerns raised in the previous review.
However, after carefully examining the revised version and the response to reviewers, I believe that several key issues remain insufficiently resolved. Therefore, I recommend major revision before the manuscript can be considered for publication.

-The evidence supporting the claim of a “nearly bare-metal interface” remains largely indirect. The authors should either provide more direct structural evidence for this claim or moderate the wording accordingly.
-The revised manuscript introduces DFT calculations describing the reduction of THF leading to MgO formation as a model reaction to explain electrolyte decomposition. However, the relevance of this reaction pathway to the APC electrolyte system is not sufficiently justified.
-In APC electrolytes, interfacial reactions are widely understood to be dominated by chloride-based chemistry involving Mg–Cl complexes and the formation of $MgCl_2$-containing interphases, rather than direct solvent decomposition to MgO. Therefore, the THF→MgO model reaction may not adequately represent the dominant decomposition pathway in this system.
-As a result, the presented thermodynamic analysis does not yet convincingly explain why twin boundaries would suppress interphase formation. A clearer mechanistic framework linking twin structures to electrolyte decomposition processes is still needed.

Reviewer #3

(Remarks to the Author)
The authors have made an effort to address the concerns raised in the initial review, and some improvements are acknowledged. However, several of the fundamental electrochemical and methodological issues identified in the original report remain unresolved, and in some cases the revised manuscript has introduced additional inconsistencies. I still can not recommend this manuscript for publication.

The points in question are the following:

- About point 1): the authors state that all chronoamperometric measurements have been re-performed in a three-electrode configuration, with pure Mg acting as quasi-reference electrode (QRE) and counter electrode (CE), and pure or twinned Mg as the working electrode (WE). The authors describe this configuration as an "asymmetric cell." In current electrochemical nomenclature, an asymmetric cell refers to a configuration in which WE and CE electrodes are composed of different materials. A configuration using Mg (normal or twinned) as WE, Mg as QRE, and Mg as CE is, by definition, a three-electrode symmetric cell. And this reflects a fundamental misunderstanding of electrochemical cell terminology. In addition, no cell diagram, is provided anywhere in the revised manuscript or Supporting Information. Cell configuration specifics (type

of cell, separator, electrode surface, electrolyte volume), geometry and QRE placement play a very important role in such experiments, especially taking into account multivalent chemistries, like in the case of magnesium.
- Cell setup in point 1), has also another non-negligible consequence: since the Scharifker-Hills model was derived under specific theoretical boundaries, assuming a flat featureless and electrochemically inert substrate as WE (e.g. glassy carbon of a flat metal substrate), this assumption is violated here, since in a symmetric cell it Mg-on-Mg deposition is assumed. The model's assumption of hemispherical diffusion zone expansion on a planar surface is not representative of these substrates, in which there can be neither chemical inertness nor surface flatness. Surface roughness/ irregularities of the Mg surface, can dramatically change/direct nucleation processes from the early stages(repetitions) of such experiments.
- The CA transients in Supplementary Fig. R28 and R31 (Rebuttal Letter), report the cathodic current density values,— which are negative by IUPAC convention — on the positive y-axis quadrant but as negative numbers, with the axis labelled simply as "Current density (mA $cm^{-2}$)." This is inconsistent and does not conform to standard electrochemical reporting practice. Plotting the absolute values as positive, with the axis explicitly labelled "$-j$ / mA $cm^{-2}$" would have been the correct way, and this signals an actually concerning way of handling data for such important experiments.
- Regarding point 2) he authors invoke double-layer charging as one of the explanations for early-time deviations from the theoretical S-H curves. This explanation is incorrect at the involved timescales of these nucleation experiments. Double-layer charging is complete within milliseconds of the potential step — orders of magnitude faster than the tmax reported values (1.81–10.01 s). At the timescale of tmax, capacitive contributions are negligible and are usually subtracted during data processing. Recalling a fast process to explain behavior at the seconds timescale is not valid and does not address the discrepancy identified above.
- In response to point 3) of the original review, the authors added COMSOL simulations to argue that 3DI-like nucleation, combined with limited surface diffusion, promotes morphological instability that ultimately evolves into dendritic structures. It is well established in literature — including in work cited by the authors themselves (Davidson et al., ACS Energy Lett. 2019) — that Mg dendrite formation is not a general consequence of electrodeposition. Dendritic growth in Mg is observed only under specific conditions of very high current density, where mass transport limitations become so severe to drive tip-enhanced growth instabilities. Under the current densities employed in this manuscript, Mg can be electroplated in irregular but non-dendritic morphologies — granular, mossy, or lumpy — which are distinct from classical dendrites. Indeed, the relative resistance of Mg to dendrite formation compared to lithium is cited as one of the material's advantages in the paper's own introduction. A single COMSOL simulation can not demonstrate how the system will evolve, specifically into dendrites under the current experimental conditions. Morphological inhomogeneity is not equivalent to dendritic growth. So the claim that 3DI-like nucleation characteristics „promote the evolution of dendritic structures" remains unsupported by the experimental conditions used and contradicted by the established behavior of Mg electrodeposition in the literature.
- The response fo the authors to point 4) does not address the substance of the comment. The concern raised was specifically methodological — that the same invalid approach to the S-H model and the same absence of rigorous potential control was used in a prior accepted high impact publication. Describing differences in battery chemistry or deformation strategy does not resolve whether the prior paper's nucleation analysis was valid. This remains an open concern.

Reviewer #4

(Remarks to the Author)
The new version of the manuscript describes a twin-engineering strategy for magnesium metal anodes that uses deformation to allow highly reversible Mg electrodeposition at high current density and areal capacity. The authors illustrate enhanced surface diffusion, inhibited parasitic reactions, and consistent planar deposition by introducing a high density of coherent twins.
The manuscript integrates electrochemical analysis, structural characterization, and DFT calculations to present a coherent mechanistic framework linking twin boundaries to enhanced Mg deposition behavior. The electrochemical performance and long-term cycling stability are especially impressive, demonstrating that Mg metal anodes have made a real step forward.
The authors have satisfactorily addressed the comments from the previous review and have clarified several points regarding the mechanistic interpretation and the scope of the work.
I think the manuscript is good enough to be published in Nature Communications.

Version 2:

Reviewer comments:

Reviewer #2

(Remarks to the Author)
The authors have adequately addressed all my previous comments. I recommend this manuscript for publication in its current form.

**Open Access** This Peer Review File is licensed under a Creative Commons Attribution 4.0 International License, which permits use, sharing, adaptation, distribution and reproduction in any medium or format, as long as you give appropriate credit to the original author(s) and the source, provide a link to the Creative Commons license, and indicate if changes were made.
In cases where reviewers are anonymous, credit should be given to 'Anonymous Referee' and the source.
The images or other third party material in this Peer Review File are included in the article's Creative Commons license, unless indicated otherwise in a credit line to the material. If material is not included in the article's Creative Commons license and your intended use is not permitted by statutory regulation or exceeds the permitted use, you will need to obtain permission directly from the copyright holder.
To view a copy of this license, visit https://creativecommons.org/licenses/by/4.0/

-----------------------------------------------------

**Reply to the report of referee #1**

----------------------------------------------------

**Comment of Referee #1:**

Reviewer 1

*The article presents a novel strategy for fabricating magnesium metal anodes with a high density of twining through cyclic multidirectional deformation. The resulting twinned-Mg anode significantly enhances ion diffusion kinetics while effectively suppressing undesirable interfacial reactions. The electrochemical performance is exceptional, as demonstrated by an asymmetric coin cell employing the twinned-Mg anode in APC electrolyte, which achieves a Coulombic efficiency of 99.53% for over 2000 cycles at a high current density of 30 mA cm*$^{-2}$ *and a capacity of 2 mAh cm*$^{-2}$*. To the best of our knowledge, this represents the highest reported Coulombic efficiency for a Mg metal anode to date. Furthermore, the authors provide a thorough microstructural analysis, establishing a clear relationship between the twin-boundary defects and the improved electrochemical deposition behavior. The insights offered by this work present a significant advancement toward the realization of high-performance magnesium metal batteries. However, to meet the high standards of the journal, I suggest minor revisions to address the points detailed below prior to publication.*

**Response:** We appreciate your positive and friendly comments. Our response follows each comment:

*1) Twins are also frequently observed in the research of magnesium and its magnesium alloys as structural materials. They are an important means of strengthening and toughening magnesium and its alloys. At room temperature or lower temperatures, once twins form in magnesium alloys, their stability is relatively poor during subsequent deformation or service, and detwinning is prone to occur. So, how stable are magnesium twins during the electrochemical cycling process in your study?*

**Response:** We sincerely thank you for this insightful and highly valuable comment. We fully agree that, in conventional structural applications of Mg alloys, deformation twins formed at room temperature or lower temperatures often exhibit limited stability and can undergo detwinning during subsequent mechanical deformation or service. This behaviour mainly arises because such twins are typically generated under relatively low strain and are not strongly constrained by surrounding microstructures important. However, in this study, the twins are generated through a cyclic multi-directional deformation process, which plays a crucial role in enhancing the structural stability of the twins. pecifically, repeated multidirectional strain introduces a high density of twins that are strongly constrained by each other and by the surrounding matrix. As a result, the twins in our electrodes are effectively “locked” within a mechanically constrained microstructure. Moreover, during electrochemical cycling, the Mg electrode is subjected primarily to electrochemical driving

forces rather than sustained mechanical shear stresses, which further suppresses the possibility of twin migration or detwinning.

To directly verify this stability, we performed TEM characterisation to evaluate the structural stability of the 70-twinned Mg electrode after electrochemical cycling. Specifically, after 50 cycles at a current density of 10 mA $cm^{-2}$ and a capacity of 5 mAh $cm^{-2}$, a high density of twin structure can still be clearly observed in the electrode (**Figure R1**). Corresponding SAED pattern analyses further confirm that these twins remain of the $\{10\bar{1}2\}$ type, indicating that no pronounced detwinning occurs during repeated electrochemical stripping/plating processes (**Figure R1b**). These observations provide direct experimental evidence that Mg twins can maintain good structural stability under electrochemical cycling conditions.

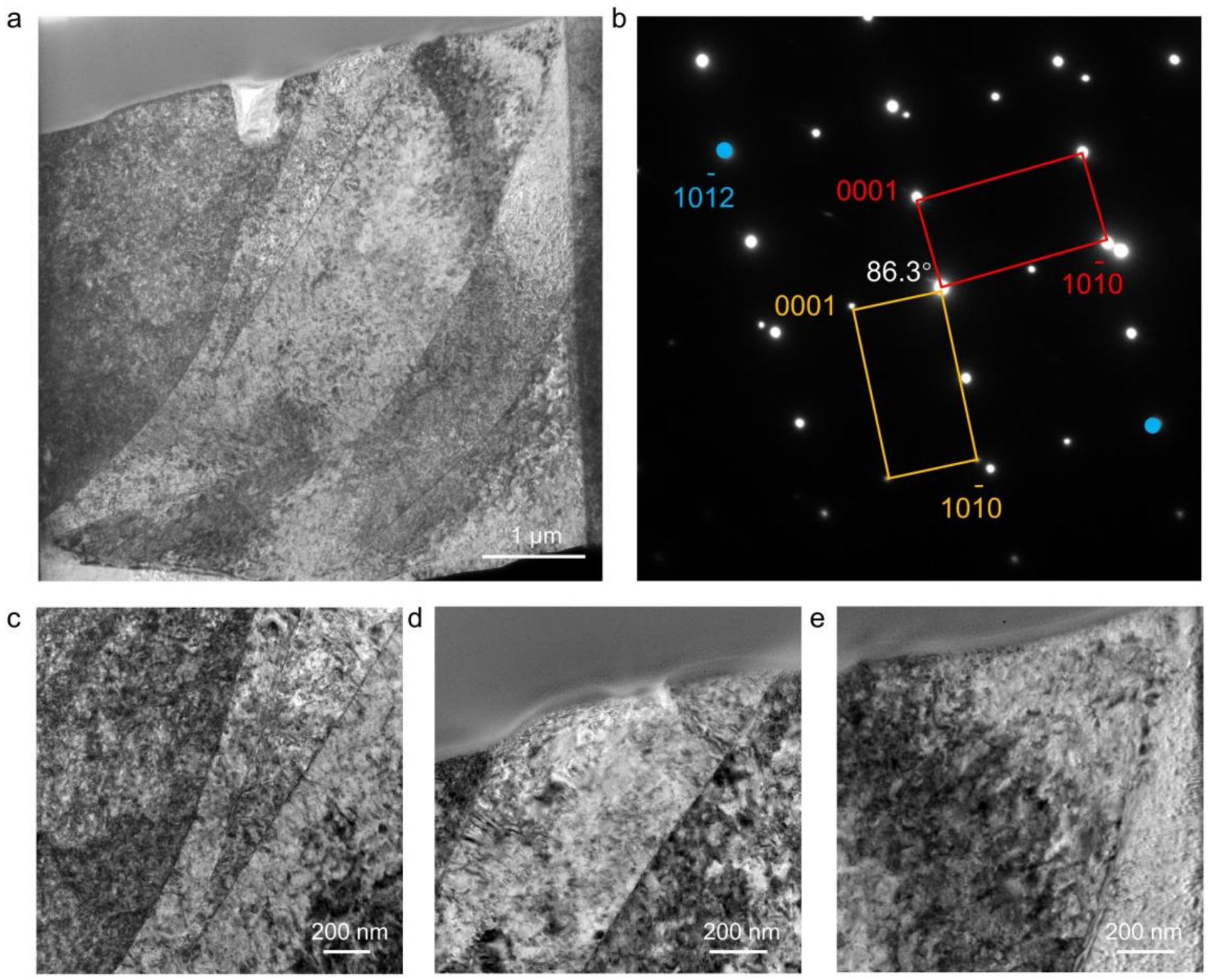


**Figure R1. a-e** TEM image (a) and corresponding SAED (b) of 70-twinned Mg after cycling (50 cycles, 10 mA $cm^{-2}$, 5 mAh $cm^{-2}$), as well as magnified detail images (c-e).

***We have made the following modifications to the manuscript:***

*In addition, the 70-twinned Mg electrode still retains a dense distribution of twinned structure after 50 cycles at a current density of 10 mA $cm^{-2}$ and a capacity of 5 mAh $cm^{-2}$ (**Supplementary Fig. S13**).* {Page 10, Line 1}

*2) Authors prepared Twinned magnesium anodes of different contents by cyclic-multiple-directional deformation route. How do the contents of different twins affect the electrochemical performance of Mg anodes?*

**Response:** Thank you for your valuable and constructive comments. Our study demonstrates that increasing the twin content effectively accelerates diffusion kinetics in Mg anodes while simultaneously suppressing parasitic side reactions during repeated charge–discharge processes. As a result, the electrochemical performance of Mg batteries is comprehensively and substantially enhanced (**Figure R2**).

During cyclic multidirectional deformation, twin formation in Mg evolves through a sequence of gradual activation, rapid multiplication, and, at excessive strain, detwinning or slip-dominated deformation. This structural evolution directly governs both deposition kinetics and interfacial stability. At relatively low twin contents (50-twinned Mg obtained after a single deformation cycle), twin defects can act as preferential adsorption sites for Mg deposition and partially reduce the nucleation energy barrier. However, these twin defects are spatially discrete and lack continuity. Consequently, they fail to establish an effective in-plane diffusion network, leading to constrained surface diffusion of Mg and a persistent mismatch between electrochemical reaction rates and diffusion kinetics. Under such conditions, Mg deposition preferentially proceeds via sustained growth at a limited number of active sites, resulting in non-uniform morphology, progressively increased interfacial resistance, and only modest performance improvements relative to pristine Mg (**Figure R2a, b**).

When the twin content increases to approximately 70% (70-twinned Mg after three cycles of multidirectional deformation), the system enters an optimal regime characterized by strong coupling between structural features and electrochemical kinetics. At this stage, a high density of $\{10\bar{1}2\}$ twins forms a continuous and well-distributed twin-boundary network within the grains and across the surface. These twin defects exhibit enhanced Mg affinity and enhanced binding energy, enabling more homogeneous Mg nucleation [*Nat. Commun. 2025, 16: 1795*]. Meanwhile, DFT calculations combined with electrochemical analyses demonstrate that twin defects substantially lower the surface diffusion barrier for Mg, thereby shifting the deposition process to less diffusion-limited behavior, as evidenced by a significantly reduced Damköhler number. As a result, Mg deposits in a dense and planar morphology. In addition, the high-density twin structure modulates the interfacial electronic structure, minimizing the mismatch between the reorganization energy and the operating overpotential. This effect effectively suppresses electrolyte decomposition and inhibits continuous passivation-layer growth. The combined benefits of accelerated diffusion kinetics and suppressed parasitic reactions underpin the exceptional cycling stability observed for the 70-twinned Mg anode (**Figure R2c**).

In contrast, when the deformation level is further increased (after five deformation cycles), the system no longer accumulates twins monotonically. Instead, sustained strain-energy accumulation induces detwinning and dislocation slip, reducing the effective twin fraction to 40% (40-twinned Mg). Although this structure still lowers the nucleation barrier to some extent, the twin distribution becomes increasingly discontinuous, and its influence on Mg diffusion and nucleation becomes spatially heterogeneous and unstable. As a consequence, continuous low-barrier diffusion pathways comparable to those in 70-twinned Mg cannot be maintained. Deposition kinetics therefore become unbalanced, interfacial side reactions are intensified, and the electrochemical performance deteriorates markedly relative to the optimally twinned sample (**Figure R2c, d**).

Overall, the dependence of Mg anode performance on twin content originates from its regulation of the coupled relationship among nucleation uniformity, surface diffusion kinetics, and interfacial reaction activity. Only when the twin density is sufficiently high and structurally stable can rapid Mg transport, uniform deposition, and a nearly passivation-free dynamic bare-metal interface be simultaneously achieved. This mechanism explains the distinctly superior cycling stability of the 70% twinned Mg anode compared with the other samples.

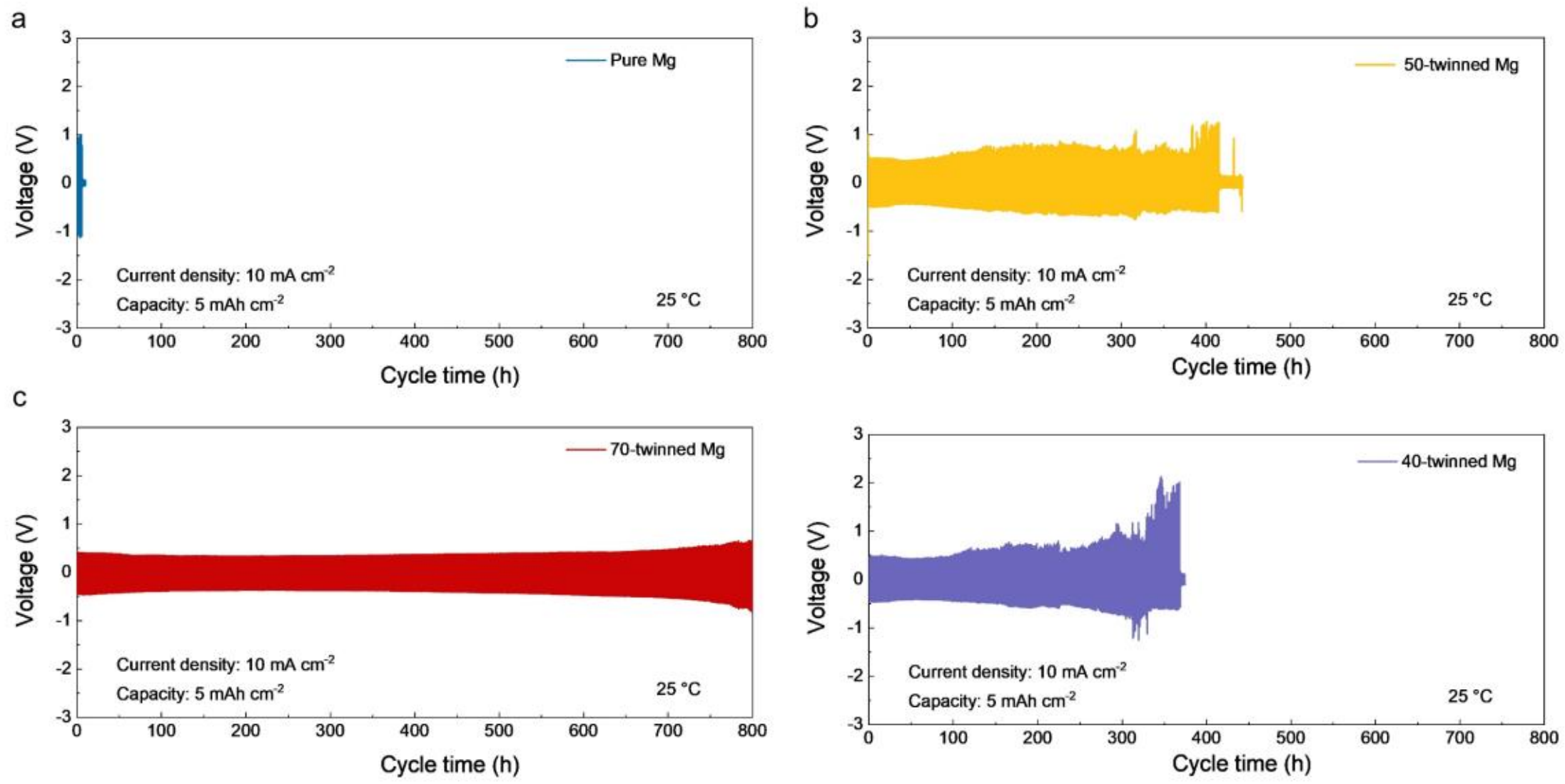


**Figure R2. a-d** Cyclic stability of the pure Mg|| pure Mg (a), 50-twinned Mg||50-twinned Mg (b), 70-twinned Mg||70-twinned Mg (c), and 40-twinned Mg||40-twinned Mg (d) symmetric cells at 10 mA $cm^{-2}$/5 mAh $cm^{-2}$.

*3) How are the proportions of the three samples (Tiwn-40%, Tiwn-50% and 70 Tiwn-50%) determined? Please provide a detailed explanation. Additionally, are the twin crystal types of the three samples the same?*

**Response:** Thank you for your insightful comment. The twin fractions of the three samples were determined through quantitative statistical analysis of electron backscatter diffraction

(EBSD) data. Specifically, systematic misorientation analyses were performed to identify characteristic misorientation peaks associated with $\{10\bar{1}2\}$ compression twins, which enables reliable discrimination between twin boundaries and conventional grain boundaries (**Figure R3, 4**). Based on this approach, the fraction of twin boundaries relative to the total interfacial length (including both grain boundaries and twin boundaries) was quantitatively evaluated. The statistical results reveal pronounced variations in twin-boundary fractions among samples subjected to different numbers of cyclic multidirectional deformation steps, thereby confirming the distinct twin contents and their corresponding proportions in the three samples.

From the standpoint of deformation mechanisms, after a single cycle of multidirectional compressive deformation, twin boundaries account for approximately 50% of the total interfacial length (defined as 50-twinned Mg) (**Figure R3a, R4**). This behavior is governed by the intrinsic crystallographic characteristics of hexagonal close-packed (HCP) Mg metal. At room temperature, non-basal dislocation slip systems in Mg require relatively high critical resolved shear stresses, whereas $\{10\bar{1}2\}$ compression twinning involves a lower geometric resistance. Consequently, twinning is preferentially activated and becomes the dominant plastic deformation mode [*Acta Mater. 2024, 277: 120210; Mater. Trans. 2008, 49, 710-714*]. As the number of cyclic multidirectional deformation steps increases to three, the applied strain continuously accumulates and is redistributed along multiple directions within the grains, facilitating sustained twin nucleation and propagation. This process results in the formation of a denser and more continuous twin-boundary network within the grains and near grain boundaries, increasing the twin-boundary fraction to approximately 70% (defined as 70-twinned Mg) (**Figure R3b, R4**). However, when the number of deformation cycles is further increased to five, the deformation mechanism undergoes a distinct transition. Excessive accumulation of plastic strain destabilizes the twin structure, and dislocation slip progressively replaces twinning as the dominant deformation mode, accompanied by pronounced detwinning behavior. During this stage, a portion of the previously formed twin boundaries is disrupted or annihilated by slip processes, leading to a reduction of the effective twin-boundary fraction to approximately 40% (defined as 40-twinned Mg) (**Figure R3c, R4**). Accordingly, the twin fractions of 40%, 50%, and 70% are not arbitrarily defined but instead emerge naturally from the competition among twin nucleation, twin proliferation, and detwinning at different stages of cyclic multidirectional deformation.

Regarding the twin types, EBSD analyses further demonstrate that all three samples contain the same twin variant, namely $\{10\bar{1}2\}$ tension twins (with the misorientation angles concentrated at ~86.3°) (**Figure R3**) [*J. Alloys Compd., 2017, 725: 1282-1287*]. This outcome reflects the inherent deformation preference of Mg under the applied conditions (room temperature and a multidirectional compressive stress state) under which $\{10\bar{1}2\}$ twinning exhibits the lowest activation barrier and the most favorable crystallographic

orientation relationship [*Acta Mater., 2010, 58: 5873-5885*]. As a result, this twin type predominates across samples with different twin fractions. It should be emphasized that the key distinction among the three samples lies in the twin content rather than in the twin type itself, which ensures a consistent structural basis and enables a meaningful comparison of how twin fraction influences the electrochemical behavior of Mg metal anodes.

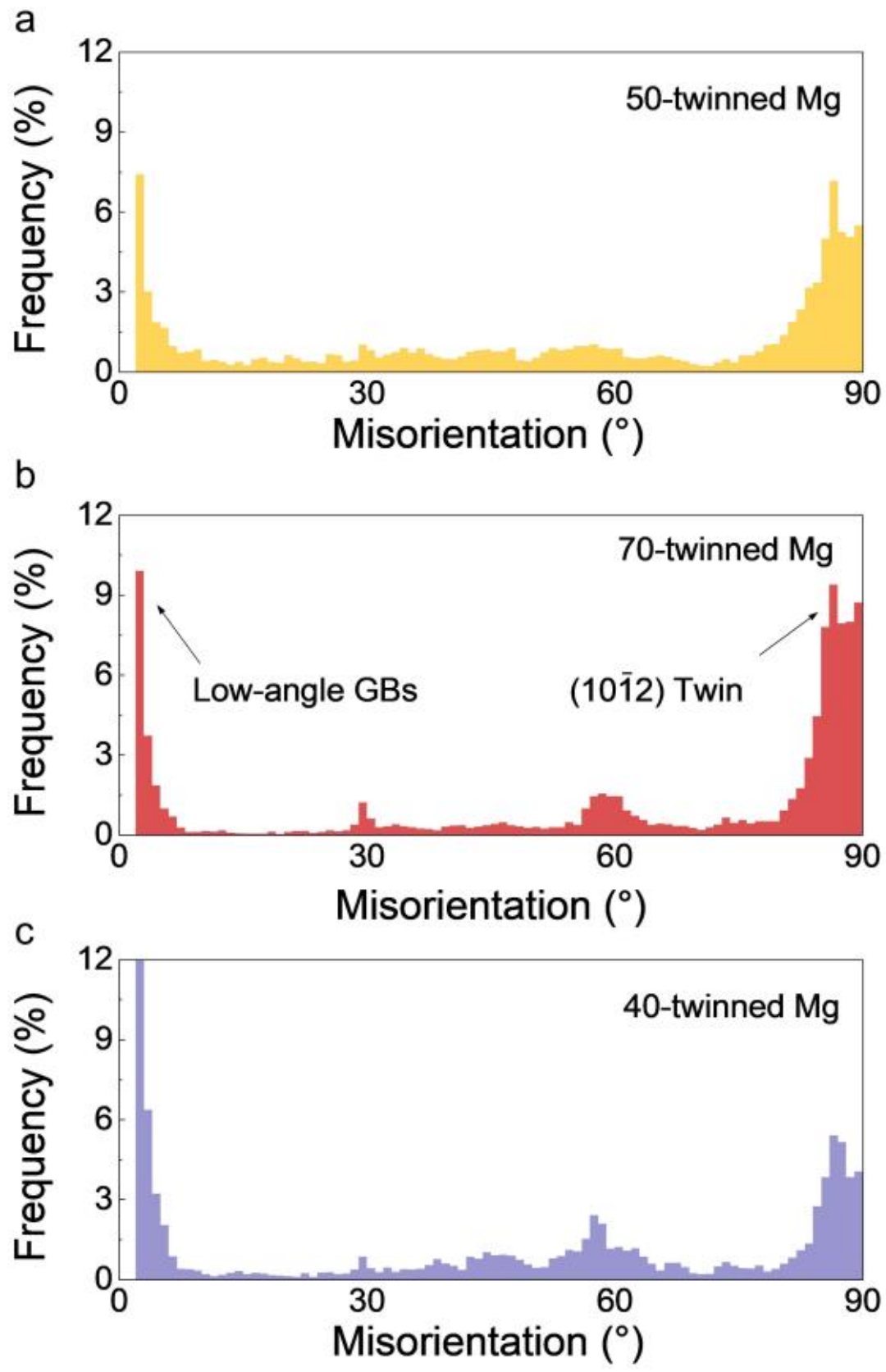


**Figure R3. a-c** Statistical analyses of the misorientation on the 50-twinned Mg (a), 70-twinned Mg (b),and 40-twinned Mg (c).

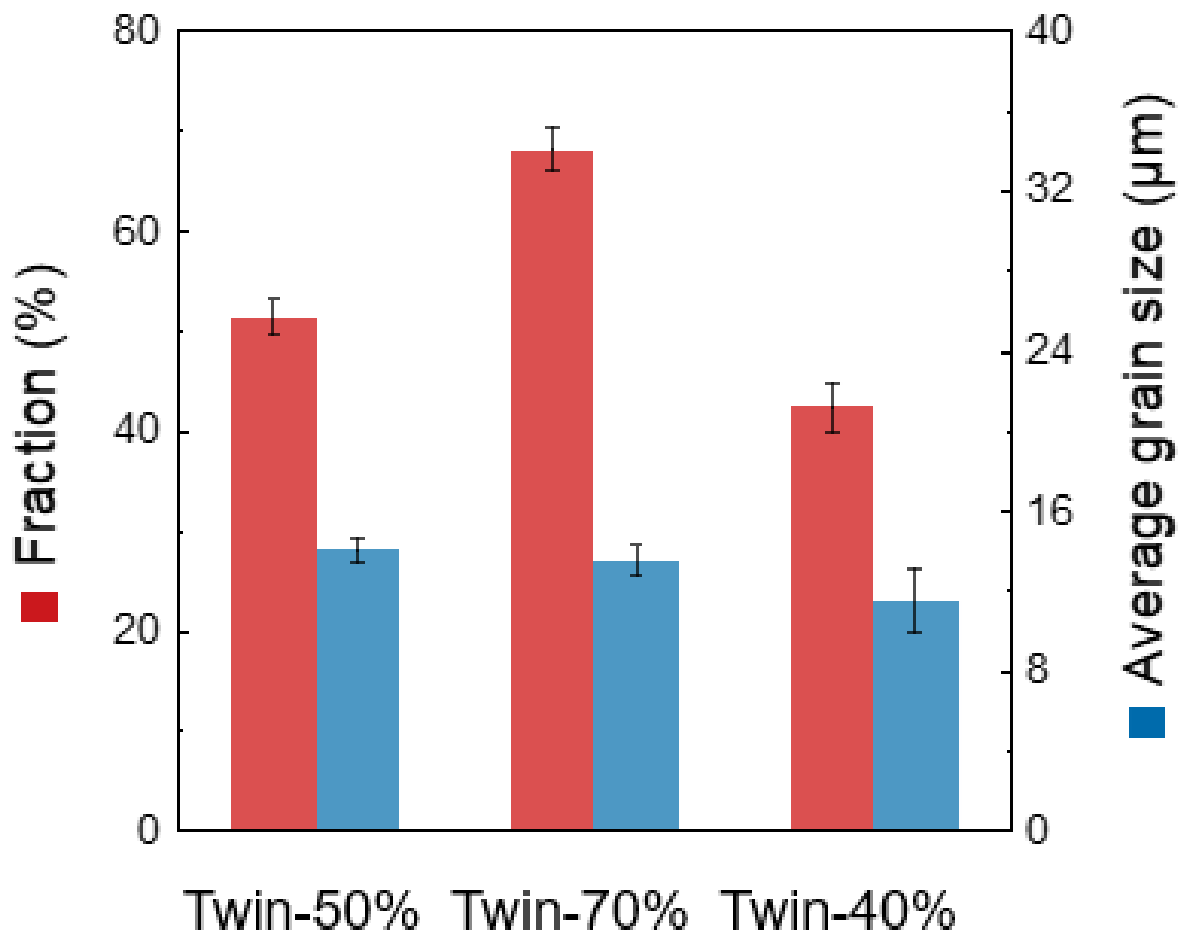


**Figure R4.** Fraction of twins and average grain size of different anodes.

***We have made the following modifications to the manuscript:***

Subsequently, according to quantitative EBSD analysis (**Fig. 1e**), twin boundaries account for approximately 70% of the total interface length (involving grain boundaries and twin boundaries) after three cycles of compression (defined as 70-twinned Mg), with the misorientation angles concentrated at ~86.3°. In contrast, the fractions of $(10\bar{1}2)$ twin boundaries after one or five cycles of compression were only 50% and 40%, respectively (defined as 50-twinned Mg and 40-twinned Mg, respectively). {Page 6, Line 10}

*4) Please provide the fitting circuit diagrams for Figures 5e and 5f and carry out the fitting.*

**Response:** Thank you for your constructive suggestion. We have supplemented the EIS data in Figures 5e and 5f with the corresponding equivalent circuit models and carried out systematic impedance fitting analyses (**Figure R5**). The detailed fitting parameters have been provided in the Supporting Information (**Table R1**).

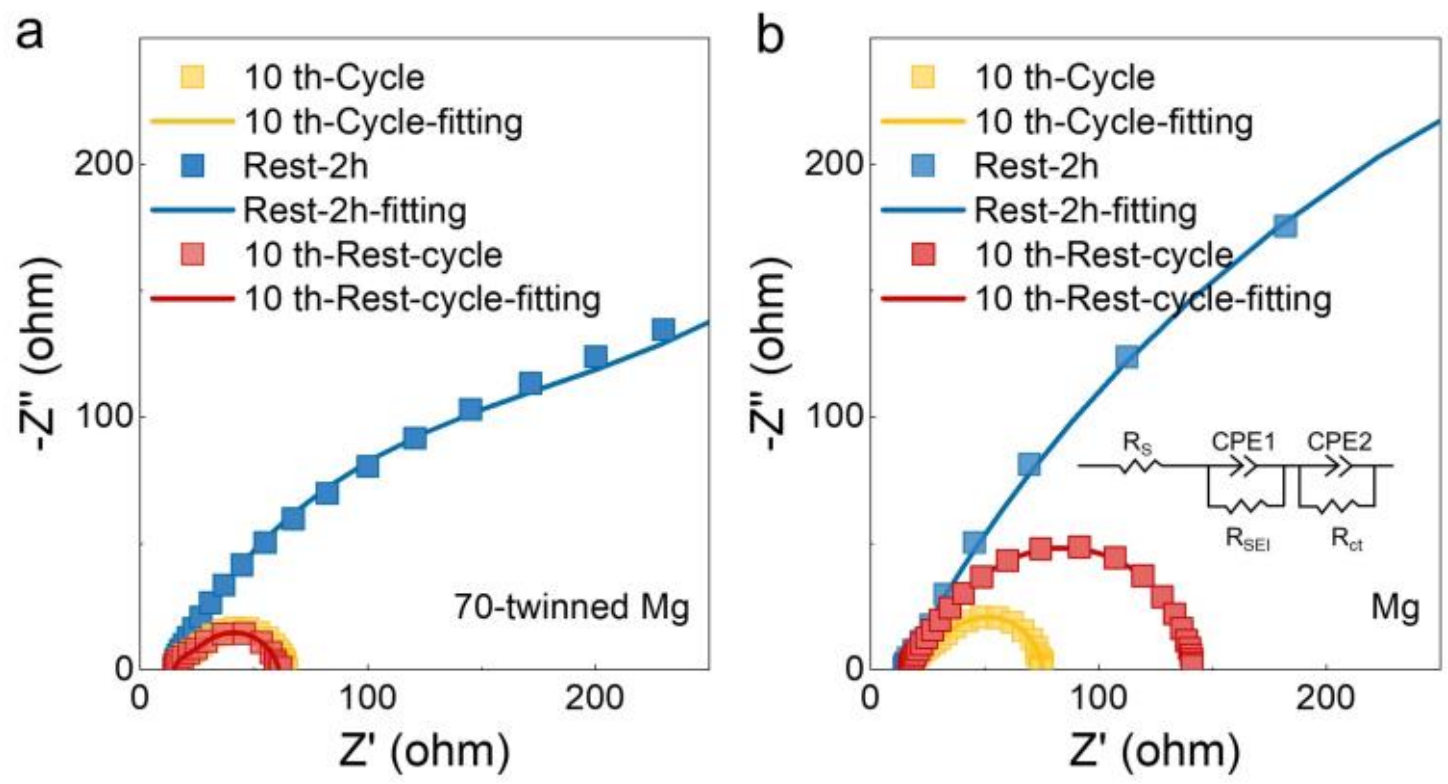


**Figure R5**. The EIS spectra of a 70-twinned Mg||70-twinned Mg and a Mg||Mg cell cell measured after cycling, resting for 2 h, and re-cycling period, under conditions of 10 mA cm$^{-2}$ and 1 mAh cm$^{-2}$. The inset corresponds to the fitted circuit.

**Table R1**. Fitting parameters of EIS curves of different state samples in terms of fitting circuits.

| | | 10th-Cycle | Rest-2h | 10th-Rest-cycle |
|---|---|---|---|---|
| 70 twinned Mg | $R_S$ | 14.88 | 14.47 | 14.83 |
| | $R_{SEI}$ | 9.58 | 49.65 | 8.7 |
| | $R_{ct}$ | 39.75 | 171.4 | 37.42 |
| Mg | $R_S$ | 16.64 | 14.19 | 16.49 |
| | $R_{SEI}$ | 14.89 | 110.6 | 17.95 |
| | $R_{ct}$ | 43.92 | 754.1 | 107.3 |

***We have made the following modifications to the manuscript:***

*Notably, upon reapplication of the electrodeposition/stripping current, the 70-twinned Mg electrode reverted to its initial state, indicating reversible interfacial behaviour (**Fig. 5e and Supplementary Table S5**), and maintained a stable interfacial impedance even after 100 cycles (**Supplementary Fig. S26**).* {Page 14, Line 24}

*However, the impedance of pure Mg symmetric cells remained significantly higher than the initial value after cycling, revealing sluggish charge-transfer processes during deposition and stripping (**Fig. 5f and Supplementary Table S5**), which further deteriorated after 100 cycles (**Supplementary Fig. S26**).* {Page 15, Line 6}

*5) TOF-SIMS results show that no $MgO^+$/$MgCl^+$ is detected in the bulk phase, but it is still detected on the surface. Does this mean that the surface passivation layer will continue to grow and affect performance under longer cycle periods or higher capacities?*

**Response:** Thank you for your constructive and insightful question regarding the possible evolution of the surface passivation layer during prolonged cycling or under higher areal capacities. To directly address this concern, we conducted additional post-cycling characterizations on the Mg anodes after extended cycling at a higher areal capacity (10 mA $cm^{-2}$, 5 mAh $cm^{-2}$), including XPS and in situ EIS after 50 and 100 cycles.

Specifically, the passivation products on the surface of the pure Mg electrode increased significantly after 50 cycles compared to after 10 cycles, indicating that the passivation products continued to accumulate on the surface (**Figure R6**). However, for the 70-twinned Mg anode, the interfacial chemical composition and the relative proportions of surface species remain essentially unchanged after 10, 50, and 100 cycles, indicating that the surface passivation layer does not undergo continuous growth during prolonged cycling (**Figure R7**).

Consistent with the ex situ XPS results, long-term in situ EIS measurements further demonstrate that the 70-twinned Mg anode maintains a stable interfacial impedance even after 100 cycles (**Figure R8**). Notably, the dynamic interfacial resistance during cycling show no significant increase; instead, a slight decrease in the static interfacial resistance after a 2 h rest is observed during cycling, which can be attributed to gradual interfacial activation. By contrast, the pure Mg electrode shows a pronounced and continuous increase in all impedance components during cycling, clearly indicating ongoing interfacial passivation (**Figure R8**).

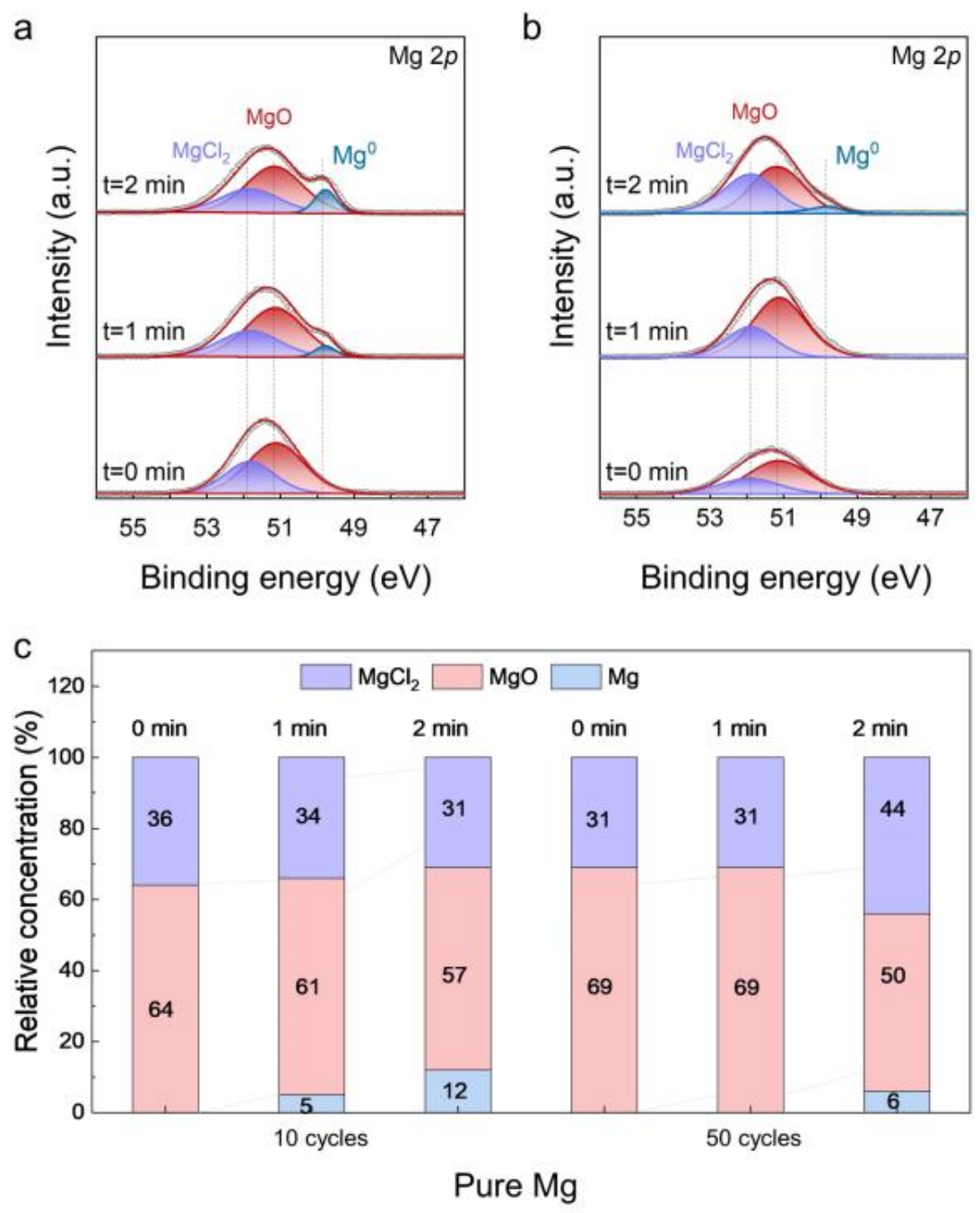


**Figure R6**. **a, b** High-resolution XPS spectra of Mg 2p of pure Mg after 10 cycling (a), 50 cycling (b) at 10 mA $cm^{-2}$, 5 mAh $cm^{-2}$. **c** The relative concentrations of decomposition species measured on the surface after cycling at sputtering depths of 0, 1 and 2 min.

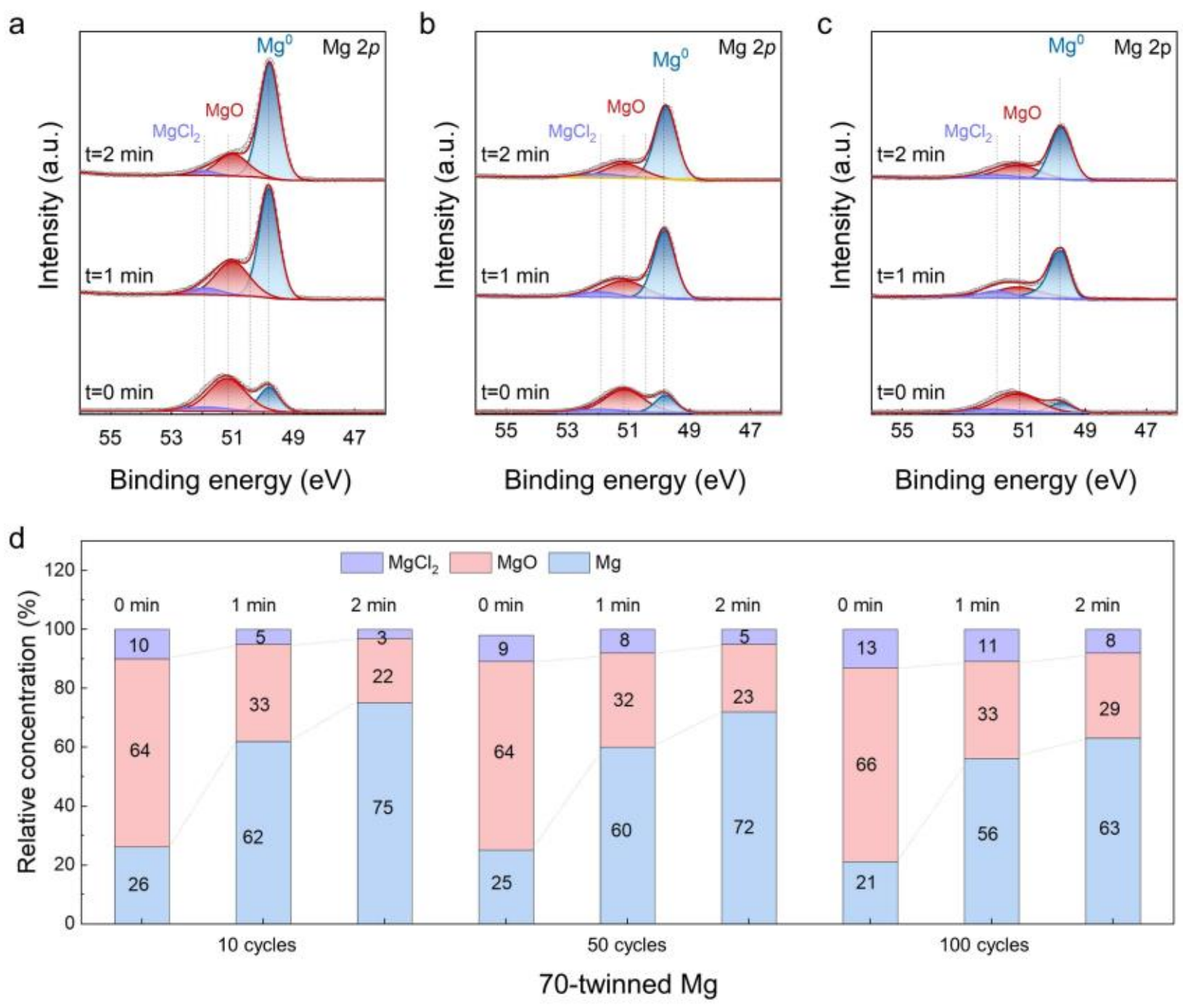


**Figure R7**. **a-c** High-resolution XPS spectra of Mg 2p of 70-twinned Mg after 10 cycling (a), 50 cycling (b), 100 cycling (c) at 10 mA $cm^{-2}$, 5 mAh $cm^{-2}$. **d** The relative concentrations of decomposition species measured on the surface after cycling at sputtering depths of 0, 1 and 2 min.

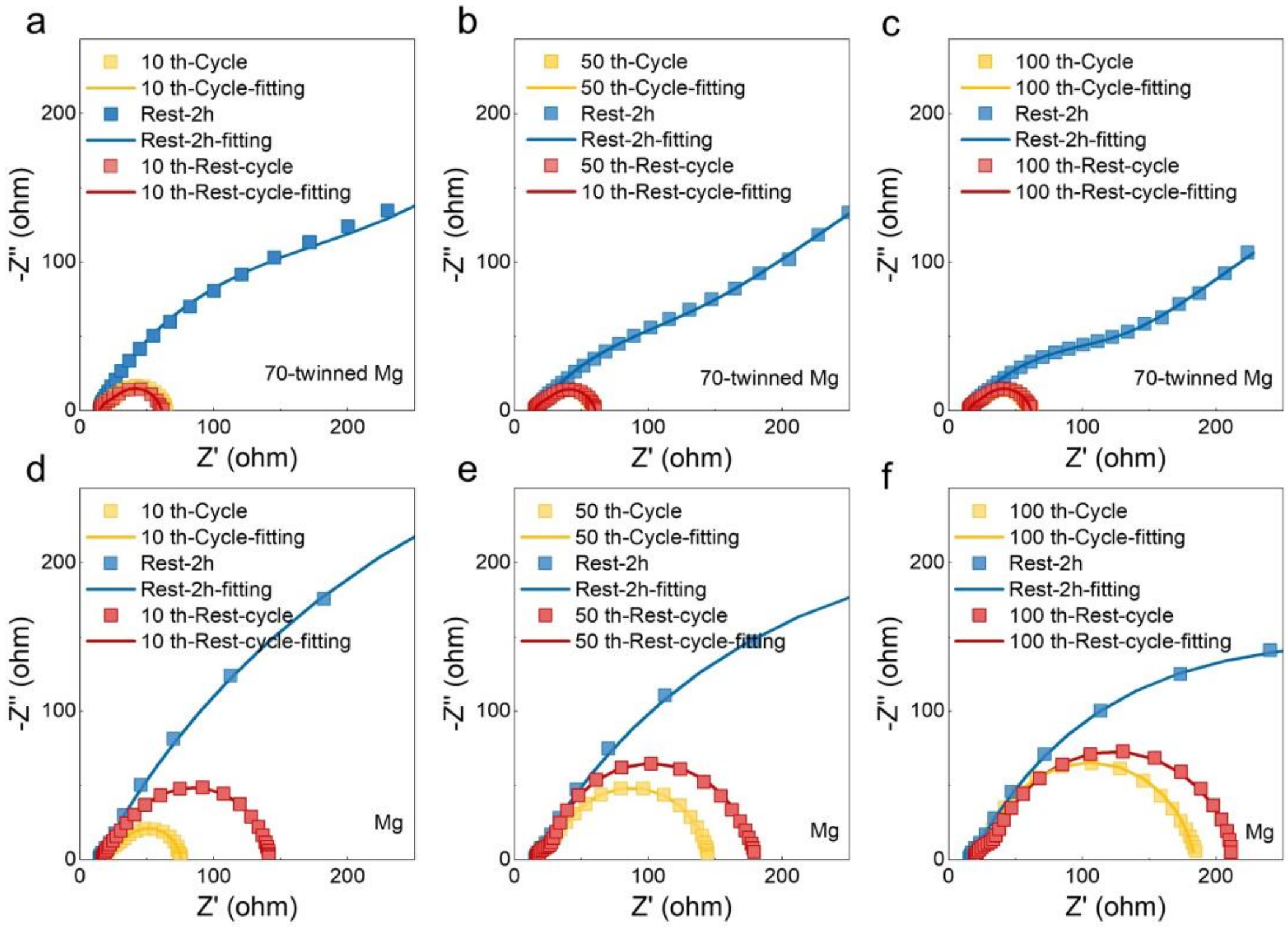


**Figure R8. a-f** The in situ EIS spectra of a 70-twinned Mg||70-twinned Mg (a-c) and a Mg||Mg cell (d-f) cell measured after 10 (a, d), 50 (b, e) and 100 (c, f) cycling, resting for 2 h, and re-cycling period, under conditions of 10 mA $cm^{-2}$ and 1 mAh $cm^{-2}$.

***We have made the following modifications to the manuscript:***

*Notably, the passivation products on the surface of the pure Mg electrode increased significantly after 50 cycles compared to after 10 cycles (**Supplementary Fig. S24**). However, for the 70-twinned Mg anode, the interfacial chemical composition and the relative proportions of surface species remain essentially unchanged after 10, 50, and 100 cycles (**Supplementary Fig. S25**).* {Page 14, Line 3}

*Notably, upon reapplication of the electrodeposition/stripping current, the 70-twinned Mg electrode reverted to its initial state, indicating reversible interfacial behaviour (**Fig. 5e and Supplementary Table S5**), and maintained a stable interfacial impedance even after 100 cycles (**Supplementary Fig. S26**).* {Page 14, Line 24}

*However, the impedance of pure Mg symmetric cells remained significantly higher than the initial value after cycling, revealing sluggish charge-transfer processes during deposition and stripping (**Fig. 5f and Supplementary Table S5**), which further deteriorated after 100 cycles (**Supplementary Fig. S26**).* {Page 15, Line 6}

*6) For the convenience of readers, please mark the size of the sample test area in the diagram in Top-SIMS in Fig 5d.*

**Response:** We thank the reviewer for this detailed and constructive suggestion. In the revised manuscript, explicit annotations have been added to the ToF-SIMS image in Figure 5d to clearly indicate the size of the analyzed area. Specifically, the ToF-SIMS measurements were conducted on a representative surface region with dimensions of 100×100 μm$^2$ **(Figure R9)**, ensuring a well-defined signal origin and consistent spatial resolution.

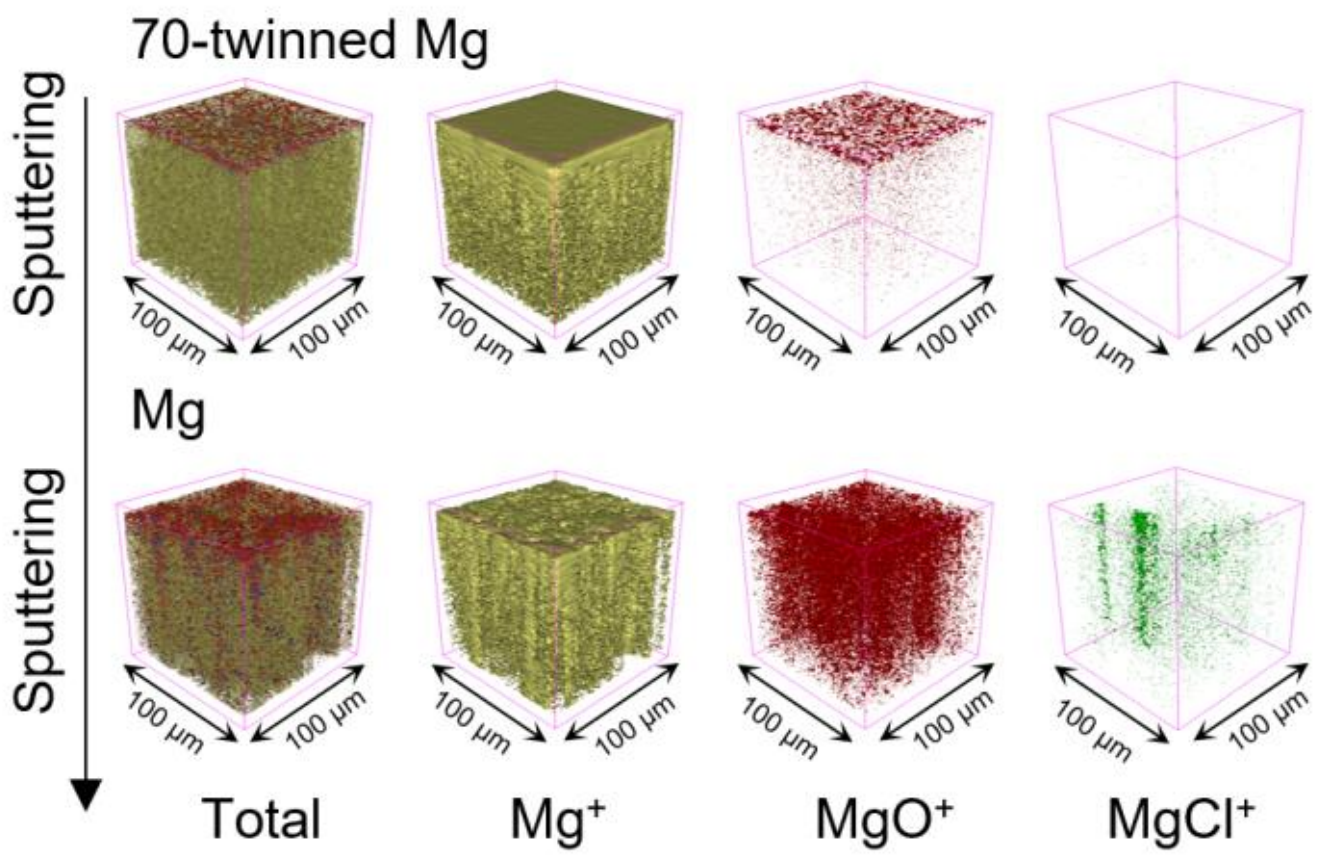


**Figure R9.** 3D rendering for the $Mg^+$, $MgO^+$, and $MgCl^+$ secondary ions and the overlay in the ToF-SIMS tested for the interfaces formed on the Mg and 70-twinned Mg electrodes.

*7) The authors conducted TEM to demonstrate that the 70-twined Mg sample had a thinner SEI after battery cycling. However, the image in Fig. 5h is too blurry. Please replace it.*

**Response:** We sincerely thank you for your careful comment. In response to the issue of insufficient TEM image sharpness, we have replaced Fig. 5h with the obtained TEM image (**Figure R10**). The revised image more clearly reveals the interfacial region of the 70-twinned Mg electrode after cycling, showing the presence of a thin interface layer.

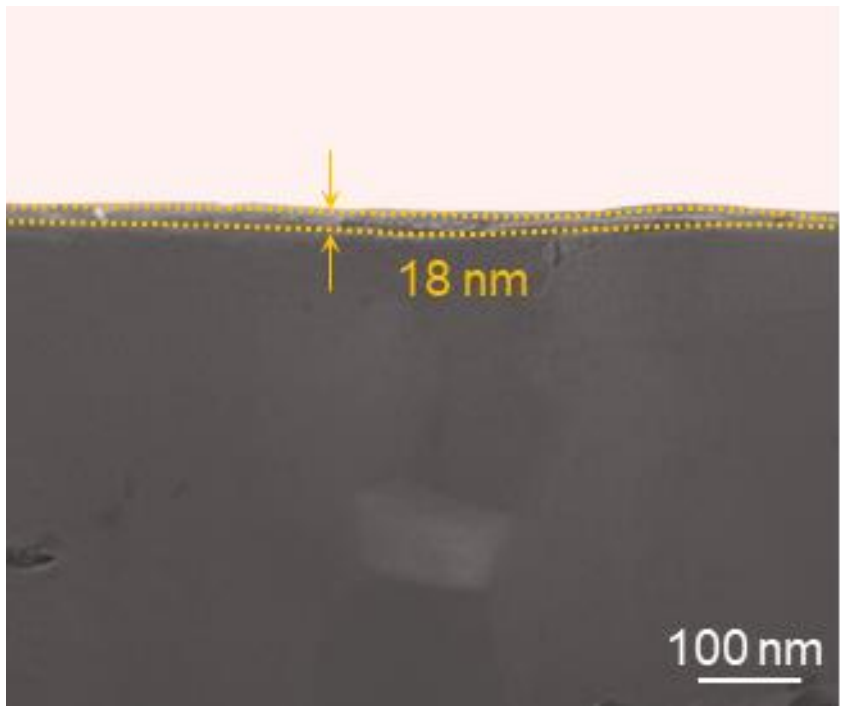


**Figure R10.** TEM images and of the interface and deoposited Mg after cycling for the 70-twinned Mg electrodes.

*8) In Fig. 6c, when comparing the charge and discharge curves of the full cells of the 70-twined Mg sample at different rates, voltage fluctuations occur above 1.6V in the charging curves at 0.5C and 1C rates. Why?*

**Response:** We thank the reviewer for their careful attention to the features observed in Fig. 6c. The voltage fluctuations occuring above 1.6 V in the charging curves at 0.5C and 1C are primarily attributed to transient polarization instabilities arising from kinetically limited $Mg^{2+}$ intercalation at the cathode under low-rate conditions. Specifiacally, at lower rates (0.5C and 1C), the charging process extends over a longer time period, during which $Mg^{2+}$ ions are gradually stripped from the Mg metal anode and subsequently migrate into the $Mo_6S_8$ cathode host lattice to complete the intercalation process. Compared with monovalent ions, $Mg^{2+}$ possesses a higher charge density and stronger solvation interactions, resulting in intrinsically sluggish desolvation and solid-state diffusion within the cathode material [*Chem. Mater. 2006, 18: 5492-5503*]. As the charging voltage exceeds 1.6 V, local $Mg^{2+}$ concentration gradients progressively accumulate in the cathode, leading to a transient mismatch between the intercalation kinetics and interfacial charge-transfer processes [*Solid State Ionics 2005, 176: 1695; Adv. Mater. 2022, 34: 2203783*]. This kinetic imbalance manifests as slight voltage fluctuations or subtle plateau undulations in the charging profiles. Similar behaviors have been widely reported in Mg full cell systems (**Figure R11**) [*Adv. Funct. Mater. 2022, 32: 2208735; Adv. Mater. 2022, 34: 2203783; ACS Energy Lett. 2024, 9: 2685-2695; Adv. Mater. 2020, 32, 1904987*].

**[Figure Redacted]**

**Figure R11**. Charge–discharge profiles. (a) Adv. Funct. Mater. 2022, 32: 2208735. (b) Adv. Mater. 2022, 34: 2203783. (c) ACS Energy Lett. 2024, 9: 2685-2695. (d) Adv. Mater. 2020, 32, 1904987.

*9) In the article, APC is used as the electrolyte. For the convenience of readers, please use the full name for the first time and specify what the specific components of APC are?*

**Response:** We thank the reviewer for this detailed and reader-friendly suggestion. We have provided the full definition of "APC" at its first occurrence in the manuscript and clearly

specified its composition. Specifically, APC refers to a fully phenyl-coordinated Mg electrolyte composed of 0.4 M PhMgCl in tetrahydrofuran (THF) with 0.25M $AlCl_3$.

***We have made the following modifications to the manuscript:***

*Mg was employed for both the working and counter electrodes, with the APC electrolyte (0.4 M PhMgCl in tetrahydrofuran (THF) with 0.25 M $AlCl_3$; Suzhou DoDoChem. Co., Ltd., China) serving as the medium.* {Page 21, Line 5}

---------------------------------------------------

**Reply to the report of referee #2**

---------------------------------------------------

**Comment of Referee #2:**

Reviewer 2

*This manuscript reports the use of deformation-induced twinning in magnesium metal to enhance deposition reversibility in APC-based electrolytes. The authors demonstrate improved cycling performance and a detailed structural analysis of the twinned Mg. While the topic is timely and the authors present substantial experimental data, I find that the current manuscript lacks the conceptual depth, mechanistic clarity, and generality required for publication in Nature Communications. The main claims particularly the nearly bare-metal interface and the general applicability of the twin-engineering concept are not fully supported by the provided evidence.*

*Several major concerns limit the scientific impact and rigor of the study:*

**Response:** We appreciate your meaningful and helpful comments. Our response follows each comment below.

*1) The central argument that twin boundaries suppress interphase formation and promote a nearly bare-metal interface is insufficiently supported. Most interfacial analyses (XPS, ToF-SIMS) are ex situ and taken after only 10 cycles, which does not convincingly capture interphase evolution.*

**Response:** Thank you for your meaningful and constructive comment. Regarding your concern about the sufficiency of evidence for twin boundaries suppress interphase formation and promote a nearly bare-metal interface, we have supplemented and strengthened our manuscript from two aspects: theoretical mechanism analysis and long-term interface evolution experimental verification.

Specifically, at the theoretical mechanism level, we have supplemented our work with DFT calculations to demonstrate the crucial roles of electrode potential and twin structure in interfacial evolution [*Angew. Chem. 2022, 134: e202208743; J. Power Sources 2012, 200: 83-91*]. Considering that tetrahydrofuran (THF) is the solvent in the APC electrolyte, we used the reaction process of THF reduction to MgO as a model reaction and systematically compared the reaction thermodynamic behavior of pure Mg and twinned Mg under different electrode potentials (**Figure R12 and R13**). At the same electrode potential, the Gibbs free energy for THF reduction on twinned Mg is significantly higher than that on pure Mg ($\Delta G_{twinned\text{-}Mg} > \Delta G_{pure\text{-}Mg}$), indicating that the twin structure intrinsically raises the energy barrier for solvent decomposition and passivation product formation. In addition, for the same anode material, increasing the electrode potential markedly increases the Gibbs free energy of the reduction reaction, thereby suppressing interfacial side reactions. Notably, this

“potential-interfacial reaction synergistic suppression effect” on twinned Mg provides clear thermodynamic evidence that twin structures favour the formation of a nearly bare-metal interface.

Secondly, in response to the comment that existing interfacial characterisations are mostly ex situ and limited to a small number of cycles, we further incorporated in situ characterisation after extended cycling and long-term in situ interfacial kinetic monitoring. Specifically, we performed systematic XPS analysis on the interface between pure Mg and 70-twinned Mg anodes after 10, 50, and 100 cycles. The results show that, for pure Mg electrodes, passivation-related species such as MgO and $MgCl_2$ increase significantly after 50 cycles compared to 10 cycles and continue to accumulate during subsequent cycling, clearly indicating continuous growth of the passivation layer (**Figure R14**). In contrast, for the 70-twinned Mg electrode, the interfacial chemical composition and the relative proportions of surface species remain essentially unchanged after 10, 50, and 100 cycles, with no evidence of continuous passivation layer thickening (**Figure R15**), suggesting that the interface maintains a dynamically stable state during long-term cycling.

Consistent with the above observations, we further performed long-term in situ EIS measurements to dynamically monitor the evolution of interfacial resistance (**Figure R16**). The results show that the interfacial impedance of the 70-twinned Mg anode remained stable overall after 100 cycles. In contrast, for pure Mg electrodes, the resistance increase continuously and significantly during cycling, clearly indicating ongoing interfacial passivation.

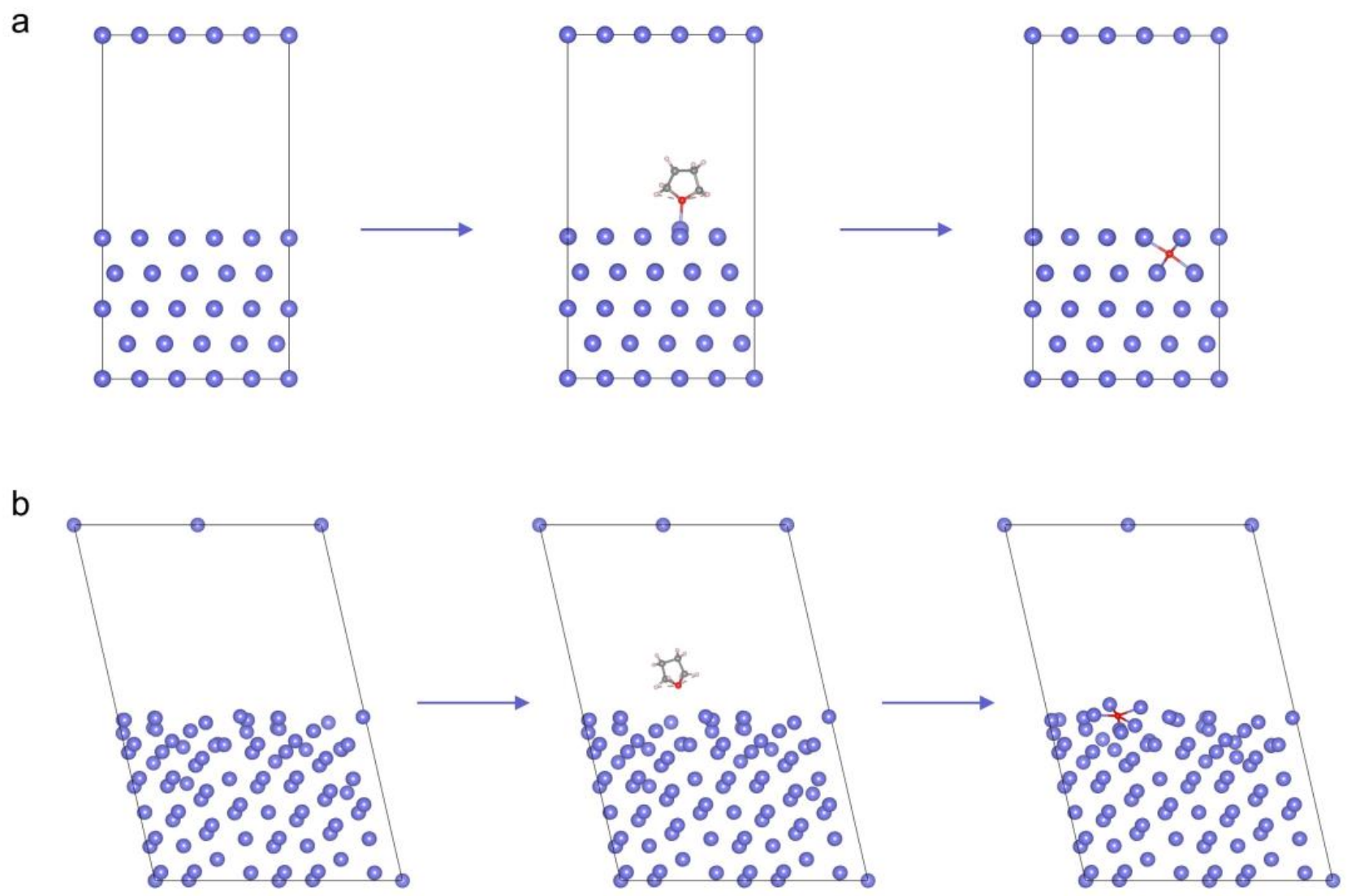


**Figure R12. a, b** Configurational changes before and after adsorption of THF molecules on pure Mg (a) and 70-twinned Mg (b) surfaces. (red, oxygen; grey, carbon; white, hydrogen; and purple, Mg).

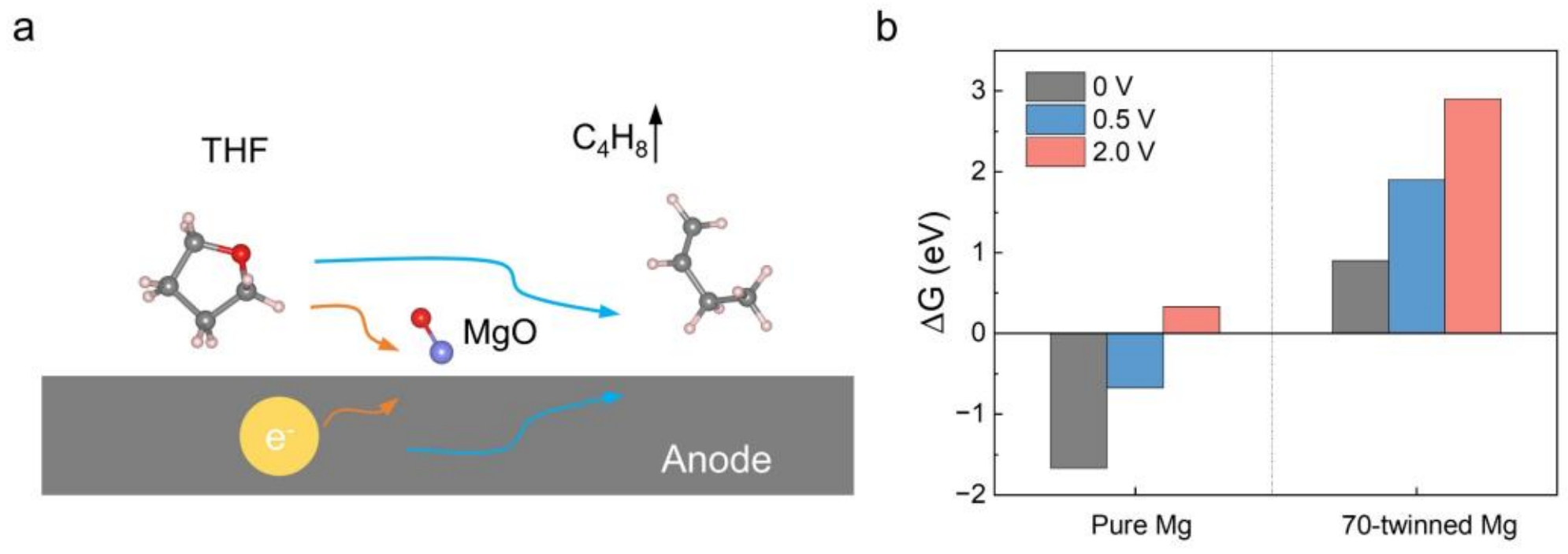


**Figure R13.** Theoretical calculation of the Gibbs free energy change of the reaction forming $C_4H_8$ or MgO on different anodes. a) Scheme of the calculation model (red, oxygen; grey, carbon; white, hydrogen; and purple, Mg); b) the free energy calculation results. (The reason for choosing potentials of 0.5 V and 2.0 V is that the overpotential of a 70-twinned Mg symmetric cell at 10 mA $cm^{-2}$ is 0.43V (~0.5 V); while the overpotential of a pure Mg symmetric cell at 10 mA $cm^{-2}$ is 2.26V (~2.0 V)).

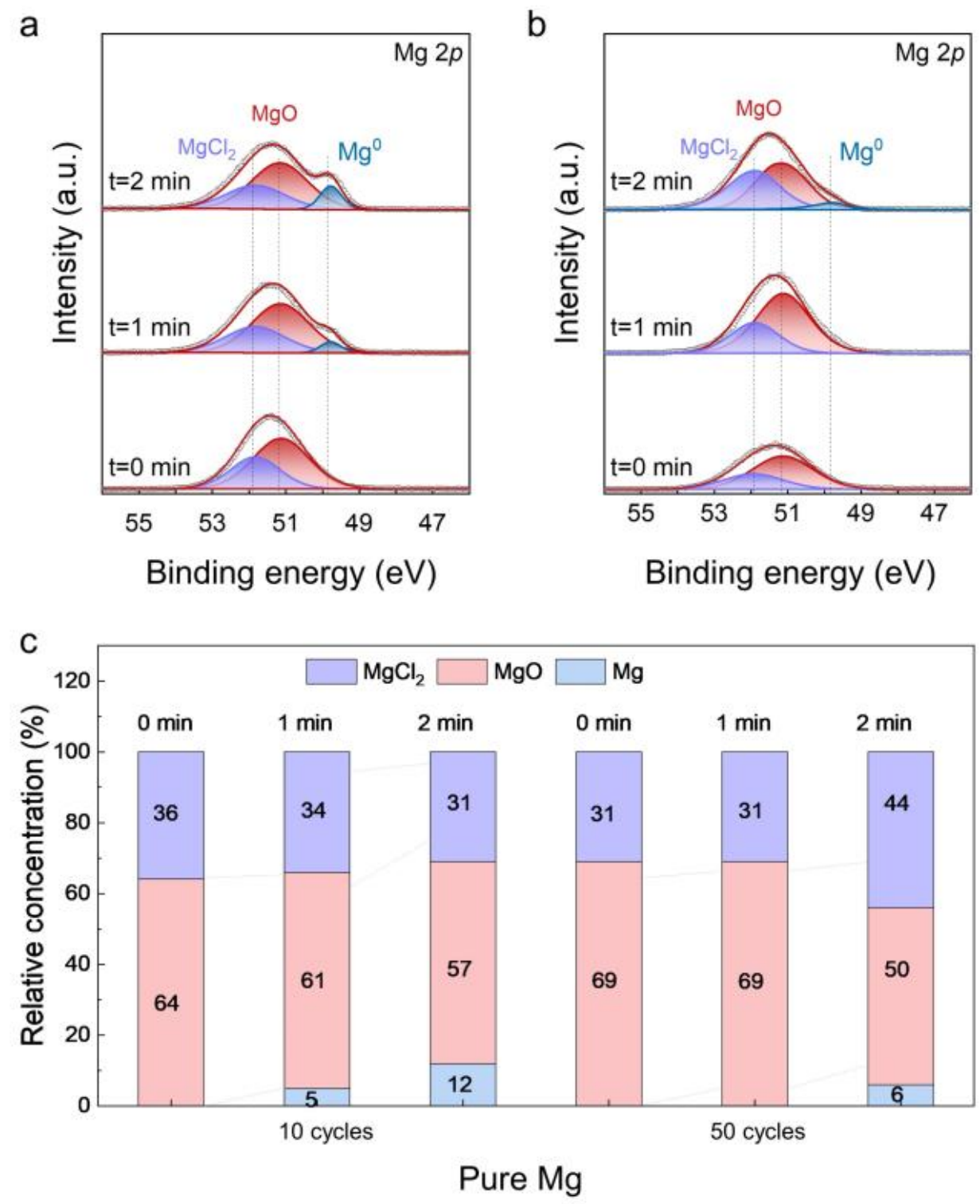


**Figure R14**. **a, b** High-resolution XPS spectra of Mg 2*p* of pure Mg after 10 cycling (a), 50 cycling (b) at 10 mA $cm^{-2}$, 5 mAh $cm^{-2}$. **c** The relative concentrations of decomposition species measured on the surface after cycling at sputtering depths of 0, 1 and 2 min.

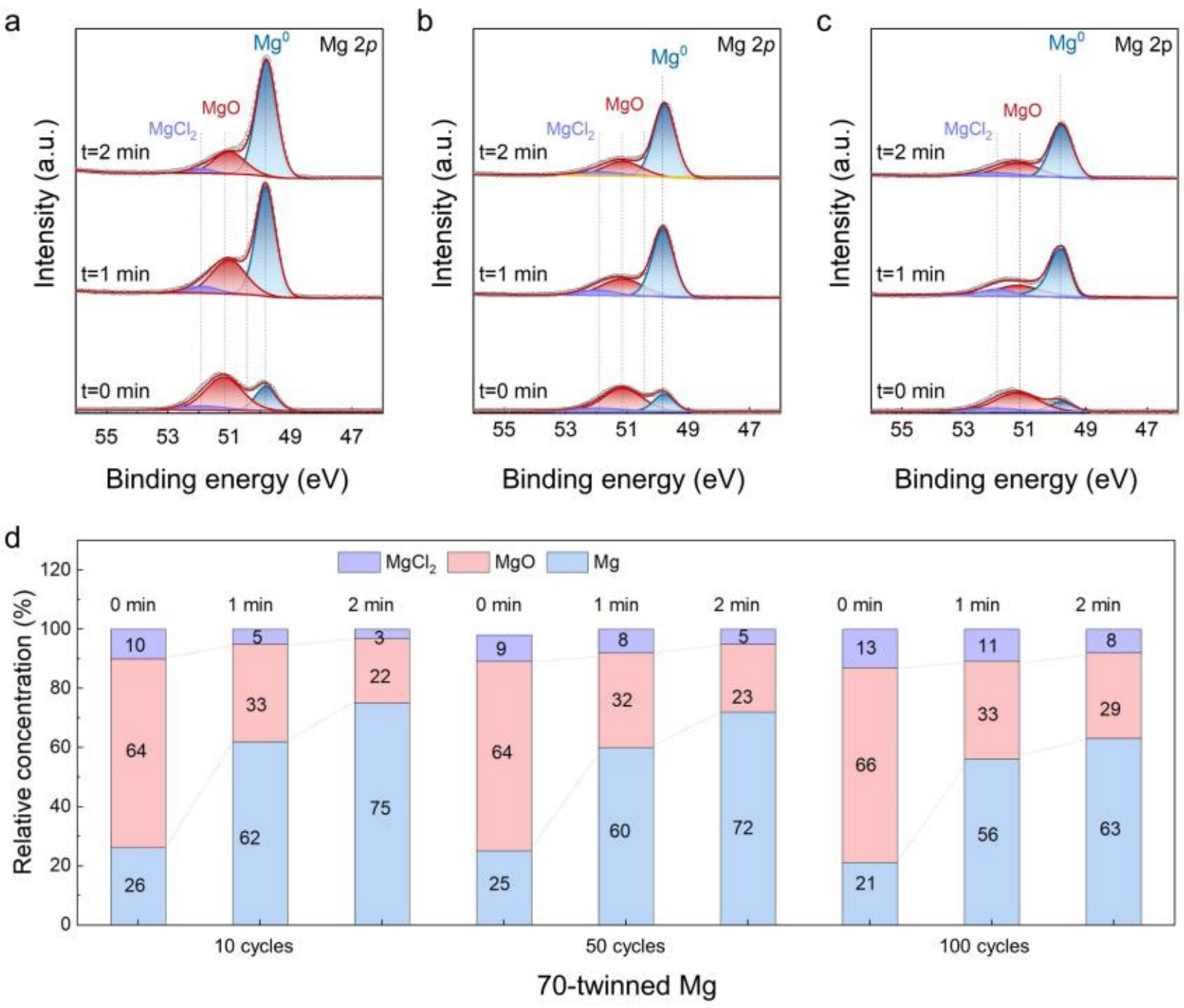


**Figure R15**. **a-c** High-resolution XPS spectra of Mg 2*p* of 70-twinned Mg after 10 cycling (a), 50 cycling (b), 100 cycling (c) at 10 mA $cm^{-2}$, 5 mAh $cm^{-2}$. **d** The relative concentrations of decomposition species measured on the surface after cycling at sputtering depths of 0, 1 and 2 min.

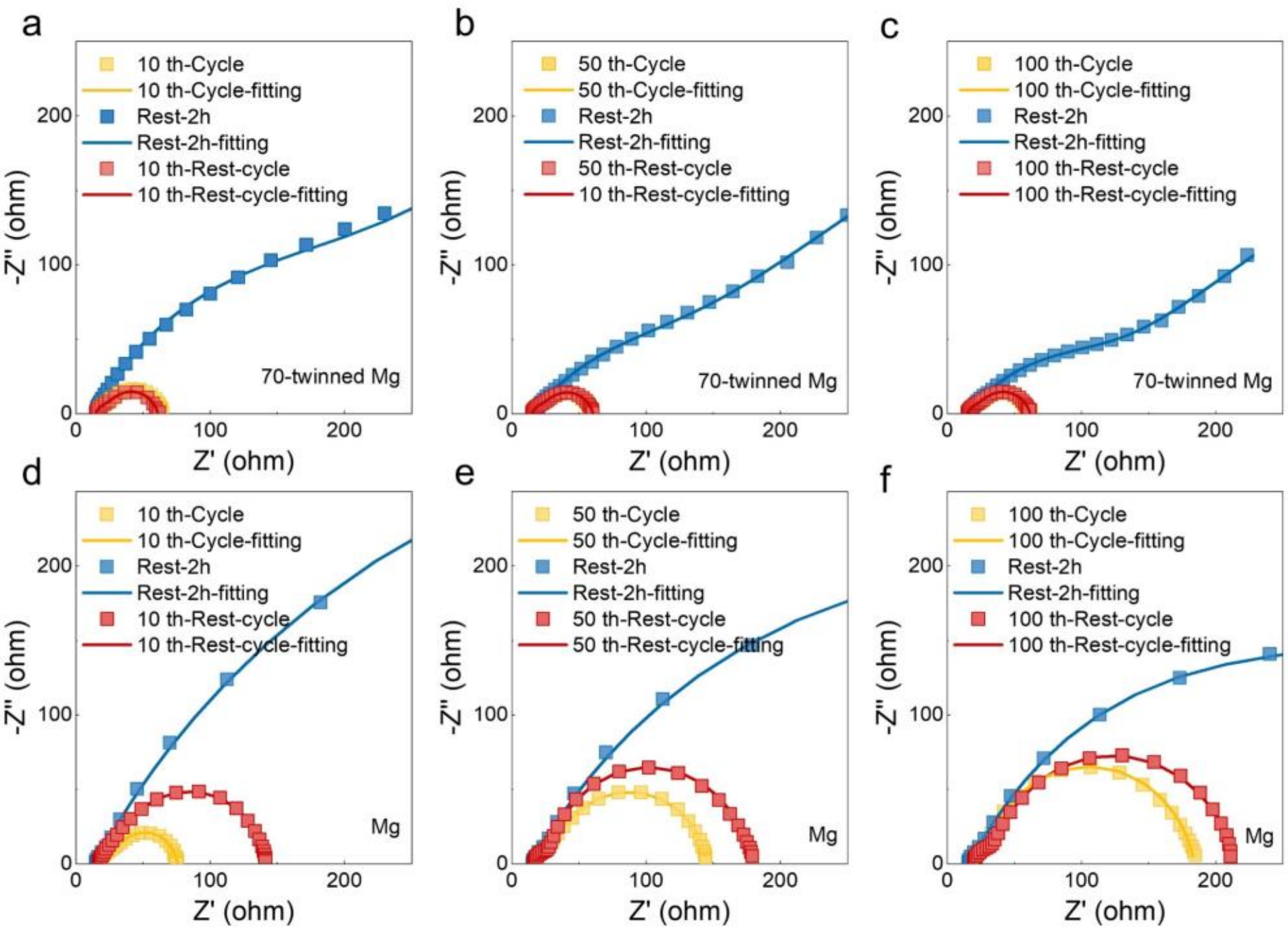


**Figure R16. a-f** The in situ EIS spectra of a 70-twinned Mg||70-twinned Mg (a-c) and a Mg||Mg cell (d-f) cell measured after 10 (a, d), 50 (b, e) and 100 (c, f) cycling, resting for 2 h, and re-cycling period, under conditions of 10 mA $cm^{-2}$ and 1 mAh $cm^{-2}$.

***We have made the following modifications to the manuscript:***

*Additionally, at the theoretical mechanism level, DFT calculations are used to demonstrate the crucial roles of electrode potential and twin structure in interfacial evolution. Considering that tetrahydrofuran (THF) is the solvent in the APC electrolyte, we used the reaction process of THF reduction to MgO as a model reaction and systematically compared the reaction thermodynamic behavior of pure Mg and twinned Mg under different electrode potentials (**Supplementary Fig. S27 and Fig. S28**). At the same electrode potential, the Gibbs free energy for THF reduction on twinned Mg is significantly higher than that on pure Mg ($\Delta G_{twinned\text{-}Mg} > \Delta G_{pure\text{-}Mg}$), indicating that the twin structure intrinsically raises the energy barrier for solvent decomposition and passivation product formation. In addition, for the same anode material, increasing the electrode potential markedly increases the Gibbs free energy of the reduction reaction, thereby suppressing interfacial side reactions. Notably, this "potential-interfacial reaction synergistic suppression effect" on twinned Mg provides clear thermodynamic evidence that twin structures favour the formation of a nearly bare-metal interface.* {Page 15, Line 12}

*Notably, the passivation products on the surface of the pure Mg electrode increased significantly after 50 cycles compared to after 10 cycles (**Supplementary Fig. S24**). However, for the 70-twinned Mg anode, the interfacial chemical composition and the relative proportions of surface species remain essentially unchanged after 10, 50, and 100 cycles (**Supplementary Fig. S25**).* {Page 14, Line 3}

*Notably, upon reapplication of the electrodeposition/stripping current, the 70-twinned Mg electrode reverted to its initial state, indicating reversible interfacial behaviour (**Fig. 5e and Supplementary Table S5**), and maintained a stable interfacial impedance even after 100 cycles (**Supplementary Fig. S26**).* {Page 14, Line 24}

*However, the impedance of pure Mg symmetric cells remained significantly higher than the initial value after cycling, revealing sluggish charge-transfer processes during deposition and stripping (**Fig. 5f and Supplementary Table S5**), which further deteriorated after 100 cycles (**Supplementary Fig. S26**).* {Page 15, Line 6}

*2) The entire study is performed exclusively in APC ($AlCl_3$/PhMgCl), an electrolyte known to inherently form Mg–Cl complex layers and promote relatively smooth deposition. It is therefore unclear whether the observed improvements originate from the twin structure or from APC's intrinsic chemistry.*

**Response:** We sincerely thank you for your insightful and profound comment. In response to your reasonable concern that the observed uniform Mg deposition might originate from the intrinsic chemistry of the APC electrolyte, we further investigated three representative chloride-free Mg electrolytes and carried out a systematic comparative analysis of deposition morphology and electrochemical performance.

Specifically, in all three chloride-free electrolyte systems, the 70-twinned Mg electrode consistently exhibits markedly more uniform deposition than the pure Mg electrode. The resulting surface morphology is smoother and denser, showing trends highly consistent with those observed in the APC electrolyte (**Figure R17**). Meanwhile, symmetric cells assembled with 70-twinned Mg under identical electrolyte conditions display clearly superior cycling stability during variable-rate cycling compared with those based on pure Mg electrodes (**Figure R18**).

In summary, these morphological characterisations and electrochemical measurements indicate that the improvement in Mg deposition behavior observed in this manuscript primarily arises from the intrinsic regulatory effect of the twin structure within the Mg electrode, rather than from the specific chemical characteristics of the APC electrolyte.

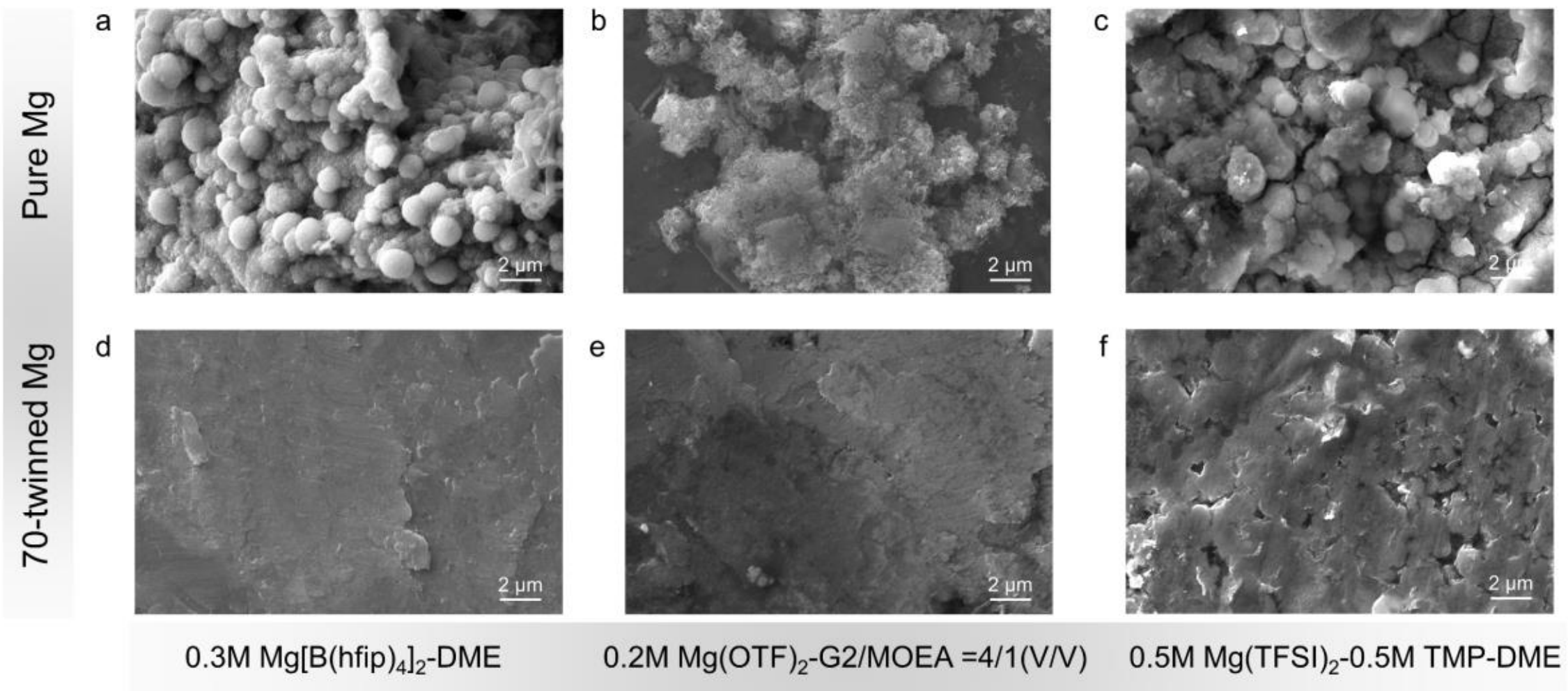


**Figure R17.** Mg deposition morphology on pure Mg (a-c) and 70-twinned Mg (d-f) surfaces under 10 mA cm$^{-2}$, with 5 mAh cm$^{-2}$ in different electrolyte. ($Mg[B(hfip)_4]_2$: Magnesium tetrakis(hexafluoroisopropyloxy)borate; DME: 1-2 dimethoxyethane; $Mg(OTF)_2$: Magnesium trifluoromethanesulphonate; G2: Diglyme; MOEA: 2-Methoxyethylamine; $Mg(TFSI)_2$: Magnesium bis(trifluoromethylsulfonyl)imide; TMP: Trimethyl phosphate)

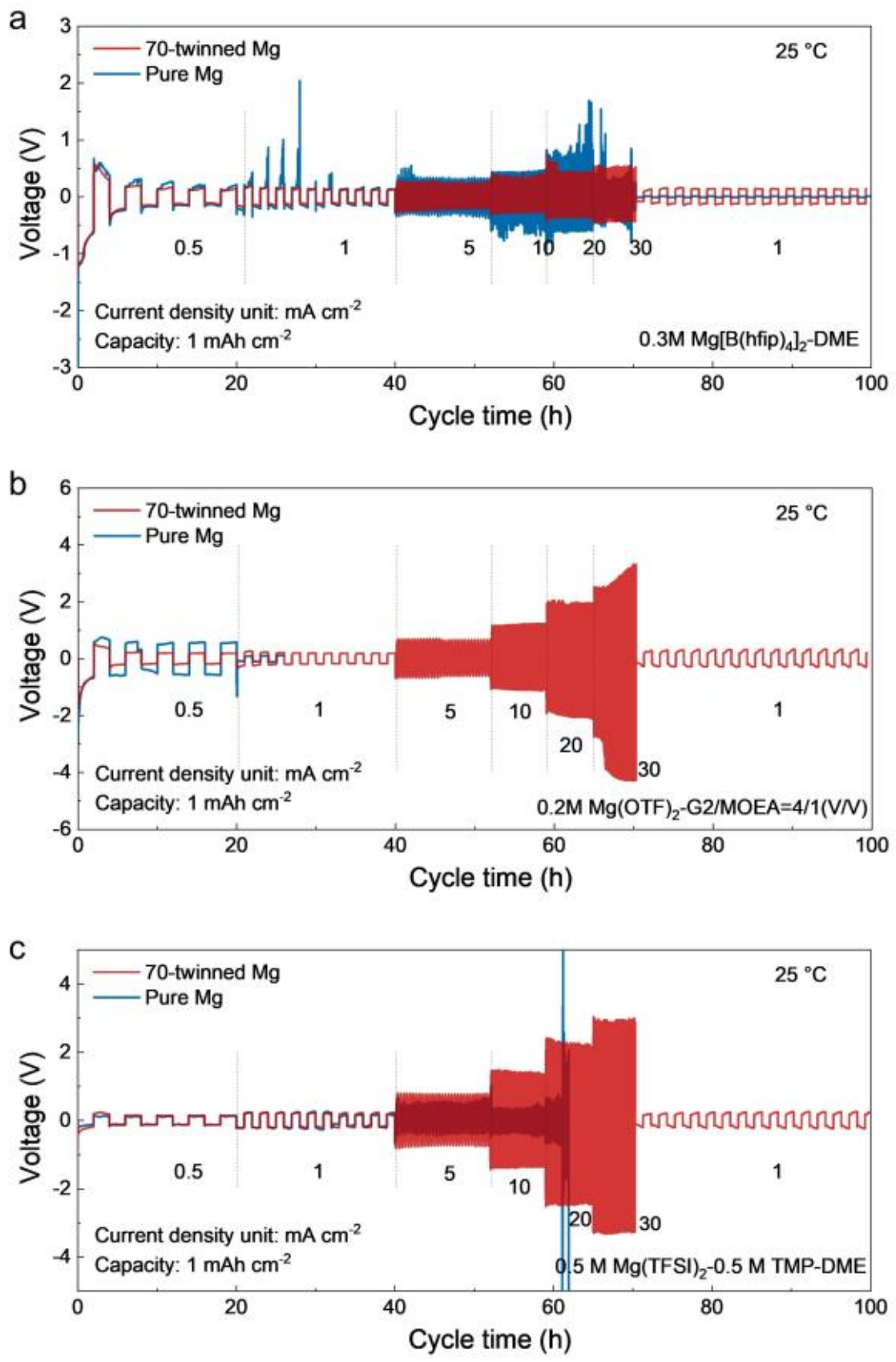


**Figure R18. a-c** Rate performance of the pure Mg and 70-twinned Mg symmetric cells in different electrolyte at various current densities with the same capacity of 1 mAh $cm^{-2}$..

***We have made the following modifications to the manuscript:***

*Notably, in the other three chloride-free electrolyte systems, the 70-twinned Mg electrode consistently exhibits markedly more uniform deposition than the pure Mg electrode (**Supplementary Fig. S8**). The resulting surface morphology is smoother and denser, showing trends highly consistent with those observed in the APC electrolyte. Therefore, the enhanced Mg deposition behavior primarily originates from the intrinsic regulatory effect of the twin structure within the Mg electrode, rather than from the specific chemical characteristics of the APC electrolyte.* {Page 7, Line 23}

*Moreover, the excellent rate performance of the 70-twinned Mg||70-twinned Mg symmetric cells can also be maintained in the other three chlorine-free electrolytes (**Supplementary Fig. S15**).* {Page 10, Line 8}

*3) Although the authors show reduced diffusion barriers along twin boundaries, the manuscript does not establish how this translates to suppressed MgO/$MgCl_2$ formation or reduced electrolyte decomposition. The mechanistic connection between crystallographic defects and chemical passivation remains speculative.*

**Response:** We sincerely thank you for your valuable comments. We agree that demonstrating reduced diffusion barriers alone is insufficient to fully elucidate how twin boundaries suppress chemical passivation, including $MgO/MgCl_2$ formation and electrolyte decomposition. Therefore, based on your valuable feedback, we have strengthened and clarified this mechanism through supplementary theoretical calculations and experimental data.

First, from a theoretical mechanism perspective, we carried out supplementary DFT calculations using tetrahydrofuran (THF), the solvent in the APC electrolyte, as a representative reactant. By modelling the reduction pathway of THF leading to MgO formation, we systematically compared the reaction thermodynamics on pure Mg and twinned Mg surfaces under different electrode potentials (**Figure R19 and R20**). At the same potential, the Gibbs free energy for THF reduction on twinned Mg is significantly higher than that on pure Mg ($\Delta G_{twinned\text{-}Mg}>\Delta G_{pure\text{-}Mg}$), indicating that the twin structure intrinsically raises the energy barrier for solvent decomposition and passivation product formation. In addition, for the same anode material, increasing the electrode potential markedly increases the Gibbs free energy of the reduction reaction, thereby suppressing interfacial side reactions. Notably, this "potential-interfacial reaction synergistic suppression effect" on twinned Mg provides clear thermodynamic evidence that twin structures favour the formation of a nearly bare-metal interface. [*Angew. Chem. 2022, 134: e202208743; J. Power Sources 2012, 200: 83-91*].

Second, to directly connect these theoretical insights with experimental interfacial chemistry, we extended the interphase analysis to longer cycling conditions (**Figure R21**). Systematic XPS measurements were performed on pure Mg and 70-twinned Mg anodes after 10, 50, and 100 cycles. For pure Mg, the contents of MgO and $MgCl_2$ species increase markedly with cycling, indicating continuous electrolyte decomposition and passivation layer growth. In sharp contrast, the twinned Mg anode exhibits nearly unchanged interfacial composition even after 100 cycles, with stable relative fractions of MgO and $MgCl_2$ related species, demonstrating that the suppression of chemical passivation is persistent rather than transient. These observations directly link the presence of twin structures to reduced accumulation of decomposition products.

Third, we further corroborated this mechanistic connection through long-term in situ EIS (**Figure R22**). The twinned Mg anode maintains a stable interfacial resistance over 100 cycles, whereas the resistance of pure Mg increase continuously. This difference in impedance evolution provides dynamic, in situ experimental evidence that enhanced surface diffusion at twin structures inhibits interface growth and reduces chemical passivation during repeated Mg plating/stripping processes.

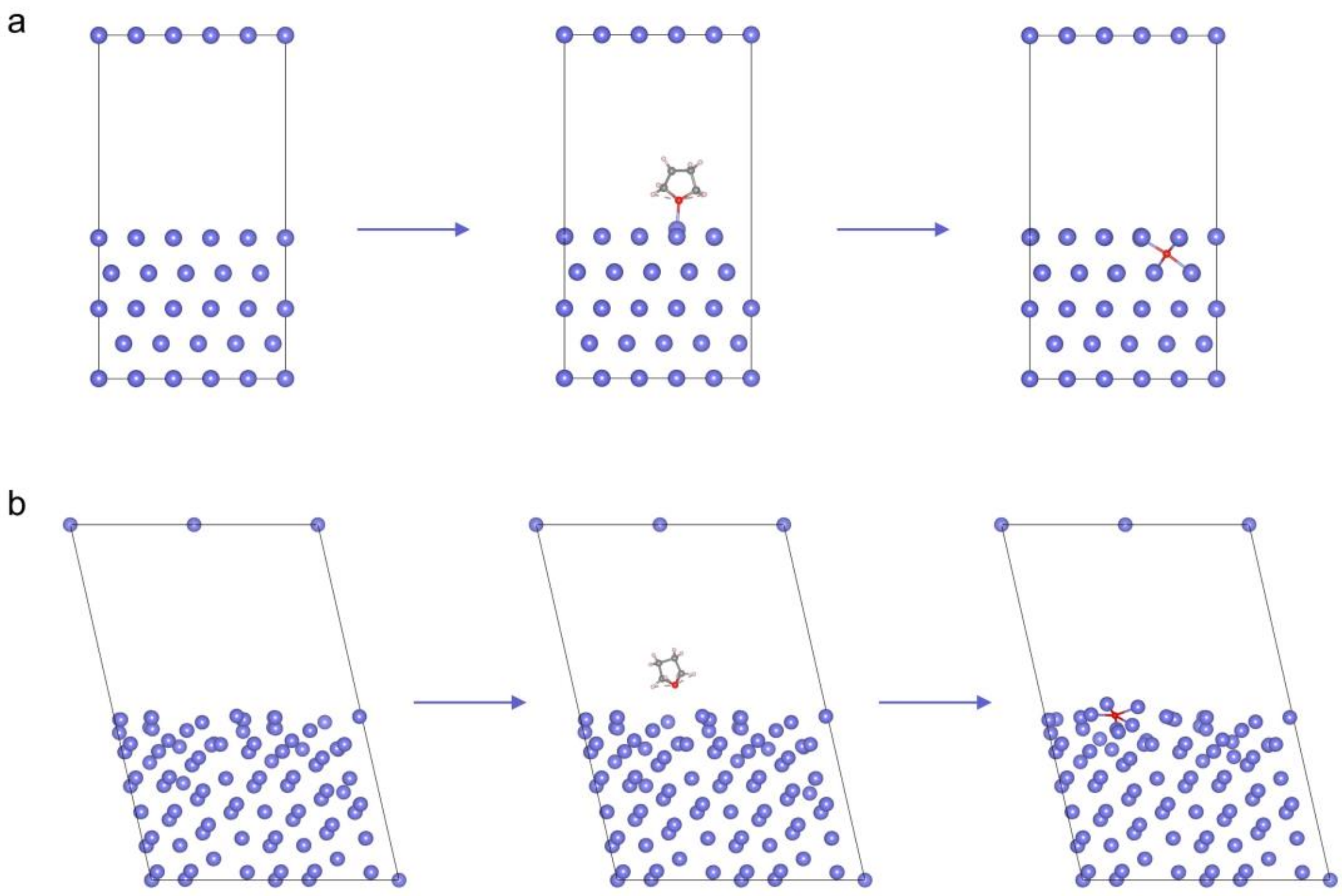


**Figure R19. a, b** Configurational changes before and after adsorption of THF molecules on pure Mg (a) and 70-twinned Mg (b) surfaces. (red, oxygen; grey, carbon; white, hydrogen; and purple, Mg).

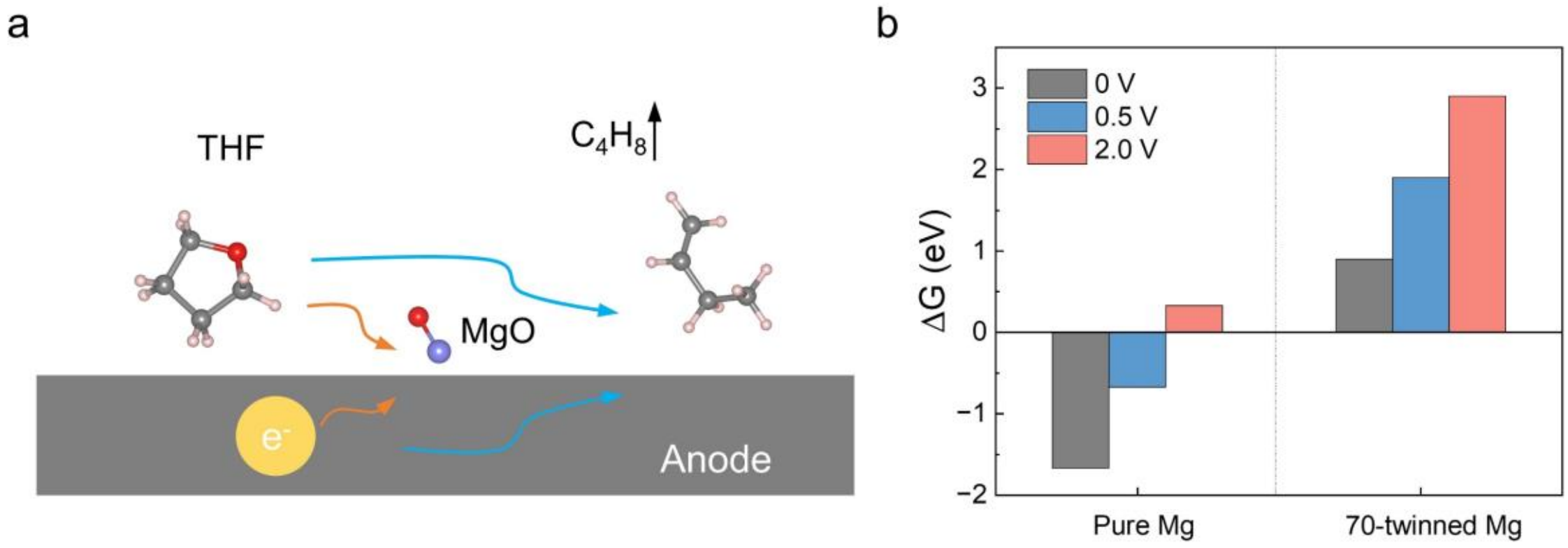


**Figure R20.** Theoretical calculation of the Gibbs free energy change of the reaction forming $C_4H_8$ or MgO on different anodes. a) Scheme of the calculation model (red, oxygen; grey, carbon; white, hydrogen; and purple, Mg); b) the free energy calculation results. (The reason for choosing potentials of 0.5 V and 2.0 V is that the overpotential of a 70-twinned Mg symmetric cell at 10 mA cm$^{-2}$ is 0.43V (~0.5 V); while the overpotential of a pure Mg symmetric cell at 10 mA cm$^{-2}$ is 2.26V (~2.0 V)).

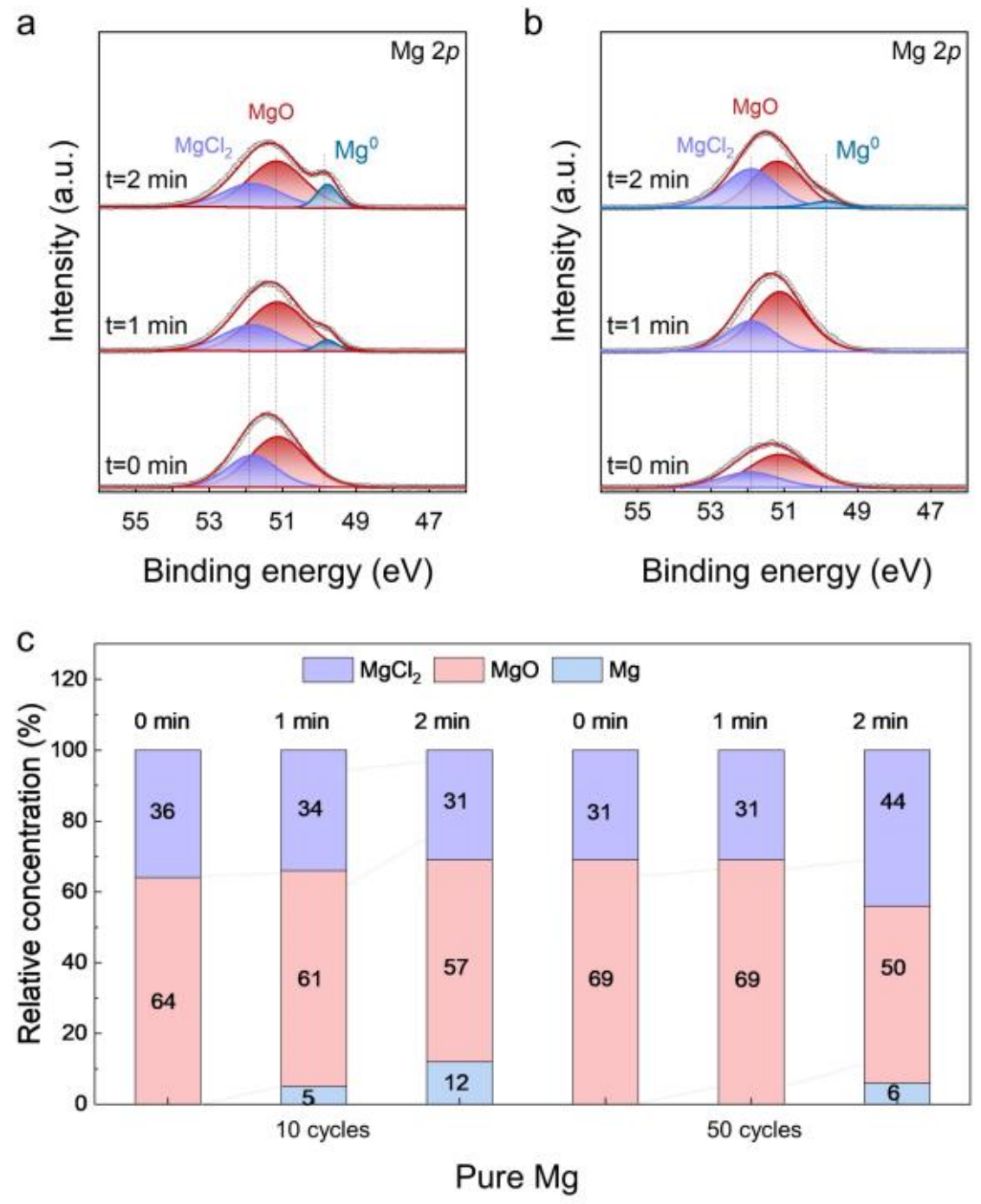


**Figure R21**. **a, b** High-resolution XPS spectra of Mg 2*p* of pure Mg after 10 cycling (a), 50 cycling (b) at 10 mA cm$^{-2}$, 5 mAh cm$^{-2}$. **c** The relative concentrations of decomposition species measured on the surface after cycling at sputtering depths of 0, 1 and 2 min.

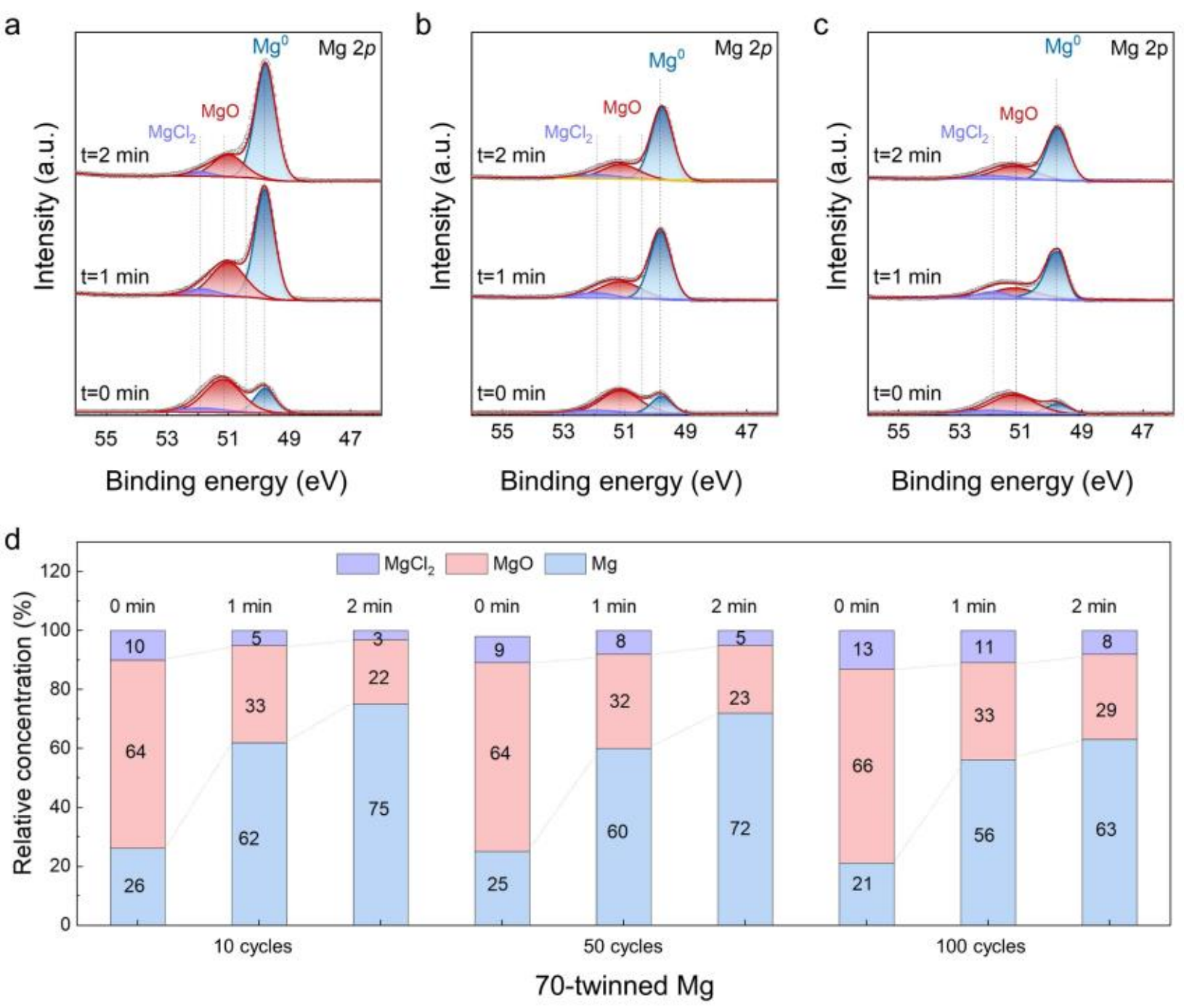


**Figure R22**. **a-c** High-resolution XPS spectra of Mg 2*p* of 70-twinned Mg after 10 cycling (a), 50 cycling (b), 100 cycling (c) at 10 mA cm$^{-2}$, 5 mAh cm$^{-2}$. **d** The relative concentrations of decomposition species measured on the surface after cycling at sputtering depths of 0, 1 and 2 min.

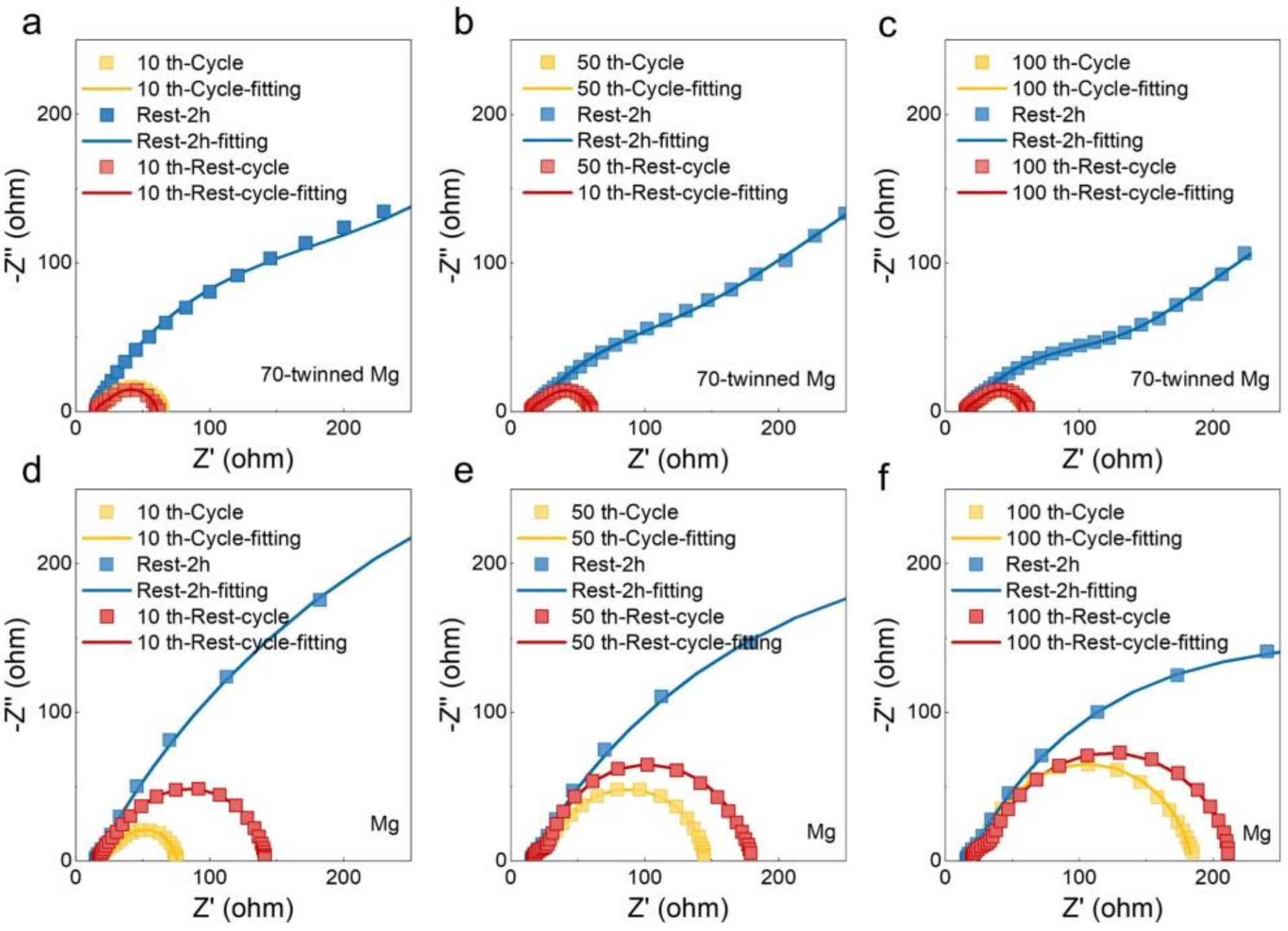


**Figure R23. a-f** The in situ EIS spectra of a 70-twinned Mg||70-twinned Mg (a-c) and a Mg||Mg cell (d-f) cell measured after 10 (a, d), 50 (b, e) and 100 (c, f) cycling, resting for 2 h, and re-cycling period, under conditions of 10 mA $cm^{-2}$ and 1 mAh $cm^{-2}$.

***We have made the following modifications to the manuscript:***

*Additionally, at the theoretical mechanism level, DFT calculations are used to demonstrate the crucial roles of electrode potential and twin structure in interfacial evolution. Considering that tetrahydrofuran (THF) is the solvent in the APC electrolyte, we used the reaction process of THF reduction to MgO as a model reaction and systematically compared the reaction thermodynamic behavior of pure Mg and twinned Mg under different electrode potentials (**Supplementary Fig. S27 and Fig. S28**). At the same electrode potential, the Gibbs free energy for THF reduction on twinned Mg is significantly higher than that on pure Mg ($\Delta G_{twinned\text{-}Mg} > \Delta G_{pure\text{-}Mg}$), indicating that the twin structure intrinsically raises the energy barrier for solvent decomposition and passivation product formation. In addition, for the same anode material, increasing the electrode potential markedly increases the Gibbs free energy of the reduction reaction, thereby suppressing interfacial side reactions. Notably, this "potential-interfacial reaction synergistic suppression effect" on twinned Mg provides clear thermodynamic evidence that twin structures favour the formation of a nearly bare-metal interface.* {Page 15, Line 12}

*Notably, the passivation products on the surface of the pure Mg electrode increased significantly after 50 cycles compared to after 10 cycles (**Supplementary Fig. S24**). However, for the 70-twinned Mg anode, the interfacial chemical composition and the*

*relative proportions of surface species remain essentially unchanged after 10, 50, and 100 cycles (**Supplementary Fig. S25**).* {Page 14, Line 3}

*Notably, upon reapplication of the electrodeposition/stripping current, the 70-twinned Mg electrode reverted to its initial state, indicating reversible interfacial behaviour (**Fig. 5e and Supplementary Table S5**), and maintained a stable interfacial impedance even after 100 cycles (**Supplementary Fig. S26**).* {Page 14, Line 24}

*However, the impedance of pure Mg symmetric cells remained significantly higher than the initial value after cycling, revealing sluggish charge-transfer processes during deposition and stripping (**Fig. 5f and Supplementary Table S5**), which further deteriorated after 100 cycles (**Supplementary Fig. S26**).* {Page 15, Line 6}

*4) Although DFT calculations illustrate reduced diffusion barriers, the correlation between theoretical predictions and observed morphology requires deeper analysis. For example, the manuscript does not clarify how the Damköhler number analysis corresponds to experimental nucleation behavior under realistic conditions.*

**Response:** We greatly appreciate your insightful comment. To address the relationship between DFT-derived diffusion barriers, Damköhler (Da) number analysis, and realistic nucleation behavior, we performed a series of experiments designed to bridge theory and experiment.

Specifically, Mg single crystals were grown using a Bridgman-type crystal growth furnace, followed by crystallographic orientation cutting to obtain four well-defined Mg single-crystal surfaces: (0002)-Mg, (10$\bar{1}$1)-Mg, (10$\bar{1}$2)Mg, and (10$\bar{1}$0)-Mg (**Figure R24**). The experimental parameters are as follows: To prepare Mg single crystals with a diameter of 12 mm and a height of 30 mm, a dual-temperature zone heating method was used to establish a stable temperature gradient. The high-temperature zone was set at 780˚C, and the low-temperature zone was set at 600˚C, with the temperature gradient precisely controlled within 1˚C /cm. The crucible descent rate was adjusted to 10 mm/h, and Ar gas was used as a protective atmosphere throughout the experiment to prevent oxidation contamination of the Mg single crystals during preparation. These surfaces correspond exactly to the crystal planes used in our theoretical calculations of adsorption energy, surface diffusion barriers, and associated Da numbers.

We then systematically examined the Mg deposition morphologies on these four crystallographic surfaces under electrochemical conditions at a current density of 10 mA $cm^{-2}$ and a capacity of 2 mAh $cm^{-2}$ (**Figure R25**). The experimental results show that (0002)-Mg, (10$\bar{1}$1)-Mg, and (10$\bar{1}$2)-Mg surfaces predominantly exhibit island-type deposition morphologies, while the (10$\bar{1}$0)-Mg surface displays even more pronounced deposition inhomogeneity, accompanied by localized dendritic features. Importantly, these observed morphologies are fully consistent with the Da-based theoretical calculations, where higher

Da values (reactions dominate) favor localized nucleation and island growth, whereas lower Da values (diffusion predominate) correspond to more uniform deposition tendencies. This one-to-one correspondence directly validates the effectiveness of the Damköhler analysis, which links surface diffusion kinetics and reaction kinetics to deposition morphology under actual electrochemical conditions.

For the 70-twinned Mg electrode, the structure is more complex due to its heterogeneous surface nature. EBSD analysis confirmed the presence of multiple crystal orientations within the matrix, along with approximately 70% of the internal interfaces are $(10\bar{1}2)$ typed tension twin (**Figure R26**). Notably, deposition on pristine single-crystal surfaces alone leads to island-like morphologies rather than planar growth (**Figure R27**), indicating that the observed uniform deposition on 70-twinned Mg cannot be explained solely by basal-plane exposure. Therefore, in our analysis, we adopt a justified approximation by identifying twin boundaries as the dominant structural feature governing deposition behavior. These twin boundaries reduces Mg diffusion barriers, as demonstrated by DFT, effectively accelerating surface Mg transport and lowering the Da number to values far below 1 (**Figure R27**). As a result, the presence of a high density of twin boundaries shifts the effective deposition regime from diffusion-limited statement toward a state in which diffusion constraints are substantially alleviated, enabling more homogeneous nucleation and planar growth. In this sense, the Da number serves as the link between diffusion kinetics at the atomic level and microstructural deposition patterns, meaning that the deposition behavior observed at the electrode can be explained by calculating diffusion rates and reaction rates [*ACS Energy Lett. 2019, 4: 375-376; Nat. Commun. 2025, 16: 1795*].

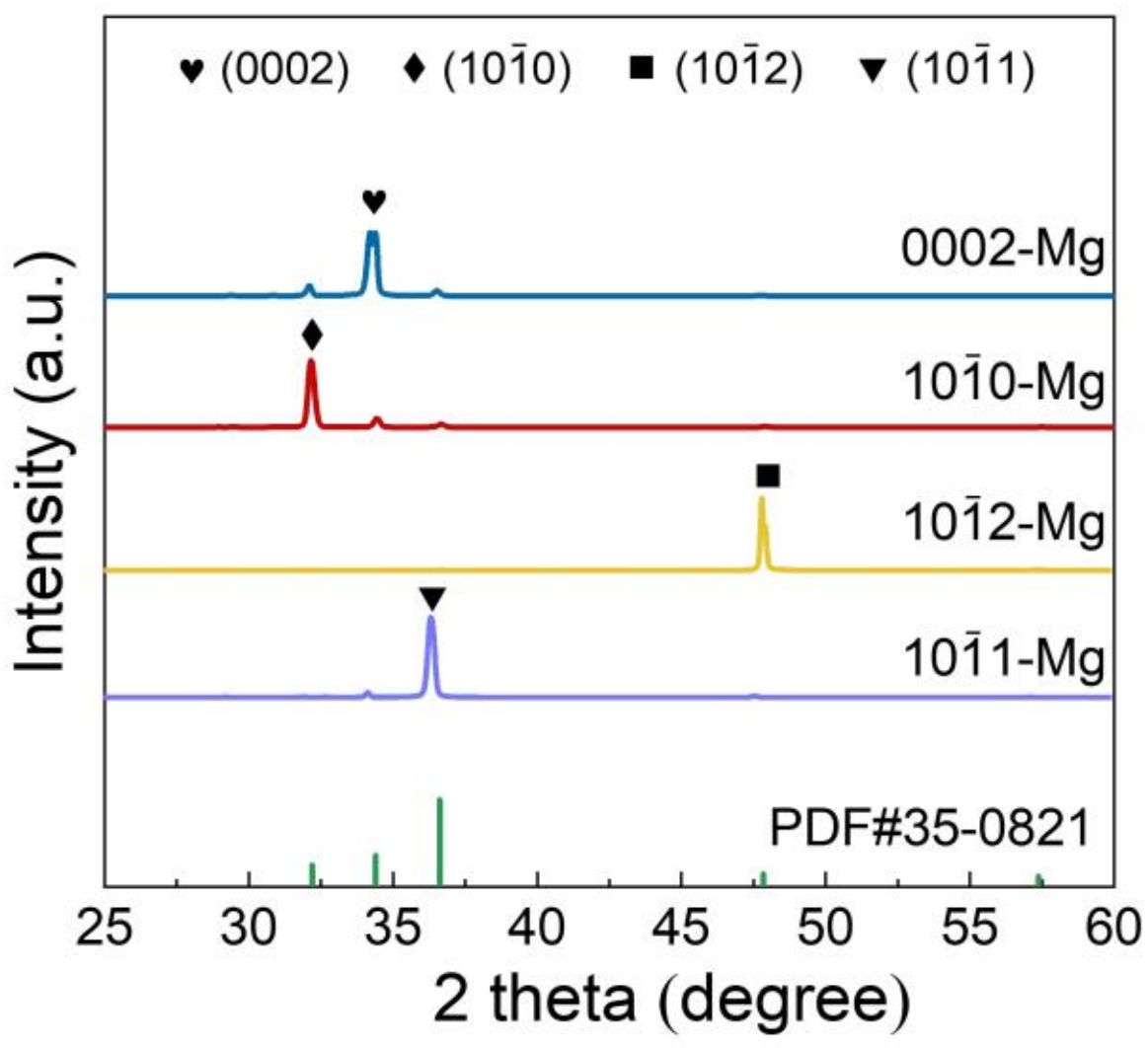


**Figure R24** XRD patterns of four Mg single crystals: (0002)-Mg, $(10\bar{1}1)$-Mg, $(10\bar{1}2)$-Mg, and $(10\bar{1}0)$-Mg.

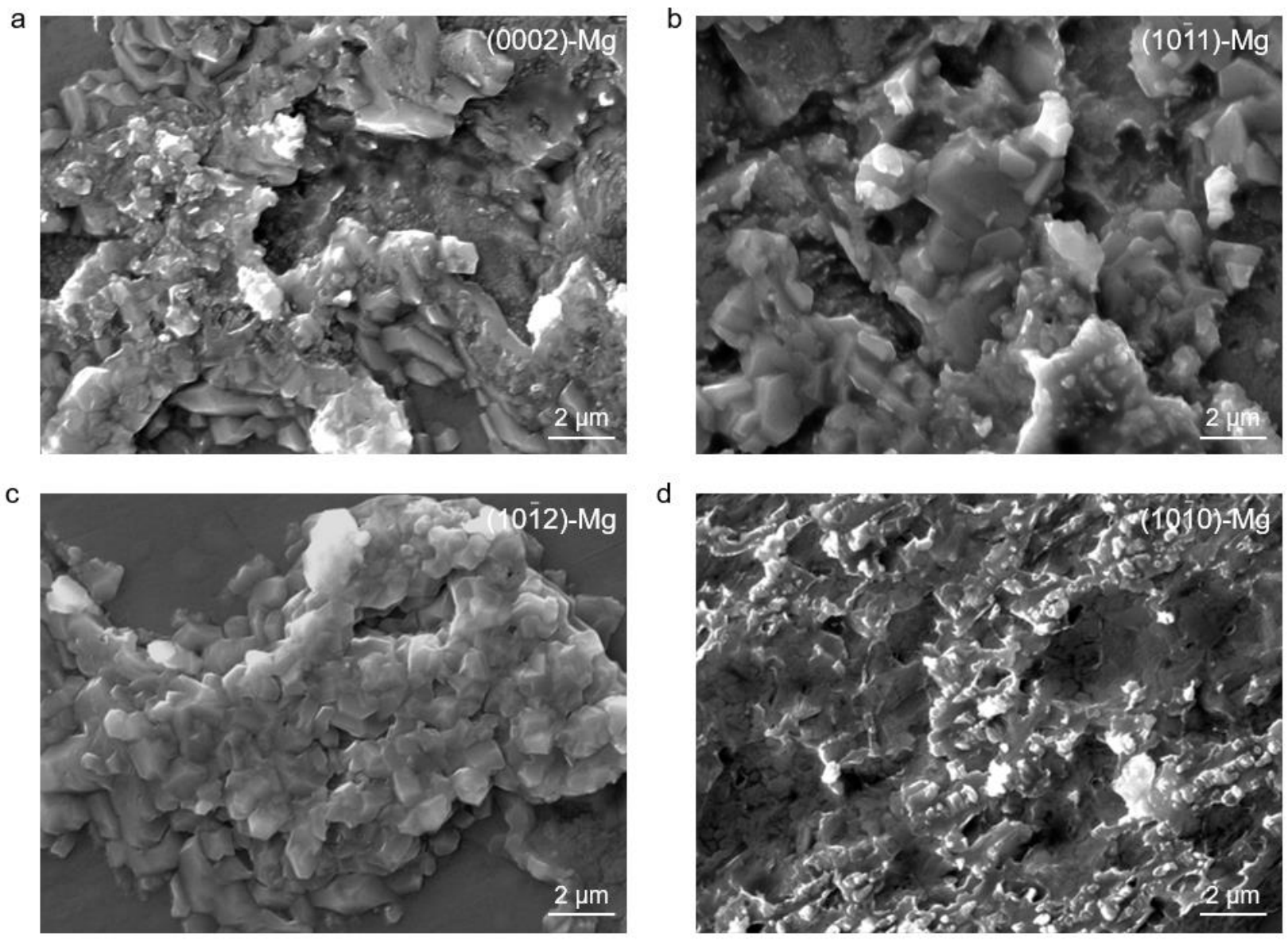


**Figure R25 a-d** SEM morphologies of magnesium deposited on (0002)-Mg, (10$\bar{1}$1)-Mg, (10$\bar{1}$2)-Mg, and (10$\bar{1}$0)-Mg surfaces at a current density of 10 mA cm$^{-2}$ and a capacity of 2 mAh cm$^{-2}$.

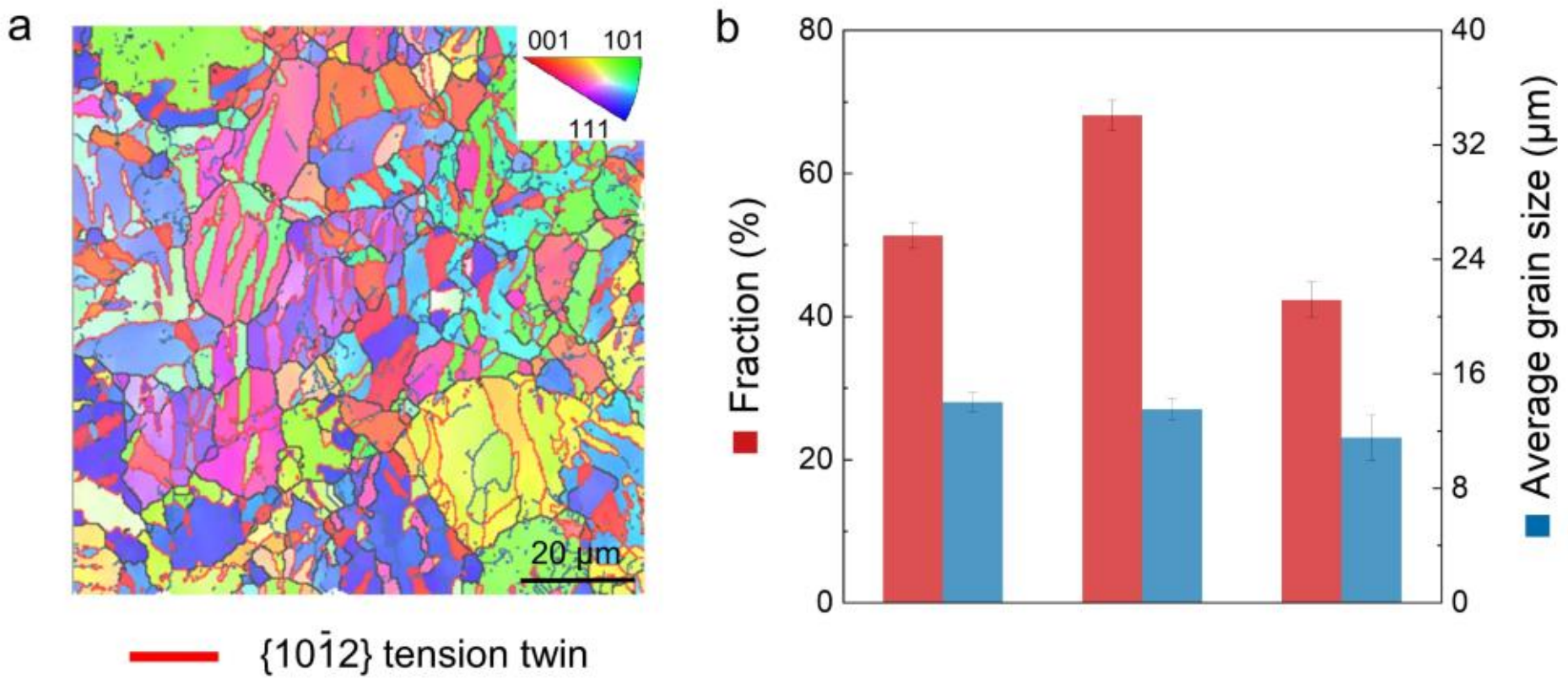


**Figure R26 a** Orientation maps of 70-twinned Mg. **b** Fraction of twins and average grain sizes of different anodes.

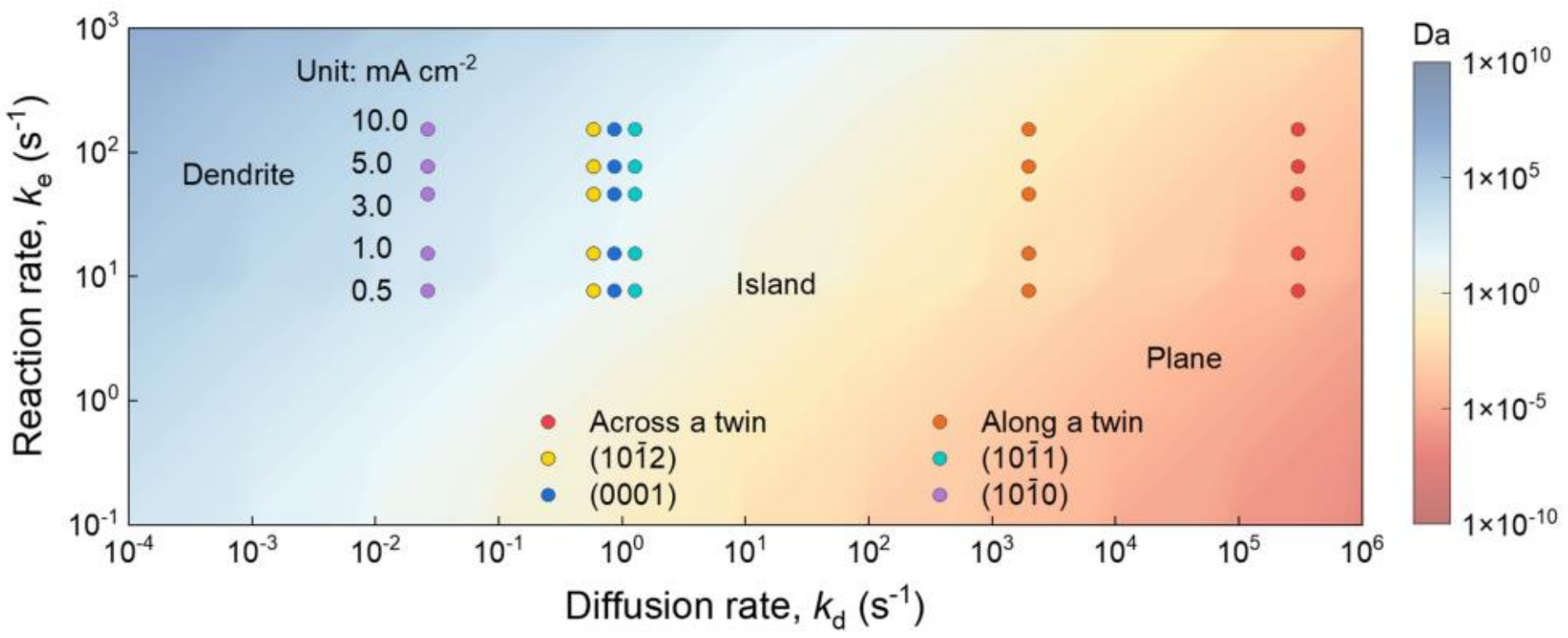


**Figure R27** Deposition morphology phase map as a function of Damköhler number.

***We have made the following modifications to the manuscript:***

*Notably, we further obtain four Mg single-crystal surfaces with well-defined orientations and correlate the observed Mg deposition behaviors on these surfaces with the corresponding Da number results. This one-to-one comparison more clearly validates the reliability of the Da analysis in describing deposition kinetic differences among different crystallographic planes (**Supplementary Fig. S19 and Fig. S20 and Supplementary Note 4**). In contrast, the presence of twinned structures in twinned Mg substantially alleviates diffusion limitations, resulting Da values much smaller than 1 and thereby promoting uniform, planar Mg deposition (**Fig. 4i and Supplementary Note 4**).* {Page 12, Line 1}

***Supplementary Note 4***

*Mg single crystals were grown using a Bridgman-type crystal growth furnace, ……, These surfaces correspond exactly to the crystal planes used in our theoretical calculations of adsorption energy, surface diffusion barriers, and associated Da numbers.* {Supporting information Page 45, Line 1}

*We then systematically examined the Mg deposition morphologies ……, which links surface diffusion kinetics and reaction kinetics to deposition morphology under actual electrochemical conditions.* {Supporting information Page 45, Line 13}

*For the 70-twinned Mg electrode, the structure is more complex …… meaning that the deposition behavior observed at the electrode can be explained by calculating diffusion rates and reaction rates.* {Supporting information Page 45, Line 23}

---------------------------------------------------

**Reply to the report of referee #3**

---------------------------------------------------

**Comment of Referee #3:**

Reviewer 3

*The submitted paper draft reports investigations about Twinned-magnesium metal anodes which show highly reversible electrodeposition, with, on the one hand, impressive performance, an interesting procedure for the anodes preparation and impressive claims overall. On the other hand, there are some fundamental flaws in the application of electrochemical models and methodologies which make the supporting investigations invalid, thus compromising the whole argument.*

*For this reason, this draft cannot be published in Nature Communications. Given the lack of scientifically solid and sound foundations, major revisions would not be enough to address its shortcomings.*

**Response:** We are grateful for your critical evaluation and we hope that the revised version has answered all your comments.

*1) Electrochemical experiments are constantly ran in a 2-electrode setup; considering the very sensitive subject of multivalent battery chemistries, in which a clear definition of potential values is fundamental, that´s already a non-negligible flaw in the foundations of this study.*

**Response:** We sincerely thank you for your professional comments. Your points regarding the electrochemical experimental setup are crucial, and we fully agree with your emphasis on the importance of clear potential definition in multivalent metal battery systems. Therefore, we have supplemented the relevant experimental data to address your concerns.

Specifically, in studies involving multivalent metal battery systems, the presence of pronounced polarisation, complex deposition/stripping processes, and distinct interfacial kinetic limitations makes an accurate determination of the true working-electrode potential essential. In a conventional two-electrode configuration, the measured voltage represents the overall potential difference between the working and counter electrodes, inevitably incorporating contributions from counter-electrode polarisation, electrolyte resistance, and interfacial reactions. While such a configuration is well suited for evaluating the overall reversibility and long-term cycling stability of symmetric cells, it does not allow for a rigorous, quantitative assessment of the intrinsic thermodynamic and kinetic behaviour of the working electrode [*Energy Storage Mater. 2025, 82: 104632;* ]. By contrast, a three-electrode configuration, through the introduction of an independent reference electrode (e.g. Mg or $Ag/Ag^{+}$) [*Batteries Supercaps 2024, 7: e202400250; Nat. Commun. 2025, 16: 3142; Nat. Commun. 2023, 14: 7080*], enables direct and stable monitoring of the working-

electrode potential with respect to a well-defined reference, thereby allowing a more precise characterisation of interfacial electrochemical processes.

Based on this consideration, we have incorporated chronoamperometry (CA) measurements performed in a three-electrode configuration in the revised manuscript to directly probe nucleation behaviour on different electrodes (**Figure R28**) [*Batteries Supercaps 2024, 7: e202400250*]. In these experiments, a stable and electrolyte-compatible pure Mg foil was used as the reference, the working electrode consisted of the investigated substrate (pure Mg or twinned Mg), and the counter electrode was pure Mg. The results show that CA transients recorded under constant overpotential conditions clearly distinguish the nucleation behaviour on different substrates: pure Mg exhibits characteristic three-dimensional instantaneous nucleation, whereas twinned Mg shows a progressive nucleation mode, directly reflecting the intrinsic role of twin structures in regulating early-stage deposition kinetics (**Figure R29**) . In addition, the three-electrode configuration and its implementation method have been explicitly described in the experimental section and the electrochemical characterisation part of the manuscript.

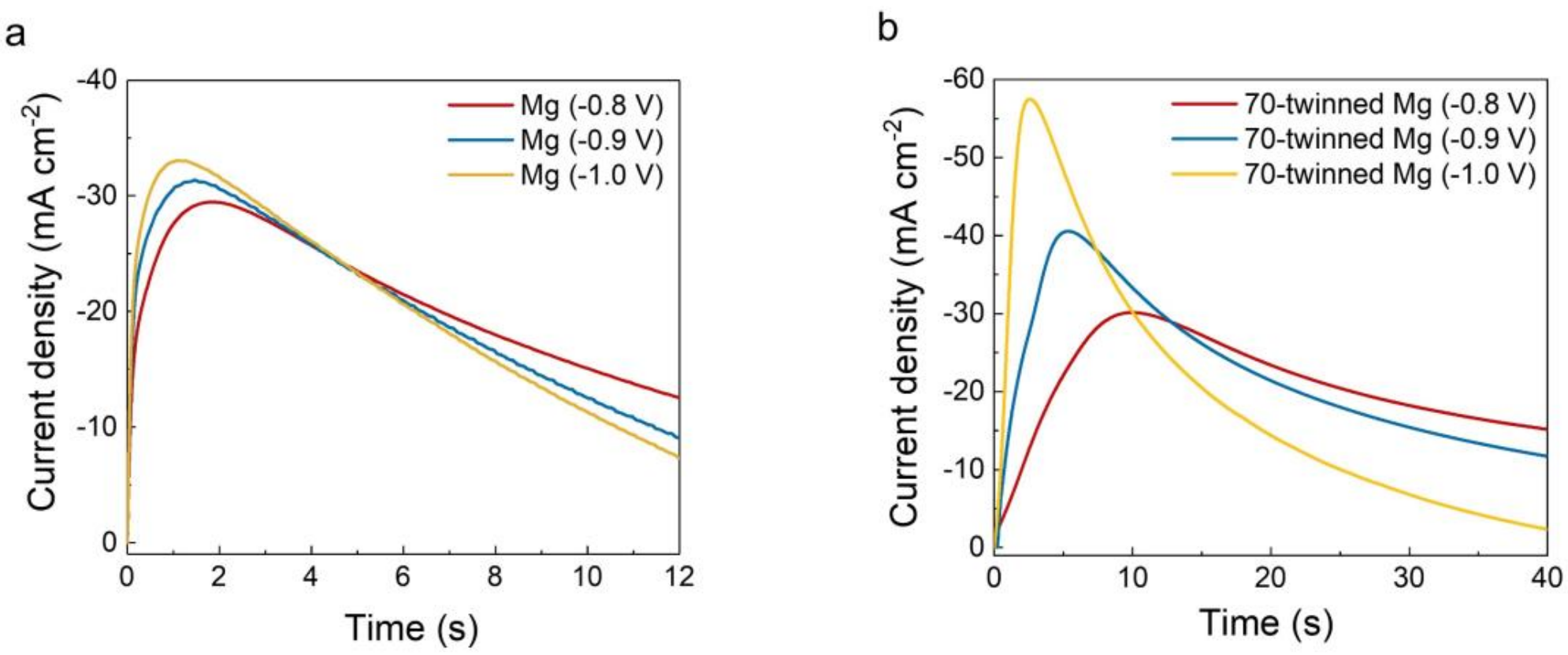


**Figure R28** Current-time transients obtained at predetermined potentials.

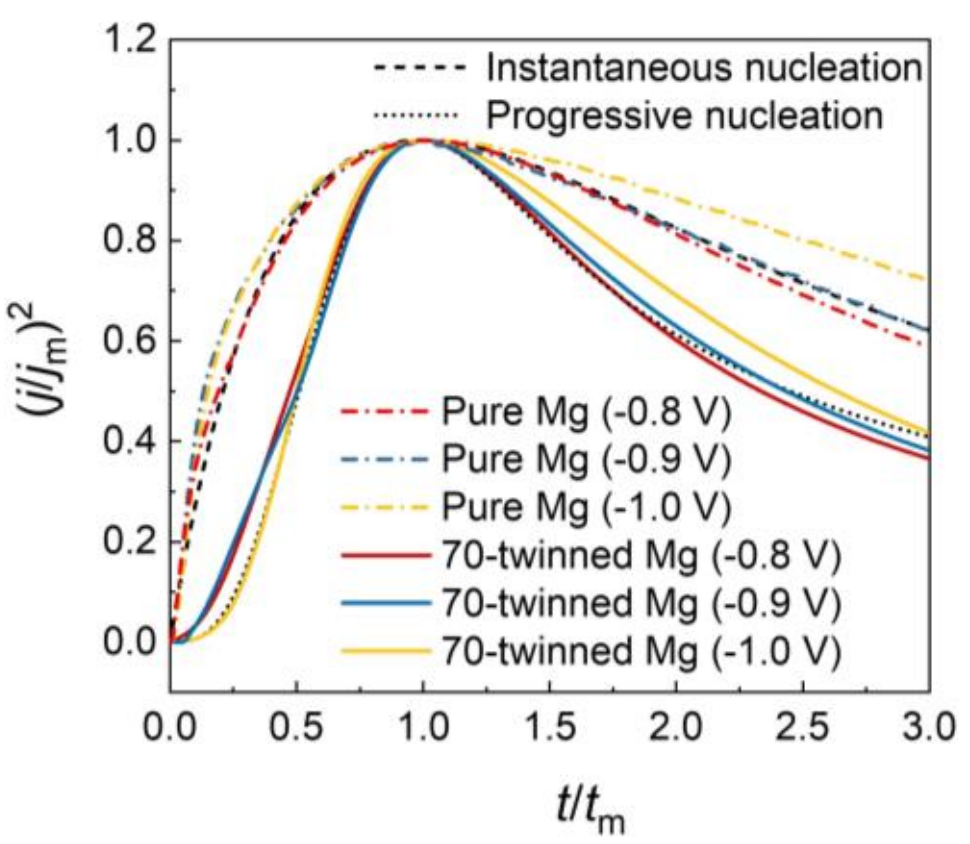


**Figure R29** Plots of $t/t_m$ and $(j/j_m)^2$ in comparison with theoretical 3D models for a Mg cell using Mg and 70-twinned Mg electrodes.

***We have made the following modifications to the manuscript:***

*All CA experiments discussed herein were conducted in a rigorously defined three-electrode configuration using an asymmetric cell, in which pure Mg served as both the reference electrode and counter electrode, while pure Mg or twinned Mg were used as the working electrodes. This configuration ensures an unambiguous definition of the working-electrode potential and eliminates polarization contributions from the counter electrode, thereby satisfying the fundamental requirements for kinetic analysis based on nucleation models.* {Page 22, Line 5}

*2) One fundamental flaw is the misapplication and reporting of the Scharifker-Hills model on nucleation and growth. Once more, and more importantly here, a 3-electrode setup is of paramount importance for the clear definition of the potential values, and that is missing. The model in its application involves the sequential repetitions of step-wise chronoamperometric measurements within small intervals to check whether the model is applicable, through the calculation of a diagnostic parameter, which is the product (jmax)2*tmax which in principle should remain if not constant, in a very close range of values. this observation alone makes the application of the model in this draft invalid and conceptually wrong. The draft does not unambiguously states if a symmetric cell or an asymmetric cell is used. Only one potential is reported and more importantly the curve fittings, which don´t overlap completely with the theoretical curves (3DP and 3DI) in the most important region of the plot, i.e. before tmax, are just assigned to a general "mixed 3DP + 3DI" mechanism which, has a limited to none reference in literature; the statement anyway provides no reference or further explanation.*

**Response:** We sincerely thank you for your detailed and professional comments. We fully acknowledge that an unambiguous potential definition and the correct application of the Scharifker–Hills (S–H) nucleation model are fundamental prerequisites for any meaningful kinetic interpretation, particularly in multivalent metal systems. In response to your concerns, we have comprehensively re-designed the relevant electrochemical experiments, re-analysed the data, and substantially revised the manuscript to ensure conceptual correctness and experimental rigor.

First, regarding the electrochemical configuration and potential definition, we fully agree that the use of a three-electrode setup is essential for applying the S–H model. In a two-electrode configuration, the measured potential inevitably includes counter-electrode polarization and electrolyte resistance, which precludes an accurate definition of the working-electrode overpotential and inaccurates nucleation analysis. Accordingly, in the revised manuscript, all chronoamperometric (CA) experiments used for S–H analysis were re-conducted exclusively in a three-electrode configuration. All measurements were performed in an asymmetric cell, employing a stable and electrolyte-compatible pure Mg

reference electrode, the investigated electrode (pure Mg or twinned Mg) as the working electrode, and pure Mg was used as the counter electrode. This configuration ensures that the applied overpotentials (-0.8, -0.9, and -1.0 V) are defined with respect to a stable Mg reference, providing a thermodynamic driving force for nucleation (**Figure R30**). In addition, the cell configuration and electrode roles are stated in the Method Section to avoid any ambiguity.

Second, concerning the validity of the S–H model application and the diagnostic parameter ($(j_{max})^2 * t_{max}$), its approximate constancy in stepwise potential measurements is a necessary condition for the applicability of the S-H model [*Batteries Supercaps 2024, 7: e202400250; Results in Surfaces and Interfaces 2022, 6: 100044; J. Solid State Electrochem. 2021, 25: 1889-1900*]. Therefore, based on S-H model, we performed sequential, step-wise CA measurements under closely spaced overpotentials (-0.8, -0.9, and -1.0 V) and re-evaluated the diagnostic parameter. Specifically, the re-analysed data show that for twinned Mg electrodes, ($(j_{max})^2 * t_{max}$) values of $9.09*10^3$, $8.77*10^3$, and $8.46*10^3$ $mA^2$ s $cm^{-4}$ were obtained at -0.8, -0.9, and -1.0 V, respectively, while for pure Mg electrodes the corresponding values are $1.57*10^3$, $1.45*10^3$, and $1.20*10^3$ $mA^2$ s $cm^{-4}$ (**Table R2**). These values remain within a very close range and fall well inside the experimental variability commonly accepted in classical electrochemical nucleation studies, particularly when considering unavoidable minor variations in real electrode surface states [*Batteries Supercaps 2024, 7: e202400250*; *J. Appl. Electrochem. 2020, 50: 159-168*]. This consistency confirms that the experimental conditions satisfy the applicability requirements of the S-H model and that the previous concerns regarding conceptual invalidity have been addressed.

Finally, regarding curve fitting, mechanism assignment, and the interpretation of deviations from ideal theoretical transients, we have re-fitted and re-presented all CA data (**Figure R31**). The agreement between experimental normalized transients and the theoretical 3DI and 3DP curves is now significantly improved, especially in the kinetically critical region prior to $t_{max}$. Importantly, we have removed the previously ambiguous and insufficiently supported description of a generic “mixed 3DI + 3DP” mechanism. Instead, we now base our interpretation strictly on the normalized $(j/j_{max})^2$ *vs*. ($t/t_{max}$) evolution, in line with established S–H analysis. This revised analysis clearly shows that pure Mg exhibits behavior closer to 3DI, whereas twinned Mg consistently follows a 3DP mechanism [*Batteries Supercaps 2024, 7: e202400250; Nat. Commun. 2025, 16: 3142; Nat. Commun. 2023, 14: 7080*]. In addition, the minor deviations from the ideal theoretical curve at the early stage (t<tmax) does not indicate a change in the nucleation mechanism but instead arises from intrinsic non-idealities in real electrochemical systems. First, practical electrode surfaces inevitably exhibit non-uniform distributions of surface energy and active sites, which leads to minor deviations from the idealised theoretical response. Second, immediately following the potential step, a substantial fraction of the initial current

originates from double-layer capacitive charging; this non-Faradaic contribution is superimposed on the Faradaic nucleation current, resulting in a transient deviation from the theoretical curve. Such behaviour is commonly observed in studies and reflects an inherent experimental artefact rather than a mechanistic difference [*Batteries Supercaps 2024, 7: e202400250; Nat. Commun. 2023, 14: 7080; Adv. Mater. 2024, 36: 2406257; Adv. Funct. Mater., 2026: e27860; Adv. Mater. 2026, 38: e16427*].

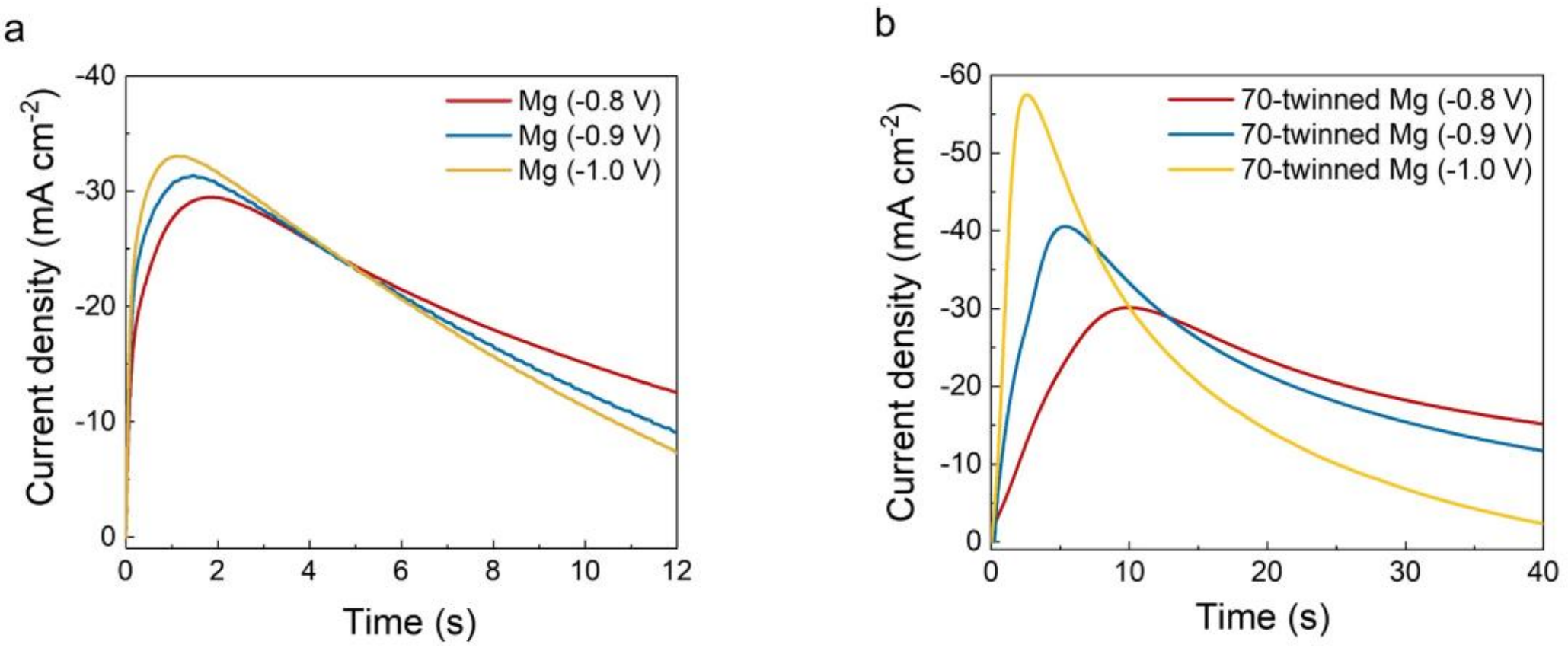


**Figure R30** Current-time transients obtained at predetermined potentials.

**Table R2** Summary of $t_{max}*(j_{max})^2$ values as requirement for the applicability of the S-H Method.

| | 70-twinned Mg | | | Pure Mg | | |
|---|---|---|---|---|---|---|
| Potential (vs. $Mg/Mg^{2+}$) | -0.8 V | -0.9 V | -1.0 V | -0.8 V | -0.9 V | -1.0 V |
| $j_m$ | -30.13 | -40.56 | -57.49 | -29.48 | -31.37 | -33.05 |
| $t_m$ | 10.01 | 5.33 | 2.56 | 1.81 | 1.47 | 1.10 |
| $t_m*j_m^2$ | $9.09*10^3$ | $8.77*10^3$ | $8.46*10^3$ | $1.57*10^3$ | $1.45*10^3$ | $1.20*10^3$ |

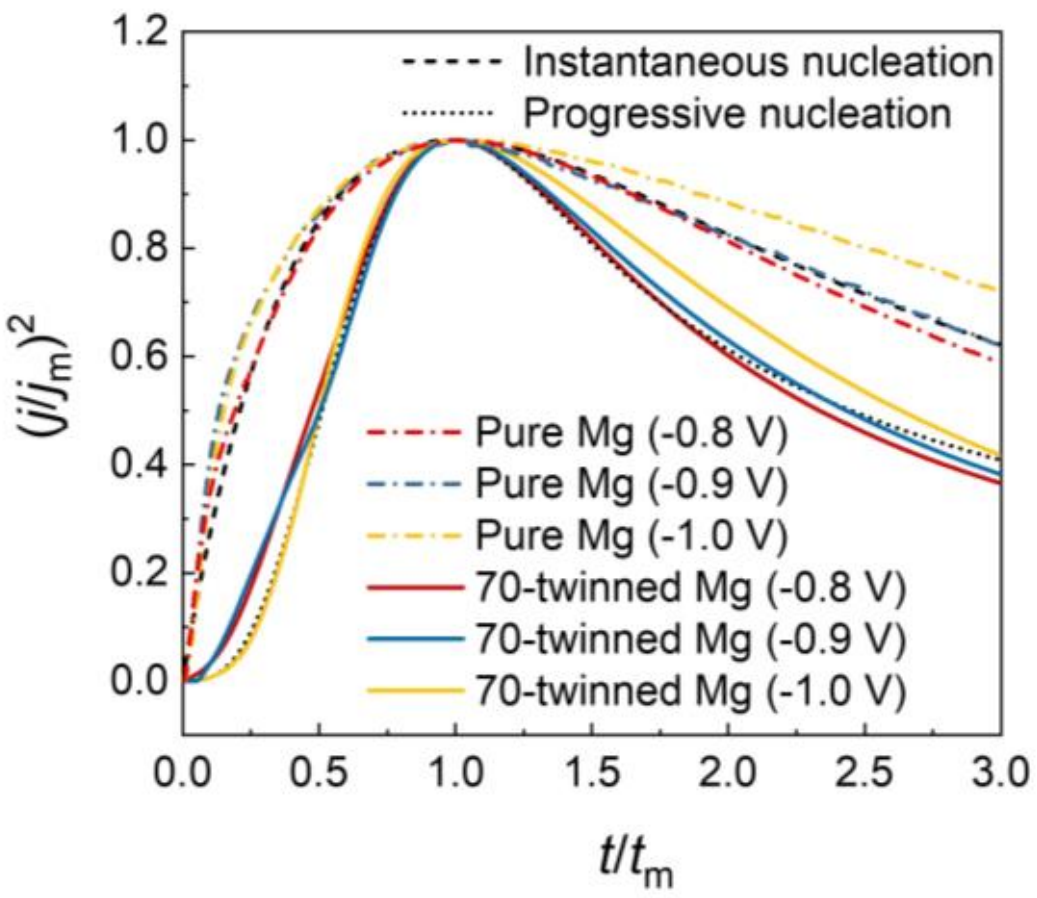


**Figure R31** Plots of $t/t_m$ and $(j/j_m)^2$ in comparison with theoretical 3D models for a Mg cell using Mg and 70-twinned Mg electrodes.

***We have made the following modifications to the manuscript:***

*The electrocrystallisation of new phases is strongly controlled by overpotential and can be directly tracked through current transients. Hence, chronoamperometry (CA) was employed to investigate the early-stage nucleation kinetics of Mg deposition (**Supplementary Fig. S21**). The results indicate that all measured transients reveal a distinct peak current ($j_m$) that is characteristic of a 3D nucleation process. To better assess the nucleation mechanism, the CA transients obtained at closely spaced overpotentials (-0.8, -0.9, and -1.0 V vs. $Mg/Mg^{2+}$) were normalized with respect to the peak current density ($j_m$) and the corresponding peak time ($t_m$), and subsequently compared with the theoretical predictions of the Scharifker–Hills (S–H) model (**Supplementary Note 5**). Notably, prior to mechanistic interpretation, the applicability of the S–H model was explicitly verified by evaluating the diagnostic parameter $(j_{max})^2*t_{max}$ across the different overpotentials. For both pure Mg and 70-twinned Mg electrodes, $(j_{max})^2*t_{max}$ remains within a narrow and consistent range, confirming that the experimental conditions satisfy the diffusion-controlled nucleation assumption inherent to the S–H model (**Supplementary Table S4**). Subsequently, according to this model, nucleation can proceed in two modes: instantaneous (3DI), in which all nuclei form at the onset, or progressive (3DP), in which nuclei gradually form. Based on the normalized transients, Mg deposition on pure Mg is predominantly described by the 3DI nucleation mode (**Fig. 4j**). It should be emphasized, however, that the S–H model is specifically intended to describe early-stage nucleation kinetics and does not directly capture long-term growth processes or predict the final deposition morphology.* {Page 12, Line 9}

*3) The statement that 3DI nucleation enables "uncontrolled dendritic growth" has no literature reference and besides that, it is a bold and baseless statement since the model itself does not describe the later stages of growth, but only earlier initial times, which may or may have not any correlation with the final bulk deposits.*

**Response:** We sincerely thank you for your important comment. We agree that the Scharifker–Hills model, including the 3DI nucleation, is limited to describing early-stage nucleation kinetics and does not directly explain and account for subsequent growth stages or the final deposition morphology. Therefore, we fully acknowledge that our statement in the original manuscript—attributing "uncontrolled dendritic growth" directly to 3DI nucleation based solely on the S–H model—was insufficiently. However, under conditions of highly localized nucleation and limited surface diffusion, the early-stage nucleation behavior described by the 3DI model may be indirectly correlated with non-uniform growth tendencies observed at later stages, which has been reported in several articles. [*Nat. Commun. 2023, 14: 7080; Adv. Mater. 2024, 36: 2406257; Adv. Funct. Mater., 2026: e27860; Adv. Mater. 2026, 38: e16427*].

To strengthen our manuscript and rigorously address your concerns, we have made corresponding revisions. First, we have removed the statement that directly links 3DI nucleation to "uncontrolled dendritic growth." The revised text now explicitly clarifies that the S–H model is employed exclusively to analyze initial nucleation behavior. Second, we have clarified the relationship between the early nucleation process described by the 3DI model and the later-stage growth processes. Specifically, 3DI nucleation causes nearly all available active sites to be rapidly activated at the onset of deposition, resulting in a fixed nucleation density. Subsequent metal deposition must therefore proceed through isolated three-dimensional growth on a limited number of nuclei [*Nat. Commun. 2023, 14: 7080; Batteries Supercaps 2024, 7: e202400250*]. Because no additional nucleation sites emerge to redistribute current, the local current density and ionic flux progressively concentrate at these existing nuclei. This localisation effect is further intensified by ion depletion gradients and limited surface diffusion, which together promote current focusing and amplify morphological perturbations during growth. In contrast, in the 3DP nucleation regime, new nuclei continuously form throughout deposition, dynamically redistributing ionic flux and current density, thereby favouring spatially uniform growth and smoother, denser deposits. Subsequent metal deposition must therefore proceed through isolated three-dimensional growth on a limited number of nuclei [*Nat. Commun. 2023, 14: 7080*].

Moreover, based on the SEM morphological analysis results and the actual characteristics of rapid depletion of active sites and localized high ion flux in localized regions under the 3DI nucleation model, we conducted further validation and analysis using supplementary COMSOL simulations (**Figrue R32**). These simulations demonstrate that 3DI-like nucleation characteristics (particularly the rapid depletion of active sites and ion concentration in localized regions) can amplify local inhomogeneities. When combined with limited surface diffusion, such inhomogeneities increase the probability of morphological instability during subsequent growth, ultimately evolving into dendritic structures. This phenomenon aligns with mechanisms widely reported in the literature and is not solely

inferred from the S-H model. [*Nat. Commun. 2023, 14: 7080; Adv. Mater. 2022, 34: 2203783; Adv. Mater. 2026, 38: e16427*].

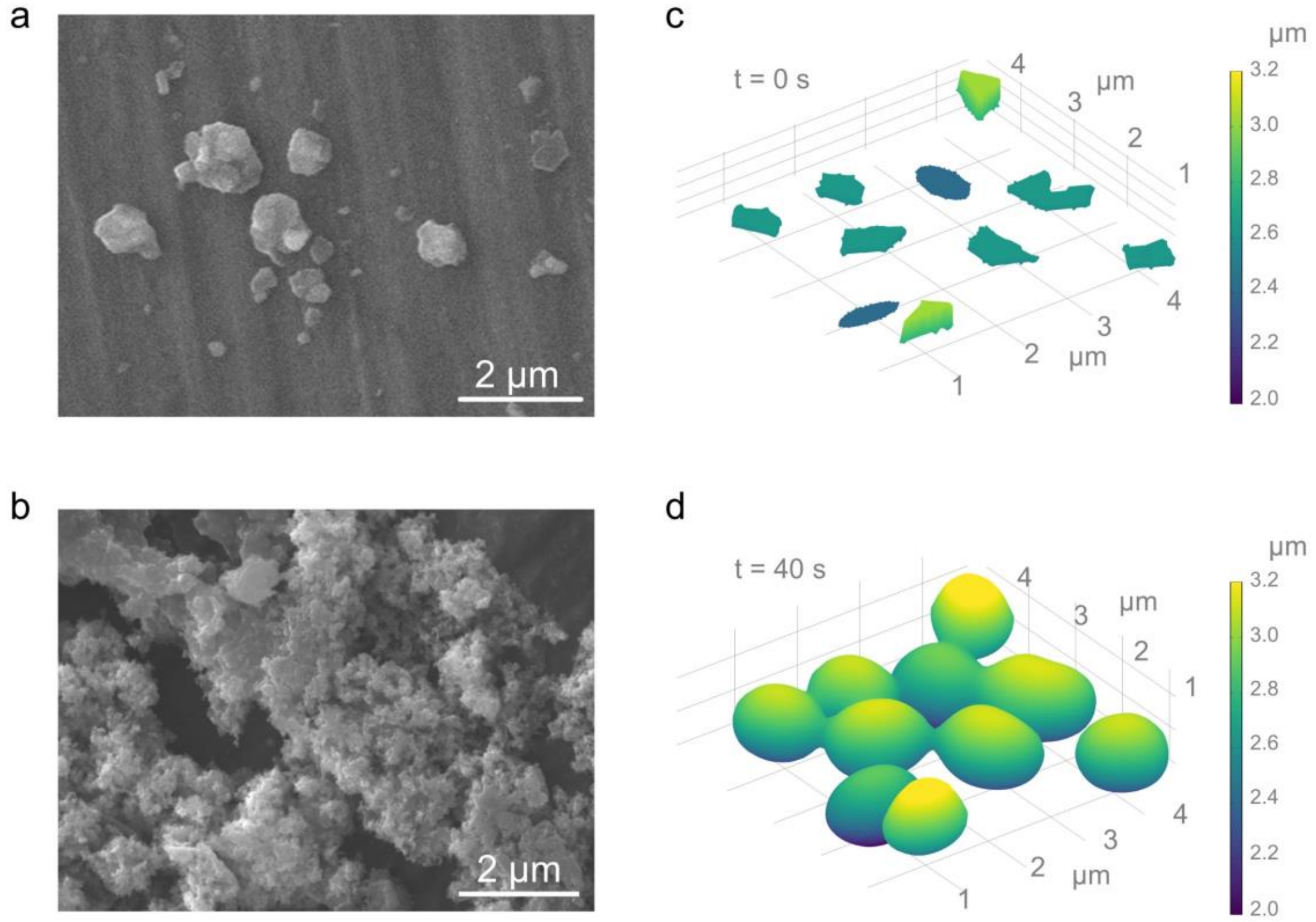


**Figure R32 a, b** Mg deposition on pure Mg anode at 0.1 mAh $cm^{-2}$ (a), 2 mAh $cm^{-2}$ (b). **c, d** Simulated morphology at 0 s (c) and 40 s (d) under the condition of non-uniform nucleation distribution (representing pure Mg).

***We have made the following modifications to the manuscript:***

*Based on the normalized transients, Mg deposition on pure Mg is predominantly described by the 3DI nucleation mode (**Fig. 4j**). It should be emphasized, however, that the S–H model is specifically intended to describe early-stage nucleation kinetics and does not directly capture long-term growth processes or predict the final deposition morphology. Nevertheless, early-stage nucleation characteristics can be indirectly linked to subsequent growth behavior. In particular, the 3DI mode observed on pure Mg electrodes implies rapid and highly localized nucleation at a limited number of active sites, which can result in accelerated local ion consumption and rapid depletion of available nucleation sites. COMSOL simulations (**Supplementary Fig. S22**) further demonstrate that such 3DI-like nucleation characteristics, under conditions of limited surface diffusion, these initial inhomogeneities are prone to being magnified during subsequent growth, thereby increasing morphological instability and ultimately promoting the evolution of dendritic structures. In contrast, the normalized transients of the 70-twinned Mg electrode tend to exhibit the 3DP*

*nucleation mode (**Fig. 4j**). This behavior originates from the internal twin structures, whose strong magnesiophilicity supplies a large number of nucleation sites that can be progressively activated. The resulting uniformly distributed nuclei effectively regulate subsequent Mg growth, leading to a more homogeneous deposition process, in good agreement with the experimentally observed smoother and denser Mg layers (**Fig. 2a-c**).* {Page 12, Line 24}

*4) Unfortunately this fundamentally wrong approach to the S-H model has been applied to another paper with similar structure (Damköhler number, S-H model, twinning strategy), published earlier this year in Nature Communications from almost all the same authors. Doi: https://doi.org/10.1038/s41467-025-56957-w.*

**Response:** We sincerely thank you for this critical and thoughtful comment. Although a similar analytical framework was adopted in our previous work and was accepted by the *Nat. Commun.*, your insight has prompted us to carefully re-examine the underlying assumptions and scope of applicability of the S–H model in the context of Mg electrodeposition. In particular, we recognise that, when describing the intricate coupling between ion transport and interfacial reaction kinetics in divalent metal systems (Mg batteries), our earlier interpretation may have been insufficiently rigorous in certain aspects. We therefore regard the present review process as a valuable opportunity to refine the kinetic framework, clarify the physical meaning of the extracted parameters, and establish a more robust and transparent description of Mg deposition dynamics. In the present manuscript, this issue has been carefully corrected: all chronoamperometric measurements used for nucleation analysis have been completely re-performed in a strictly defined three-electrode configuration, and the applicability criteria of the S-H framework have been re-examined. We emphasize that the revised manuscript now applies the S-H model to analyze early nucleation dynamics within the appropriate scope and no longer extends it beyond its theoretical limitations.

In addition, the Da number is a classical dimensionless parameter that quantitatively describes the competition between reaction kinetics and diffusion rate, defined as the ratio of the electrochemical reaction rate ($k_e$) to the surface diffusion rate ($k_d$) [*Adv. Mater.2023, 36: 2306395; ACS Appl. Mater. Interfaces 2018, 10: 26320-26327*]. Its application in electrochemical systems is widely documented, because it directly reflects whether a process is reaction-controlled or diffusion-controlled [*Nat. Synth. 2025, 4: 552-561; Acta Mater. 2024, 276: 120089; ACS Energy Lett. 2019, 4: 375-376*]. In the revised manuscript, we have strengthen the connection between theory and practical experiments to clearly demonstrate the full rationale for introducing the Da number to quantify transport-reaction competition in metal electrodeposition.

More importantly, the present manuscript differs fundamentally from our earlier work in several key respects.

First, unlike our prior study on sodium metal systems, this work focuses specifically on multivalent magnesium batteries and demonstrates a twin-engineering strategy for modifying Mg metal anodes (hexagonal Zn as a proof-of-concept strategy), whereby a high density of twin defects is deliberately introduced to enhance electrochemical performance.

Second, taking into account the intrinsic crystallographic characteristics of hexagonal Mg, we employ a cyclic multidirectional deformation to generate controllable twin density; the twin density also can be systematically tuned through deformation parameters.

Third, in contrast to the high-pressure solid solution followed by Joule-heating treatment used previously, the present study adopts a far simpler and more industrially scalable deformation route, thereby offering clearer prospects for large-scale implementation.

Fourth, beyond kinetic regulation, we reveal a previously unreported mechanism whereby twin structures mitigate interfacial passivation on Mg metal. This is a critical distinction from sodium systems: whereas Na anodes typically form ionically conductive SEI layers, Mg anodes suffer from dense, poorly conductive passivation films that severely limit performance. Our results demonstrate that twin engineering simultaneously promotes uniform Mg nucleation and suppresses passivation-layer thickening, thereby addressing two fundamental bottlenecks in Mg batteries.

Fifth, we further establish a direct structure–kinetics correlation that was not achieved in our previous work. Specifically, four Mg single-crystal surfaces with well-defined orientations were prepared, and the experimentally observed deposition behaviour on each surface was quantitatively correlated with the corresponding Damköhler numbers. This one-to-one comparison provides particularly strong validation of the reliability of Da analysis in capturing crystallographic anisotropy in deposition kinetics.

Finally, in direct response to your concerns, we have substantially revised the experimental methodology and kinetic analysis. All chronoamperometry measurements have been reperformed according to a strictly defined three-electrode configuration to ensure the accuracy of the results. We have also re-evaluated the diagnostic parameter required for valid application of the S–H model and incorporated finite-element simulations to explicitly bridge early-stage nucleation behaviour with subsequent growth evolution.

---------------------------------------------------

**Reply to the report of referee #4**

---------------------------------------------------

**Comment of Referee #4:**

Reviewer 4

*This manuscript reports a deformation-induced twin-engineering strategy for magnesium metal anodes that enables highly reversible Mg electrodeposition under high current density and areal capacity. By introducing a high density of coherent ($10\bar{1}2$) twins, the authors convincingly demonstrate enhanced Mg surface diffusion, suppressed parasitic interfacial reactions, and excellent long-term cycling stability. The work is original and mechanistically well grounded, relying on intrinsic crystallographic defect engineering rather than coatings, alloying, or electrolyte modification. The link between twin boundaries, reduced diffusion barriers, favorable Damköhler numbers, and uniform planar deposition is well supported by a combination of DFT calculations, electrochemical analysis, and multiscale structural characterization.*

*The experimental evidence is particularly strong, with complementary use of EBSD, TEM, in situ optical microscopy, and three-dimensional X-ray CT providing a coherent and convincing picture of deposition morphology and interfacial stability. The electrochemical performance, including high Coulombic efficiency over thousands of cycles at high current density and areal capacity, clearly exceeds the current state of the art for Mg metal anodes, and the full-cell validation further supports the practical relevance of the approach. Overall, the manuscript is well-suited for publication in Nature Communications.*

**Response:** We appreciate your valuable comments, and we hope the revised version has addressed all your concerns.

*1) The manuscript states that the twin structure shifts Mg deposition from diffusion-controlled to reaction-controlled. While the Damköhler analysis supports a relative shift, diffusion remains important in Mg electrodeposition. The wording could be softened to indicate that deposition becomes less diffusion-limited rather than strictly reaction-controlled.*

**Response:** We thank the reviewer for raising this highly professional and critical comment. We fully agree with the reviewer's assessment that, even when the diffusion kinetics are substantially optimized by the presence of twin structures, Mg deposition should not be strictly described as "fully reaction-controlled." The original wording, "from diffusion-controlled to reaction-controlled," may indeed convey an overly absolute interpretation. Accordingly, we have revised the relevant descriptions in line with the reviewer's suggestion.

Specifically, the introduction of a high density of twin boundaries markedly enhances the interfacial Mg diffusion rate, thereby substantially mitigating diffusion-related

limitations. The Damköhler number analysis reflects the ratio of the electrochemical reaction rate (*k*e) to the surface diffusion rate (*k*d). For the 70-twinned Mg electrode, indicating that Mg deposition that becomes less diffusion-limited and transitions toward a regime in which reaction kinetics play a more prominent role.

***We have made the following modifications to the manuscript:***

*Comprehensive morphological characterisation combined with theoretical calculations reveals that the twinned Mg anode exhibits significantly enhanced kinetics, leading to a reduced dimensionless electrochemical Damköhler number and indicating that Mg deposition becomes less diffusion-limited.* {Page 2, Line 6}

*Notably, we further obtain four Mg single-crystal surfaces with well-defined orientations and correlate the observed Mg deposition behaviors on these surfaces with the corresponding Da number results. This one-to-one comparison more clearly validates the reliability of the Da analysis in describing deposition kinetic differences among different crystallographic planes (**Supplementary Fig. S19 and Fig. S20 and Supplementary Note 4**). In contrast, the presence of twinned structures in twinned Mg substantially alleviates diffusion limitations, resulting Da values much smaller than 1 and thereby promoting uniform, planar Mg deposition (**Fig. 4i and Supplementary Note 4**).* {Page 12, Line 1}

*Moreover, complementary theoretical calculations reveal that the magnesiumphilic twin structure increases the diffusion rate, resulting in Mg deposition that becomes progressively less diffusion-limited and gradually transitions toward a regime in which reaction kinetics play a more prominent role.* {Page 19, Line 16}

*2) Although long-term electrochemical stability is demonstrated, the structural stability of twin boundaries during extended cycling is not explicitly discussed. A brief comment on whether the twin density is expected to persist during cycling, or post-cycling structural evidence if available, would strengthen the conclusions.*

**Response:** We sincerely thank you for your insightful and constructive comments. In response to the concern regarding the structural stability of twin boundaries during long-term electrochemical cycling, we performed post-cycling TEM characterisation on the 70-twinned Mg electrode to directly examine the retention of twin boundaries after extended electrochemical operation.

Specifically, a high density of twin structure can still be clearly observed within the electrode after 50 cycles at a current density of 10 mA $cm^{-2}$ and an areal capacity of 5 mAh $cm^{-2}$ (**Figure R33**). In addition, selected area electron diffraction (SAED) analysis analysis further confirms that these twin structures remain of the $\{10\bar{1}2\}$ tensile twins (**Figure R33b**), indicating that no evident detwinning or structural degradation of the twin boundaries occurs during repeated stripping/plating processes.

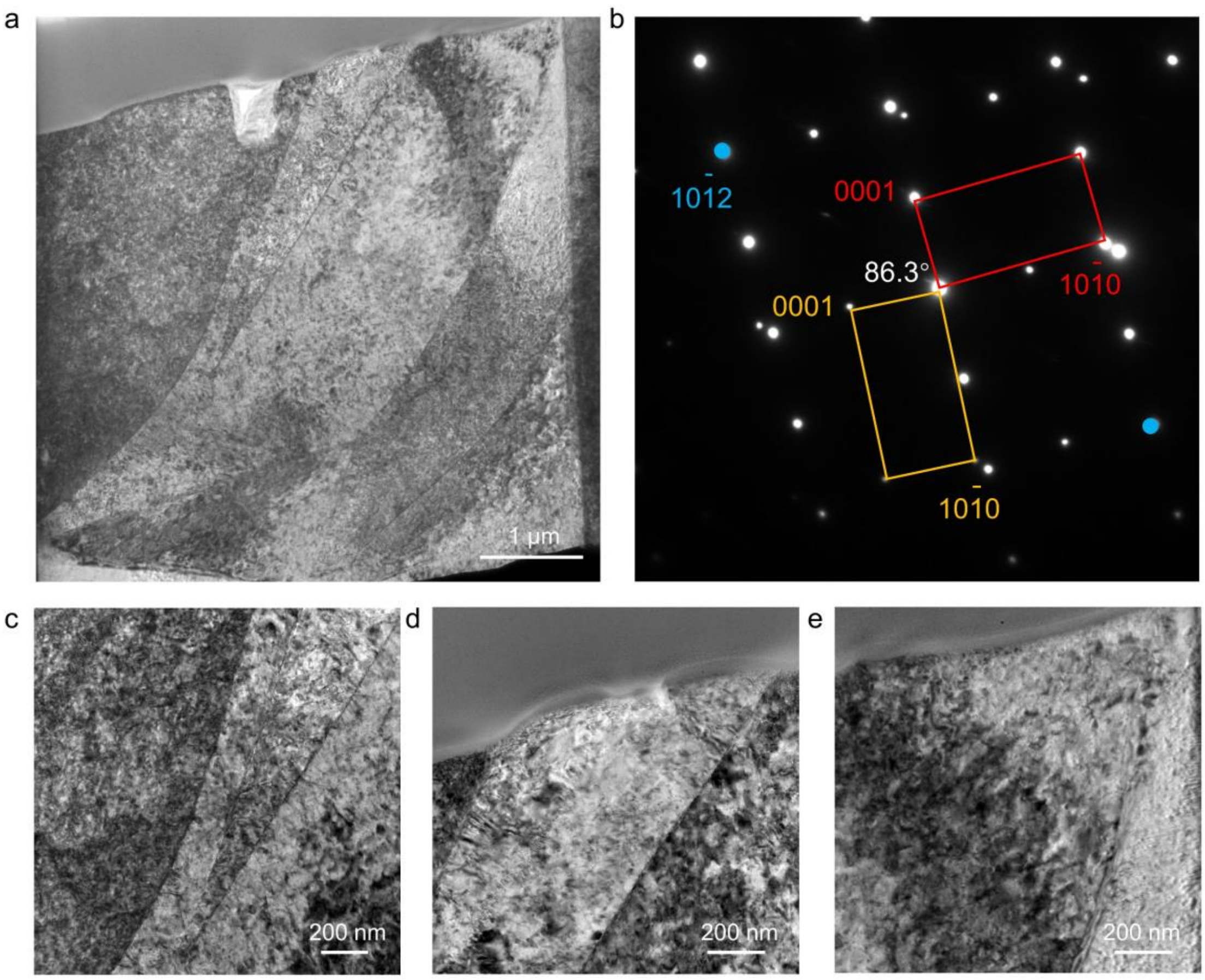


**Figure R33. a-e** TEM image (a) and corresponding SAED image (b) of 70-twinned Mg after cycling (50 cycles, 10 mA cm$^{-2}$, 5 mAh cm$^{-2}$), as well as magnified detail images (c-e).

***We have made the following modifications to the manuscript:***

*In addition, the 70-twinned Mg electrode still retains a dense distribution of twinned structure after 50 cycles at a current density of 10 mA cm$^{-2}$ and a capacity of 5 mAh cm$^{-2}$ (**Supplementary Fig. S13**).* {Page 10, Line 1}

*3) All electrochemical data are obtained in APC-type halide electrolytes. A brief discussion on the expected applicability and possible limitations of the twin-engineering strategy in other Mg electrolyte systems would enhance its generality.*

**Response:** We sincerely thank you for your insightful and profound comment. In response to your reasonable concern that the observed uniform Mg deposition might originate from the intrinsic chemistry of the APC electrolyte, we further investigated three representative chloride-free Mg electrolytes and carried out a systematic comparative analysis of deposition morphology and electrochemical performance.

Specifically, in all three chloride-free electrolyte systems, the 70-twinned Mg electrode consistently exhibits markedly more uniform deposition than the pure Mg electrode. The

resulting surface morphology is smoother and denser, showing trends highly consistent with those observed in the APC electrolyte (**Figure R34**). Meanwhile, symmetric cells assembled with 70-twinned Mg under identical electrolyte conditions display clearly superior cycling stability during variable-rate cycling compared with those based on pure Mg electrodes (**Figure R35**).

In summary, these morphological characterisations and electrochemical measurements indicate that the improvement in Mg deposition behavior observed in this manuscript primarily arises from the intrinsic regulatory effect of the twin structure within the Mg electrode, rather than from the specific chemical characteristics of the APC electrolyte.

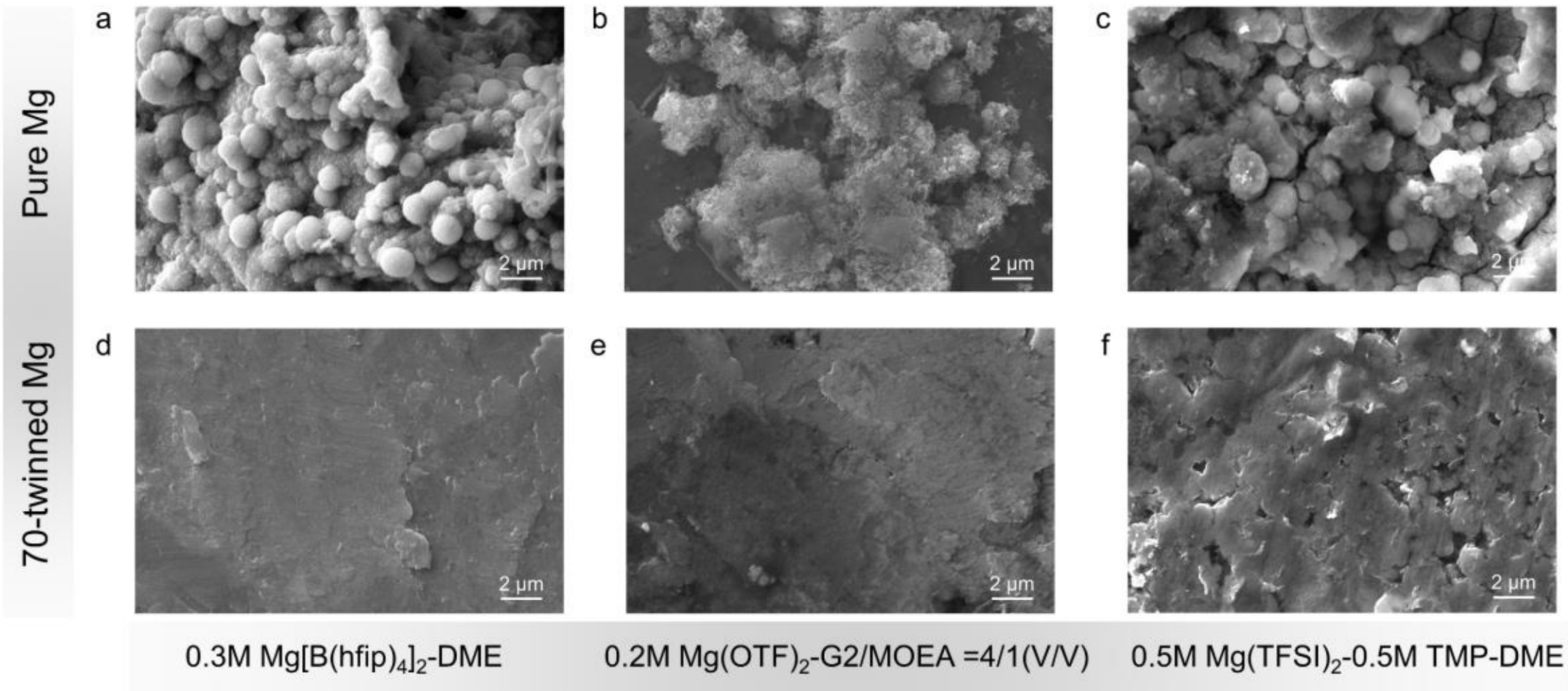


**Figure R34.** Mg deposition morphology on pure Mg (a-c) and 70-twinned Mg (d-f) surfaces under 10 mA $cm^{-2}$, with 5 mAh $cm^{-2}$ in different electrolyte. ($Mg[B(hfip)_4]_2$: Magnesium tetrakis(hexafluoroisopropyloxy)borate; DME: 1-2 dimethoxyethane; $Mg(OTF)_2$: Magnesium trifluoromethanesulphonate; G2: Diglyme; MOEA: 2-Methoxyethylamine; $Mg(TFSI)_2$: Magnesium bis(trifluoromethylsulfonyl)imide; TMP: Trimethyl phosphate)

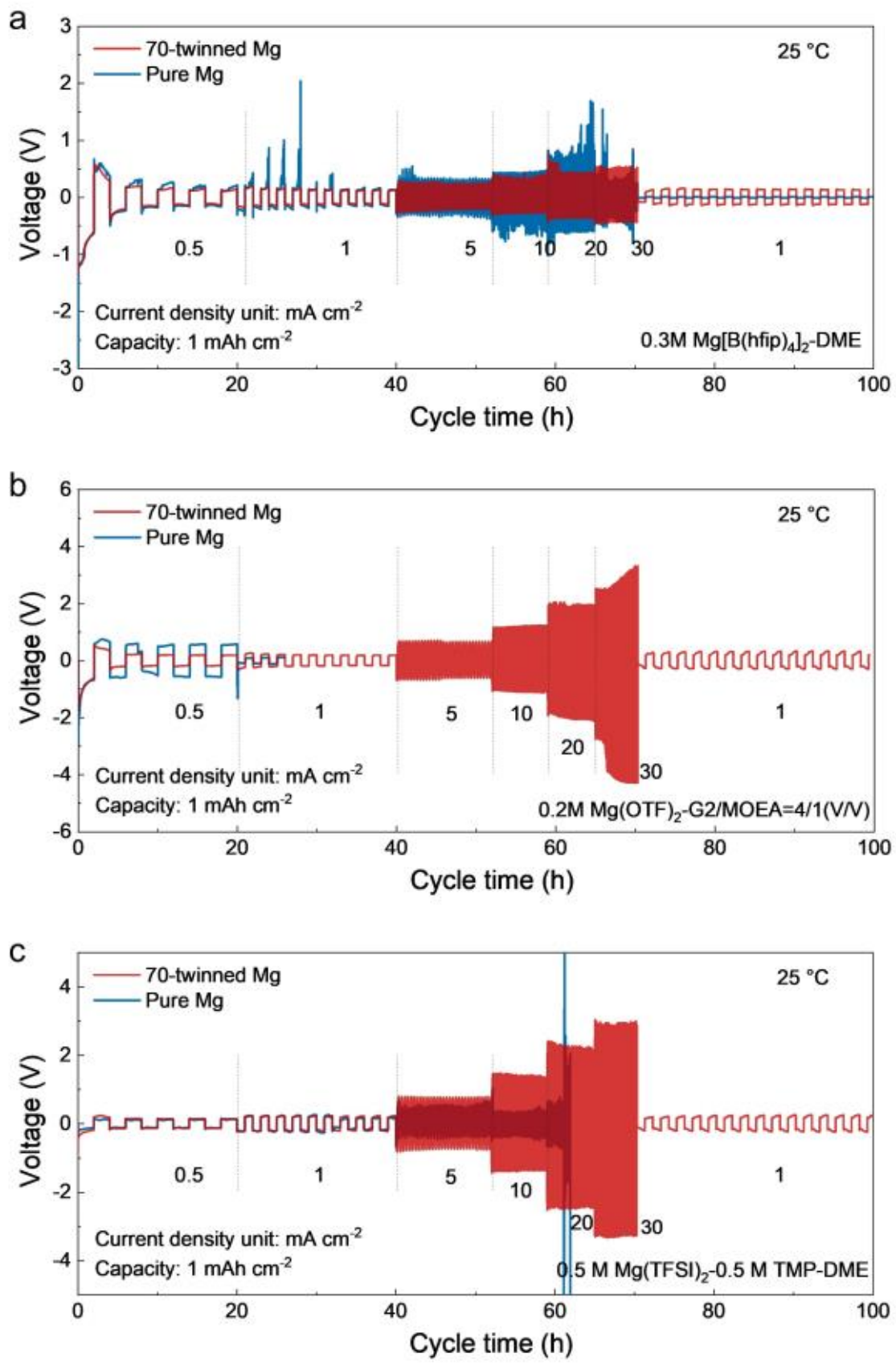


**Figure R35. a-c** Rate performance of the pure Mg and 70-twinned Mg symmetric cells in different electrolyte at various current densities with the same capacity of 1 mAh cm$^{-2}$.

***We have made the following modifications to the manuscript:***

*Notably, in the other three chloride-free electrolyte systems, the 70-twinned Mg electrode consistently exhibits markedly more uniform deposition than the pure Mg electrode (**Supplementary Fig. S8**). The resulting surface morphology is smoother and denser, showing trends highly consistent with those observed in the APC electrolyte. Therefore, the enhanced Mg deposition behavior primarily originates from the intrinsic regulatory effect of the twin structure within the Mg electrode, rather than from the specific chemical characteristics of the APC electrolyte.* {Page 7, Line 23}

*Moreover, the excellent rate performance of the 70-twinned Mg||70-twinned Mg symmetric cells can also be maintained in the other three chlorine-free electrolytes (**Supplementary Fig. S15**).* {Page 10, Line 8}.

*4) The extension of the strategy to Zn metal supports the generality of the concept, but the mechanistic analysis is less detailed than for Mg. It would be appropriate to explicitly frame the Zn results as a proof-of-concept validation rather than a fully equivalent mechanistic study.*

**Response:** We sincerely appreciate the reviewer's constructive comment. Therefore, in the revised manuscript, we have explicitly framed the zinc-related results as a proof-of-concept validation, rather than as a fully equivalent mechanistic study. In addition, we have added XPS analysis to further clarify the role of twin structures in suppressing byproduct accumulation in zinc batteries (**Figure R36**).

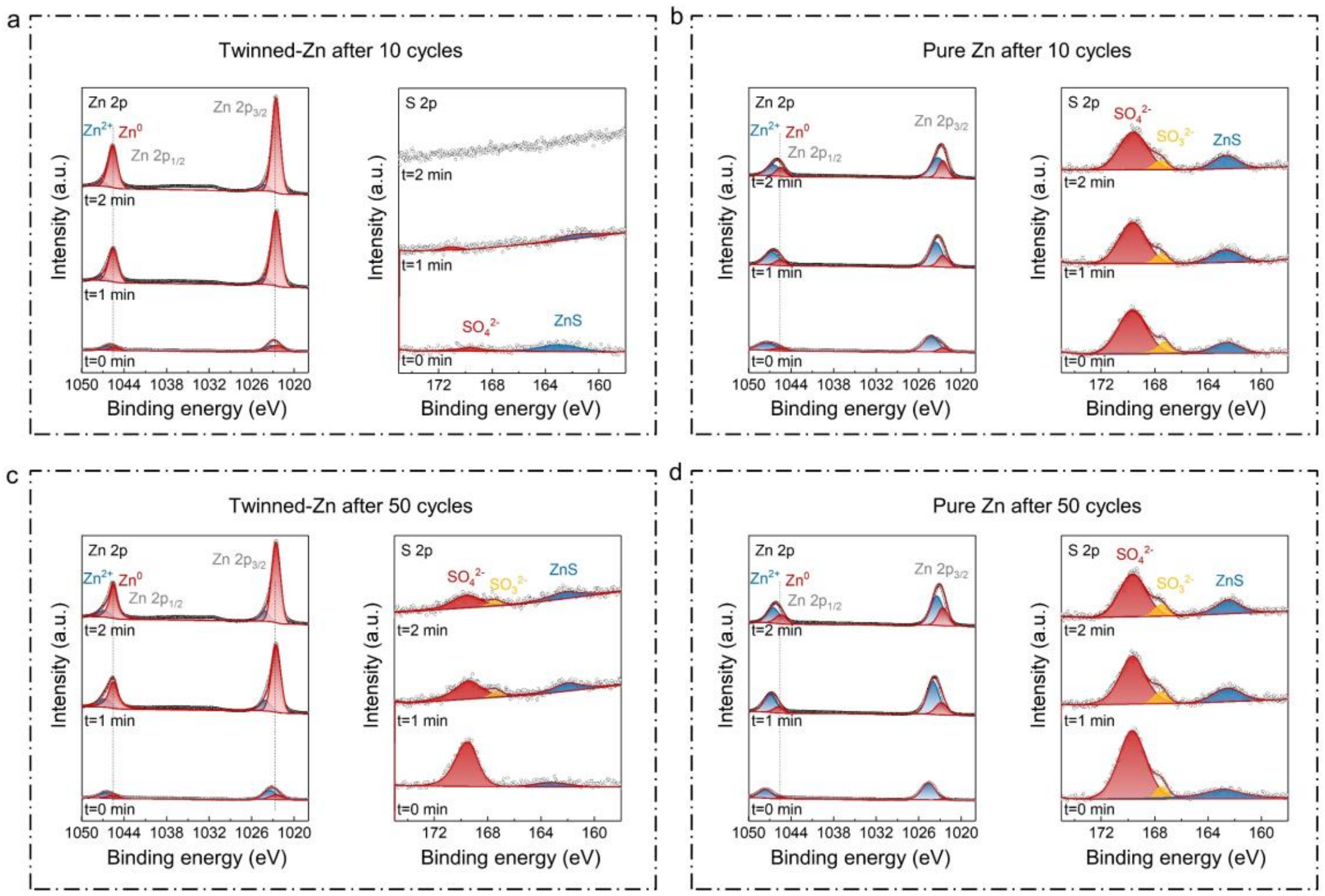


**Figure R36 a, b** High-resolution XPS spectra of Zn 2*p* and S 2*p* of twinned Zn (a) and pure Zn (b) after 10 cycling. **c, d** High-resolution XPS spectra of Zn 2*p* and S 2*p* of twinned Zn (c) and pure Zn (d) after 50 cycling.

***We have made the following modifications to the manuscript:***

*This simple strategy has been successfully extended to the zinc metal system as a proof of concept, demonstrating the universal potential of twin engineering for achieving highly reversible metal electrodeposition.* {Page 2, Line 15}

*Furthermore, the successful extension of this twin-engineering strategy to Zn metal serves as a proof-of-concept validation, underscoring its potential generality for designing stable metallic anodes.* {Page 5, Line 16}

*To verify the universality of the twinning-defect-induced highly reversible electrodeposition strategy, we further extended this concept from Mg to Zn metal systems, building upon the successful application of twinned-Mg in significantly enhancing the electrochemical performance of Mg metal batteries. The primary purpose of the Zn study is to serve as a proof-of-concept, aiming to examine whether core strategies derived from the*

*Mg system can be transferred to other metal systems. The choice of Zn as a proof-of-concept system carries important crystallographic and electrochemical implications.…….. Electrochemical testing revealed that the symmetric cells assembled with the twinned-Zn electrodes achieved stable cycling under high current densities without any observable Zn dendrite formation (**Supplementary Fig. S37 and Fig. S38**), while also exhibiting reduced accumulation of by-products (**Supplementary Fig. S39**). These results demonstrate that the twin structures play a continuous regulatory and stabilising role during dynamic metal deposition, consistent with the trends observed in the Mg system, thereby providing experimental support for the generality of the proposed strategy. A more in-depth mechanistic elucidation of Zn deposition behavior will be the focus of future studies.* {Page 18, Line 13}

*While this work focuses on an in-depth mechanistic investigation of the Mg system, the successful preliminary extension of the strategy to Zn metal provides an important proof-of-concept for its general applicability and opens a promising pathway toward the development of dendrite-free metal batteries.* {Page 19, Line 15}

-------------------------------------------------------

**Reply to the report of referee #1**

------------------------------------------------------

**Comment of Referee #1:**

Reviewer 1

*The author have well addressed my comments. Therefore, I recommend the publication of this work in Nature Communications.*

**Response:** We appreciate the reviewer's recommendation for publishing our manuscript on Nature Communications.

---------------------------------------------------

**Reply to the report of referee #2**

---------------------------------------------------

**Comment of Referee #2:**

Reviewer 2

*I appreciate that the authors have made a substantial effort to address the comments. In particular, the revised manuscript now includes additional experimental characterization (e.g., XPS after extended cycling and in situ EIS monitoring) and theoretical calculations intended to support the proposed mechanism. These additions improve the manuscript and partially address the concerns raised in the previous review.*

*However, after carefully examining the revised version and the response to reviewers, I believe that several key issues remain insufficiently resolved. Therefore, I recommend major revision before the manuscript can be considered for publication.:*

*1) The evidence supporting the claim of a "nearly bare-metal interface" remains largely indirect. The authors should either provide more direct structural evidence for this claim or moderate the wording accordingly.*

**Response:** Thank you for your careful evaluation and helpful suggestion. We agree that the previous wording "nearly bare-metal interface" was not well supported by direct evidence and could be misleading. Therefore, we have revised the manuscript accordingly. The term "nearly bare-metal interface" has been replaced with "significantly weakened passivation effect" to better match the experimental results and avoid overstatement. Our data consistently show that the interfacial layer is reduced but not removed (**Figure R1**). XPS results indicate a lower amount of insulating species after cycling. In situ EIS shows decreased interfacial resistance, suggesting improved charge transfer. TOF-SIMS and TEM both confirm that the interphase is thinner, but still present. DFT calculations also support this point. They show reduced barriers for $Mg^{2+}$ transport and less tendency for electrolyte decomposition, which explains the thinner interphase. Based on these results, we now clearly state that the passivation layer is weakened rather than eliminated. This wording is more accurate and better supported by the data.

***We have made the following modifications to the manuscript:***

*Furthermore, reorganisation energy analysis based on Marcus–Hush–Chidsey kinetics, in conjunction with the battery overpotential, indicates that the twin structure effectively mitigates parasitic reactions between the anode and the electrolyte, thereby significantly reducing the passivation effect and enabling long-term reversible Mg electrodeposition.* {Page 2, Line 9}

*These observations, in agreement with the results of the XPS analysis, indicate that a significant reduction in interfacial products during electroplating.* {Page 14, Line 16}

*To further confirm that the twinned structure effectively suppresses interfacial passivation,* {Page 14, Line 19}

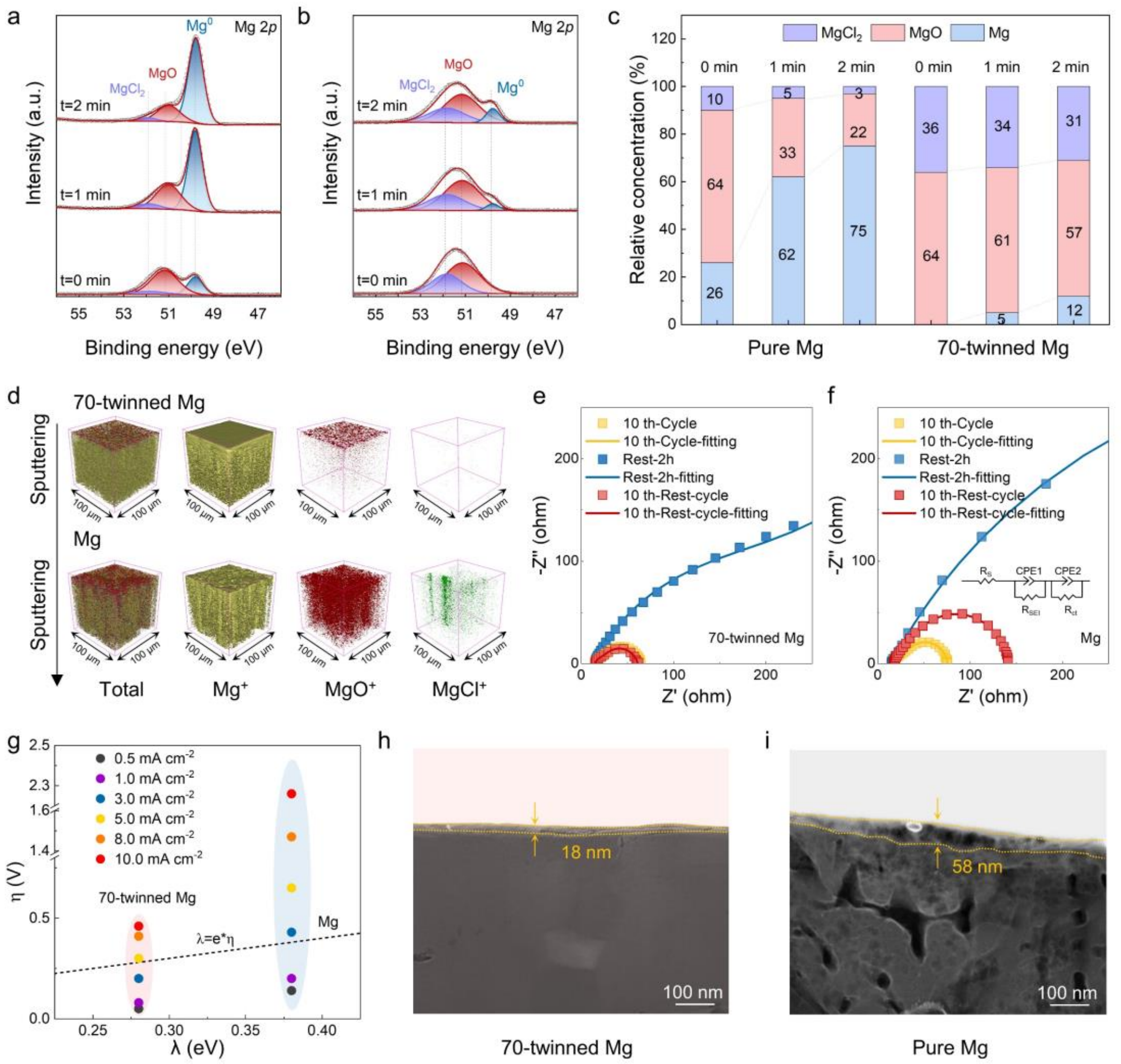


**Figure R1. Interface analysis and characterisation**. **a, b** High-resolution XPS spectra of Mg 2*p* of 70-twinned Mg (a) and pristine Mg (b) electrodes after cycling (10 cycles at 10 mA $cm^{-2}$). **c** The relative concentrations of decomposition species measured on the surface after cycling at sputtering depths of 0, 1 and 2 min. **d** 3D rendering for the $Mg^+$, $MgO^+$, and $MgCl^+$ secondary ions and the overlay in the ToF-SIMS tested for the interfaces formed on the Mg and 70-twinned Mg electrodes. **e, f** The EIS spectra of a 70-twinned Mg||70-twinned Mg (e) and a Mg||Mg cell (f) cell measured after 10 cycling, resting for 2 h, and re-cycling period, under conditions of 10 mA $cm^{-2}$ and 1 mAh $cm^{-2}$. The inset corresponds to the fitted circuit. **g** The correlation between overpotential (η) and reorganization energy (λ) for different reaction modes of the Mg anodes. The dashed line represents when the η for electron transfer is equal to λ. **h, i** TEM images and of the interface and deoposited Mg after cycling for the 70-twinned Mg (h) and pure Mg (i) electrodes.

*2) The revised manuscript introduces DFT calculations describing the reduction of THF leading to MgO formation as a model reaction to explain electrolyte decomposition. However, the relevance of this reaction pathway to the APC electrolyte system is not sufficiently justified.*

**Response:** We sincerely thank you for your insightful and profound comment. As the sole solvent in APC electrolytes, THF may decompose on the surface of metallic Mg at sufficiently negative potentials. Previous studies have shown that ether-based solvents can decompose on reactive metal anodes (e.g., Li and Mg) through ring-opening or dehydrogenation reactions, forming inorganic and organometallic species [*Angew. Chem. Int. Ed. 2022, 61: e202208743; J. Organomet. Chem., 1985, 284: 149-158*].

In this work, we focus on the formation of inorganic passivation products, particularly MgO. Therefore, the reduction of THF to MgO is used as a simplified model reaction to compare the thermodynamic tendency of solvent decomposition on pure Mg and twinned Mg. This reaction pathway is based on a feasible assumption ($C_4H_8O$ + Mg → MgO + $C_4H_8$).

To verify the feasibility of this reaction pathway, we immersed Mg foil in THF and observed that the surface color darkened after soaking, indicating that a surface reaction had occurred (**Figure R2**). Subsequently, we performed XPS characterization on the Mg samples before and after immersion. The results revealed a significantly enhanced MgO signal after soaking, confirming that Mg can react with THF (**Figure R3**). In addition, we conducted differential electrochemical mass spectrometry (DEMS) measurements during the Mg deposition process in Mg symmetric cells using the APC electrolyte. The results showed the evolution of $C_4H_8$ gas during electrochemical process (**Figure R4**), further supporting the possible existence of the proposed reaction pathway.

However, it is worth noting that in practice, the interfacial decomposition of THF can be more complex and may produce oligomers or other organic species, but the free energy of these processes is difficult to evaluate reliably. Therefore, MgO is selected as a representative inorganic passivation product to reflect the relative ability of different electrodes to suppress solvent decomposition. In the revised manuscript, we have clarified the rationale for this model and its limitations, and we emphasize that the purpose of this calculation is to compare the relative difference between twinned Mg and pure Mg, rather than to quantitatively predict the actual decomposition products.

In addition, as you pointed out, chloride chemistry plays a dominant role in the APC electrolyte, and Mg–Cl complexes are key precursors for interfacial products [*Adv. Mater. 2025, 37: e10488; Adv. Mater. 2025, 37: 2417652*]. Compared with THF, $[MgCl\cdot 5THF]^+$ is more electrochemically active and more likely to be reduced on the electrode surface [*J. Phys. Chem. C 2013, 117: 26881-26888*]. Therefore, we have also included calculations of the decomposition of $[MgCl\cdot 5THF]^+$ on different electrode surfaces in the revised

manuscript. In particular, we considered the reduction pathway from $[MgCl\cdot5THF]^+$ to $MgCl_2$, which directly reflects the consumption of active species at the interface, and its by-products are directly involved in the formation of the interfacial passivation layer (**Figure R5, 6**). This model reaction is therefore more directly relevant to the APC system.

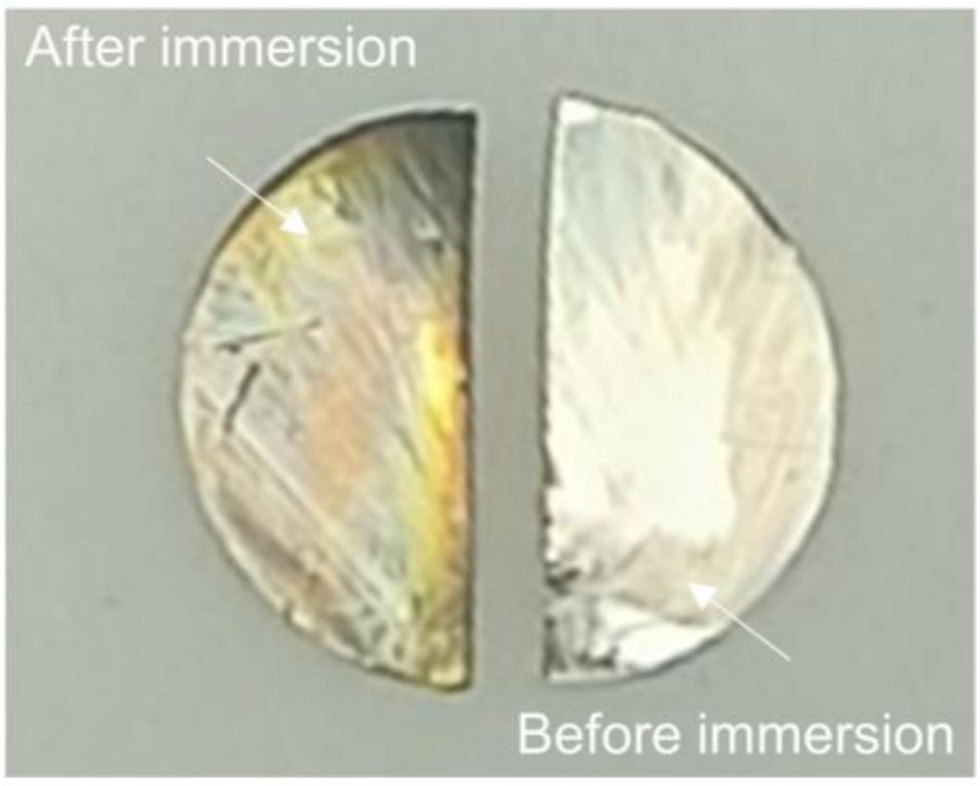


**Figure R2.** Optical images of Mg before and after immersion in THF.

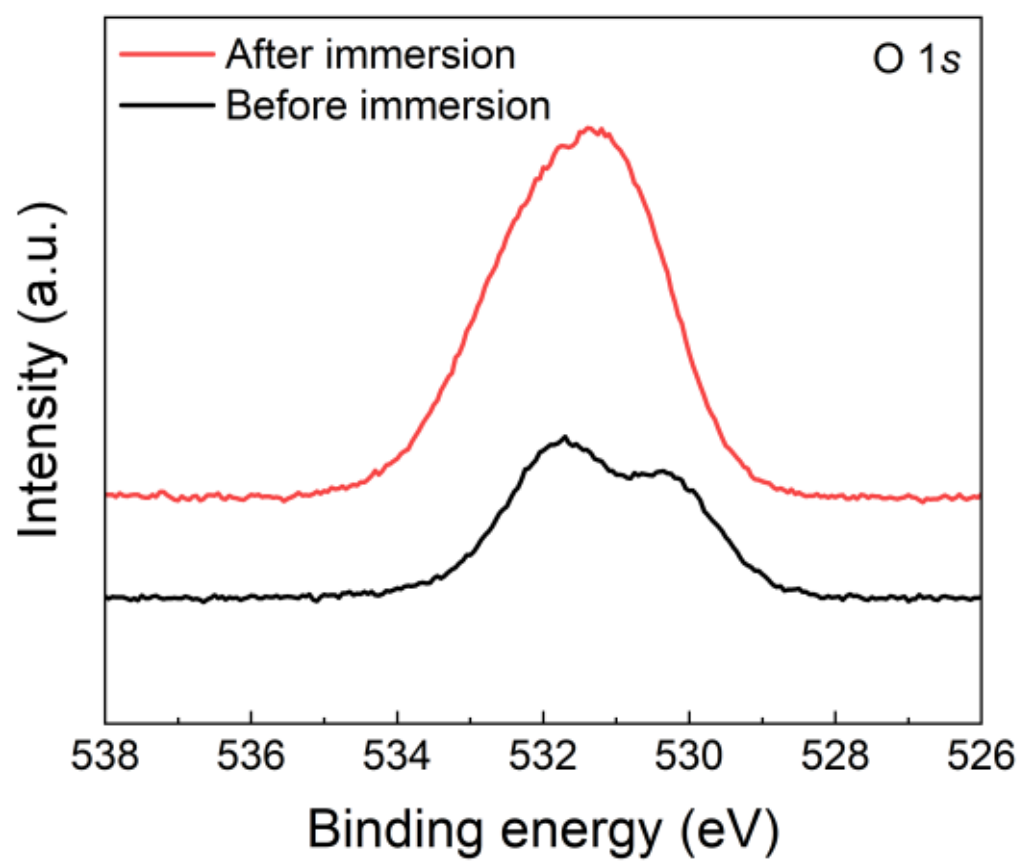


**Figure R3.** XPS analysis of the surface of Mg before and after immersion in THF.

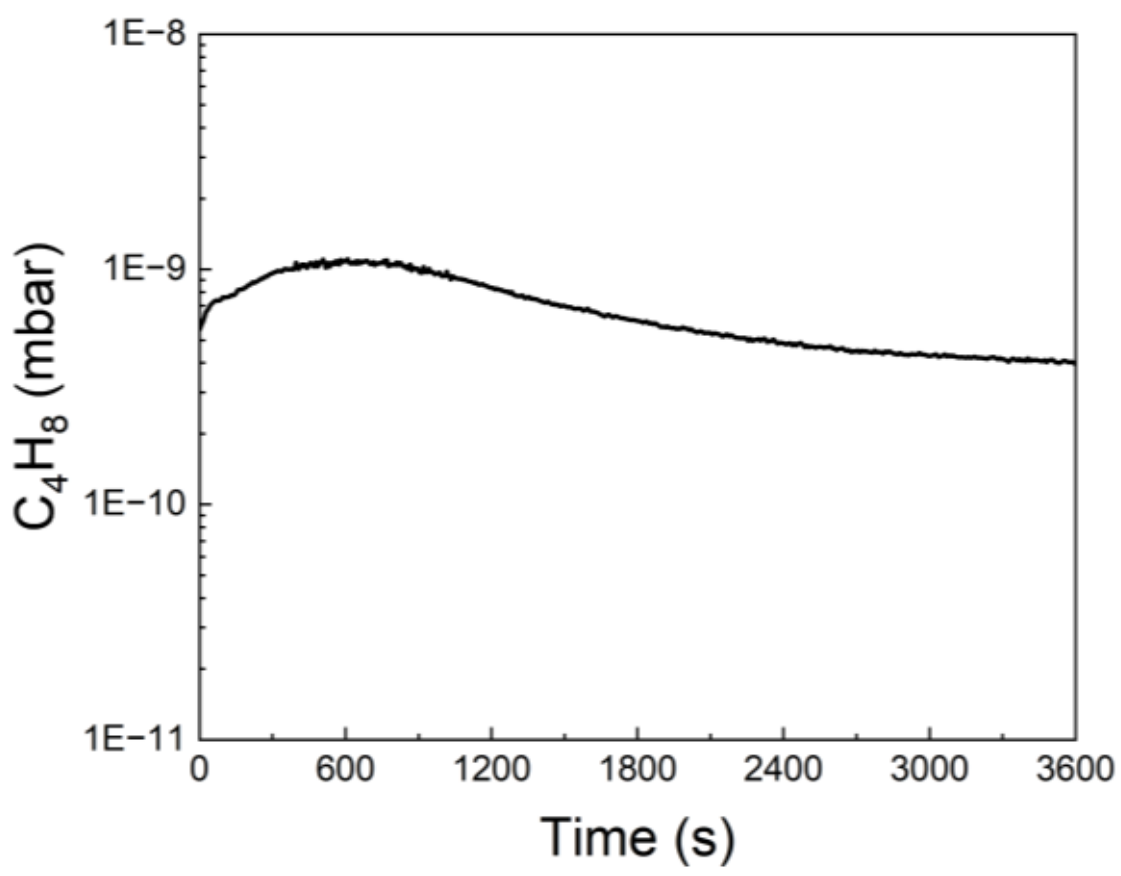


**Figure R4.** The content of $C_4H_8$ in Mg batteries during electrochemical testing.

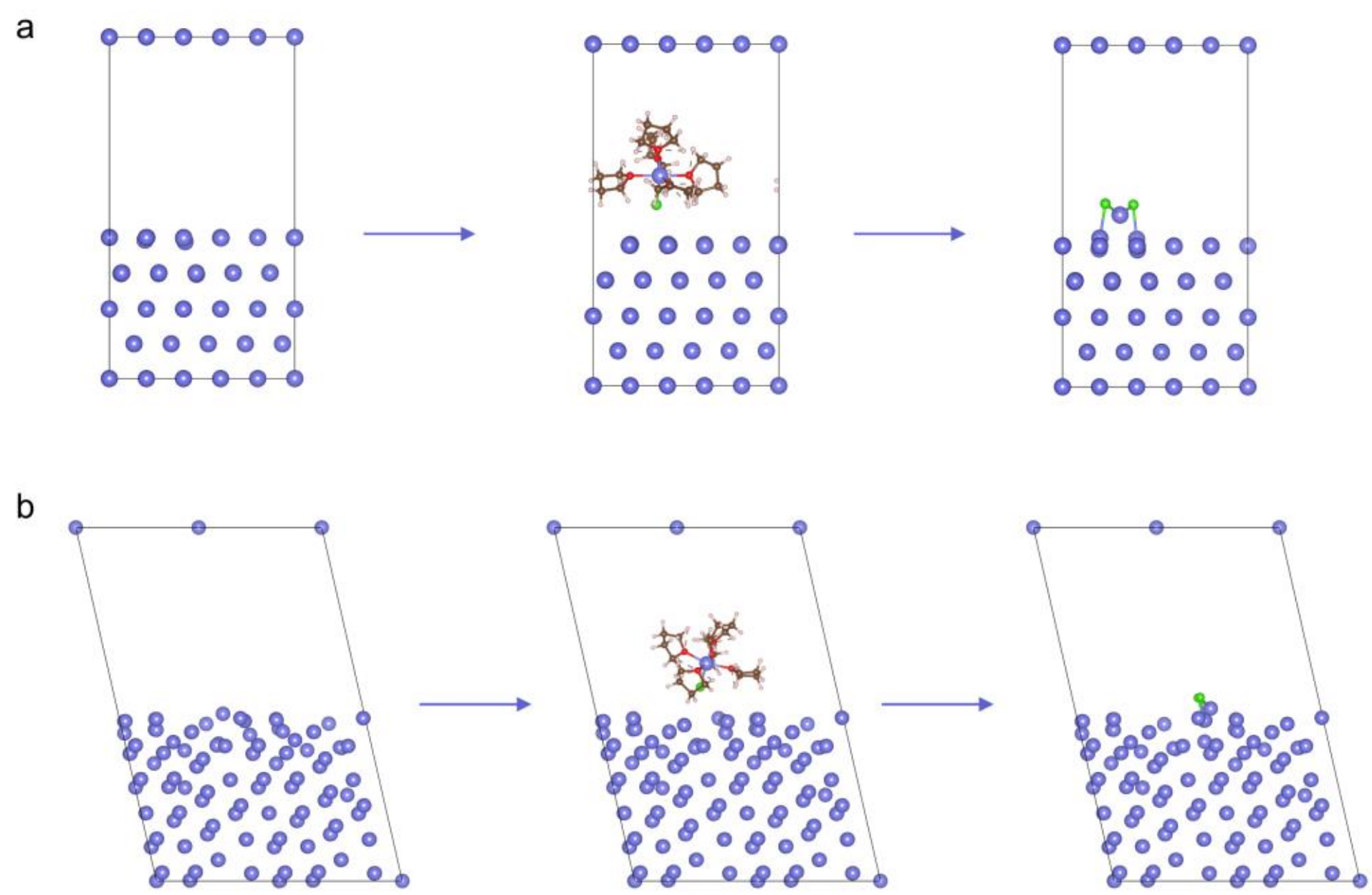


**Figure R5. a, b** Configurational changes before and after adsorption of $[MgCl{\cdot}5THF]^+$ on pure Mg (a) and 70-twinned Mg (b) surfaces. (red, oxygen; green, chlorine; grey, carbon; white, hydrogen; and purple, Mg).

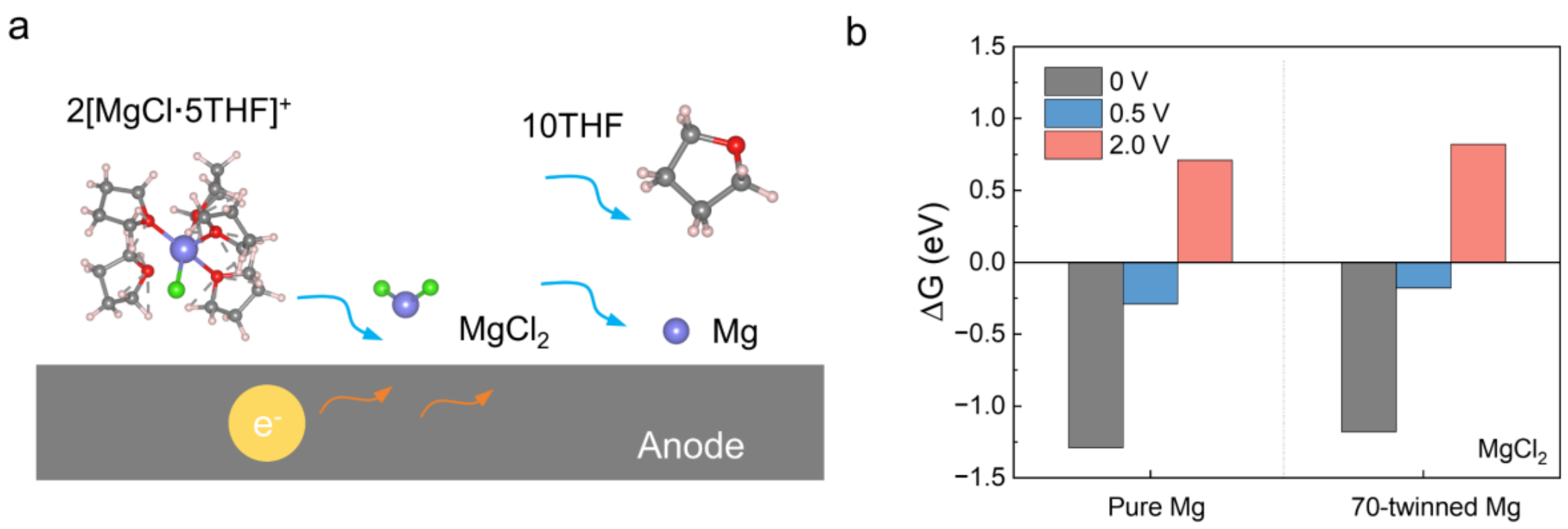


**Figure R6.** Theoretical calculation of the Gibbs free energy change of the reaction forming THF , adsorbed Mg or $MgCl_2$ on different anodes. a) Scheme of the calculation model (red, oxygen; green, chlorine; grey, carbon; white, hydrogen; and purple, Mg). The free energy calculation results. (The reason for choosing potentials of 0.5 V and 2.0 V is that the overpotential of a 70-twinned Mg symmetric cell at 10 mA $cm^{-2}$ is 0.43V (~0.5 V); while the overpotential of a pure Mg symmetric cell at 10 mA $cm^{-2}$ is 2.26V (~2.0 V)).

***We have made the following modifications to the manuscript:***

*Considering that tetrahydrofuran (THF) serves as the solvent in the APC electrolyte and $[MgCl{\cdot}5THF]^+$ species act as its active components, we selected $[MgCl{\cdot}5THF]^+$ and THF as two representative models for our calculations. Based on these models, we systematically compared the reaction thermodynamics on pure Mg and twinned Mg under*

*different electrode potentials (**Supplementary Figs. S29-S32**). At the same electrode potential, the Gibbs free energy for the reduction of [MgCl·5THF]$^+$ and THF on twinned Mg is significantly higher than that on pure Mg ($\Delta G_{twinned\text{-}Mg} > \Delta G_{pure\text{-}Mg}$), indicating that the twin structure intrinsically increases the energy barrier for the formation of interfacial passivation products (MgO and $MgCl_2$). In addition, for the same anode material, increasing the electrode potential markedly increases the Gibbs free energy of the reduction reaction, thereby suppressing interfacial side reactions. Notably, this "potential-interfacial reaction synergistic suppression effect" on twinned Mg provides clear thermodynamic evidence that twin structure is conducive to interfacial passivation suppression.* {Page 15, Line 12}

*3) In APC electrolytes, interfacial reactions are widely understood to be dominated by chloride-based chemistry involving Mg–Cl complexes and the formation of $MgCl_2$-containing interphases, rather than direct solvent decomposition to MgO. Therefore, the THF→MgO model reaction may not adequately represent the dominant decomposition pathway in this system.*

**Response:** We thank you for your insightful comment. We appreciate the point that interfacial reactions in the APC electrolyte are dominated by chloride-based chemistry rather than direct solvent decomposition. In this system, Mg–Cl complexes play a key role in the formation of interfacial products [*Nat. Commun. 2026, 17: 323; J. Phys. Chem. C 2013, 117: 26881-26888*]. To address this, we have added model reactions that directly describe the decomposition of active species in the APC electrolyte on different electrode surfaces.

Specifically, compared with the solvent THF, [MgCl·5THF]$^+$ shows higher electrochemical activity and is more likely to be reduced on the electrode surface. Its decomposition is closely related to the formation of the interfacial passivation layer [*Adv. Mater. 2025, 37: e10488; J. Phys. Chem. C 2013, 117: 26881-26888*]. Therefore, we have calculated the reduction pathway of [MgCl·5THF]$^+$ to $MgCl_2$ in the revised manuscript (**Figure R7, 8**). This reaction reflects the consumption of active species at the interface, and its products directly contribute to the growth of the passivation layer. This model is directly relevant to the APC system.

The calculation results show that, at the same electrode potential, the Gibbs free energy of the [MgCl·5THF]$^+$ reduction reaction on twinned-Mg is significantly higher than that on pure Mg. This indicates that, even for the dominant chlorine-based chemical pathway in the APC system, the twinned structure increases the energy barrier for the passivation reaction, thereby effectively suppressing the growth of the $MgCl_2$-containing interfacial layer.

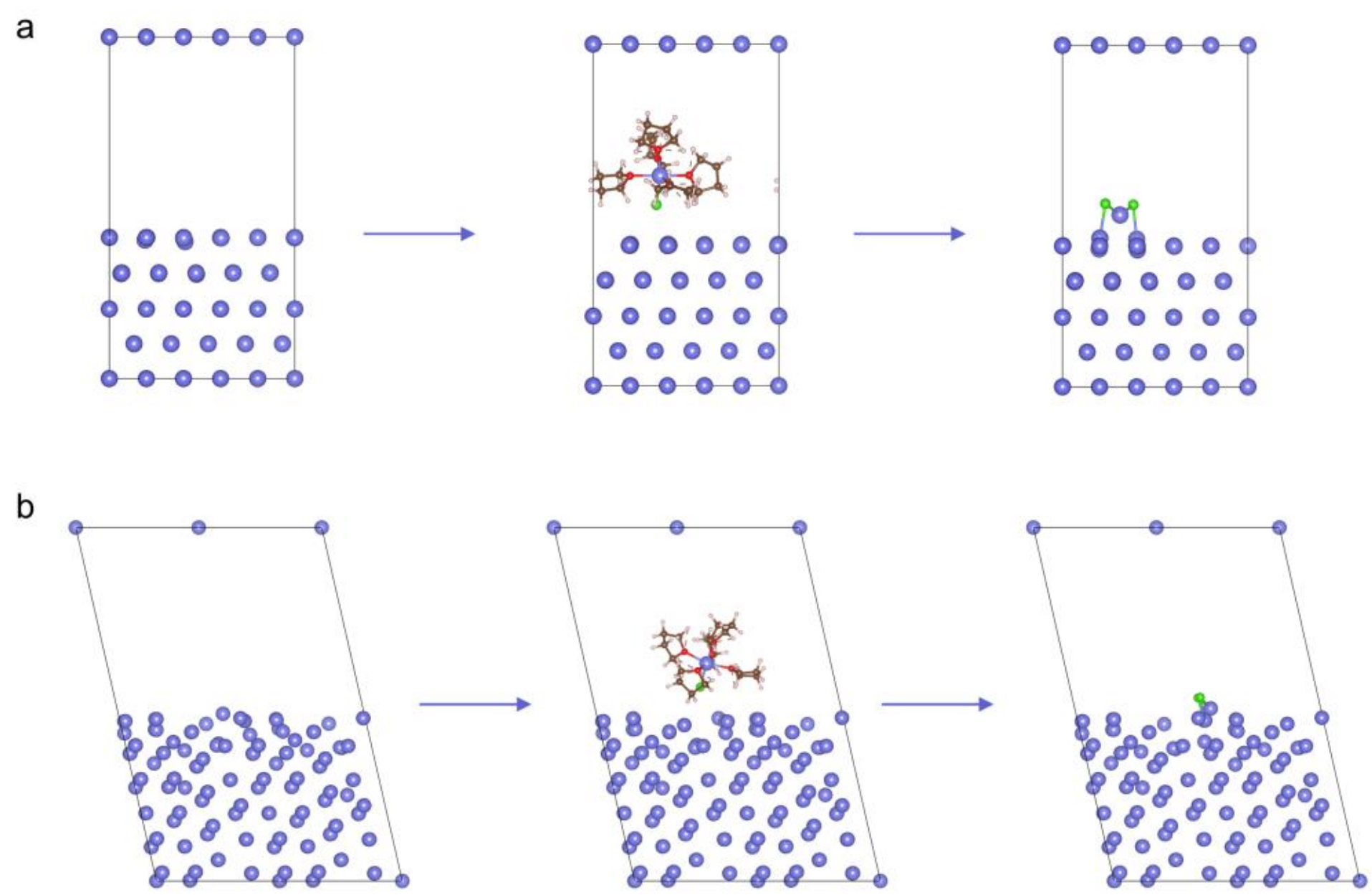


**Figure R7. a, b** Configurational changes before and after adsorption of $[MgCl\cdot5THF]^{+}$ on pure Mg (a) and 70-twinned Mg (b) surfaces. (red, oxygen; green, chlorine; grey, carbon; white, hydrogen; and purple, Mg).

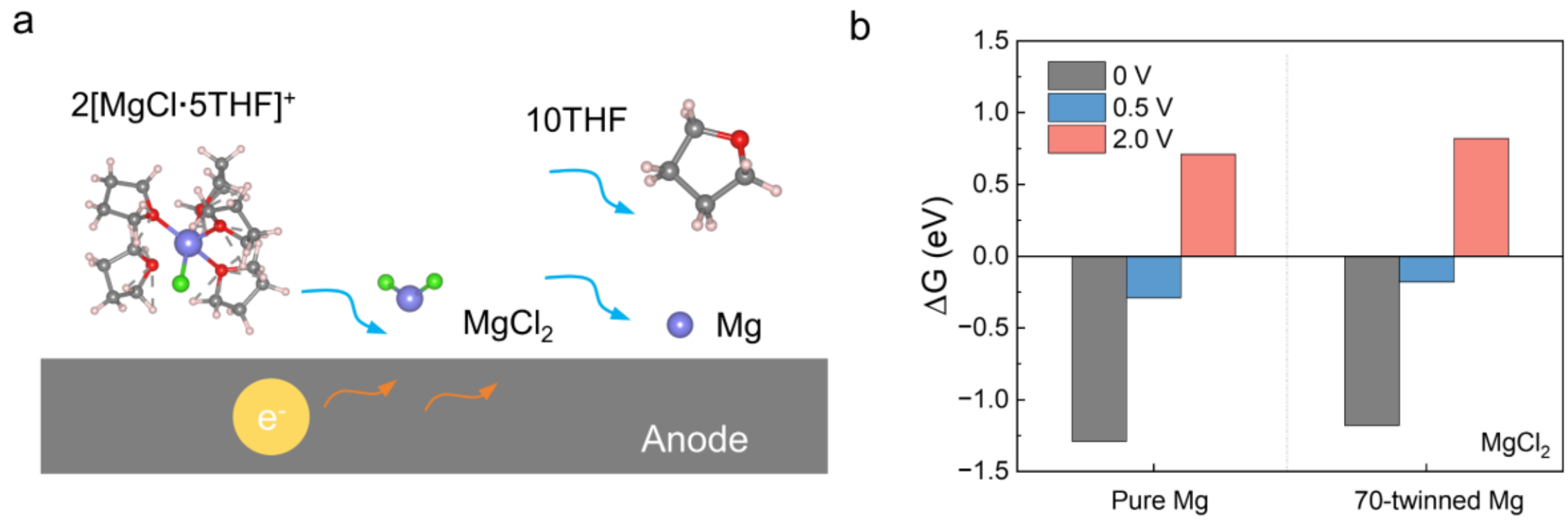


**Figure R8.** Theoretical calculation of the Gibbs free energy change of the reaction forming THF , adsorbed Mg or $MgCl_2$ on different anodes. a) Scheme of the calculation model (red, oxygen; green, chlorine; grey, carbon; white, hydrogen; and purple, Mg). The free energy calculation results. (The reason for choosing potentials of 0.5 V and 2.0 V is that the overpotential of a 70-twinned Mg symmetric cell at 10 mA $cm^{-2}$ is 0.43V (~0.5 V); while the overpotential of a pure Mg symmetric cell at 10 mA $cm^{-2}$ is 2.26V (~2.0 V)).

***We have made the following modifications to the manuscript:***

*Considering that tetrahydrofuran (THF) serves as the solvent in the APC electrolyte and $[MgCl\cdot5THF]^{+}$ species act as its active components, we selected $[MgCl\cdot5THF]^{+}$ and THF as two representative models for our calculations. Based on these models, we systematically compared the reaction thermodynamics on pure Mg and twinned Mg under*

*different electrode potentials (**Supplementary Figs. S29-S32**). At the same electrode potential, the Gibbs free energy for the reduction of [MgCl·5THF]$^+$ and THF on twinned Mg is significantly higher than that on pure Mg ($\Delta G_{twinned-Mg} > \Delta G_{pure-Mg}$), indicating that the twin structure intrinsically increases the energy barrier for the formation of interfacial passivation products (MgO and $MgCl_2$). In addition, for the same anode material, increasing the electrode potential markedly increases the Gibbs free energy of the reduction reaction, thereby suppressing interfacial side reactions. Notably, this "potential-interfacial reaction synergistic suppression effect" on twinned Mg provides clear thermodynamic evidence that twin structure is conducive to interfacial passivation suppression.* {Page 15, Line 12}

*4) As a result, the presented thermodynamic analysis does not yet convincingly explain why twin boundaries would suppress interphase formation. A clearer mechanistic framework linking twin structures to electrolyte decomposition processes is still needed.*

**Response:** We sincerely thank you for the important comment. In response, we have constructed a clearer mechanistic framework in the revised manuscript by integrating multiple experimental characterisations and theoretical calculations to elucidate the relationship between twin structures and electrolyte decomposition processes. The details are presented as follows.

First, XPS and TOF-SIMS results show that a large amount of MgO and $MgCl_2$ accumulates on the pure Mg electrode after cycling, with little change along the depth direction, indicating continuous electrolyte decomposition and growth of the interfacial passivation layer (**Figure R9a-d and Figure R10, 11**). In contrast, the 70-twinned Mg electrode shows much lower contents of MgO and $MgCl_2$, which gradually decrease with sputtering depth. In addition, after 10, 50, and 100 cycles, the surface composition of the twinned Mg electrode remains nearly unchanged, whereas the passivation products on pure Mg increase clearly with cycling. These results indicate that the twin structure changes the evolution of interfacial reactions and suppresses the accumulation of decomposition products.

Second, in situ EIS provides further kinetic evidence (**Figure R9e, f and Figure R12**). The interfacial resistance of the 70-twinned Mg electrode recovers to its initial value after current is reapplied and remains stable even after 100 cycles, suggesting fast charge transfer across the interface. In contrast, the resistance of the pure Mg electrode remains higher than the initial value and increases with cycling, indicating continuous growth of the passivation layer and slow ion transport.

More importantly, from a theoretical perspective, DFT calculations show that, at the same electrode potential, the Gibbs free energies for the reduction of [MgCl·5THF]$^+$ to $MgCl_2$ and THF to MgO are both higher on 70-twinned Mg than on pure Mg, indicating a higher energy barrier for electrolyte decomposition (**Figure R13, 14**). In addition, Marcus–Hush–Chidsey analysis shows that the overpotential of the twinned Mg electrode is close to the

reorganization energy of the $Mg^{2+}$ redox process, suggesting that reversible Mg deposition/stripping dominates with limited side reactions (**Figure R9g**). In contrast, the pure Mg electrode shows a larger deviation, indicating that electrolyte decomposition is dominant and leads to continuous interfacial degradation.

Finally, TEM observations show that the interfacial layer on twinned Mg is about 18 nm, while that on pure Mg is about 58 nm thick and porous (**Figure R9h, i**). These results confirm that the twin structure leads to a thinner and more stable interfacial layer.

Overall, the twin structure increases the energy barrier for electrolyte decomposition and promotes reversible Mg deposition, which suppresses side reactions and prevents continuous growth of the passivation layer.

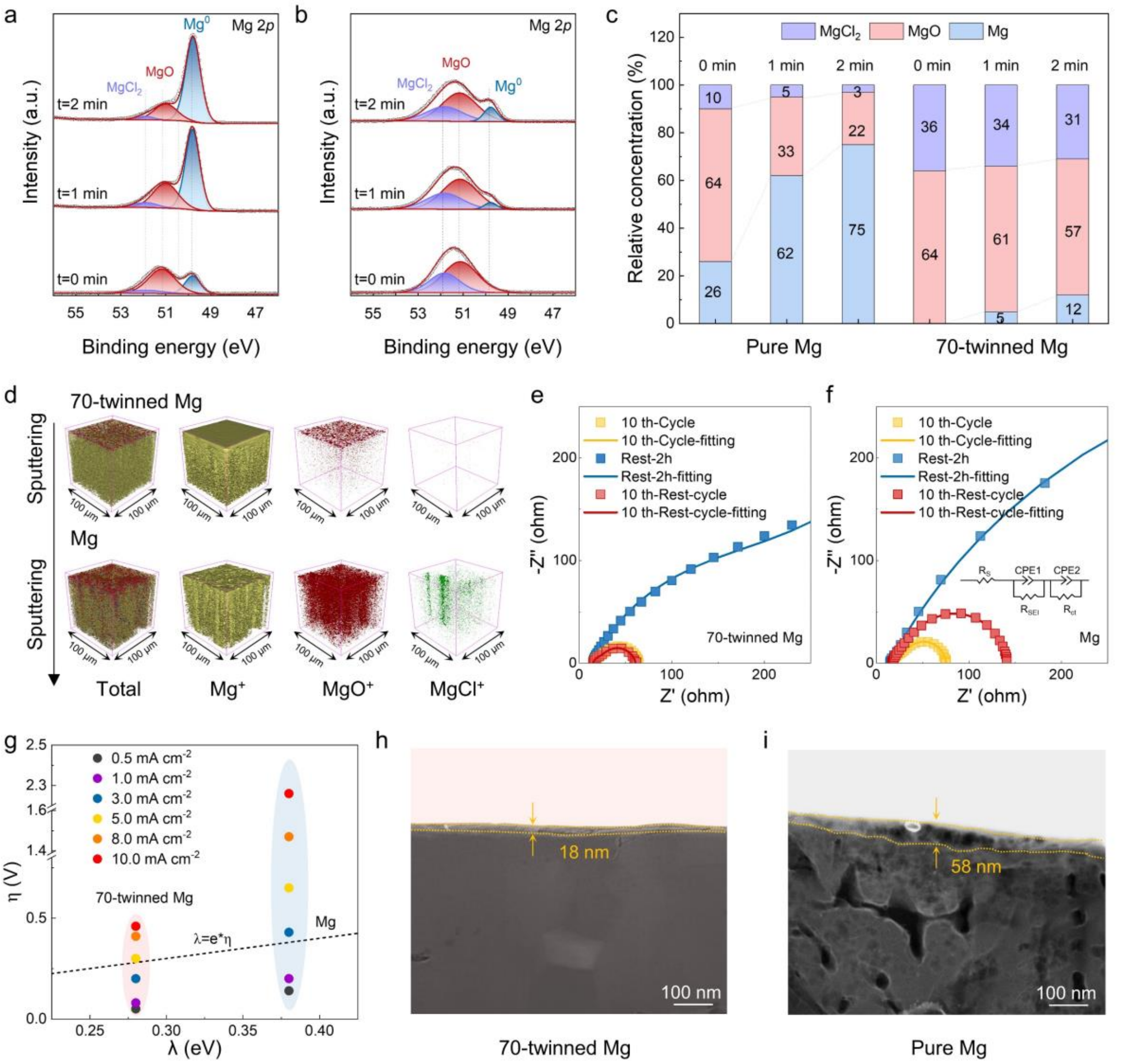


**Figure R9. Interface analysis and characterisation**. **a, b** High-resolution XPS spectra of Mg 2*p* of 70-twinned Mg (a) and pristine Mg (b) electrodes after cycling (10 cycles at 10 mA $cm^{-2}$). **c** The relative concentrations of decomposition species measured on the surface after cycling at sputtering depths of 0, 1 and 2 min. **d** 3D rendering for the $Mg^{+}$, $MgO^{+}$, and

$MgCl^+$ secondary ions and the overlay in the ToF-SIMS tested for the interfaces formed on the Mg and 70-twinned Mg electrodes. **e, f** The EIS spectra of a 70-twinned Mg||70-twinned Mg (e) and a Mg||Mg cell (f) cell measured after 10 cycling, resting for 2 h, and re-cycling period, under conditions of 10 mA $cm^{-2}$ and 1 mAh $cm^{-2}$. The inset corresponds to the fitted circuit. **g** The correlation between overpotential (η) and reorganization energy (λ) for different reaction modes of the Mg anodes. The dashed line represents when the η for electron transfer is equal to λ. **h, i** TEM images and of the interface and deoposited Mg after cycling for the 70-twinned Mg (h) and pure Mg (i) electrodes.

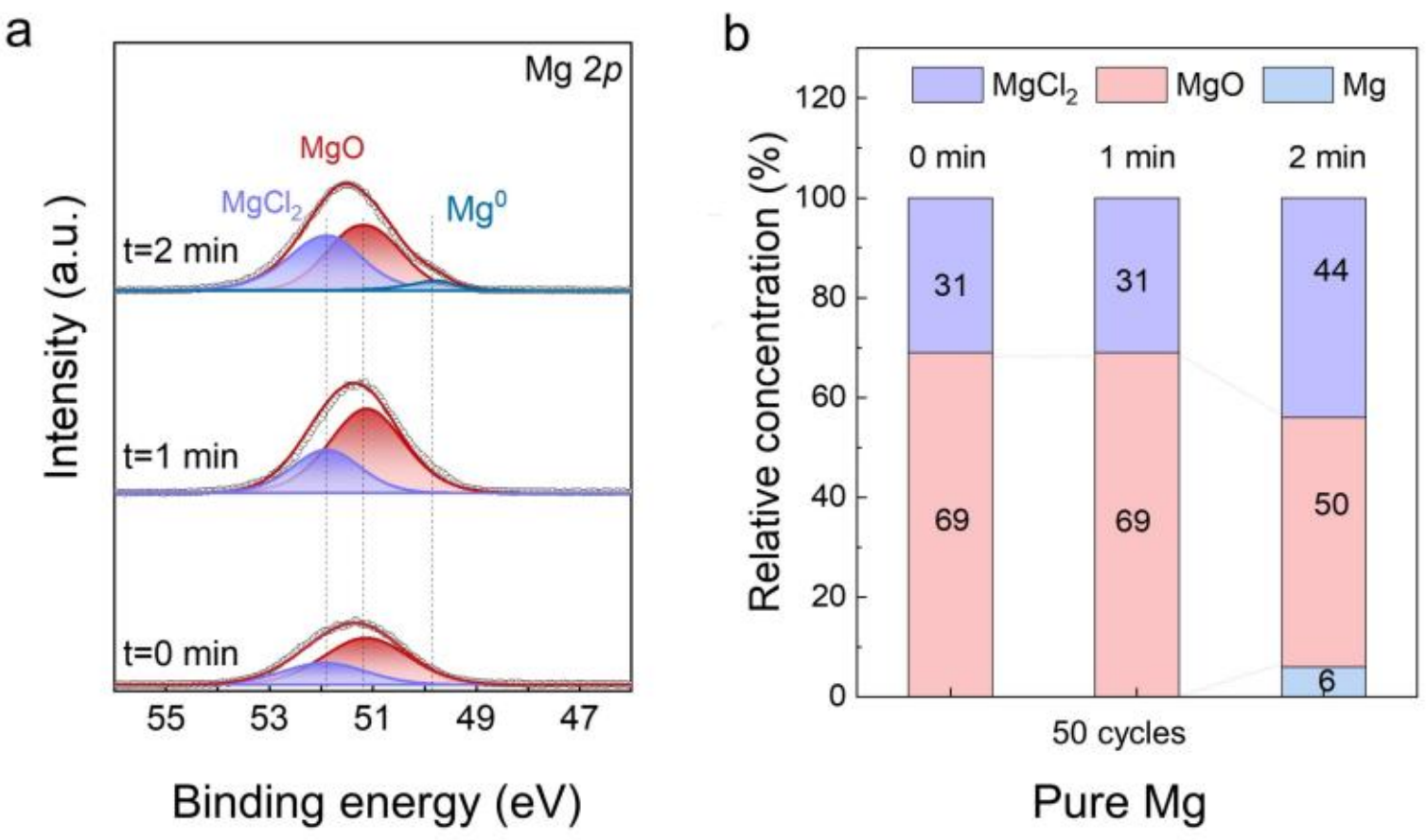


**Figure R10. a** High-resolution XPS spectra of Mg 2*p* of pure Mg electrodes after 50 cycling at 10 mA $cm^{-2}$, 5 mAh $cm^{-2}$. **b** The relative concentrations of decomposition species measured on the surface after 50 cycling at sputtering depths of 0, 1 and 2 min.

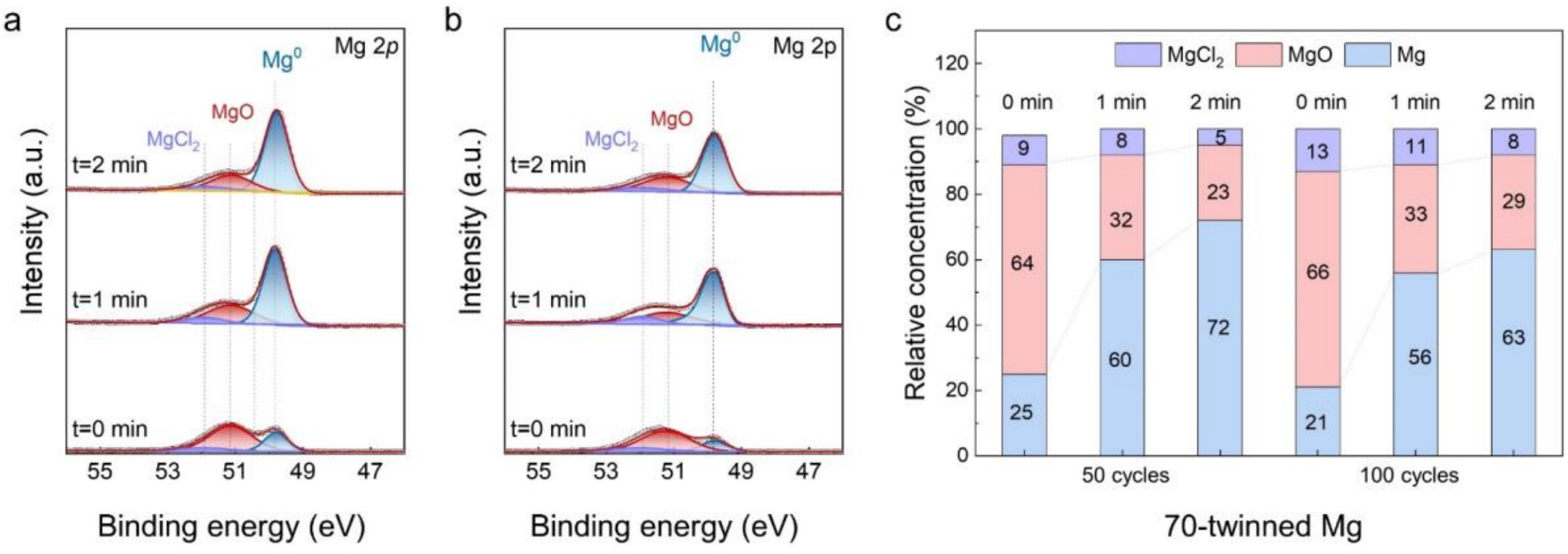


**Figure R11. a, b** High-resolution XPS spectra of Mg 2*p* of 70-twinned Mg electrodes afte 50 cycling (a) 100 cycling (b) at 10 mA $cm^{-2}$, 5 mAh $cm^{-2}$. **c** The relative concentrations of decomposition species measured on the surface after 50 and 100 cycling at sputtering depths of 0, 1 and 2 min.

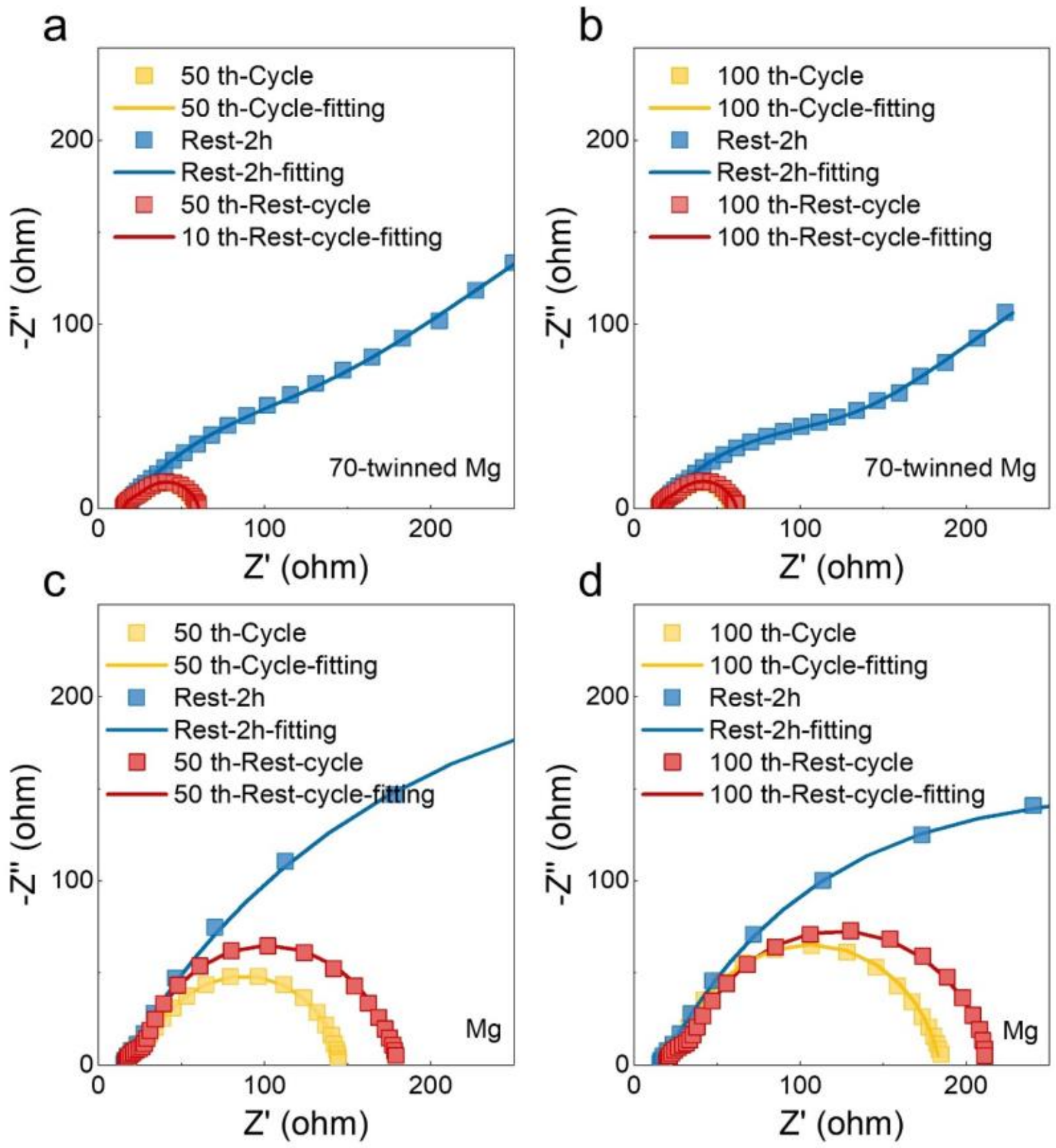


**Figure R12. a-d** The in situ EIS spectra of a 70-twinned Mg||70-twinned Mg (a, b) and a Mg||Mg cell (c, d) cell measured after 50 (a, c) and 100 (b, d) cycling, resting for 2 h, and re-cycling period, under conditions of 10 mA cm$^{-2}$ and 1 mAh cm$^{-2}$.

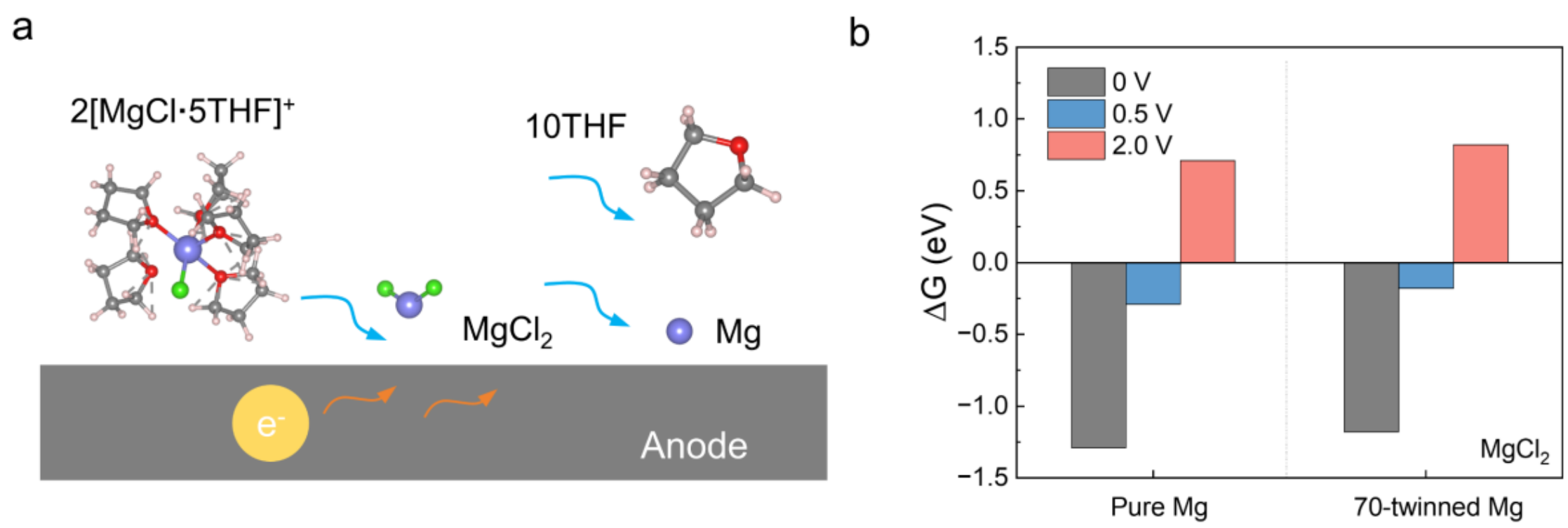


**Figure R13.** Theoretical calculation of the Gibbs free energy change of the reaction forming THF , adsorbed Mg or $MgCl_2$ on different anodes. a) Scheme of the calculation model (red, oxygen; green, chlorine; grey, carbon; white, hydrogen; and purple, Mg). The free energy calculation results. (The reason for choosing potentials of 0.5 V and 2.0 V is that the overpotential of a 70-twinned Mg symmetric cell at 10 mA cm$^{-2}$ is 0.43V (~0.5 V); while the overpotential of a pure Mg symmetric cell at 10 mA cm$^{-2}$ is 2.26V (~2.0 V)).

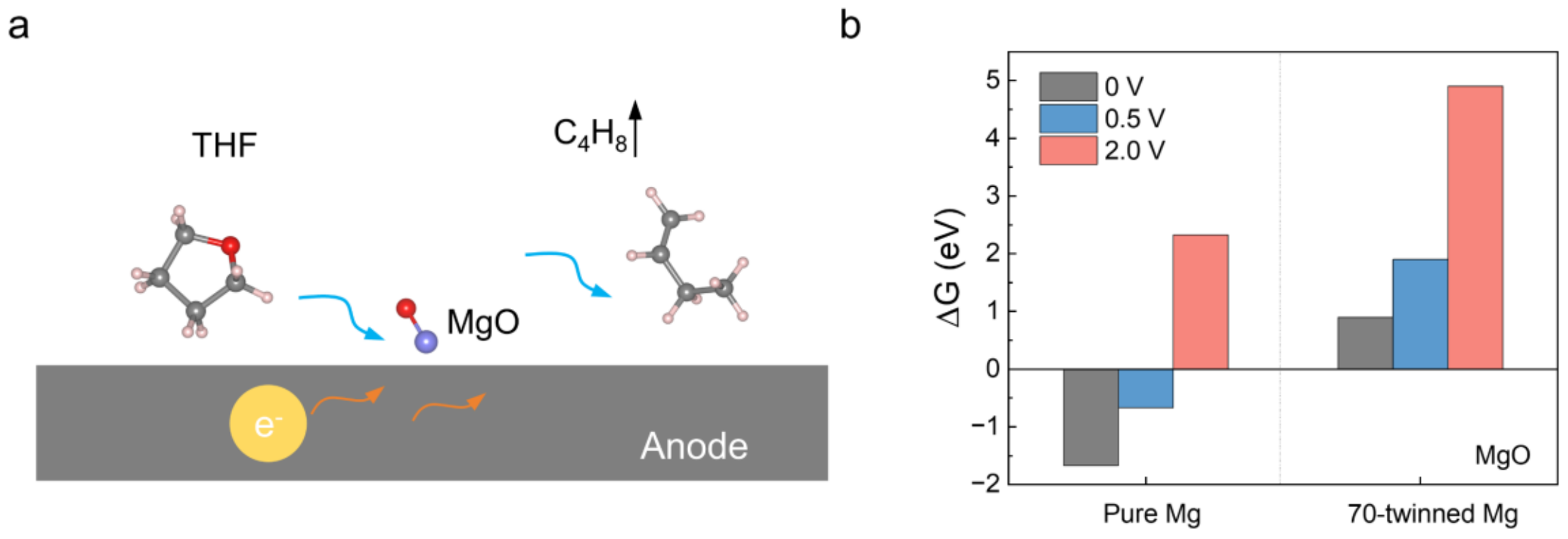


**Figure R14.**Theoretical calculation of the Gibbs free energy change of the reaction forming $C_4H_8$ or MgO on different anodes. a) Scheme of the calculation model (red, oxygen; grey, carbon; white, hydrogen; and purple, Mg); b) the free energy calculation results. (The reason for choosing potentials of 0.5 V and 2.0 V is that the overpotential of a 70-twinned Mg symmetric cell at 10 mA $cm^{-2}$ is 0.43V (~0.5 V); while the overpotential of a pure Mg symmetric cell at 10 mA $cm^{-2}$ is 2.26V (~2.0 V)).

***We have made the following modifications to the manuscript:***

*Considering that tetrahydrofuran (THF) serves as the solvent in the APC electrolyte and $[MgCl{\cdot}5THF]^+$ species act as its active components, we selected two representative model reactions: the reduction of $[MgCl{\cdot}5THF]^+$ to $MgCl_2$ and the reduction of THF to MgO. Based on these models, we systematically compared the reaction thermodynamics on pure Mg and twinned Mg under different electrode potentials (**Supplementary Figs. S29-S32**). At the same electrode potential, the Gibbs free energy for the reduction of $[MgCl{\cdot}5THF]^+$ and THF on twinned Mg is significantly higher than that on pure Mg ($\Delta G_{twinned\text{-}Mg} > \Delta G_{pure\text{-}Mg}$), indicating that the twin structure intrinsically increases the energy barrier for the formation of interfacial passivation products. In addition, for the same anode material, increasing the electrode potential markedly increases the Gibbs free energy of the reduction reaction, thereby suppressing interfacial side reactions. Notably, this "potential-interfacial reaction synergistic suppression effect" on twinned Mg provides clear thermodynamic evidence that twin structure is conducive to interfacial passivation suppression.* {Page 15, Line 12}

-----------------------------------------------------

**Reply to the report of referee #3**

-----------------------------------------------------

**Comment of Referee #3:**

Reviewer 3

*The authors have made an effort to address the concerns raised in the initial review, and some improvements are acknowledged. However, several of the fundamental electrochemical and methodological issues identified in the original report remain unresolved, and in some cases the revised manuscript has introduced additional inconsistencies. I still can not recommend this manuscript for publication.*

*The points in question are the following:*

*1) About point 1): the authors state that all chronoamperometric measurements have been re-performed in a three-electrode configuration, with pure Mg acting as quasi-reference electrode (QRE) and counter electrode (CE), and pure or twinned Mg as the working electrode (WE). The authors describe this configuration as an "asymmetric cell." In current electrochemical nomenclature, an asymmetric cell refers to a configuration in which WE and CE electrodes are composed of different materials. A configuration using Mg (normal or twinned) as WE, Mg as QRE, and Mg as CE is, by definition, a three-electrode symmetric cell. And this reflects a fundamental misunderstanding of electrochemical cell terminology. In addition, no cell diagram, is provided anywhere in the revised manuscript or Supporting Information. Cell configuration specifics (type of cell, separator, electrode surface, electrolyte volume), geometry and QRE placement play a very important role in such experiments, especially taking into account multivalent chemistries, like in the case of magnesium.*

**Response:** We sincerely thank you for the careful evaluation and for pointing out the issues related to electrochemical terminology and cell configuration. In the revised manuscript, we have carefully corrected and clarified these aspects.

First, we acknowledge that the term "asymmetric cell" was inaccurately used in our previous revision. According to standard electrochemical definitions, a configuration employing Mg (pure or twinned) as the working electrode (WE), Mg as the quasi-reference electrode (QRE), and Mg as the counter electrode (CE) should be properly described as a three-electrode symmetric configuration. We apologise for this imprecise terminology and have corrected it throughout the manuscript to avoid any ambiguity.

Second, regarding the experimental configuration, we appreciate your emphasis on the importance of cell geometry and electrode arrangement. All chronoamperometric measurements used for nucleation analysis and comparison with the Scharifker–Hills model

were performed in a well-defined three-electrode configuration using a Swagelok-type cell, and a schematic diagram has now been added (**Figure R15**). Specifically, the working electrode was either pure Mg foil or twinned-Mg foil, while both the counter and quasi-reference electrodes were pure Mg foils; All electrodes were polished with 3000# sandpaper and cut into 10 mm diameter discs. A glass fiber separator was used, and 200 μL of APC electrolyte was added to ensure sufficient wetting.

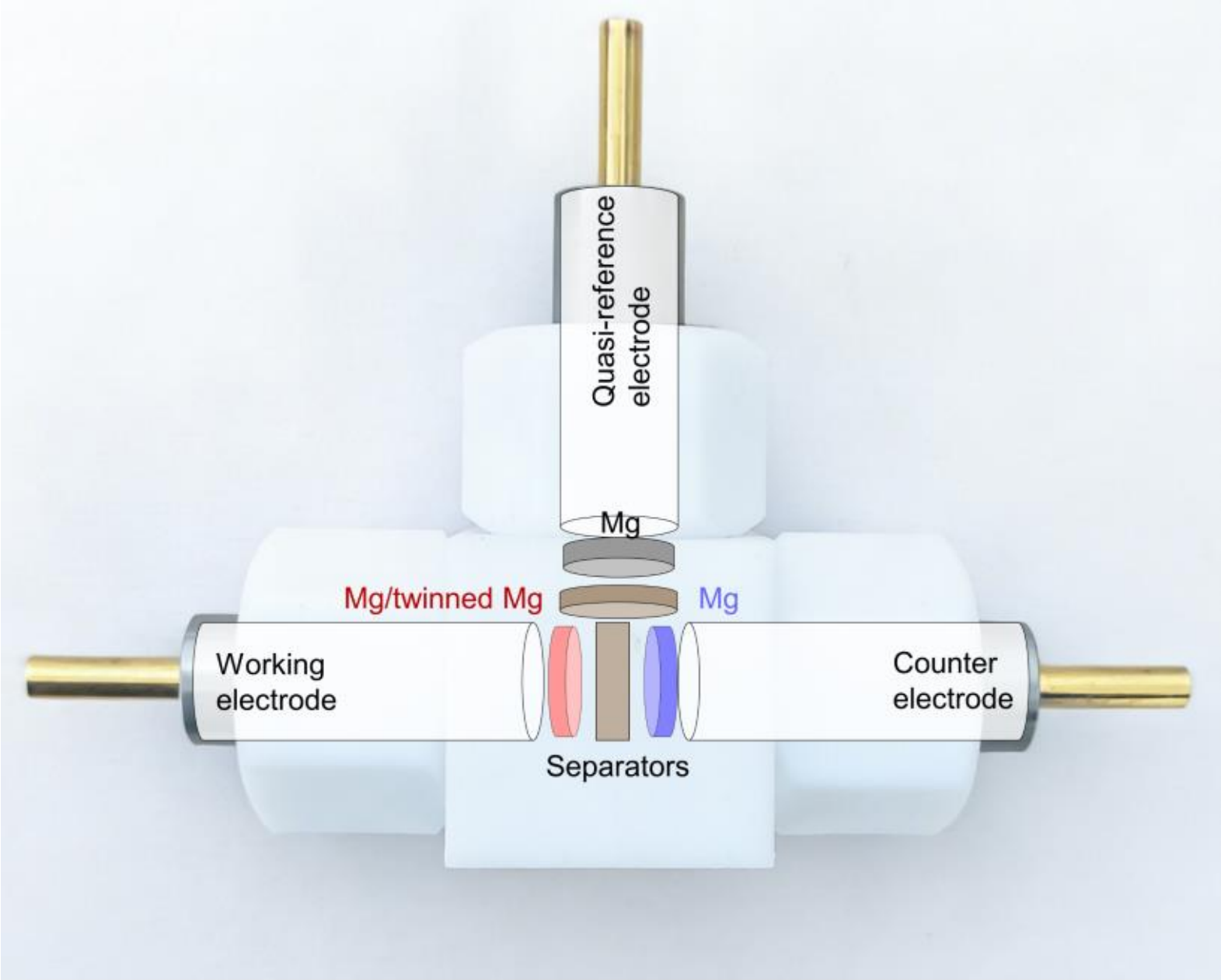


**Figure R15.** Schematic diagram of the Swagelok-type cell.

***We have made the following modifications to the manuscript:***

*Notably, all CA experiments discussed in this paper were conducted as symmetric cell tests under a strictly defined three-electrode configuration, using Swagelak-type cells. In addtion, the working electrode was either pure Mg foil or twinned-Mg foil, while both the counter and quasi-reference electrodes were pure Mg foils; Unless otherwise specified, all electrodes were polished with 3000# sandpaper and cut into 10 mm diameter discs. A glass fiber separator was used, and 200 μL of APC electrolyte was added to ensure sufficient wetting.* {Page 22, Line 8}

*2) Cell setup in point 1), has also another non-negligible consequence: since the Scharifker-Hills model was derived under specific theoretical boundaries, assuming a flat featureless and electrochemically inert substrate as WE (e.g. glassy carbon of a flat metal substrate), this assumption is violated here, since in a symmetric cell it Mg-on-Mg deposition is assumed. The model's assumption of hemispherical diffusion zone expansion on a planar surface is not representative of these substrates, in which there can be neither chemical inertness nor surface flatness. Surface roughness/ irregularities of the Mg surface, can dramatically change/direct nucleation processes from the early stages(repetitions) of such experiments.*

**Response:** We sincerely thank you for this technically important comment regarding the applicability of the Scharifker–Hills (S–H) model under the present experimental conditions. We agree that the original S–H model was derived based on idealised assumptions, including a flat, featureless, and electrochemically inert substrate, as well as hemispherical diffusion zone expansion [*Electrochimica acta, 1983, 28(7): 879-889*]. We also acknowledge that these assumptions are not strictly fulfilled in Mg-on-Mg deposition systems, where the substrate is both electrochemically active and inherently non-ideal in terms of surface morphology. To address this concern, we have carefully re-examined the role of substrate roughness and chemical activity in our system and have revised the manuscript accordingly to provide a more rigorous and transparent interpretation.

First, regarding surface morphology, we know that surface roughness and heterogeneity can influence early-stage nucleation behaviour by altering the local distribution of active sites and electric field. Therefore, in our original experiments, we did mechanically polish the Mg substrates with 3000# sandpaper to approximate the theoretical model as closely as possible. However, to further evaluate this effect, we have now introduced an additional control using a rougher Mg surface (400# polishing) and performed identical chronoamperometric measurements (**Figure R16 and R17**). The results show that increased surface roughness indeed leads to a more pronounced deviation from the ideal S–H theoretical curves. However, this deviation does not alter the qualitative identification of nucleation modes: the pure Mg electrode consistently exhibits behaviour characteristic of three-dimensional instantaneous nucleation (3DI), whereas the twinned Mg electrode follows a three-dimensional progressive nucleation (3DP) trend. This indicates that while surface roughness affects the degree of agreement with the ideal model, it does not invalidate the comparative analysis of nucleation mechanisms in our study.

Second, concerning the electrochemical activity of the Mg substrate, we agree that Mg cannot be considered an inert substrate, and this represents a deviation from the original assumptions of the S–H model. However, in the context of this work, the use of an active Mg substrate is intentional and essential, as the objective is to investigate Mg deposition behaviour under realistic battery-relevant conditions. Employing an inert substrate (e.g., glassy carbon) would eliminate the intrinsic interfacial chemistry and thus fail to capture the true deposition characteristics of Mg metal anodes. In addition, both the pure Mg and twinned Mg electrodes were evaluated under identical active conditions; therefore, the influence of substrate reactivity is internally consistent across all comparative analysis.

Furthermore, we note that the application of the S–H model to non-ideal or electrochemically active substrates has been widely reported in the literature, including studies on multivalent batteries (such as Zn metal systems) using symmetric-cell configurations (**Table R1 and Figrue R18-24**). These works generally recognise that, under such conditions, the S–H model should be interpreted as a qualitative tool for analysing early-stage nucleation trends

rather than a strictly quantitative description. In line with this understanding, we have revised the manuscript to explicitly state the limitations of the model and to avoid overinterpretation of the fitting results.

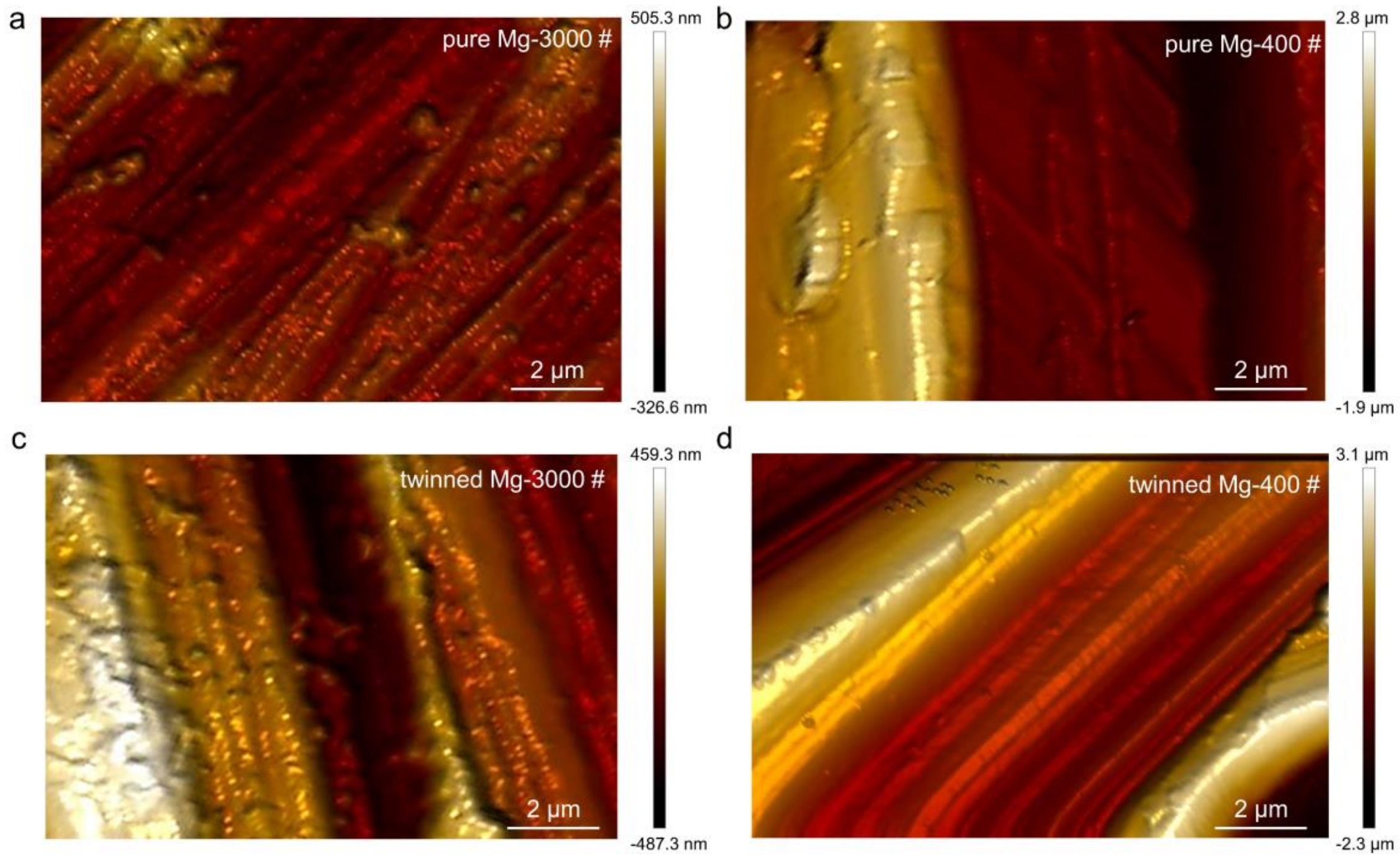


**Figure R16. a-d** Mg and twinned Mg substates with different roughness.

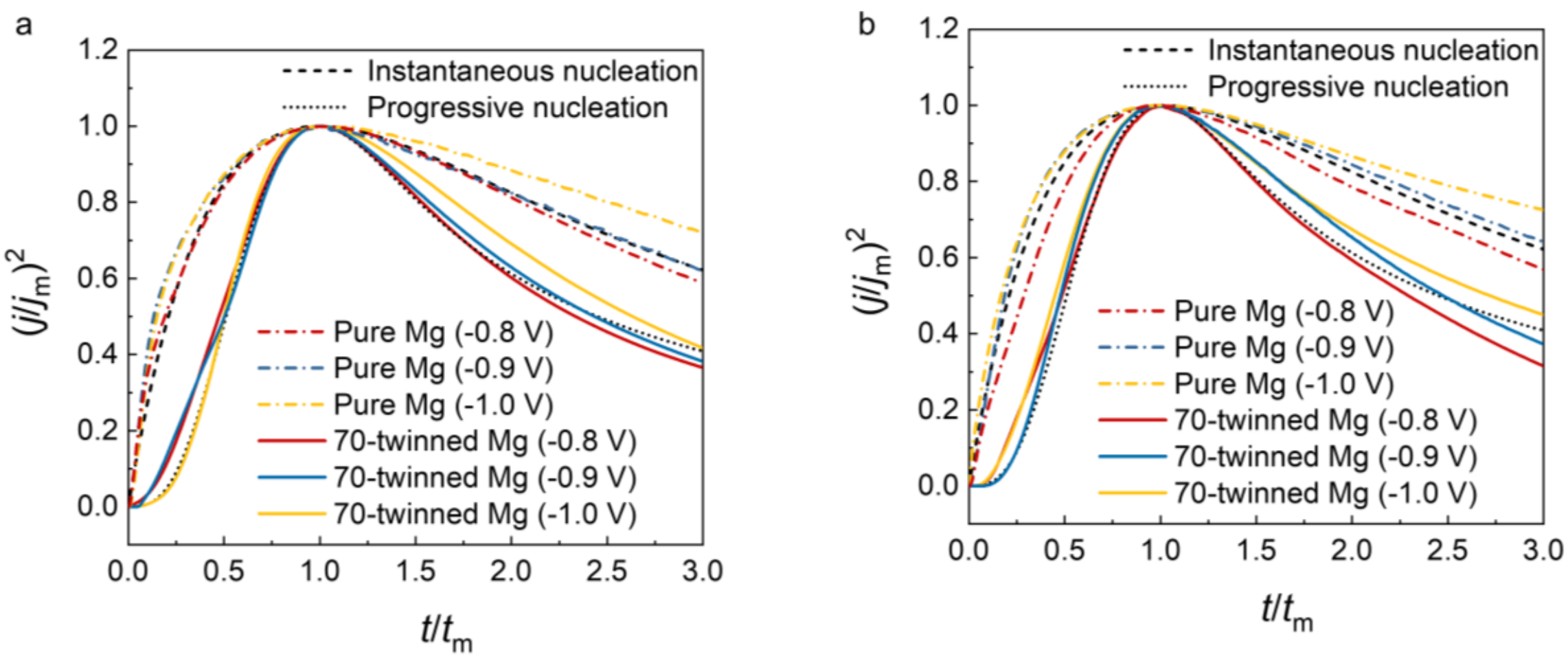


**Figure R17.** Plots of $t/t$m and $(j/j_{\mathrm{m}})^2$ in comparison with theoretical 3D models for a Mg cell using Mg and 70-twinned Mg electrodes with (a) 3000# roughness and (b) 400# roughness.

**Table R1.** Application statistics of the S-H model

| Articles | Working | Counter | Quasi-Reference |
|---|---|---|---|
| Nat. Commun. 2023, 14, 7080 | Zn/Zn-Ti alloy | Pt foil | Ag/AgCl |
| J. Am. Chem. Soc. 2025, 147, 35, 32216–32224 | Zn/Zn power-Sn foil (APS) | Pt | Ag/AgCl |
| Joule 2024, 8, 3054–3071 | Zn/MI-on-Zn | Zn/MI-on-Zn | Two electrode |
| Angew. Chem. 2025, 137, e202514181 | Zn/Ni@DPA-Zn | Zn/Ni@DPA-Zn | Two electrode |
| Nat. Commun. 2026, 17, 3549 | Zn | Zn | Two electrode |
| Adv. Funct. Mater. 2025, e22185 | Mg, Mg-Mn, Mg-Gd, Mg-Gd-Mn | Mg,Mg-Mn,Mg-Gd, Mg-Gd-Mn | Two electrode |

**[Figure Redacted]**

**Figure R18.** Relevant test parameters in recently published articles.

[Figure Redacted]

**Figure R19.** (a) Current-time transients obtained at predetermined potentials. (b) Experimental dimensionless transients in comparison with the theoretical 3D nucleation models.{Nat. Commun. 2023, 14:7080}.

[Figure Redacted]

**Figure R20.** Experimental dimensionless transients in comparison with the theoretical 3D nucleation models. {J. Am. Chem. Soc. 2025, 147, 35, 32216–32224}.

[Figure Redacted]

**Figure R21.** (a) Dimensionless experimental transients compared with theoretical 3D nucleation models. (b) Current-time transients obtained at predetermined potentials. {Joule 2024, 8, 3054–3071}.

**[Figure Redacted]**

**Figure R22.** (a) The tested CA curve and the (b) comparison of experimental dimensionless curves with theoretical nucleation models. {Angew. Chem. 2025, 137, e202514181}.

**[Figure Redacted]**

**Figure R23.** Normalized current–time transients at different applied potentials, compared with classical nucleation models. {Nat. Commun. 2026,17, 3549}

**[Figure Redacted]**

**Figure R24.** (a) Current-time transients obtained at predetermined potentials, (b) The fitted CA curve.{Adv. Funct. Mater. 2025, e22185}.

***We have made the following modifications to the manuscript:***

*Notably, S–H model was derived based on idealised assumptions. However, in the Mg-on-Mg deposition systems, these assumptions are not not strictly fulfilled because the substrate inherently has non-idealized surface morphology. Therefore, it may cause a certain deviation between the experimental transient and the ideal S-H curve. To address this concern, we compared the Mg electrode with a rougher surface treatment (400# polished) and the 3000# polished Mg electrode mentioned in the main text, and performed identical CA measurements. The results show that increased surface roughness indeed leads to a more pronounced deviation from the ideal S–H theoretical curves. However, this deviation does not alter the qualitative identification of nucleation modes: the pure Mg electrode consistently exhibits behaviour characteristic of three-dimensional instantaneous nucleation (3DI), whereas the twinned Mg electrode follows a three-dimensional progressive nucleation (3DP) trend. This indicates that while surface roughness affects the degree of agreement with the ideal model, it does not invalidate the comparative analysis of nucleation mechanisms in our study.* {Supporting information Page 51, Line 20}

*3) The CA transients in Supplementary Fig. R28 and R31 (Rebuttal Letter), report the cathodic current density values,— which are negative by IUPAC convention — on the positive y-axis quadrant but as negative numbers, with the axis labelled simply as "Current density (mA cm⁻²)." This is inconsistent and does not conform to standard electrochemical reporting practice. Plotting the absolute values as positive, with the axis explicitly labelled "−j / mA cm⁻²" would have been the correct way, and this signals an actually concerning way of handling data for such important experiments.*

**Response:** We sincerely thank the reviewer for this careful observation. The manuscript and Supporting Information have been carefully revised to ensure that all figures now follow consistent and standardised electrochemical conventions. (**Figure R25**).

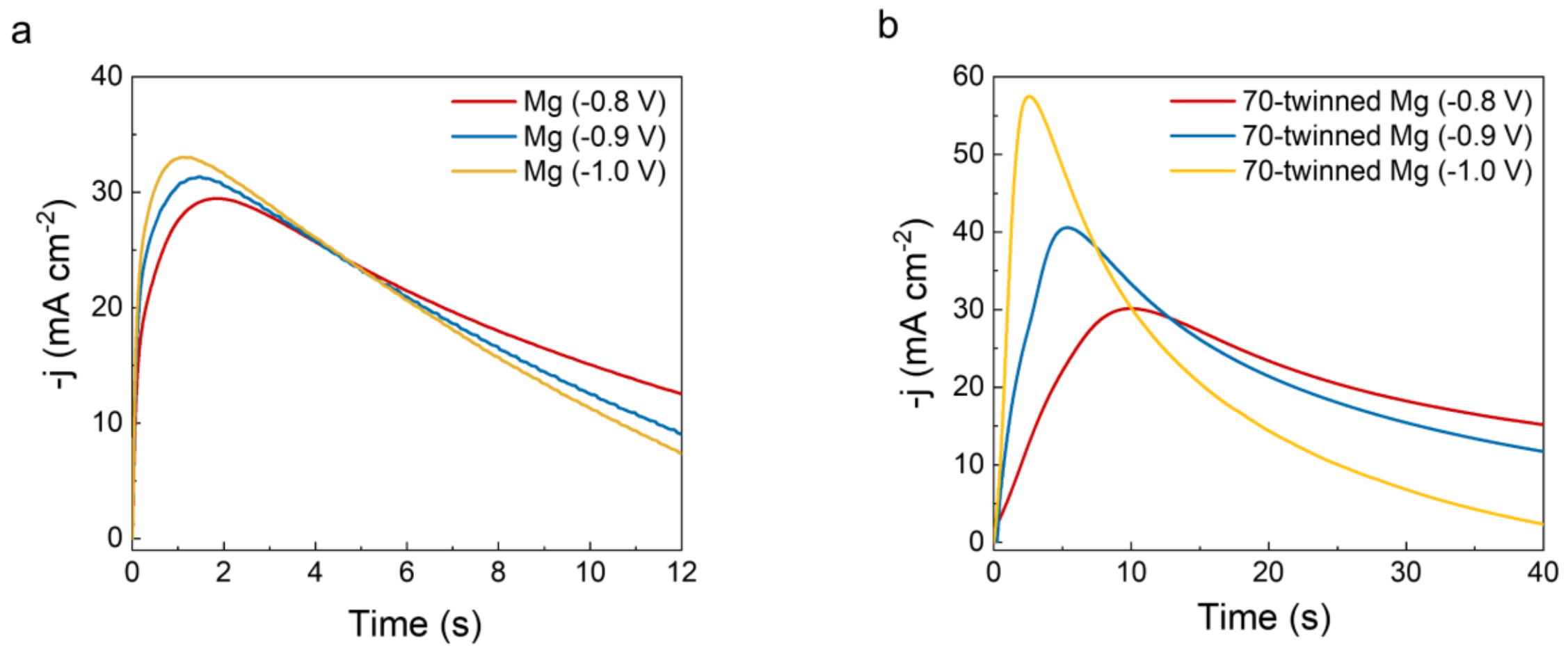


**Figure R25.** Current-time transients obtained at predetermined potentials.

*4) Regarding point 2) he authors invoke -layer charging as one of the explanations for early-time deviations from the theoretical S-H curves. This explanation is incorrect at the involved timescales of these nucleation experiments. Double-layer charging is complete within milliseconds of the potential step — orders of magnitude faster than the tmax reported values (1.81–10.01 s). At the timescale of tmax, capacitive contributions are negligible and are usually subtracted during data processing. Recalling a fast process to explain behavior at the seconds timescale is not valid and does not address the discrepancy identified above.*

**Response:** We sincerely thank you for your important clarification. We agree that double-layer charging is a rapid process, and therefore it is inaccurate to use it to explain the deviation from the S-H theoretical curve in the early stages. Therefore, we have completely removed this explanation from the revised manuscript.

In addition, we have re-examined the origin of the deviations between experimental transients and the ideal Scharifker–Hills curves from a more physically relevant perspective. Specifically, we attribute the observed differences to this non-ideal property of the active Mg electrode surface roughness, which may affect nucleation behavior. To demonstrate this point, we have conducted additional experiments by varying the surface roughness of the Mg substrate. In the original experiments, the Mg electrodes were polished using 3000# sandpaper to obtain a relatively smooth surface. In the revised study, we introduced a rougher surface prepared using 400# polishing and performed identical CA measurements. The results show that increasing surface roughness leads to a more pronounced deviation from the ideal S–H theoretical curves (**Figure R26 and R27**).

Importantly, despite these deviations, the qualitative identification of nucleation modes remains unchanged: the pure Mg electrode consistently exhibits characteristics of three-dimensional instantaneous nucleation, whereas the twinned Mg electrode follows a three-dimensional progressive nucleation behaviour. This indicates that while surface roughness affect the accuracy of the model, they do not compromise the validity of the comparative mechanistic conclusions.

***We have made the following modifications to the manuscript:***

*Notably, S–H model was derived based on idealised assumptions. However, in the Mg-on-Mg deposition systems, these assumptions are not not strictly fulfilled because the substrate inherently has non-idealized surface morphology. Therefore, it may cause a certain deviation between the experimental transient and the ideal S-H curve. To address this concern, we compared the Mg electrode with a rougher surface treatment (400# polished) and the 3000# polished Mg electrode mentioned in the main text, and performed identical CA measurements. The results show that increased surface roughness indeed leads to a more pronounced deviation from the ideal S–H theoretical curves. However, this deviation does not alter the qualitative identification of nucleation modes: the pure Mg*

*electrode consistently exhibits behaviour characteristic of three-dimensional instantaneous nucleation (3DI), whereas the twinned Mg electrode follows a three-dimensional progressive nucleation (3DP) trend. This indicates that while surface roughness affects the degree of agreement with the ideal model, it does not invalidate the comparative analysis of nucleation mechanisms in our study.* {Supporting information Page 51, Line 20}

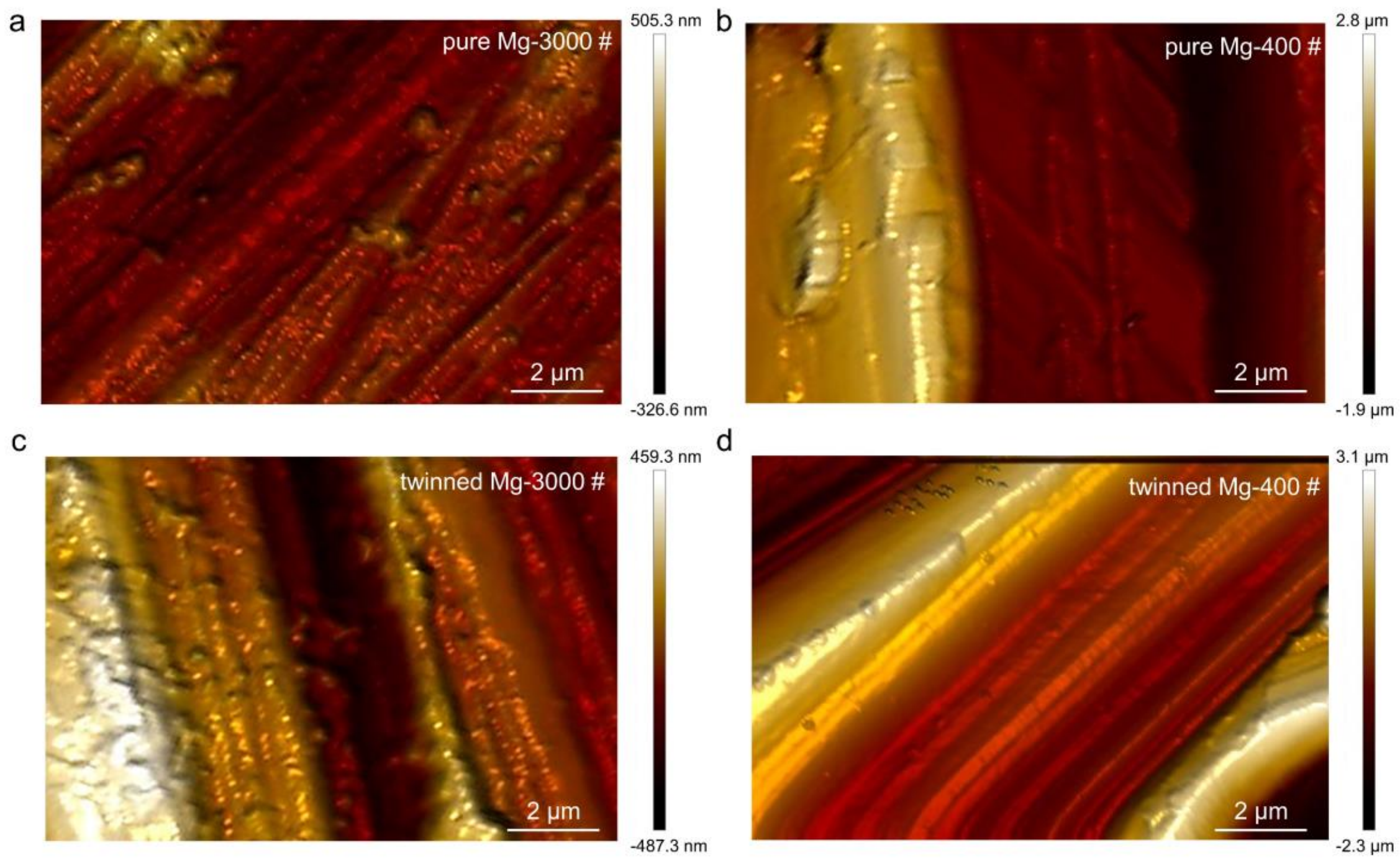


**Figure R26. a-d** Mg and twinned Mg substates with different roughness.

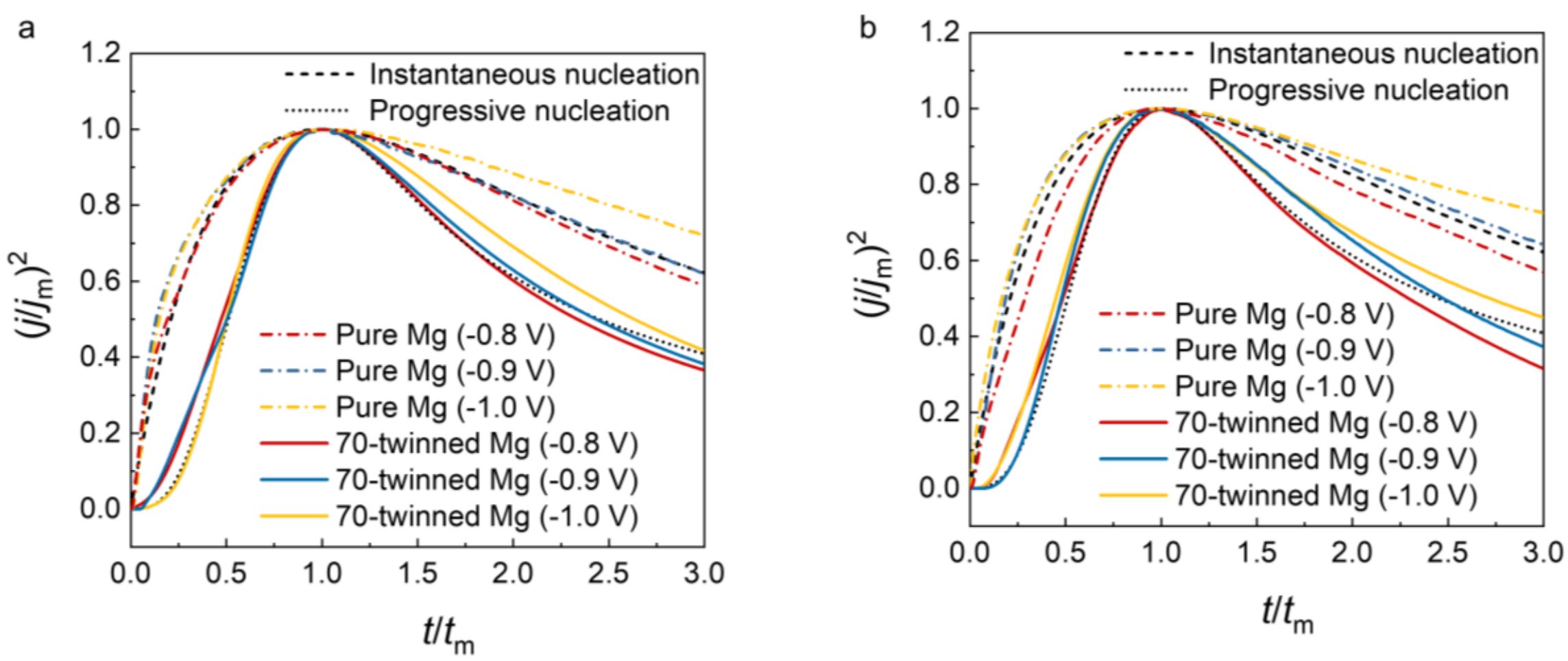


**Figure R27.** Plots of $t/t$m and $(j/j_m)^2$ in comparison with theoretical 3D models for a Mg cell using Mg and 70-twinned Mg electrodes with (a) 3000# roughness and (b) 400# roughness.

*5) In response to point 3) of the original review, the authors added COMSOL simulations to argue that 3DI-like nucleation, combined with limited surface diffusion, promotes morphological instability that ultimately evolves into dendritic structures. It is well*

*established in literature — including in work cited by the authors themselves (Davidson et al., ACS Energy Lett. 2019) — that Mg dendrite formation is not a general consequence of electrodeposition. Dendritic growth in Mg is observed only under specific conditions of very high current density, where mass transport limitations become so severe to drive tip-enhanced growth instabilities. Under the current densities employed in this manuscript, Mg can be electroplated in irregular but non-dendritic morphologies — granular, mossy, or lumpy — which are distinct from classical dendrites. Indeed, the relative resistance of Mg to dendrite formation compared to lithium is cited as one of the material's advantages in the paper's own introduction. A single COMSOL simulation can not demonstrate how the system will evolve, specifically into dendrites under the current experimental conditions. Morphological inhomogeneity is not equivalent to dendritic growth. So the claim that 3DI-like nucleation characteristics „promote the evolution of dendritic structures" remains unsupported by the experimental conditions used and contradicted by the established behavior of Mg electrodeposition in the literature.*

**Response:** We sincerely thank you for this critical and thoughtful comment. We acknowledge that our previous wording overstated the evolution of the deposition morphology and did not clearly distinguish between general morphological instability and true dendritic growth in Mg systems. The manuscript has been carefully revised to correct this issue and to ensure consistency with the established understanding of Mg electrodeposition.

Specifically, we acknowledge that dendritic growth in Mg is not a universal result of electrodeposition, typically occurring only under extreme conditions (e.g., extremely high current densities), where severe mass transfer constraints induce tip-enhanced growth [*ACS Energy Lett., 2018, 4(2): 375-376*]. At the relatively moderate current densities used in this study, Mg deposition should more accurately be described as forming irregular morphologies, which are fundamentally different from typical dendrites. We also fully agree that, as stated in the introduction, the relatively low dendritic tendency of Mg compared to Li is an important and recognized advantage.

Therefore, we have revised the manuscript, removing the previous statement that "3DI-type nucleation promotes the evolution of dendritic structures." Instead, we now describe more precisely the effect of 3DI-type nucleation on morphological uniformity. The COMSOL simulation results are now rigorously interpreted as follows: the initial localized nucleation distribution (a characteristic of 3DI behavior) leads to enhanced local current density and ion flux inhomogeneities, thereby exacerbating interface roughening and growth inhomogeneities.

***We have made the following modifications to the manuscript:***

*COMSOL simulations (Supplementary Fig. S24) further demonstrate that such 3DI-like nucleation characteristics, these initial inhomogeneities are prone to being magnified during subsequent growth, thereby increasing morphological instability*. {Page 13, Line 7}

*6) The response fo the authors to point 4) does not address the substance of the comment. The concern raised was specifically methodological — that the same invalid approach to the S-H model and the same absence of rigorous potential control was used in a prior accepted high impact publication. Describing differences in battery chemistry or deformation strategy does not resolve whether the prior paper's nucleation analysis was valid. This remains an open concern.*

**Response:** We sincerely thank the reviewer for once again providing critical comments on the methodological aspects of the Scharifker–Hills model analysis, and we respond as follows.

First, to enhance the methodological rigor of this manuscript, we have introduced a three-electrode configuration in the revised version; and calculated a diagnostic parameter ($t_{max}*(j_{max})^2$) to verify the applicability of the model; at the same time, we systematically investigated the influence of electrode surface roughness on nucleation behavior. These improvements ensure that the conditions required for applying the S–H model are more appropriately satisfied when analyzing the nucleation mechanism.

Second, we would like to clarify that the S-H model analysis in our previously published papers and this manuscript is only used as supplementary evidence. The main conclusions (e.g., twinned electrodes have higher diffusion rates and can reduce surface passivation during cycling) do not depend on these analytical results. Meanwhile, the role of the S–H model-related tests in this paper is limited to assisting the interpretation of SEM observations, specifically indicating that the twinned structure promotes a more uniform Mg deposition. Furthermore, regarding the current application of the S–H model, we note that it is indeed widely used in the literature on electrodeposition nucleation, with experimental conditions spanning both two- and three-electrode systems, as well as active working electrodes (**Table R2 and Figure R28-34**).

Thank you again for your time and expertise in the S-H model section of this work.

**Table R2.** Application statistics of the S-H model

| Articles | Working | Counter | Quasi-Reference |
| --- | --- | --- | --- |
| Nat. Commun. 2023, 14, 7080 | Zn/Zn-Ti alloy | Pt foil | Ag/AgCl |

| | | | |
|---|---|---|---|
| J. Am. Chem. Soc. 2025, 147, 35, 32216–32224 | Zn/Zn power-Sn foil (APS) | Pt | Ag/AgCl |
| Joule 2024, 8, 3054–3071 | Zn/MI-on-Zn | Zn/MI-on-Zn | Two electrode |
| Angew. Chem. 2025, 137, e202514181 | Zn/Ni@DPA-Zn | Zn/Ni@DPA-Zn | Two electrode |
| Nat. Commun. 2026, 17, 3549 | Zn | Zn | Two electrode |
| Adv. Funct. Mater. 2025, e22185 | Mg, Mg-Mn, Mg-Gd, Mg-Gd-Mn | Mg,Mg-Mn,Mg-Gd, Mg-Gd-Mn | Two electrode |

**[Figure Redacted]**

**Figure R28.** Relevant test parameters in recently published articles.

**[Figure Redacted]**

**Figure R29.** (a) Current-time transients obtained at predetermined potentials. (b) Experimental dimensionless transients in comparison with the theoretical 3D nucleation models.{Nat. Commun. 2023, 14:7080}.

**[Figure Redacted]**

**Figure R30.** Experimental dimensionless transients in comparison with the theoretical 3D nucleation models. {J. Am. Chem. Soc. 2025, 147, 35, 32216–32224}.

**[Figure Redacted]**

**Figure R31.** (a) Dimensionless experimental transients compared with theoretical 3D nucleation models. (b) Current-time transients obtained at predetermined potentials. {Joule 2024, 8, 3054–3071}.

**[Figure Redacted]**

**Figure R32.** (a) The tested CA curve and the (b) comparison of experimental dimensionless curves with theoretical nucleation models. {Angew. Chem. 2025, 137, e202514181}.

**[Figure Redacted]**

**Figure R33.** Normalized current–time transients at different applied potentials, compared with classical nucleation models. {Nat. Commun. 2026,17, 3549}

**[Figure Redacted]**

**Figure R34.** (a) Current-time transients obtained at predetermined potentials, (b) The fitted CA curve.{Adv. Funct. Mater. 2025, e22185}.

----------------------------------------------------

**Reply to the report of referee #4**

----------------------------------------------------

**Comment of Referee #4:**

Reviewer 4

*The new version of the manuscript describes a twin-engineering strategy for magnesium metal anodes that uses deformation to allow highly reversible Mg electrodeposition at high current density and areal capacity. The authors illustrate enhanced surface diffusion, inhibited parasitic reactions, and consistent planar deposition by introducing a high density of coherent twins.*

*The manuscript integrates electrochemical analysis, structural characterization, and DFT calculations to present a coherent mechanistic framework linking twin boundaries to enhanced Mg deposition behavior. The electrochemical performance and long-term cycling stability are especially impressive, demonstrating that Mg metal anodes have made a real step forward.*

*The authors have satisfactorily addressed the comments from the previous review and have clarified several points regarding the mechanistic interpretation and the scope of the work.*

*I think the manuscript is good enough to be published in Nature Communications.*

**Response:** We appreciate the reviewer's positive comments on our manuscript and recommendation for publication.
